# Iron-catalysed direct coupling of organosodium compounds

**DOI:** 10.1038/s44160-025-00771-1

**Published:** 2025-03-25

**Authors:** Ikko Takahashi, Andreu Tortajada, David E. Anderson, Laurean Ilies, Eva Hevia, Sobi Asako

**Affiliations:** 1 RIKEN Center for Sustainable Resource Science, Wako, Japan; 2 Department für Chemie, Biochemie und Pharmacie, Universität Bern, Bern, Switzerland

**Keywords:** Catalysis, Sustainability, Synthetic chemistry methodology, Cross-coupling reactions, Reaction mechanisms

## Abstract

Sodium is one of the most abundant elements on Earth and a sustainable alternative to less sustainable metals such as lithium, which is becoming increasingly depleted and expensive. Traditionally, however, organosodium reagents have been considered highly reactive, engaging in uncontrollable reactions, and as a result, they have been scarcely used in organic synthesis, especially in combination with transition-metal catalysis. Here we report the use of organosodium compounds as C(*sp*^2^)–Na nucleophilic partners in iron-catalysed oxidative homocoupling and cross-coupling with alkyl halides. Mechanistic investigations based on the preparation and characterization of putative organoiron intermediates reveal that a bidentate additive coordinates both sodium and the iron centre, exerting control over the catalytic reactivity. This combination of two abundant and non-toxic metals, powered by molecular-level mechanistic understanding, is expected to open new avenues for the use of sustainable organometallic reagents in organic synthesis.

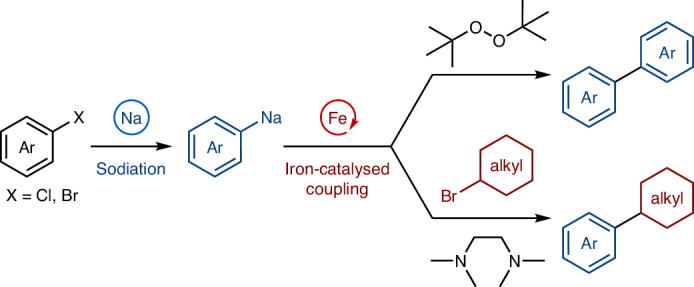

## Abstract (web summary)

Despite the abundance and non-toxic nature of sodium, organosodium reagents have rarely been used in organic synthesis. Now iron-catalysed homocoupling and C(*sp*^2^)–C(*sp*^3^) cross-coupling reactions of arylsodium compounds are reported. The observed C(*sp*^2^)–C(*sp*^3^) cross-coupling reactivity of arylsodium reagents is enabled by Lewis donor additive *N*,*N*,*N*′,*N*′-tetramethylethylenediamine.

## Main

**Fig. 1:**
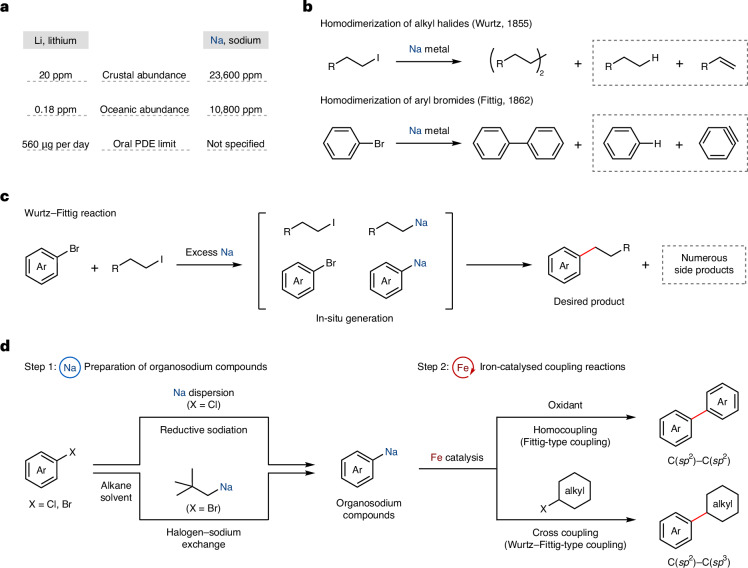
The use of sodium for carbon–carbon bond formation in organic synthesis. **a**, The abundance and toxicity of sodium^1,2^. **b**, Sodium-mediated carbon–carbon bond formation through homodimerization^4–6^. **c**, Wurtz–Fittig reaction of aryl bromides with alkyl iodides using sodium metal^7,8^. **d**, This work: merger of organosodium chemistry and iron catalysis for oxidative homocoupling and cross-coupling with alkyl halides. PDE, permissible daily exposure; Ar, aryl.

Sodium is the most abundant alkali metal, and the resources of sodium on Earth (23,600 ppm in the crust, together with a large amount in the ocean) are substantially greater than those of lithium (20 ppm in the crust; Fig. 1a)^1^. Moreover, sodium is largely non-toxic: whereas the amount of permissible daily exposure to lithium in drugs is specified (560 μg per day), sodium is not subjected to such regulations^2^. Thus, sodium is an ideal metal for sustainable and environmentally benign organic synthesis. For over half a century, transition-metal-catalysed coupling reactions have revolutionized organic synthesis, enabling the precise and efficient creation of carbon–carbon bonds^3^. Notably, the historical beginnings of coupling reactions did not require the use of a precious transion metal and relied on sodium-mediated approaches such as homodimerization of alkyl halides (1855, the Wurtz reaction)^4,5^ or aryl halides (1862, the Fittig reaction)^6^ in the presence of a superstoichiometric amount of sodium metal (Fig. 1b) and cross-coupling of alkyl iodides with aryl bromides mediated by an excess amount of sodium to produce alkylarenes (the Wurtz–Fittig reaction; Fig. 1c)^7,8^. Despite these initial discoveries and the merits of sodium, the subsequent explosive development of transition-metal-catalysed coupling reactions has relied on various other organometallic reagents^9^, and the utilization of organosodium compounds has been largely neglected in modern organic synthesis^10^. This situation has been caused mainly by the higher ionic character of the C–Na bond as compared with other metals such as boron, zinc, magnesium and lithium, inducing uncontrollably high reactivity. As a result, the high reactivity of organosodium leads to non-selective reactions generating many undesired by-products. In addition, their high ionic character leads to the formation of highly aggregated structures, making organosodium compounds sparingly soluble in non-polar solvents. This, combined with their instability in ethereal solvents, makes their manipulation as well as the isolation of key reaction intermediates and mechanistic analysis particularly difficult.

Another reason for the slow development of organosodium chemistry has been the lack of efficient synthetic methods. Recently, there has been a surge of interest in the preparation of organosodium compounds^11–14^, alleviating the long-standing prejudice that organosodium compounds cannot be of synthetic utility, but applications have still been limited to a reduced number of organic transformations. Nevertheless, a handful of reports have shown that organosodium reagents are amenable to transition-metal catalysis. Thus, a few studies have reported the direct use of (hetero)arylsodiums in Pd- and Ni-catalysed C(*sp*^2^)–C(*sp*^2^) cross-coupling for the construction of biaryls^15,16^ and polythiophenes^17,18^, respectively, although these approaches may suffer from issues associated with sustainability or toxicity of the catalysts. Meanwhile, the use of organosodium reagents in iron-catalysed C–C bond formation processes remains unexplored. Breaking new ground in this evolving area of research, we report here the integration of organosodium chemistry with sustainable transition-metal catalysis, specifically the direct coupling of aryl and alkenyl organosodium compounds using abundant, non-toxic and environmentally benign iron as a catalyst (Fig. 1d)^19–22^. Furthermore, mechanistic studies based on the preparation and characterization of organoiron complexes and radical-clock experiments provided important insights into the dual role of the key bidentate donor additive, and into the catalytic cycle. Iron catalysis has also been notoriously difficult to control, and the present study demonstrates that two capricious metals can be tamed in a productive way for organic synthesis.

## Results and discussion

As described in the introduction, the chemistry of organosodium is appealing for the development of sustainable synthesis, but the high reactivity of these compounds is difficult to control. A common way to control reactivity and selectivity in coupling reactions is the use of a transition-metal catalyst; however, organosodium compounds often uncontrollably reduce transition metals to unproductive mixtures of low-valent species. This is especially true for iron, which is notoriously difficult to control even for the reaction of organomagnesium or organolithium compounds^23–26^.

To develop practical coupling reactions with organosodium compounds, we decided to divide the reactions into two steps: the preparation of the organosodium compound and the catalytic C–C bond formation step, thereby suppressing the premature decomposition of organosodium (Fig. 1d). We have been exploring organosodium chemistry for some time^15,16,27–30^, and we expected that our recently developed efficient methods for generating organosodiums in hydrocarbon solvents under mild conditions, followed by judicious choice of oxidant and additive for the subsequent iron catalysis step, could suppress side reactions and achieve selective and efficient homo- and cross-coupling reactions.

### Iron-catalysed oxidative homocoupling of organosodium

**Fig. 2:**
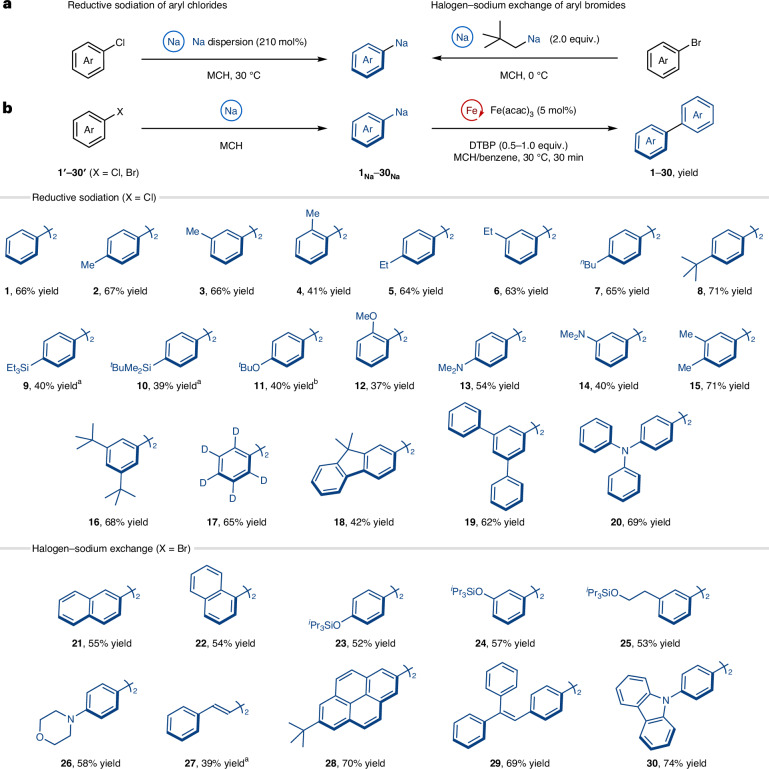
Iron-catalysed oxidative homocoupling of organosodium compounds generated in situ through reductive sodiation or halogen–sodium exchange. **a**, The preparation of organosodium compounds **1**_**Na**_–**30**_**Na**_ by reductive sodiation of aryl chlorides or halogen–sodium exchange with aryl- and alkenylbromides. **b**, Iron-catalysed oxidative homocoupling of aryl- and alkenylsodium compounds. Yields were determined by isolation of the homocoupled products. ^a^The homocoupling was conducted at 50 °C. ^b^Fe(acac)_3_ (10 mol%) was used. Ar, aryl.

We began our investigation with identifying the key parameters for the iron-catalysed oxidative homocoupling of organosodiums, the catalytic version of the Fittig-type coupling. We generated 4-(*tert*-butyl)phenylsodium (**8**_**Na**_) in situ by reductive sodiation of the corresponding aryl chloride (**8**′) with sodium dispersion^13,15^, and we used it as a model substrate for iron-catalysed oxidative homocoupling in one pot (Fig. 2 and Supplementary Table 1). Screening of iron sources and other parameters identified commercially available iron (III) acetylacetonate (acac) as the optimal catalyst, whereas iron fluoride, chloride, bromide, acetate and triflate resulted in low yields (<5%), which we speculate is partly due to the low solubility of these iron salts in hydrocarbon solvent. Methylcyclohexane (MCH) was a better solvent than hexane, and di-*tert*-butyl peroxide (DTBP)^31^ was a much more efficient oxidant than 1,2-dichloroethane^32^.

A variety of aryl and alkenyl organosodium compounds **1**_**Na**_–**30**_**Na**_ were prepared in situ through reductive sodiation of aryl chlorides^15,33^ or halogen–sodium exchange of aryl bromides^16,33,34^, and the scope of the iron-catalysed oxidative homocoupling was explored for the construction of biaryl derivatives **1**–**30** (Fig. 2a,b). A key factor in minimizing the formation of undesired side products is the efficiency of the sodiation protocol; the starting materials (**1**′–**30**′) were converted to the corresponding organosodium compounds (**1**_**Na**_–**30**_**Na**_) with high yields, avoiding unproductive reactions of unreacted aryl halides in the subsequent step. One-pot homocoupling of phenylsodium (**1**_**Na**_) prepared by reductive sodiation of chlorobenzene (**1**′) went smoothly. This reaction was also applicable to mono- and dialkylated arylsodium compounds, to give the corresponding biaryl products (**2**–**8**, **15** and **16**) in moderate-to-good yields. Other arylsodium compounds bearing silyl (**9**_**Na**_ and **10**_**Na**_), alkoxy (**11**_**Na**_ and **12**_**Na**_) and amino (**13**_**Na**_ and **14**_**Na**_) groups also reacted well. The synthesis of fully deuterated biphenyl (**17**) was achieved using readily available chlorobenzene-*d*_5_ (**17**′), and oligoarylene derivatives (**18** and **19**) could be conveniently synthesized through this Fittig-type coupling. For compounds **21**–**30**, the organosodiums were generated through halogen–sodium exchange of the corresponding aryl bromides with neopentylsodium and used for one-pot iron-catalysed oxidative homocoupling under the same conditions. Conjugated molecules were synthesized by the reaction of π-extended aryl- and alkenylsodium nucleophiles (**21**_**Na**_, **22**_**Na**_ and **27**_**Na**_–**29**_**Na**_). Despite the strong reducing and basic conditions, a silyl protecting group on phenol (**23** and **24**) and alcohol (**25**) and a carbazolyl group (**30**) were well tolerated. The iron-catalysed homocoupling of organosodium was successfully applied to the synthesis of functional organic molecules such as TPB ((**20**, *NN*,*N*,*N*′,*N*′-tetraphenylbenzidine), DPVBi (**29**, 4,4’-bis(2,2-diphenylvinyl)-1,1’-biphenyl), and CBP (**30**,4,4’-di(9*H*-carbazol-9-yl)-1,1’-biphenyl), demonstrating the potential of iron/sodium chemistry for synthetic applications in materials science.

### Investigation of additives for the development of cross-coupling

**Fig. 3:**
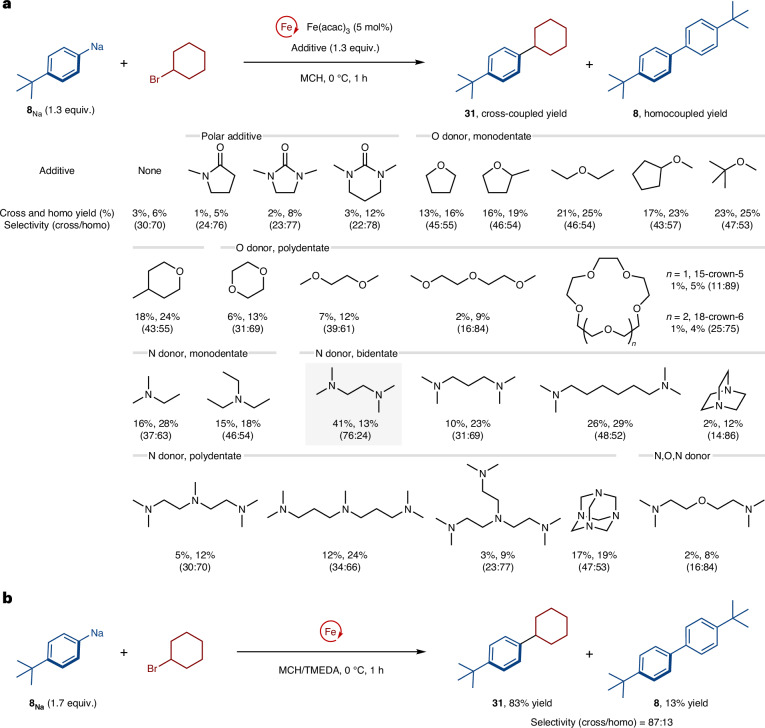
Influence of polar and Lewis donor additives on the iron-catalysed cross-coupling of arylsodium with bromocyclohexane. **a**, The effect of polar and Lewis donor additives. **b**, Selective cross-coupling reaction under the optimized reaction conditions with Fe(acac)_3_ (5 mol%) and TMEDA as a cosolvent (see the details in the Supplementary Information). Yields were determined by gas chromatography analysis using dodecane as an internal standard, after calibration.

Building upon the success in controlling the catalytic reactivity for the oxidative homocoupling using organosodium reagents and an iron catalyst, we subsequently investigated the C(*sp*^2^)–C(*sp*^3^) cross-coupling reaction of arylsodiums with alkyl halides (Wurtz–Fittig-type coupling). The initial attempt at iron-catalysed cross-coupling of 4-(*tert*-butyl)phenylsodium (**8**_**Na**_) with a secondary alkyl bromide such as bromocyclohexane gave only a trace amount of the desired cross-coupled product **31**, and the main compound obtained was the homocoupled product **8** (Fig. 3 and Supplementary Fig. 1). Thus, we decided to investigate the reaction parameters in order to switch the selectivity towards cross-coupling.

Highly ionic organosodium compounds typically aggregate to form complex polymeric structures in solution and solid state^35,36^. Therefore, to control the aggregation state of the organosodium compounds in MCH, we screened various additives capable of disrupting their aggregation. Thus, the iron-catalysed cross-coupling of arylsodium **8**_**Na**_ with bromocyclohexane was conducted in the presence of various polar and Lewis donor additives (Fig. 3a). Although the use of polar additives, such as *N*-methylpyrrolidone (NMP)^37,38^, *N*,*N*'-dimethylethyleneurea (DMI)^39^ and *N*,*N*′-dimethylpropyleneurea (DMPU)^40^ is known to enhance the iron-catalysed coupling of Grignard reagents, the amide and urea moieties are incompatible with the highly nucleophilic organosodium, and both cross- and homocoupling products were obtained in low yield. The use of ethereal compounds improved the yield of the cross-coupling product^41^ but also promoted homocoupling, where the haloalkane acted as an oxidant rather than as an electrophile. The results obtained with mono-, di- and higher dentate *N*-donor additives^42,43^ mirrored those observed when *O*-donor additives were used. Intriguingly, only *N*,*N*,*N*′,*N*′-tetramethylethylenediamine (TMEDA)^44,45^ inverted the selectivity of the reaction, resulting in cross-selective coupling (cross/homo = 76:24). With TMEDA as a cosolvent^46^, increasing the amount of organosodium nucleophile to 1.7 equivalent resulted in a much improved yield of cross-coupled product with high cross-selectivity (Fig. 3b). A control experiment confirmed that, in the absence of the iron catalyst, the cross-coupling product was not obtained at all (Supplementary Table 3). Notably, the use of other transition-metal catalysts such as palladium, nickel and copper did not promote the cross-coupling reaction and produced exclusively the homocoupled product (Supplementary Table 3). Thus, the metallic impurities contained in the iron salt are considered to exert a minimal influence on the cross-coupling reaction^47^, and the unique efficiency of iron as a catalyst illustrates the importance of appropriate pairing of the two metals to control their reactivity.

### Scope of the iron-catalysed cross-coupling of organosodium

**Fig. 4:**
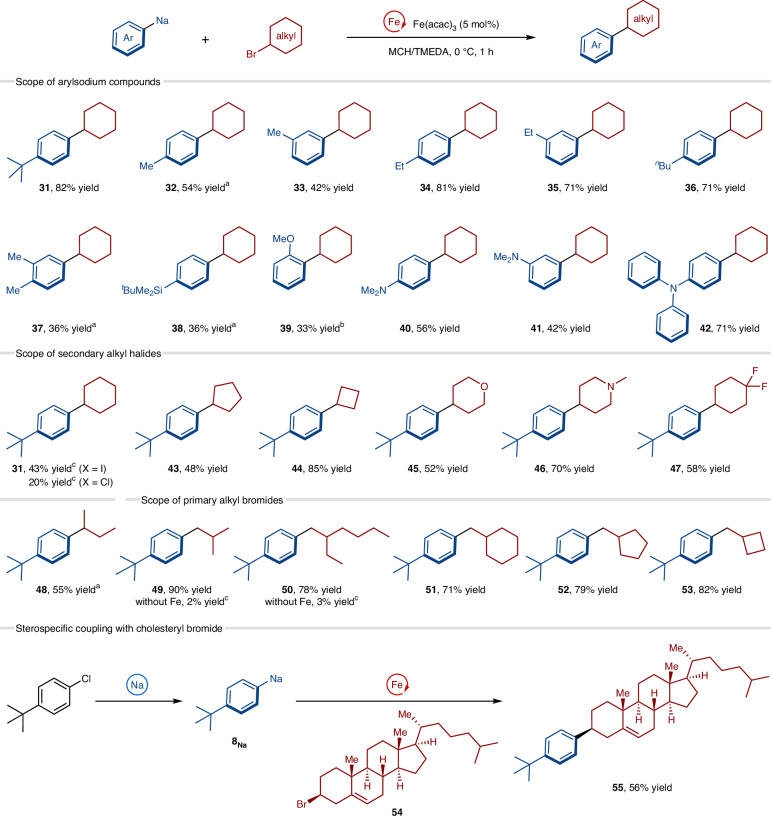
Scope of the iron-catalysed cross-coupling reaction of aryl organosodium compounds with alkyl halides. Yields were determined by isolation of the cross-coupled products. ^a^Fe(acac)_3_ (10 mol%) was used. ^b^Fe(acac)_3_ (20 mol%) was used and the cross-coupling was conducted at 30 °C. ^c^Yields were determined by gas chromatography analysis using dodecane as an internal standard. Ar, aryl.

With the optimized conditions, we examined the substrate scope for the iron-catalysed cross-coupling (Fig. 4). First, several arylsodium compounds were evaluated for the cross-coupling with bromocyclohexane, and the corresponding products were obtained in moderate-to-good yield. Methyl (**32**, **33** and **37**), ethyl (**34** and **35**), butyl (**31** and **36**), silyl (**38**), methoxy (**39**) and amino (**40**, **41** and **42**) groups on the *para*-, *meta*- and *ortho*-positions of the nucleophilic aromatic ring were tolerated. We next explored the scope of secondary and primary alkyl halides. The use of cyclohexyl iodide or chloride instead of bromide gave the cross-coupled product (**31**) in a lower yield. Cycloalkyl bromides with different ring sizes (**43** and **44**) and those containing oxygen (**45**), nitrogen (**46**) and fluorine (**47**) reacted well. An acyclic secondary alkyl bromide gave product **48** in good yield. Primary alkyl bromides substituted with a *β*-alkyl group gave the Wurtz–Fittig-type products (**49**–**53**) in good-to-excellent yields; the iron-free coupling reaction of arylsodium in TMEDA cosolvent did not proceed under these conditions (**49** and **50**). Finally, we applied the cross-coupling protocol for the selective functionalization of a bioactive molecule; the arylation of cholesteryl bromide (**54**) proceeded smoothly with the retention of the stereochemistry, forming **55** as a single stereoisomer^48,49^.

### Mechanistic investigations

**Fig. 5:**
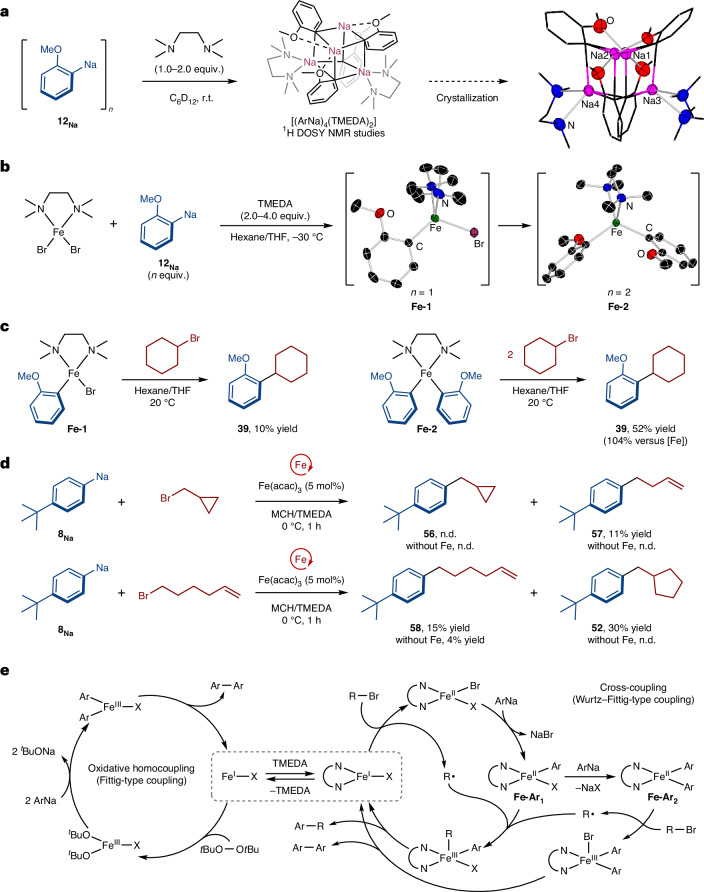
Mechanistic investigation and proposed catalytic cycle. **a**, TMEDA deaggregation of organosodium reagents. **b**, The direct synthesis and characterization of organoiron species. **c**, Stoichiometric reaction with organoiron complexes. **d**, Radical-clock experiments. **e**, Plausible mechanism of iron-catalysed direct coupling of organosodium compounds. Ar, aryl; r.t., room temperature; n.d., not detected.

To better understand the selective formation of the cross-coupled product with alkyl bromides, we first examined the effect of varying equivalents of TMEDA on the composition of the formed arylsodiums. To that end, we prepared salt- and donor-free **8**_**Na**_ and **12**_**Na**_ and studied their solution behaviour upon addition of the Lewis donor TMEDA. We observed that, upon addition of stoichiometric amounts of the donor, **8**_**Na**_ remained insoluble in cyclohexane, whereas **12**_**Na**_ was solubilized. ^1^H diffusion-ordered spectroscopy nuclear magnetic resonance (^1^H DOSY NMR) spectroscopic studies in deuterated cyclohexane of the latter revealed the formation of an aggregate with an estimated molecular weight of 816 g mol^−1^, which would fit with a tetrameric structure of the form [(ArNa)_4_(TMEDA)_2_]. Crystallization at −30 °C provided single crystals that confirmed the suspected tetrameric structure suggested by ^1^H DOSY NMR spectroscopic studies, [(C_6_H_4_OMeNa)_4_(TMEDA)_2_] (Fig. 5a). This compound exhibits a cubane structure, with Na and C at alternate corners. Interestingly, two different sodium environments are present; Na1/Na2 are stabilized by forming Na–O interactions with two neighbouring OMe groups, whereas Na3/Na4 achieve coordinative saturation by bonding to the two N atoms of a TMEDA molecule (Fig. 5a). Further addition of an excess of TMEDA into the highly insoluble **8**_**Na**_ (30 equiv.) and **12**_**Na**_ (2 equiv.) in deuterated cyclohexane delivered more soluble aggregates, with estimated molecular weights of 709 g mol^−1^ and 609 g mol^−1^. The calculated molecular weights of the aggregates present in an excess of TMEDA suggest that a dimeric motif (dimer, 545 g mol^−1^) is present in the solution for **8**_**Na**_ and that an aggregate with a motif between the tetrameric structure observed by crystallography ([(C_6_H_4_OMeNa)_4_(TMEDA)_2_], 753 g mol^−1^) and a dimer (dimer, 493 g mol^−1^) is present in the solution for **12**_**Na**._ The results of our ^1^H DOSY NMR spectroscopic studies demonstrate how the addition of TMEDA is able to not only solubilize these arylsodiums but also decrease their level of aggregation, increasing their kinetic reactivity. We can correlate these observations with the key role of TMEDA for an efficient cross-coupling, being able to form in this case less aggregated and more soluble arylsodium reagents. The role of other common Lewis donors in deaggregating **12**_**Na**_ was also probed by ^1^H DOSY NMR spectroscopic studies. A tetrameric structure was observed in solution upon the addition of 1 equiv. of PMDETA, and the addition of 4 equiv. of tetrahydrofuran (THF) to **12**_**Na**_ led to a trisolvated dimeric structure, whereas the addition of the crown ether 15-crown-5 led to a rapid decomposition of the donor as monitored by NMR spectroscopy. Although the deaggregation of **12**_**Na**_ was observed with other donors, it was also noticed that different yields of the cross-coupled product were obtained when using different donors, suggesting that the donor may play an additional role in the catalysis beyond that of solvating the arylsodium intermediates.

Thus, we expected that the addition of TMEDA would also have an impact on the iron catalytic cycle. For iron-catalysed cross-coupling reactions, a mechanistic understanding of the catalytic cycle and identification of the reactive species is critically important for successful reaction development, as iron is known to exhibit a rich redox chemistry with formal oxidation states typically from Fe(−II) to Fe(+III), and a variety of reactivity patterns^50–52^. To gain insights into the mechanism of iron-catalysed coupling of organosodiums, we first investigated the reactivity of arylsodium reagents with an iron complex. We started with (TMEDA)FeBr_2_, because we speculated that Fe(II) bromide species might form during the catalytic cycle of the cross-coupling with alkyl bromides in the presence of TMEDA^53^. Upon addition of 1 or 2 equiv. of **12**_**Na**_ in the presence of TMEDA, we obtained the corresponding [(TMEDA)FeBr(C_6_H_4_OMe)] **Fe-1** and [(TMEDA)Fe(C_6_H_4_OMe)_2_] **Fe-2** (Fig. 5b)_._ These complexes decompose rapidly in a few hours at room temperature, forming variable amounts of 2,2′-dimethoxybiphenyl (**12**) and other unidentified products. Crystallographic studies confirmed the identity of these species, showing their monomeric structure, analogous to the structures reported bearing a phenyl group by Neidig and coworkers, which were prepared by salt metathesis with PhMgBr in THF^53^. Similarly, the reaction of in situ formed **Fe-2** with cyclohexyl bromide delivered the corresponding cross-coupled product in 52% yield (104% yield versus **Fe-2**), whereas **Fe-1** delivered the cross-coupled product in just 10% yield (Fig. 5c). These observations are consistent with the radical mechanism proposed for the coupling of aryl Grignard reagents catalysed by iron in the presence of TMEDA^53^, based on extensive analysis of the reaction mixtures by Mössbauer spectroscopy and the isolation of iron intermediates. The feasibility of the proposed mechanism was also supported by DFT calculations^54^, showing that the Fe(II)/Fe(III)/Fe(I) is the most plausible mechanism. Because we access similar iron species in solution using organosodium nucleophiles instead of organomagnesium, we propose that the formed **Fe-2** is able to react with alkyl bromides in a similar way to generate an alkyl radical species and provide a higher yield of cross-coupled product **39**, whereas **Fe-1** is unable to generate the alkyl radical efficiently and results in a poor yield of the cross-coupled product. Control experiments reacting Fe(acac)_3_ with the tetrameric form of **12**_**Na**_, [(C_6_H_4_OMeNa)_4_(TMEDA)_2_], in the presence or absence of additional TMEDA in C_6_D_6_ show the formation of **Fe-2** in solution (Supplemantary Information section 8 and Supplementary Figs. 6 and 7), suggesting that the same TMEDA-bound iron species are formed in catalytic conditions where Fe(acac)_3_ is used instead of FeBr_2_.

To confirm the formation of radical intermediates in solution, we performed radical**-**clock experiments using electrophile probes such as (bromomethyl)cyclopropane and 6-bromohex-1-ene, in parallel with control experiments in the absence of any iron catalyst (Fig. 5d). The reaction with (bromomethyl)cyclopropane resulted in the exclusive formation of the ring-opened product (**57**), with no cyclic product (**56**) observed. When 6-bromohex-1-ene was used as a substrate, the ring-closed product (**52**) was obtained as the main product, together with some uncyclized product (**58**). These results indicated that an alkyl radical species is generated by homolytic cleavage of the C–Br bond facilitated by the active iron species.

On the basis of these experimental results and the consideration of literature reports^31,53,55,56^, we herein propose a plausible mechanism in iron-catalysed coupling reactions of organosodium compounds (Fig. 5e). The Fe(III) precursor is reduced by arylsodium to generate reduced Fe(I) or Fe(II) species, with the release of the biaryl compound, in the absence or presence of TMEDA. For the oxidative homocoupling of arylsodium reagents, the Fe(I) complex is reoxidized by DTBP and the subsequent transmetallation with arylsodium followed by reductive elimination affords the biaryl product to close the catalytic cycle. In the case of cross-coupling in the presence of excess TMEDA, an arylsodium complex solvated by TMEDA can transmetallate to the Fe(II) centre to form (TMEDA)FeArX (**Fe-Ar**_**1**_) or (TMEDA)FeAr_2_ (**Fe-Ar**_**2**_), the latter of which can react with alkyl bromide to generate an alkyl radical. The recombination of **Fe-Ar**_**1**_ with the alkyl radical yields the corresponding Fe(III) intermediate, which reductively eliminates the cross-coupled product. The resulting reduced (TMEDA)Fe(I)X complex can react more efficiently with the alkyl bromide to generate the Fe(II) species and release an alkyl radical that will recombine with **Fe-Ar**_**1**_ as in the first cycle and propagate the catalytic cycle. The occasional formation of **Fe-Ar**_**2**_ can explain the radical initiation pathway of the catalytic cycle as well as the formation of a small amount of homocoupled product, which is observed in experimental conditions. The low solubility of arylsodium reagents (even in the presence of excess TMEDA) would limit the amount of diaryliron species formed in solution, favouring the formation of cross-coupling product and avoiding the need for slow addition of the organometallic reagent, which is typically required when using other polar organometallics such as ArMgX or ArLi (refs. ^44,57–59^). Nevertheless, we cannot completely rule out the formation of sodium ferrates in solution (such as NaFeAr_3_). Previous studies have shown that ferrate complexes can react with alkyl bromides to give the corresponding cross-coupled products; Bedford showed that the [FeMes_3_]^−^ intermediate generated from a bulky mesitylmagnesium bromide is more reactive than (TMEDA)FeMes_2_(ref. ^60^), although more recent studies by Neidig assessing the reactivity of ArMgBr in these reactions have shown that ferrates with less sterically hindered substituents are thermally less stable and react less efficiently with an electrophile than the relevant (TMEDA)FeAr_2_ species^53,61^. Multiple attempts to isolate sodium tris(aryl)ferrate by reacting (TMEDA)FeBr_2_ with an excess of **12**_**Na**_ resulted in the isolation of iron bis(aryl) **Fe-2**. We next attempted the synthesis of a heteroleptic sodium ferrate via co-complexation of a bis(alkyl) complex, (TMEDA)Fe(CH_2_SiMe_3_)_2_, with **12**_**Na**_. The Fe(II) alkyl complex has been shown to be thermally robust^62^ and can form stable ferrates with sodium alkyl reagents^63^. Interestingly, the stoichiometric reaction of the in situ formed ferrate (TMEDA)_2_NaFe(CH_2_SiMe_3_)_2_(Ar) (**I**) with cyclohexyl bromide led to the formation of the cross-coupled product in a reasonable yield of 56%. However, attempts to isolate ferrate (**I**) gave (TMEDA)_2_NaFe(CH_2_SiMe_3_)_3_ as a crystalline solid^63^. This is consistent with ligand redistribution in **I**, and concomitant formation of (TMEDA)_2_NaFe(Ar)_3_, which can ultimately lead to the reactive (TMEDA)FeAr_2_ (**Fe-2**) complex, possibly explaining the formation of the cross-coupled product in this stoichiometric experiment.

## Conclusions

We have developed an iron-catalysed homo- and cross-coupling of organosodium compounds, which enables the formation of C(*sp*^2^)–C(*sp*^2^) and C(*sp*^2^)–C(*sp*^3^) bonds in a more sustainable manner. We applied this reaction to the synthesis of various biaryl and alkylarene molecules, including π-conjugated molecules and a cholesterol derivative. A plausible catalytic cycle involving the generation of organoiron(II) species, which reacts with an alkyl halide through single-electron transfer to initiate the radical process, was proposed and supported by mechanistic studies, paralleling the proposed mechanism for the iron-catalysed cross-coupling of organomagnesium compounds. Iron-catalysed couplings have focused primarily on the utilization of organomagnesium, organozinc and organoboron reagents and, recently, organolithiums^59,64^. When reactive compounds such as organomagnesium and organolithium are used, the slow addition of the organometallic reagent and/or cryogenic conditions are generally required for controlling the catalytic reactivity of iron; in this study, iron catalysis has met organosodium chemistry, and notably, the reactions proceeded under mild conditions and without slow addition of reagents.

The use of sustainable metals such as iron for catalysis has become an important area of modern research^65^; we believe that the present demonstration of the direct use of organosodium compounds in catalysis will lay the foundations for a concept of sustainable organometallic reagents^66^. There has been a renaissance of interest in the development of synthetic reactions using organosodium chemistry^67–71^, some of which use more sustainable approaches such as green solvents^33^, flow chemistry^34,72–74^ and mechanochemistry^75^. We, thus, believe that the coupling reactions described here will further stimulate the development of organosodium chemistry for sustainable synthesis.

## Methods

### General procedure for iron-catalysed oxidative homocoupling of organosodium compounds

In a dry Schlenk tube equipped with a glass-coated stirring bar, aryl chloride (0.50 mmol, if liquid) was added to a mixture of MCH (1.0 ml) and sodium dispersion (ca. 26 wt%, ca. 92.8 mg, 1.05 mmol, 210 mol%) under nitrogen. If the starting material was solid, the sodium dispersion was added to a mixture of aryl chloride (0.50 mmol) and MCH (1.0 ml). After stirring at 30 °C for 1 h to generate the corresponding arylsodium, Fe(acac)_3_ (8.8 mg, 25 μmol, 5 mol%), benzene (0.50 ml) and DTBP (46 μl, 0.25 mmol, 0.5 equiv.) were added to the suspension of the arylsodium in MCH at 0 °C, and the reaction mixture was stirred at 30 °C for 30 min. The reaction was quenched with D_2_O (0.1 ml) and then with a saturated aqueous solution of NH_4_Cl (1 ml) at 0 °C. After extraction with ethyl acetate (EtOAc), diethyl ether (Et_2_O) or dichloromethane (CH_2_Cl_2_) three times, the combined organic layers were passed through a plug of silica gel with EtOAc, Et_2_O or CH_2_Cl_2_ and concentrated under reduced pressure. The crude product was purified by column chromatography on silica gel to afford the desired compound.

### General procedure for iron-catalysed cross-coupling of organosodium compounds with alkyl halides

The organosodium compound was prepared using the same procedure as above. Fe(acac)_3_ (5.3 mg, 15 μmol, 5 mol%), TMEDA (0.50 ml) and alkyl halide (0.30 mmol, 1.0 equiv.) were added to the suspension of the arylsodium in MCH (1.0 ml) at 0 °C, and the reaction mixture was stirred at 0 °C for 1 h. The reaction was quenched with D_2_O (0.1 ml) and then with a saturated aqueous solution of NH_4_Cl (1 ml) at 0 °C. After extraction with EtOAc, Et_2_O or CH_2_Cl_2_ three times, the combined organic layers were passed through a plug of silica gel with EtOAc, Et_2_O or CH_2_Cl_2_ and concentrated under reduced pressure. The crude product was purified by column chromatography on silica gel to afford the desired compound.

## Supplementary information

Supplementary Information Experimental procedures, Characterization data, Mechanistic studies, Crystallographic details, Supplementary Figs. 1–136, Supplementary Tables 1–6, Supplementary Methods and Supplementary References.

Supplementary Data 1 Crystallographic data for **Fe-1**, CCDC 2375564.

Supplementary Data 2 Crystallographic data for **Fe-2**, CCDC 2375562.

Supplementary Data 3 Crystallographic data for [(C_6_H_4_OMeNa)_4_(TMEDA)_2_], CCDC 2375563.

## Data Availability

The data that support the findings of this study are available within the Article and its Supplementary Information. Detailed conditions for each reaction and compound characterization data are provided in the Supplementary Methods, Supplementary Fig. 1 and Supplementary Tables 1–5. NMR and electron paramagnetic resonance (EPR) spectra are available in Supplementary Figs. 2–14 and 18–136. Crystal structure data have been deposited at the Cambridge Crystallographic Data Centre (CCDC nos. 2375562 (**Fe-2**), 2375563 ([(C_6_H_4_OMeNa)_4_(TMEDA)_2_]) and 2375564 (**Fe-1**)), and crystallographic data are provided in the Supplementary Methods, Supplementary Figs. 15–17 and Supplementary Table 6. These data can be obtained free of charge via The Cambridge Crystallographic Data Centre at www.ccdc.cam.ac.uk/structures/.
