# Supplementary material for: Iron-catalysed direct coupling of organosodium compounds
Source: Nat Synth. 2025 Mar 25;4(7):816–25. doi: 10.1038/s44160-025-00771-1 (PMC12254037; doi:10.1038/s44160-025-00771-1)
Supplement: Supplementary file 1 — Experimental procedures, Characterization data, Mechanistic studies, Crystallographic details, Supplementary Figs. 1–136, Supplementary Tables 1–6, Supplementary Methods and Supplementary References. [file 44160_2025_771_MOESM1_ESM.pdf]

nature synthesis

Supplementary information https://doi.org/10.1038/s44160-025-00771-1

Iron-catalysed direct coupling of organosodium compounds

In the format provided by the authors and unedited

Table of contents

I. Supplementary Methods

1. General information S2
2. Investigation of key reaction parameters S4
3. Preparation of starting materials S9
4. Iron-catalysed oxidative homocoupling of arylsodium prepared by reductive sodiation S13
5. Iron-catalysed oxidative homocoupling of organosodium prepared by halogen–sodium exchange S21
6. Iron-catalysed cross coupling of arylsodium compounds with alkyl halides S27
7. Mechanistic investigation S39
8. Synthesis and characterisation of organometallic complexes S42
9. ^{1}H DOSY NMR studies S49
10. Stoichiometric reactions with **Fe-1** and **Fe-2** S56
11. Crystallographic details S58

II. Supplementary References S64

III. Supplementary Figures S67

1. General information

Instrumentation. All reactions dealing with air- or moisture-sensitive compounds were carried out in a dry Schlenk tube under an atmosphere of nitrogen or argon or in a glovebox under an inert atmosphere of argon. Glass-coated stirring bars were used for the reactions using sodium dispersion. Analytical thin-layer chromatography was performed on glass plates coated with 0.25 mm silica gel containing a fluorescent indicator (Merck #1.05715 TLC Silica gel 60 F_{254}). Flash silica gel column chromatography was performed on silica gel 60N (Kanto, spherical and neutral, 40–50 μm) as described by Still et al[1]. ^{1}H NMR, ^{2}H NMR, ^{13}C NMR, and ^{19}F NMR spectra were recorded on a JEOL ECA-500 spectrometers or on Bruker spectrometers operating at 300 MHz, 400 MHz, or 500 MHz, and reported in parts million referenced to the signals of Me_4Si (^{1}H: 0.00 ppm), $CDCl_3$ (^{13}C: 77.0 ppm), C_6F_6 (^{19}F NMR: –164.9 ppm) as an internal reference, respectively. The data is presented as follows: chemical shift, multiplicity (s = singlet, d = doublet, t = triplet, q = quartet, quin = quintet, sept = septet, m = multiplet and/or multiplet resonances, br = broad), coupling constant in Hertz (Hz), and integration. The melting point of solid materials were determined on a Yanaco MP-500 apparatus and were uncorrected. Gas chromatographic (GC) analyses were performed on Shimadzu GC-2014 equipped with an FID detector and capillary column (HR-1, 0.320-mm i.d. x 30 m). Mass spectra (GC-MS) were performed on SHIMADZU QP2010SE equipped with a capillary column (Rxi-5Sil MS, 0.25-mm i.d. x 30 m). Gel permeation chromatography (GPC) was performed on SHIMADZU LC-20A quipped with FP-2002 columns. High resolution mass spectra were obtained on Bruker microTOF-Q III (APCI). Elemental analyses (C, H, and N) were conducted with a Flash 2000 Organic Elemental Analyser (Thermo Scientific). Samples were prepared in the glovebox under argon atmosphere and sealed in an air-tight container prior to analyses. All results were obtained by the Analytical Research and Services Schürch Group of the University of Bern. Samples were weighed on a Mettler Toledo balance with ±2 μg resolution and sample weights from 1–3 mg were used. For calibration, a reference material such as cysteine was used. The presented values are the average of determinations in triplicate to ensure consistency.

Materials. Unless otherwise noted, reagents were purchased from Tokyo Chemical Industry Co., Ltd., Sigma-Aldrich Co., BLD Pharmatech Ltd., and other commercial suppliers and were used as received. Anhydrous hexane and tetrahydrofuran (THF) were purchased from Kanto Chemical Co., Inc. and purified prior to use by a solvent purification system (GlassContour) equipped with columns of activated alumina and supported copper catalyst (Q-5) prior to use[2]. Anhydrous methylcyclohexane (MCH) and benzene were purchased from Kanto Chemical Co., Inc. *N*,*N*,*N*',*N*'-Tetramethylethylenediamin (TMEDA) was purchased from Nacalai Tesque Inc., distilled from CaH_2, and stored over an activated molecular sieves 4Å under nitrogen in a Schlenk flask. $Fe(acac)_3$ (≥99.9% trace metal basis) was purchased from Sigma-Aldrich Co.. 1-Chloro-2,2-dimethhylpropane (neopentyl chloride) was purchased from Tokyo Chemical Industry Co., Ltd. and stored over activated molecular sieves 4Å. Di-*tert*-butyl peroxide (DTBP) was purchased from Nacalai Tesque Inc., and stored at 4 °C. Sodium dispersion (ca. 10 M suspension in mineral oil)

was provided by KOBELCO ECO-solutions Co., Ltd., and stored under argon at –20 °C. The concentration was determined by titration prior to use. The same sodium dispersion is also commercially available from Tokyo Chemical Industry Co., Ltd. [code: D5729]. Deuterated solvents (C_6D_6 and C_6D_{12}) were purchased from Sigma-Aldrich or Eurisotop, dried over NaK alloy for 16 hours, and then cycled through three rounds of degassing by employing a freeze-pump-thaw method. The deuterated solvents were then collected via vacuum transfer and stored under argon atmosphere over 4Å molecular sieves.

Caution. Sodium dispersion and organosodium compounds are insoluble in alkane solvents, and therefore efficient and vigorous stirring is very important.

2. Investigation of key reaction parameters

Iron-catalysed oxidative homocoupling of arylsodium $\mathbf{8_{Na}}$

Step 1: preparation of organosodium compound $\mathbf{8_{Na}}$.

In a dry Schlenk tube equipped with a glass-coated stirring bar, 1-(*tert*-butyl)-4-chlorobenzene (**8’**, 84.3 mg, 0.50 mmol, 1.0 equiv.) was added to a mixture of solvent (1.0 mL) and sodium dispersion (ca. 26 wt%, ca. 92.8 mg, 1.05 mmol, 210 mol%) under nitrogen. After stirring at 30 °C for 1 h, the corresponding arylsodium $\mathbf{8_{Na}}$ was generated with full conversion of the starting material. Quantitative formation was indicated by GC and GC-MS analysis of a separate reaction after quenching with D_2O.

Step 2: iron-catalysed oxidative homocoupling of organosodium compound $\mathbf{8_{Na}}$.

Iron source (25 µmol, 5 mol%), cosolvent (0.50 mL), and di-*tert*-butyl peroxide (DTBP, 46 µL, 0.25 mmol, 0.5 equiv.) were added to the suspension of arylsodium compound $\mathbf{8_{Na}}$ in solvent (1.0 mL) at 0 °C, and the reaction mixture was stirred at 30 °C for 30 min. The reaction was quenched with D_2O (0.1 mL) (to estimate the amount of active arylsodium remaining in the reaction mixture) and then with a saturated aqueous solution of NH_4Cl (1 mL) at 0 °C. The reaction mixture was diluted with ethyl acetate and passed through a plug of silica gel with ethyl acetate for GC and GC-MS analysis. The yields were estimated by GC analysis using tridecane as an internal standard.

Supplementary Table 1. Investigation of the key reaction parameters for homocoupling.

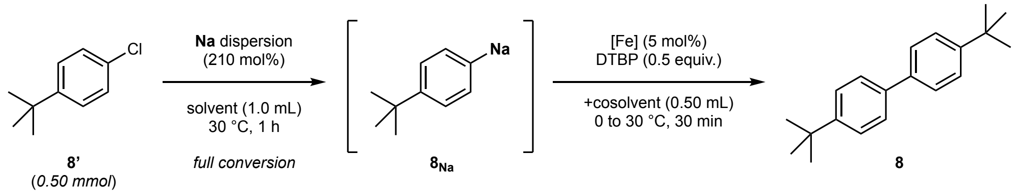

entry	solvent	cosolvent	[Fe]	**8** (%)
1	MCH	benzene	FeF_2	1
2	MCH	benzene	$FeCl_2$	1
3	MCH	benzene	$FeBr_2$	5
4	MCH	benzene	$Fe(OAc)_2$	1
5	MCH	benzene	$Fe(OTf)_2$	2
6	MCH	benzene	$Fe(acac)_2$	54
7	MCH	benzene	$Fe(acac)_3$	74 (71)

entry	solvent	cosolvent	[Fe]	**8** (%)
8	hexane	benzene	$Fe(acac)_3$	65
9	MCH	MCH	$Fe(acac)_3$	60
10	MCH	THF	$Fe(acac)_3$	37
11	MCH	Et_2O	$Fe(acac)_3$	59
12[a]	MCH	benzene	$Fe(acac)_3$	62
13[b]	MCH	benzene	$Fe(acac)_3$	6
14	MCH	benzene	none	1

a) DTBP (1.0 equiv.) was used.
b) 1,2-Dichloroethane (1.0 equiv.) was used.

Initial attempt at iron-catalysed cross coupling of arylsodium $\mathbf{8_{Na}}$ with bromocyclohexane in MCH and benzene

Step 1: preparation of organosodium compound $\mathbf{8_{Na}}$.
In a dry Schlenk tube equipped with a glass-coated stirring bar, 1-(*tert*-butyl)-4-chlorobenzene (**8'**, 67.5 mg, 0.40 mmol, 1.33 equiv.) was added to a mixture of MCH (1.0 mL) and sodium dispersion (ca. 26 wt%, ca. 72.5 mg, 0.84 mmol, 280 mol% to bromocyclohexane; 210 mol% to 1-*tert*-butyl-4-chlorobenzene) under nitrogen. After stirring at 30 °C for 1 h, the corresponding arylsodium $\mathbf{8_{Na}}$ was generated with full conversion of the starting material. Quantitative formation was indicated by GC and GC-MS analysis of a separate reaction after quenching with D_2O.

Step 2: iron-catalysed cross coupling of organosodium compound $\mathbf{8_{Na}}$ with bromocyclohexane.
$Fe(acac)_3$ (5.3 mg, 15 µmol, 5 mol%), benzene (0.50 mL), and bromocyclohexane (37 µL, 0.30 mmol, 1.0 equiv.) were added to the suspension of the arylsodium compound $\mathbf{8_{Na}}$ in MCH (1.0 mL) at 0 °C, and the reaction mixture was stirred at the same temperature for 1 h. The reaction was quenched with D_2O (0.1 mL) (to estimate the amount of active arylsodium remaining in the reaction mixture) and then with a saturated aqueous solution of NH_4Cl (1 mL) at 0 °C. The reaction mixture was diluted with ethyl acetate and passed through a plug of silica gel with ethyl acetate for GC and GC-MS analysis. The yields were estimated by GC analysis using dodecane as an internal standard, after calibration of the response curves. The yield of cross- (**31**) and homo-coupled (**8**) products was based on bromocyclohexane and 1-*tert*-butyl-4-chlorobenzene, respectively.

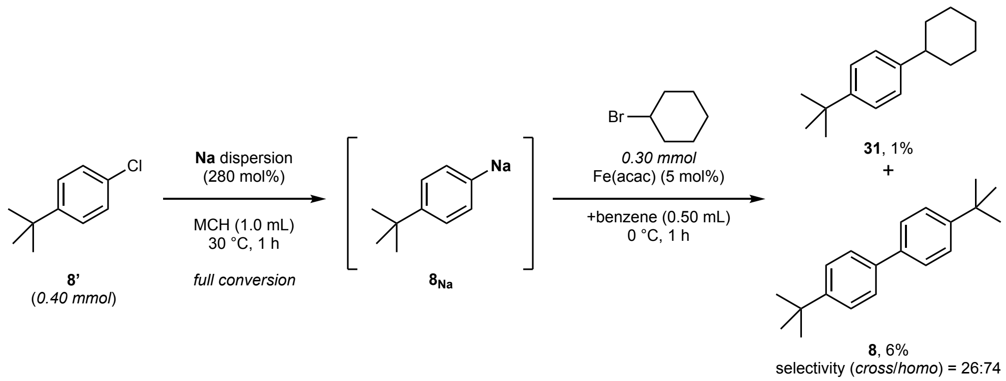

Supplementary Figure 1. Initial trial of iron-catalysed cross coupling in MCH–benzene.

Iron-catalysed cross coupling of arylsodium 8_{Na} with bromocyclohexane with additives

Step 1: preparation of organosodium compound $\mathbf{8_{Na}}$.
In a dry Schlenk tube equipped with a glass-coated stirring bar, 1-(*tert*-butyl)-4-chlorobenzene (**8'**, 67.5 mg, 0.40 mmol, 1.33 equiv.) was added to a mixture of MCH (1.0 mL) and sodium dispersion (ca. 26 wt%, ca. 72.5 mg, 0.84 mmol, 280 mol% to bromocyclohexane; 210 mol% to 1-*tert*-butyl-4-chlorobenzene) under nitrogen. After stirring at 30 °C for 1 h, the corresponding arylsodium $\mathbf{8_{Na}}$ was generated with full conversion of the starting material. Quantitative formation was indicated by GC and GC-MS analysis of a separate reaction after quenching with D_2O.

Step 2: iron-catalysed cross coupling of organosodium compound $\mathbf{8_{Na}}$ with bromocyclohexane.
$Fe(acac)_3$ (5.3 mg, 15 μmol, 5 mol%), additive (0.40 mmol, 1.33 equiv.), and bromocyclohexane (37 μL, 0.30 mmol, 1.0 equiv.) were added to the suspension of arylsodium compound $\mathbf{8_{Na}}$ in MCH (1.0 mL) at 0 °C, and the reaction mixture was stirred at the same temperature for 1 h. The reaction was quenched with D_2O (0.1 mL) (to estimate the amount of active arylsodium remaining in the reaction mixture) and then with a saturated aqueous solution of NH_4Cl (1 mL) at 0 °C. The reaction mixture was diluted with ethyl acetate and passed through a plug of silica gel with ethyl acetate for GC and GC-MS analysis. The yields were estimated by GC analysis using dodecane as an internal standard, after calibration of the response curves. The yield of cross- and homo-coupled products was based on bromocyclohexane and 1-(*tert*-butyl)-4-chlorobenzene (**8'**), respectively.

- Abbreviations –
NMP = *N*-methyl-2-pyrrolidone, DMI = 1,3-dimethyl-2-imidazolidinone, DMPU = 1,3-dimethyl-3,4,5,6-tetrahydro-2(1*H*)-pyrimidinone, THF = tetrahydrofuran, 2-MeTHF = 2-methyltetrahydrofuran, CPME = cyclopentylmethyl ether, MTBE = methyl-*tert*-butyl ether, MTHP = 4-methyltetrahydropyran, DME = 1,2-dimethoxyethane, diglyme = diethylene glycol dimethyl ether, TMEDA = *N*,*N*,*N*',*N*'-Tetramethylethylenediamin, TMPDA = *N*,*N*,*N*',*N*'-tetramethyl-1,3-propanediamine, TMHDA = *N*,*N*,*N*',*N*'-tetramethyl-1,6-hexanediamine, DABCO = 1,4-diazabicyclo[2.2.2]octane, PMDETA = *N*,*N*,*N*',*N*',*N*''-pentamethyldiethylenetriamine, PMDPTA = *N*,*N*,*N*',*N*',*N*''-pentamethyldipropene triamine, M_6TREN = Tris[2-(dimethylamino)ethyl]amine, HMTA = hexamethylenetetramine, TMDAE = *N*,*N*,*N*',*N*'-tetramethyldiaminoethyl ether.

Supplementary Table 2. Investigation of additives for iron-catalysed cross coupling.

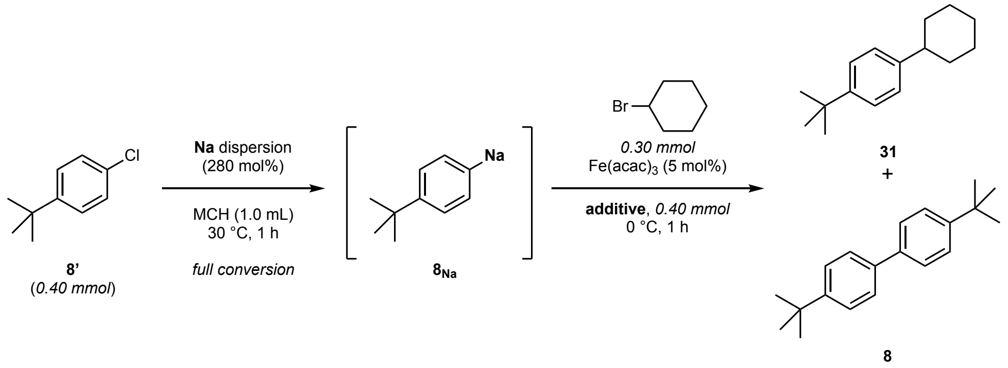

entry	additive	**31** (%)	**8** (%)	Selectivity (*cross*/*homo*)
1	none	3	6	30:70
2	NMP	1	5	24:76
3	DMI	2	8	23:77
4	DMPU	3	12	22:78
5	THF	13	16	45:55
6	2-MeTHF	16	19	46:54
7	Et_2O	21	25	46:54
8	CPME	17	23	43:57
9	MTBE	23	25	47:53
10	MTHP	18	24	43:55
11	1,4-dioxane	6	23	31:69
12	DME	7	12	39:61
13	diglyme	2	9	16:84
14	15-crown-5	1	5	11:89
15	18-crown-6	1	4	25:75
16	Me_2NEt	16	28	37:63
17	Et_3N	15	18	46:54
18	TMEDA	41	13	76:24
19	TMPDA	10	23	31:69
20	TMHDA	26	29	48:52
21	DABCO	2	12	14:86
22	PMDETA	5	12	30:70
23	PMDPTA	12	24	34:66
24	Me_6TREN	3	9	23:77
25	HMTA	17	19	47:53
26	TMDAE	2	8	16:84

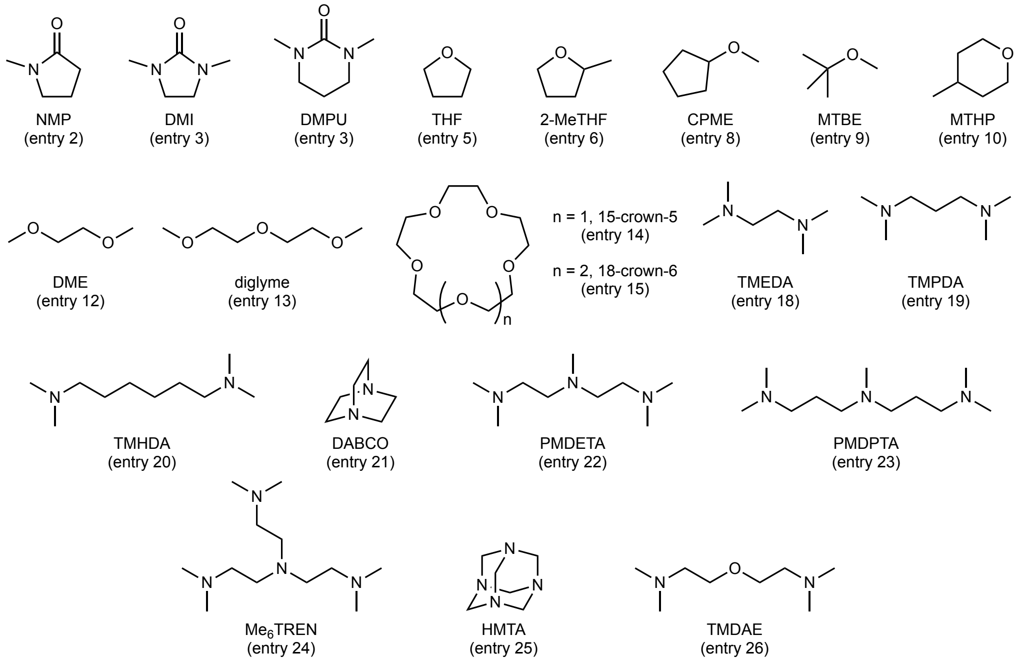

Iron-catalysed cross coupling of arylsodium 8_{Na} with bromocyclohexane using a cosolvent amount of TMEDA and control experiments with other transition metal catalysts

Step 1: preparation of organosodium compound $\mathbf{8_{Na}}$.

In a dry Schlenk tube equipped with a glass-coated stirring bar, 1-(*tert*-butyl)-4-chlorobenzene (**8'**, 84.3 mg, 0.50 mmol, 1.67 equiv.) was added to a mixture of MCH (1.0 mL) and sodium dispersion (ca. 26 wt%, ca. 92.8 mg, 1.05 mmol, 350 mol% to bromocyclohexane; 210 mol% to 1-(*tert*-butyl)-4-chlorobenzene) under nitrogen. After stirring at 30 °C for 1 h, the corresponding arylsodium $\mathbf{8_{Na}}$ was generated with full conversion of the starting material. Quantitative formation was indicated by GC and GC-MS analysis of a separate reaction after quenching with D_2O.

Step 2: transition-metal-catalysed cross coupling of organosodium compound $\mathbf{8_{Na}}$ with bromocyclohexane.

Transition metal catalyst (15 µmol, 5 mol%), TMEDA (0.50 mL), and bromocyclohexane (37 µL, 0.30 mmol, 1.0 equiv.) were added to the suspension of arylsodium compound $\mathbf{8_{Na}}$ in MCH (1.0 mL) at 0 °C, and the reaction mixture was stirred at the same temperature for 1 h. The reaction was quenched with D_2O (0.1 mL) (to estimate the amount of active arylsodium remaining in the reaction mixture) and then with a saturated aqueous solution of NH_4Cl (1 mL) at 0 °C. The reaction mixture was diluted with ethyl acetate and passed through a plug of silica gel with ethyl acetate for GC and GC-MS analysis. The yields were estimated by GC analysis using dodecane as an internal standard, after calibration. The yield of cross- and homo-coupled products was based on bromocyclohexane and 1-(*tert*-butyl)-4-chlorobenzene, respectively.

Supplementary Table 3. Investigation of other key reaction parameters and control experiments.

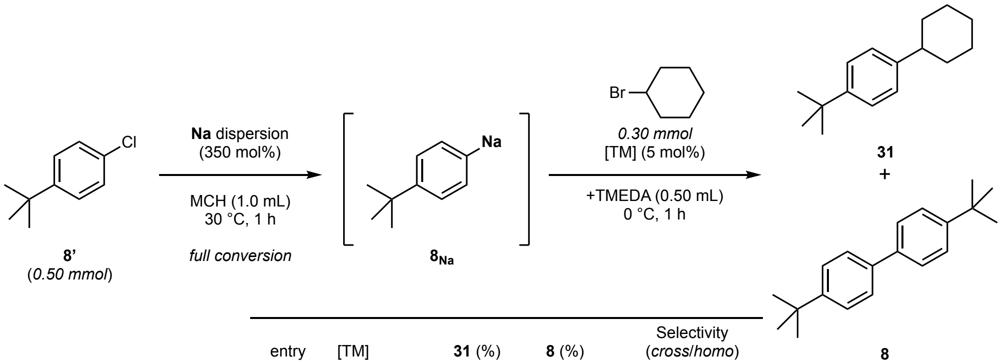

entry	[TM]	**31** (%)	**8** (%)	Selectivity (*cross*/*homo*)
1	$Fe(acac)_3$	83 (82)	13	87:13
2	none	n.d.	3	*homo* only
3	$Pd(acac)_2$	n.d.	10	*homo* only
4	$Ni(acac)_2$	n.d.	30	*homo* only
5	$Cu(acac)_2$	n.d.	4	*homo* only

3. Preparation of starting materials

Substrates **9'**, **20'**, and **27'** were prepared according to literature procedures[3,4,5].
The preparation of substrates **9'**, **10'**, **16'**, **18'**, **20'**, **25'**, and **27'** is described below.
Other substrates were purchased from commercial sources and were used as received.

(4-Chlorophenyl)triethylsilane (9')

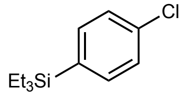

A hexane solution of *n*BuLi (1.59 M, 7.92 mL, 12.6 mmol, 1.05 equiv.) was added dropwise to a solution of 1-bromo-4-chlorobenzene (2.30 g, 12.0 mmol, 1.0 equiv.) in THF (35 mL, 0.34 M) at –78 °C. After stirring for 1 h, chlorotriethylsilane (2.41 mL, 14.4 mmol, 1.2 equiv.) was added dropwise to the reaction mixture at –78 °C. The reaction mixture was allowed to warm to room temperature and quenched with a saturated aqueous solution of NH_4Cl at 0 °C. The reaction mixture was extracted with hexane for three times, then the organic layer was washed with water and brine, and dried over Na_2SO_4. After concentration under reduced pressure, the crude product was purified by silica gel column chromatography (hexane only) and heated at 68–70 °C under high vacuum to remove butylated side product to afford the title compound **9'** as a colorless liquid (2.24 g, 9.88 mmol, 82%). The spectral data was in good agreement with the literature[3].

^{1}H NMR (500 MHz, $CDCl_3$): δ 7.40 (d, *J* = 8.3 Hz, 2H), 7.32 (d, *J* = 8.3 Hz, 2H), 0.95 (t, *J* = 8.0 Hz, 9H), 0.77 (q, *J* = 8.0 Hz, 6H).

^{13}C NMR (126 MHz, $CDCl_3$): δ 135.7, 135.5, 135.0, 127.9, 7.3, 3.3.

***tert*-Butyl(4-chlorophenyl)dimethylsilane (10')**

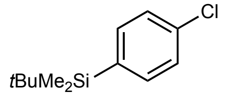

A hexane solution of *n*BuLi (1.59 M, 7.92 mL, 12.6 mmol, 1.05 equiv.) was added dropwise to a solution of 1-bromo-4-chlorobenzene (2.32 g, 12.1 mmol, 1.0 equiv.) in THF (35 mL, 0.34 M) at –78 °C. After stirring for 1 h, a solution of *tert*-butyldimethylchlorosilane (2.19 g, 14.5 mmol, 1.2 equiv.) in THF (8 mL) was added dropwise to the reaction mixture at –78 °C. The reaction mixture was allowed to warm to room temperature and quenched with a saturated aqueous solution of NH_4Cl at 0 °C. The reaction mixture was extracted with hexane for three times, then the organic layer was washed with water and brine, and dried over Na_2SO_4. After concentration under reduced pressure, the crude product was purified by silica gel column chromatography (hexane only) and heated at 68–70 °C under high vacuum to remove butylated side product to afford the title compound **10'** as a colorless liquid (1.63 g, 7.20 mmol, 60%).

^{1}H NMR (500 MHz, $CDCl_3$): δ 7.42 (d, *J* = 8.3 Hz, 2H), 7.32 (d, *J* = 8.3 Hz, 2H), 0.86 (s, 9H), 0.26 (s, 6H).

^{13}C NMR (126 MHz, $CDCl_3$): δ 136.1, 135.7, 135.1, 127.7, 26.4, 16.8, –6.2.

GC-MS (EI): *m*/*z* (relative intensity): 226 (M^+, 2), 171 (35), 170 (13), 169 (100), 91 (15).

1,3-Di-*tert*-butyl-5-chlorobenzene (16’)

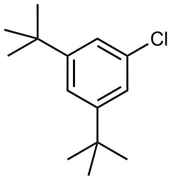

A hexane solution of *n*BuLi (1.57 M, 8.10 mL, 12.7 mmol, 1.05 equiv.) was added dropwise to a solution of 1-bromo-3,5-di-*tert*-butylbenzene (3.23 g, 12.0 mmol, 1.0 equiv.) in THF (35 mL, 0.34 M) at –78 °C and the reaction mixture was stirred for 2 h. A solution of hexachloroethane (4.26 g, 18.0 mmol, 1.5 equiv.) in THF (9.0 mL) was added dropwise to the reaction mixture at –78 °C and the reaction mixture was stirred for 1 hour at the same temperature. The reaction mixture was allowed to warm to room temperature, then it was quenched with saturated aqueous solution of NH_4Cl. The reaction mixture was extracted with hexane for three times, then the organic layer was washed with brine, and dried over Na_2SO_4. After concentration under reduced pressure, the crude product was purified by silica gel column chromatography (hexane only) and heated at 50 °C under high vacuum to remove hexachloroethane to afford the title compound **16’** as a white solid (2.45 g, 10.9 mmol, 91%).
Melting point: 50–51 °C.
^{1}H NMR (500 MHz, $CDCl_3$): δ 7.28 (dd, *J* = 1.7, 1.7 Hz, 1H), 7.17 (d, *J* = 1.7 Hz, 2H), 1.31 (s, 18H).
^{13}C NMR (126 MHz, $CDCl_3$): δ 152.7, 133.5, 122.8, 120.6, 35.0, 31.3.
HRMS (APCI+): *m/z* Calcd. for $C_{14}H_{21}Cl$ $[M]^+$: 224.1326; found: 224.1326.

2-Chloro-9,9-dimethyl-9*H*-fluorene (18’)

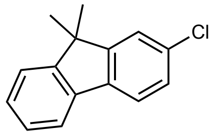

A hexane solution of *n*BuLi (1.59 M, 7.95 mL, 12.6 mmol, 1.05 equiv.) was added dropwise to a solution of 2-bromo-9,9-dimethyl-9*H*-fluorene (3.28 g, 12.0 mmol, 1.0 equiv.) in THF (35 mL, 0.34 M) at –78 °C and the reaction mixture was stirred for 2 h. A solution of hexachloroethane (3.98 g, 16.8 mmol, 1.4 equiv.) in THF (9.0 mL) was added dropwise to the reaction mixture at –78 °C and the reaction mixture was stirred for 1 hour at the same temperature. The reaction mixture was allowed to warm to room temperature, then it was quenched with a saturated aqueous solution of NH_4Cl. The reaction mixture was extracted with hexane for three times, then the organic layer was washed with brine and dried over Na_2SO_4. After concentration under reduced pressure, the crude product was purified by silica gel column chromatography (hexane only) to afford the title compound **18’** as a white solid (2.61 g, 11.4 mmol, 95%).
Melting point: 49–50 °C.
^{1}H NMR (500 MHz, $CDCl_3$): δ 7.69–7.67 (m, 1H), 7.63 (d, *J* = 8.0 Hz, 1H), 7.43–7.41 (m, 1H), 7.40 (d, *J* = 1.8 Hz, 1H), 7.36–7.30 (m, 3H), 1.48 (s, 6H).

^{13}C NMR (126 MHz, $CDCl_3$): δ 155.3, 153.3, 138.1, 137.7, 132.8, 127.5, 127.2, 127.1, 123.2, 122.6, 121.0, 120.0, 47.0, 27.0.
HRMS (APCI+): *m/z* Calcd. for $C_{15}H_{13}Cl$ $[M+H]^+$: 229.0779; found: 229.0776.

4-Chloro-*N*,*N*-diphenylaniline (20')

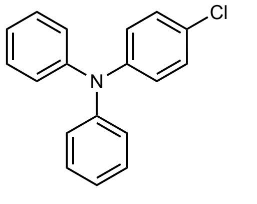

A hexane solution of *n*BuLi (1.59 M, 7.95 mL, 12.6 mmol, 1.05 equiv.) was added dropwise to a solution of 4-bromo-*N*,*N*-diphenylaniline (3.89 g, 12.0 mmol, 1.0 equiv.) in THF (35 mL, 0.34 M) at –78 °C and the reaction mixture was stirred for 1.5 h. A solution of hexachloroethane (3.98 g, 16.8 mmol, 1.4 equiv.) in THF (9.0 mL) was added dropwise to the reaction mixture at –78 °C and the reaction mixture was stirred for 1 hour at the same temperature. The reaction mixture was allowed to warm to room temperature, then it was quenched with a saturated aqueous solution of NH_4Cl. The reaction mixture was extracted with hexane for three times, then the organic layer was washed with brine and dried over Na_2SO_4. After concentration under reduced pressure, the crude product was purified by silica gel column chromatography (hexane only) to afford the title compound **20'** as a white solid (3.34 g, 11.9 mmol, quant.). The spectral data was in good agreement with the literature[4].
^1H NMR (500 MHz, $CDCl_3$): δ 7.23 (dd, J = 7.7, 7.7 Hz, 4H), 7.16 (d, J = 9.0 Hz, 2H), 7.06 (d, J = 7.7 Hz, 4H), 7.02–6.98 (m, 4H).
^{13}C NMR (126 MHz, $CDCl_3$): δ 147.4, 146.5, 129.3, 129.2, 127.3, 124.9, 124.3, 123.1.

(3-Bromophenethoxy)triisopropylsilane (25')

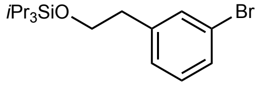

Triisopropylsilyl chloride (2.54 mL, 12.0 mmol, 1.2 equiv.) was added dropwise to a solution of 2-(3-bromophenyl)ethan-1-ol (2.01 g, 10.0 mmol, 1.0 equiv.) and imidazole (1.78 g, 26.1 mmol, 2.5 equiv.) in CH_2Cl_2 (33 mL, 0.30 M) at 0 °C. After stirring at room temperature overnight, the reaction mixture was quenched with water at 0 °C. The organic layer was washed with water, 1 M NaOH aq., and brine, and then dried over Na_2SO_4. After concentration under reduced pressure, the crude product was purified by silica gel column chromatography (hexane only) to afford the title compound **25'** as a colorless liquid (3.37 g, 9.41 mmol, 94%).
^1H NMR (500 MHz, $CDCl_3$): δ 7.39 (brs, 1H), 7.35–7.31 (m, 1H), 7.15–7.12 (m, 2H), 3.86 (t, J = 6.9 Hz, 2H), 2.81 (t, J = 6.9 Hz, 2H), 1.09–1.00 (m, 21H).
^{13}C NMR (126 MHz, $CDCl_3$): δ 141.8, 132.3, 129.7, 129.1, 127.8, 122.2, 64.3, 39.3, 17.9, 11.9.
HRMS (APCI+): *m/z* Calcd. for $C_{17}H_{29}Br^{79}OSi$ $[M+H]^+$: 357.1244; found: 357.1244.

(*E*)-(2-Bromovinyl)benzene (27’)

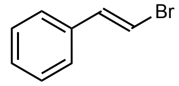

Dibromomethane (1.67 mL, 24.0 mmol, 4.0 equiv.) was added dropwise to a THF solution of NaHMDS (1.10 M, 16.4 mL, 18.0 mmol, 3.0 equiv.) in Et_2O (11 mL) at –78 °C in the dark. After 20 min, a solution of benzyl bromide (1.03 g, 6.00 mmol, 1.0 equiv.) in THF (5 mL) was added dropwise. The reaction mixture was stirred at –78 °C for 3 h and allowed to slowly warm to rt overnight. The reaction mixture was diluted with Et_2O, then the mixture was filtered through a pad of silica and the solvent was removed under reduced pressure. The crude product was purified by silica gel column chromatography (hexane only) to afford the title compound **27’** as a colorless liquid (0.71 g, 3.89 mmol, 65%, *E*/*Z* = >99:1). The spectral data was in good agreement with the literature[5].

^{1}H NMR (500 MHz, $CDCl_3$): δ 7,34–7.27 (m, 5H), 7.11 (d, J = 14.0 Hz, 1H), 6.77 (d, J = 14.0 Hz, 1H).

^{13}C NMR (126 MHz, $CDCl_3$): δ 137.2, 135.9, 128.8, 128.3, 126.1, 106.5.

4. Iron-catalysed oxidative homocoupling of arylsodium prepared by reductive sodiation

General procedure for iron-catalysed oxidative homocoupling of arylsodium

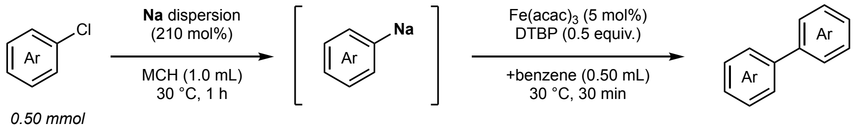

Step 1: preparation of organosodium compounds through reductive sodiation

In a dry Schlenk tube equipped with a glass-coated stirring bar, the aryl chloride (0.50 mmol, if liquid) was added to a mixture of MCH (1.0 mL) and sodium dispersion (ca. 26 wt%, ca. 92.8 mg, 1.05 mmol, 210 mol%) under nitrogen. When the starting material is a solid, the sodium dispersion was added to a mixture of the aryl chloride (0.50 mmol) and MCH (1.0 mL). The reaction mixture was stirred at 30 °C for 1 h.

Step 2: iron-catalysed oxidative homocoupling of organosodium compounds

$Fe(acac)_3$ (8.8 mg, 25 μmol, 5 mol%), benzene (0.50 mL), and di-*tert*-butyl peroxide (DTBP, 46 μL, 0.25 mmol, 0.5 equiv.) were added to the suspension of the arylsodium compound in MCH (1.0 mL) at 0 °C, and the reaction mixture was stirred at 30 °C for 30 min. The reaction was quenched with D_2O (0.1 mL) (to estimate the amount of active arylsodium remaining in the reaction mixture) and then with a saturated aqueous solution of NH_4Cl (1 mL) at 0 °C. After extraction with EtOAc, Et_2O, or CH_2Cl_2 for three times, the combined organic layers were passed through a plug of silica gel with EtOAc, Et_2O, or CH_2Cl_2 and concentrated under reduced pressure. The crude product was purified by column chromatography on silica gel using the indicated eluent to afford the desired compound.

1,1'-Biphenyl (1)

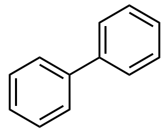

The general procedure was applied to chlorobenzene (**1'**, 56.3 mg, 0.50 mmol), sodium dispersion (25.9 wt%, 93.6 mg, 1.05 mmol), and $Fe(acac)_3$ (8.8 mg, 25 μmol). The crude product was purified by column chromatography on silica gel (pentane only) to afford the title compound as a white solid (25.6 mg, 0.17 mmol, 66%). The spectral data of product **1** was in good agreement with the literature[6].

^{1}H NMR (500 MHz, $CDCl_3$): δ 7.59 (d, J = 8.0 Hz, 4H), 7.43 (dd, J = 7.7, 7.7 Hz, 4H), 7.33 (dd, J = 7.5, 7.5 Hz, 2H).

^{13}C NMR (126 MHz, $CDCl_3$): δ 141.2, 128.7, 127.22, 127.15.

4,4'-Dimethyl-1,1'-biphenyl (2)

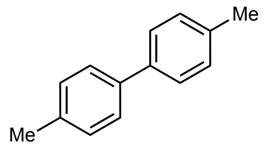

The general procedure was applied to 1-chloro-4-methylbenzene (**2'**, 63.5 mg, 0.50 mmol), sodium dispersion (26.1 wt%, 94.5 mg, 1.07 mmol), and $Fe(acac)_3$ (9.2 mg, 26 μmol). The crude product was purified by column chromatography on silica gel (pentane only) to afford the title compound as a white solid (30.8 mg, 0.17 mmol, 67%). The spectral data of product **2** was in good agreement with the literature[6].

^{1}H NMR (500 MHz, $CDCl_3$): δ 7.47 (d, J = 8.6 Hz, 4H), 7.22 (d, J = 8.0 Hz), 2.38 (s, 6H).

^{13}C NMR (126 MHz, $CDCl_3$): δ 138.3, 136.7, 129.4, 126.8, 21.1.

3,3'-Dimethyl-1,1'-biphenyl (3)

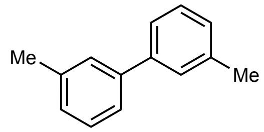

The general procedure was applied to 1-chloro-3-methylbenzene (**3'**, 63.3 mg, 0.50 mmol), sodium dispersion (25.9 wt%, 94.0 mg, 1.06 mmol), and $Fe(acac)_3$ (8.9 mg, 25 μmol). The crude product was purified by column chromatography on silica gel (pentane only) to afford the title compound as a colorless oil (29.9 mg, 0.16 mmol, 66%). The spectral data of product **3** was in good agreement with the literature[6].

^{1}H NMR (500 MHz, $CDCl_3$): δ 7.39–7.37 (m, 4H), 7.31 (dd, J = 7.5, 7.5 Hz, 2H), 7.14 (d, J = 7.5 Hz, 2H), 2.40 (s, 6H).

^{13}C NMR (126 MHz, $CDCl_3$): δ 141.3, 138.2, 128.6, 128.0, 127.9, 124.3, 21.5.

2,2'-Dimethyl-1,1'-biphenyl (4)

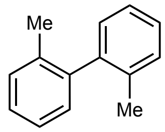

The general procedure (step 1, the reaction time was 4 h) was applied to 1-chloro-2-methylbenzene (**4'**, 63.7 mg, 0.50 mmol), sodium dispersion (25.9 wt%, 94.2 mg, 1.06 mmol), and $Fe(acac)_3$ (9.4 mg, 27 μmol). The crude product was purified by column chromatography on silica gel (pentane only) to afford the title compound as colorless oil (19.0 mg, 0.10 mmol, 41%). The spectral data of product **4** was in good agreement with the literature[7].

^{1}H NMR (500 MHz, $CDCl_3$): δ 7.27–7.20 (m, 6H), 7.10 (d, J = 6.9 Hz, 2H), 2.05 (s, 6H).

^{13}C NMR (126 MHz, $CDCl_3$): δ 141.6, 135.8, 129.8, 129.3, 127.1, 125.5, 19.8.

4,4'-Diethyl-1,1'-biphenyl (5)

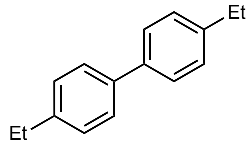

The general procedure was applied to 1-chloro-4-ethylbenzene (**5'**, 70.6 mg, 0.50 mmol), sodium dispersion (26.1 wt%, 94.7 mg, 1.08 mmol), and $Fe(acac)_3$ (9.3 mg, 26 μmol). The crude product was purified by column chromatography on silica gel (hexane only) to afford the title compound as a white solid (33.9 mg, 0.16 mmol, 64%). The spectral data of product **5** was in good agreement with the literature[8].

^1H NMR (500 MHz, $CDCl_3$): δ 7.50 (d, J = 8.6 Hz, 4H), 7.25 (d, J = 8.0 Hz, 4H), 2.68 (q, J = 7.6 Hz, 4H), 1.27 (t, J = 7.5 Hz, 6H).

^{13}C NMR (126 MHz, $CDCl_3$): δ 143.0, 138.6, 128.2, 126.9, 28.5, 15.6.

3,3'-Diethyl-1,1'-biphenyl (6)

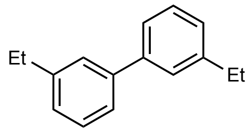

The general procedure was applied to 1-chloro-3-ethylbenzene (**6'**, 70.5 mg, 0.50 mmol), sodium dispersion (26.1 wt%, 94.5 mg, 1.07 mmol), and $Fe(acac)_3$ (9.2 mg, 26 μmol). The crude product was purified by column chromatography on silica gel (hexane only) to afford the title compound as a colorless oil (33.2 mg, 0.16 mmol, 63%). The spectral data of product **6** was in good agreement with the literature[9].

^1H NMR (500 MHz, $CDCl_3$): δ 7.42–7.40 (m, 4H), 7.34 (dd, J = 7.7, 7.7 Hz, 2H), 7.18 (d, J = 8.1 Hz, 2H), 2.71 (q, J = 7.6 Hz, 4H), 1.28 (t, J = 7.7 Hz, 6H).

^{13}C NMR (126 MHz, $CDCl_3$): δ 144.6, 141.5, 128.7, 126.9, 126.7, 124.6, 29.0, 15.7.

4,4'-Dibutyl-1,1'-biphenyl (7)

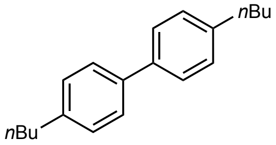

The general procedure was applied to 1-butyl-4-chlorobenzene (**7'**, 84.7 mg, 0.50 mmol), sodium dispersion (25.9 wt%, 94.5 mg, 1.07 mmol), and $Fe(acac)_3$ (9.0 mg, 25 μmol). The crude product was purified by column chromatography on silica gel (hexane only) to afford the title compound as a white solid (43.7 mg, 0.16 mmol, 65%). The spectral data of product **7** was in good agreement with the literature[10].

^1H NMR (500 MHz, $CDCl_3$): δ 7.48 (d, J = 8.1 Hz, 4H), 7.22 (d, J = 8.0 Hz, 4H), 2.63 (t, J = 7.7 Hz, 4H), 1.63 (tt, J = 7.7, 7.7 Hz, 4H), 1.38 (qt, J = 7.3, 7.3 Hz, 4H), 0.93 (t, J = 7.4 Hz, 6H).

^{13}C NMR (126 MHz, $CDCl_3$): δ 141.7, 138.5, 128.7, 126.8, 35.3, 33.7, 22.4, 14.0.

4,4'-Di-*tert*-butyl-1,1'-biphenyl (8)

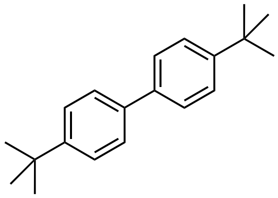

The general procedure was applied to 1-(*tert*-butyl)-4-chlorobenzene (**8'**, 83.9 mg, 0.50 mmol), sodium dispersion (26.0 wt%, 93.1 mg, 1.05 mmol), and $Fe(acac)_3$ (9.1 mg, 26 μmol). The crude product was purified by column chromatography on silica gel (hexane only) to afford the title compound as a white solid (47.1 mg, 0.18 mmol, 71%). The spectral data of product **8** was in good agreement with the literature[8].

^{1}H NMR (500 MHz, $CDCl_3$): δ 7.52 (d, J = 8.6 Hz, 4H), 7.44 (d, J = 8.6 Hz, 4H), 1.35 (s, 18H).

^{13}C NMR (126 MHz, $CDCl_3$): δ 149.9, 138.2, 126.7, 125.6, 34.5, 31.4.

4,4'-Bis(triethylsilyl)-1,1'-biphenyl (9)

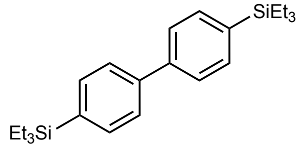

The general procedure (step 2, the reaction temperature was 50 °C) was applied to (4-chlorophenyl)triethylsilane (**9'**, 114.2 mg, 0.50 mmol), sodium dispersion (25.9 wt%, 93.5 mg, 1.05 mmol), and $Fe(acac)_3$ (9.0 mg, 25 μmol). The crude product was purified by column chromatography on silica gel (hexane only) and GPC (chloroform) to afford the title compound as colorless oil (38.1 mg, 0.10 mmol, 40%). The spectral data of product **9** was in good agreement with the literature[11].

^{1}H NMR (500 MHz, $CDCl_3$): δ 7.60–7.55 (m, 8H), 0.99 (t, J = 7.7 Hz, 12H), 0.82 (q, J = 8.0 Hz, 18H).

^{13}C NMR (126 MHz, $CDCl_3$): δ 141.4, 136.2, 134.6, 126.3, 7.4, 3.4.

4,4'-Bis(*tert*-butyldimethylsilyl)-1,1'-biphenyl (10)

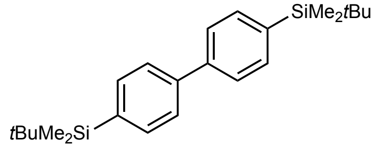

The general procedure (step 2, the reaction temperature was 50 °C) was applied to *tert*-butyl(4-chlorophenyl)dimethylsilane (**10'**, 113.7 mg, 0.50 mmol), sodium dispersion (25.9 wt%, 93.6 mg, 1.05 mmol), and $Fe(acac)_3$ (9.1 mg, 26 μmol). The crude product was purified by column chromatography on silica gel (hexane only) and GPC (chloroform) to afford the title compound **10** as a white solid (37.7 mg, 0.10 mmol, 39%).

Melting point: 83–85 °C.

^{1}H NMR (500 MHz, $CDCl_3$): δ 7.58 (brs, 8H), 0.90 (s, 18H), 0.30 (s, 12H).

^{13}C NMR (126 MHz, $CDCl_3$): δ 141.4, 136.7, 135.0, 126.1, 26.5, 17.0, –6.1.
GC-MS (EI): *m*/*z* (relative intensity): 382 (M^+, 2), 327 (12), 326 (33), 325 (100), 134 (17).
HRMS (APCI+): m/z Calcd. for $C_{24}H_{38}Si_2$ $[M]^+$: 382.2507; found: 382.2506.

4,4'-Di-*tert*-butoxy-1,1'-biphenyl (11)

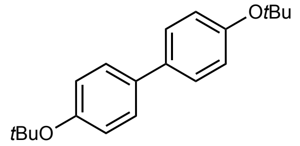

The general procedure (step 2, $Fe(acac)_3$ (10 mol%) was used) was applied to 1-(*tert*-butoxy)-4-chlorobenzene (**11'**, 92.6 mg, 0.50 mmol), sodium dispersion (26.0 wt%, 93.7 mg, 1.06 mmol), and $Fe(acac)_3$ (17.7 mg, 50 μmol). The crude product was purified by column chromatography on silica gel (hexane/EtOAc = 50:1) to afford the title compound as a white solid (30.1 mg, 0.10 mmol, 40%). The spectral data of product **11** was in good agreement with the literature[12].
^1H NMR (500 MHz, $CDCl_3$): δ 7.46 (d, J = 8.6 Hz, 4H), 7.03 (d, J = 8.6 Hz, 4H), 1.37 (s, 18H).
^{13}C NMR (126 MHz, $CDCl_3$): δ 154.6, 135.8, 127.2, 124.3, 78.5, 28.9.

2,2'-Dimethoxy-1,1'-biphenyl (12)

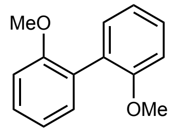

The general procedure (step 1, the reaction time was 2 h) was applied to 1-chloro-2-methoxybenzene (**12'**, 72.0 mg, 0.50 mmol), sodium dispersion (25.9 wt%, 93.8 mg, 1.06 mmol), and $Fe(acac)_3$ (9.0 mg, 25 μmol). The crude product was purified by column chromatography on silica gel (hexane/EtOAc = 25:1) to afford the title compound as a white solid (19.8 mg, 0.09 mmol, 37%). The spectral data of product **12** was in good agreement with the literature[7].
^1H NMR (500 MHz, $CDCl_3$): δ 7.34–7.31 (m, 2H), 7.24 (dd, J = 7.5, 1.7 Hz, 2H), 7.02–6.99 (m, 2H), 6.97 (d, J = 8.6 Hz, 2H), 3.77 (s, 6H).
^{13}C NMR (126 MHz, $CDCl_3$): δ 157.0, 131.4, 128.6, 127.8, 120.3, 111.1, 55.7.

N^4,N^4,$N^{4'}$,$N^{4'}$-Tetramethyl-[1,1'-biphenyl]-4,4'-diamine (13)

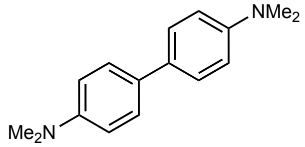

The general procedure (step 1, the reaction time was 2 h) was applied to 4-chloro-*N*,*N*-dimethylaniline (**13'**, 78.0 mg, 0.50 mmol), sodium dispersion (25.9 wt%, 93.9 mg, 1.06 mmol), and $Fe(acac)_3$ (8.6 mg, 24 μmol). The crude product was purified by column chromatography on silica gel (hexane/EtOAc = 15:1) to afford the title compound as a white solid (32.5 mg, 0.14 mmol, 54%). The spectral data of product **13** was in good agreement with the literature[13].

^{1}H NMR (500 MHz, $CDCl_3$): δ 7.45 (d, J = 8.6 Hz, 4H), 6.78 (d, J = 9.2 Hz, 4H), 2.95 (s, 12H).
^{13}C NMR (126 MHz, $CDCl_3$): δ 149.2, 129.7, 126.9, 113.0, 40.7.

N^3,N^3,$N^{3'}$,$N^{3'}$-Tetramethyl-[1,1'-biphenyl]-3,3'-diamine (14)

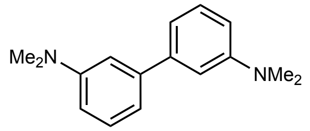

The general procedure (step 1, the reaction time was 2 h) was applied to 3-chloro-*N*,*N*-dimethylaniline (**14’**, 78.1 mg, 0.50 mmol), sodium dispersion (25.9 wt%, 93.5 mg, 1.05 mmol), and $Fe(acac)_3$ (8.8 mg, 25 µmol). The crude product was purified by column chromatography on silica gel (hexane/EtOAc = 15:1) to afford the title compound as colorless oil (24.3 mg, 0.10 mmol, 40%). The spectral data of product **14** was in good agreement with the literature[14].
^{1}H NMR (500 MHz, $CDCl_3$): δ 7.29 (dd, J = 8.0, 8.0 Hz, 2H), 6.95–6.94 (m, 4H), 6.74–6.72 (m, 2H), 2.99 (s, 12H).
^{13}C NMR (126 MHz, $CDCl_3$): δ 150.8, 143.3, 129.2, 116.1, 111.9, 111.5, 40.7.

3,3',4,4'-Tetramethyl-1,1'-biphenyl (15)

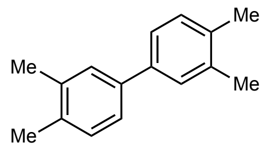

The general procedure was applied to 4-chloro-1,2-dimethylbenzene (**15’**, 70.6 mg, 0.50 mmol), sodium dispersion (25.9 wt%, 93.9 mg, 1.06 mmol), and $Fe(acac)_3$ (9.0 mg, 25 µmol). The crude product was purified by column chromatography on silica gel (hexane only) to afford the title compound as a white solid (37.4 mg, 0.18 mmol, 71%). The spectral data of product **15** was in good agreement with the literature[9].
^{1}H NMR (500 MHz, $CDCl_3$): δ 7.35 (s, 2H), 7.31 (dd, J = 7.6, 1.5 Hz, 2H), 7.17 (d, J = 7.4 Hz, 2H), 2.31 (s, 6H), 2.28 (s, 6H).
^{13}C NMR (126 MHz, $CDCl_3$): δ 138.9, 136.7, 135.3, 129.9, 128.2, 124.3, 19.9, 19.4.

3,3',5,5'-Tetra-*tert*-butyl-1,1'-biphenyl (16)

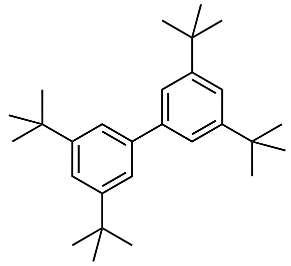

The general procedure (step 1, the reaction time was 2 h) was applied to 1,3-di-*tert*-butyl-5-chlorobenzene (**16’**, 112.8 mg, 0.50 mmol), sodium dispersion (25.9 wt%, 94.6 mg, 1.07 mmol), and $Fe(acac)_3$ (9.1 mg, 26 µmol). The crude product was purified by column chromatography on

silica gel (hexane only) to afford the title compound as colorless crystalline solid (64.8 mg, 0.17 mmol, 68%). The spectral data of product **16** was in good agreement with the literature[15].

^{1}H NMR (500 MHz, $CDCl_3$): δ 7.43 (dd, J = 1.7, 1.7 Hz, 2H), 7.38 (d, J = 1.7 Hz, 4H), 1.39 (s, 36H).

^{13}C NMR (126 MHz, $CDCl_3$): δ 150.8, 142.3, 122.13, 122.08, 35.0, 31.5.

1,1'-Biphenyl-d_{10} (17)

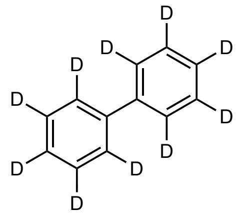

The general procedure was applied to 1-chlorobenzene-2,3,4,5,6-d_5 (**17'**, 99 atom %D, 59.4 mg, 0.51 mmol), sodium dispersion (25.9 wt%, 93.6 mg, 1.05 mmol), and $Fe(acac)_3$ (8.8 mg, 25 μmol). The crude product was purified by column chromatography on silica gel (pentane only) to afford the title compound as a white solid (26.8 mg, 0.16 mmol, 65%). The spectral data of product **17** was in good agreement with the literature[16].

^{2}H NMR (77 MHz, $CHCl_3$): δ 7.69 (brs, 4D), 7.53 (brs, 4D), 7.43 (brs, 2D).

^{13}C NMR (126 MHz, $CDCl_3$): δ 141.1, 128.3 (t, J = 24.0 Hz), 127.0–126.6 (m, 2C).

9,9,9',9'-Tetramethyl-9*H*,9*H'*-2,2'-bifluorene (18)

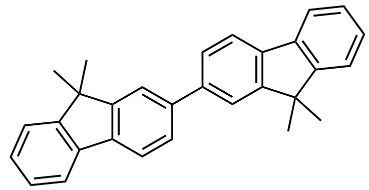

The general procedure (step 1, the reaction temperature and time were 60 °C and 2 h) was applied to 2-chloro-9,9-dimethyl-9*H*-fluorene (**18'**, 114.4 mg, 0.50 mmol), sodium dispersion (26.0 wt%, 93.9 mg, 1.06 mmol), and $Fe(acac)_3$ (8.9 mg, 25 μmol). The crude product was purified by column chromatography on silica gel (hexane/chloroform = 10:1) to afford the title compound as a white solid (40.8 mg, 0.11 mmol, 42%). The spectral data of product **18** was in good agreement with the literature[17].

^{1}H NMR (500 MHz, $CDCl_3$): δ 7.79 (d, J = 7.5 Hz, 2H), 7.75 (d, J = 6.9 Hz, 2H), 7.69 (brs, 2H), 7.63 (dd, J = 7.7, 1.4 Hz, 2H), 7.45 (d, J = 7.5 Hz, 2H), 7.37–7.30 (m, 4H), 1.56 (s, 12H).

^{13}C NMR (126 MHz, $CDCl_3$): δ 154.3, 153.9, 140.8, 138.9, 138.4, 127.2, 127.0, 126.3, 122.6, 121.4, 120.3, 120.0, 47.0, 27.3.

5',5''-Dimethyl-1,1':3',1'':3'',1'''-quaterphenyl' (19)

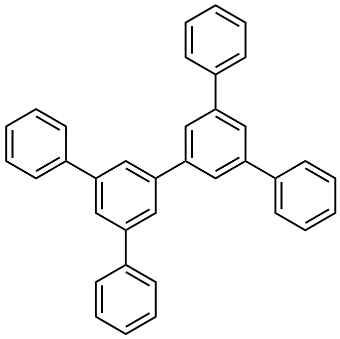

The general procedure (step 1, the reaction time was 3 h) was applied to 5'-chloro-1,1':3',1''-terphenyl (**19'**, 132.4 mg, 0.50 mmol), sodium dispersion (26.0 wt%, 94.5 mg, 1.07 mmol), and $Fe(acac)_3$ (8.9 mg, 25 μmol). The crude product was purified by column chromatography on silica gel (hexane/chloroform = 10:1) to afford the title compound as a white solid (70.9 mg, 0.15 mmol, 62%). The spectral data of product **19** was in good agreement with the literature[6].

^{1}H NMR (500 MHz, $CDCl_3$): δ 7.88 (d, *J* = 1.7 Hz, 4H), 7.83 (dd, *J* = 1.7, 1.7 Hz, 2H), 7.72 (d, *J* = 6.9 Hz, 8H), 7.48 (dd, *J* = 7.5, 7.5 Hz, 8H), 7.39 (dd, *J* = 7.5, 7.5 Hz, 4H).

^{13}C NMR (126 MHz, $CDCl_3$): δ 142.5, 142.3, 141.0, 128.9, 127.6, 127.4, 125.4, 125.3.

***N*^{4},*N*^{4},*N*$^{4'}$,*N*$^{4'}$-Tetraphenyl-[1,1'-biphenyl]-4,4'-diamine (20)**

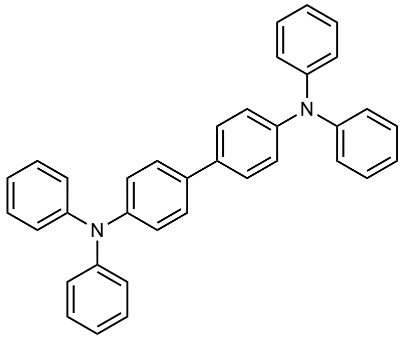

The general procedure (step 1, the reaction time was 5 h) was applied to 4-chloro-*N*,*N*-diphenylaniline (**20'**, 139.9 mg, 0.50 mmol), sodium dispersion (26.0 wt%, 93.9 mg, 1.06 mmol), and $Fe(acac)_3$ (9.0 mg, 25 μmol). The crude product was purified by column chromatography on silica gel (hexane/EtOAc/chloroform = 50:1:1) to afford the title compound as a white solid (84.5 mg, 0.17 mmol, 69%). The spectral data of product **20** was in good agreement with the literature[18].

^{1}H NMR (500 MHz, $CDCl_3$): δ 7.43 (d, *J* = 9.2 Hz, 4H), 7.25 (dd, *J* = 8.0, 8.0 Hz, 8H), 7.13–7.10 (m, 12H), 7.01 (dd, *J* = 7.4, 7.4 Hz, 4H).

^{13}C NMR (126 MHz, $CDCl_3$): δ 147.7, 146.7, 134.7, 129.2, 127.3, 124.3, 124.1, 122.8.

5. Iron-catalysed oxidative homocoupling of organosodium prepared by halogen–sodium exchange

General procedure for iron-catalysed oxidative homocoupling of organosodium compounds

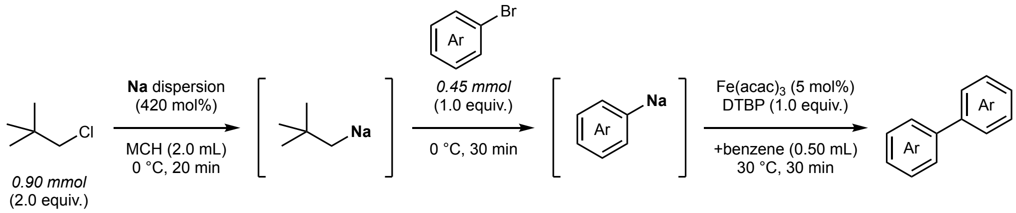

Step 1: preparation of the organosodium compounds through halogen–sodium exchange

In a dry Schlenk tube equipped with a glass-coated stirring bar, neopentyl chloride (95.9 mg, 0.90 mmol, 2.0 equiv.) was added to a mixture of MCH (2.0 mL) and sodium dispersion (ca. 26 wt%, ca. 167.1 mg, 1.89 mmol, 420 mol% to organic bromides, 210 mol% to neopentyl chloride) under nitrogen at 0 °C. After stirring at 0 °C for 20 min, the aryl bromide (0.45 mmol, 1.0 equiv.) was added to the suspension of neopentylsodium in MCH (2.0 mL) at 0 °C, and the reaction mixture was stirred for 30 min at 0 °C to form the corresponding arylsodium compound with full conversion of the starting material. Quantitative formation was indicated by GC and GC-MS analysis of a separate reaction after quenching with D_2O.

Step 2: iron-catalysed oxidative homocoupling of organosodium compounds

$Fe(acac)_3$ (7.9 mg, 23 μmol, 5 mol%), benzene (0.50 mL), and di-*tert*-butyl peroxide (DTBP, 82 μL, 0.45 mmol, 1.0 equiv.) were added to the suspension of the aryl- or alkenylsodium compound in MCH (2.0 mL) at 0 °C, and the reaction mixture was stirred at 30 °C for 30 min. The reaction was quenched with D_2O (0.2 mL) (to estimate the amount of active arylsodium remaining in the reaction mixture) and then with a saturated aqueous solution of NH_4Cl (1 mL) at 0 °C. After extraction with EtOAc or CH_2Cl_2 for three times, the combined organic layers were passed through a plug of silica gel with EtOAc or CH_2Cl_2 and concentrated under reduced pressure. The crude product was purified by column chromatography on silica gel using the indicated eluent to afford the desired compound.

Comment: The aryl- and alkenylsodium compounds described in this section are not accessible by the reductive sodiation method using organic chlorides and sodium dispersion. The halogen–sodium exchange method is essential for the preparation of these organosodium compounds.

2,2'-Binaphthalene (21)

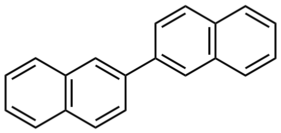

The general procedure was applied to neopentyl chloride (97.2 mg, 0.91 mmol), sodium dispersion (26.0 wt%, 171.0 mg, 1.93 mmol), 2-bromonaphthalene (**21'**, 93.4 mg, 0.45 mmol), and Fe(acac)$_3$ (8.1 mg, 23 μmol). The crude product was purified by column chromatography on silica gel (hexane/chloroform = 50:1) to afford the title compound as a white solid (31.7 mg, 0.12 mmol, 55%). The spectral data of product **21** was in good agreement with the literature[7].

^{1}H NMR (500 MHz, CDCl$_3$): δ 8.17 (s, 2H), 7.96–7.92 (m, 4H), 7.89–7.87 (m, 4H), 7.54–7.48 (m, 4H).

^{13}C NMR (126 MHz, CDCl$_3$): δ 138.4, 133.7, 132.6, 128.5, 128.2, 127.7, 126.3, 126.1, 126.0, 125.7.

1,1'-Binaphthalene (22)

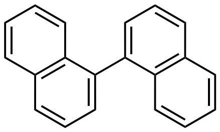

The general procedure was applied to neopentyl chloride (96.7 mg, 0.91 mmol), sodium dispersion (26.0 wt%, 168.2 mg, 1.90 mmol), 1-bromonaphthalene (**22'**, 93.3 mg, 0.45 mmol), and Fe(acac)$_3$ (8.1 mg, 23 μmol). The crude product was purified by column chromatography on silica gel (hexane/chloroform = 50:1) to afford the title compound as a white solid (30.8 mg, 0.12 mmol, 54%). The spectral data of product **22** was in good agreement with the literature[6].

^{1}H NMR (500 MHz, CDCl$_3$): δ 7.94–7.92 (m, 4H), 7.57 (dd, J = 8.0, 6.9 Hz, 2H), 7.49–7.44 (m, 4H), 7.39 (d, J = 8.0 Hz, 2H), 7.28–7.25 (m, 2H).

^{13}C NMR (126 MHz, CDCl$_3$): δ 138.4, 133.5, 132.8, 128.1, 127.9, 127.8, 126.5, 126.0, 125.8, 125.4.

4,4'-Bis((triisopropylsilyl)oxy)-1,1'-biphenyl (23)

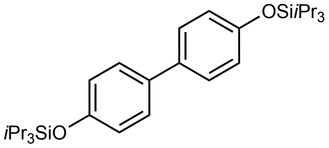

The general procedure was applied to neopentyl chloride (96.4 mg, 0.90 mmol), sodium dispersion (26.1 wt%, 167.0 mg, 1.90 mmol), (4-bromophenoxy)triisopropylsilane (**23'**, 148.4 mg, 0.45 mmol), and Fe(acac)$_3$ (8.3 mg, 24 μmol). The crude product was purified by column chromatography on silica gel (hexane only) to afford the title compound **23** as a colorless oil (58.0 mg, 0.12 mmol, 52%).

^{1}H NMR (500 MHz, CDCl$_3$): δ 7.40 (d, J = 8.6 Hz, 4H), 6.91 (d, J = 8.6 Hz, 4H), 1.28 (sept, J = 7.5 Hz, 6H), 1.12 (d, J = 7.5 Hz, 36H).

^{13}C NMR (126 MHz, $CDCl_3$): δ 155.1, 133.7, 127.5, 120.0, 17.9, 12.7.
HRMS (APCI+): *m/z* Calcd. for $C_{30}H_{50}O_2Si_2$ $[M+H]^+$: 499.3422; found: 499.3420.

3,3'-Bis((triisopropylsilyl)oxy)-1,1'-biphenyl (24)

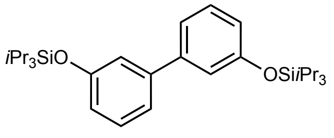

The general procedure was applied to neopentyl chloride (96.5 mg, 0.91 mmol), sodium dispersion (26.1 wt%, 167.9 mg, 1.91 mmol), (3-bromophenoxy)triisopropylsilane (**24'**, 148.1 mg, 0.45 mmol), and $Fe(acac)_3$ (8.0 mg, 23 μmol). The crude product was purified by column chromatography on silica gel (hexane only) to afford the title compound **24** as a colorless oil (63.9 mg, 0.13 mmol, 57%).
^{1}H NMR (500 MHz, $CDCl_3$): δ 7.26 (dd, *J* = 7.7, 7.7 Hz, 2H), 7.4 (d, *J* = 8.1 Hz, 2H), 7.08 (dd, *J* = 2.0, 2.0 Hz, 2H), 6.86 (ddd, *J* = 8.0, 2.3, 1.2 Hz, 2H), 1.29 (sept, *J* = 7.4 Hz, 6H), 1.12 (d, *J* = 7.4 Hz, 36H).
^{13}C NMR (126 MHz, $CDCl_3$): δ 156.3, 142.4, 129.6, 119.8, 118.9, 118.5, 17.7, 12.7.
HRMS (APCI+): *m/z* Calcd. for $C_{30}H_{50}O_2Si_2$ $[M+H]^+$: 499.3422; found: 499.3400.

3,3'-Bis(2-(triisopropylsilyl)oxy)ethyl)-1,1'-biphenyl (25)

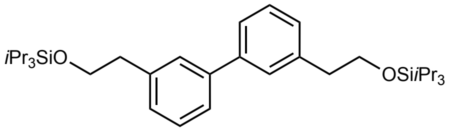

The general procedure was applied to neopentyl chloride (95.9 mg, 0.90 mmol), sodium dispersion (26.0 wt%, 168.7 mg, 1.91 mmol), (3-bromophenethoxy)trisisopropylsilane (**25'**, 160.9 mg, 0.45 mmol), and $Fe(acac)_3$ (7.9 mg, 22 μmol). The crude product was purified by column chromatography on silica gel (hexane/EtOAc = 100:1) to afford the title compound **25** as a yellow viscous oil (65.7 mg, 0.12 mmol, 53%).
^{1}H NMR (500 MHz, $CDCl_3$): δ 7.43 (brs, 2H), 7.41 (d, *J* = 7.5 Hz, 2H), 7.33 (dd, *J* = 7.7, 7.7 Hz, 2H), 7.20 (d, *J* = 7.5 Hz, 2H), 3.92 (t, *J* = 7.2 Hz, 4H), 2.92 (t, *J* = 7.2 Hz, 4H), 1.08–1.03 (m, 42H).
^{13}C NMR (126 MHz, $CDCl_3$): δ 141.3, 139.6, 128.5, 128.1, 128.0, 125.0, 64.9, 39.9, 18.0, 12.0.
HRMS (APCI+): *m/z* Calcd. for $C_{34}H_{58}O_2Si_2$ $[M+H]^+$: 555.4048; found: 555.4048.

4,4'-Dimorpholino-1,1'biphenyl (26)

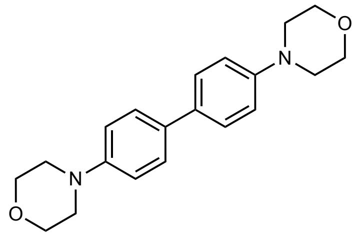

The general procedure was applied to neopentyl chloride (96.4 mg, 0.90 mmol), sodium dispersion (26.1 wt%, 167.5 mg, 1.90 mmol), 4-(4-bromopheyl)morpholine (**26'**, 109.4 mg, 0.45 mmol), and Fe(acac)$_3$ (8.2 mg, 23 μmol). The crude product was purified by column chromatography on silica gel (hexane/EtOAc = 3:1 and 1% Et$_3$N) to afford the title compound as a pale-yellow solid (42.6 mg, 0.13 mmol, 58%).
Melting point: decomposition started above 268 °C.
^{1}H NMR (500 MHz, CDCl$_3$): δ 7.49 (d, J = 8.6 Hz, 4H), 6.96 (d, J = 8.6 Hz, 4H), 3.89–3.87 (m, 8H), 3.20–3.18 (m, 8H).
^{13}C NMR (126 MHz, CDCl$_3$): δ 150.0, 132.5, 127.2, 115.9, 66.9, 49.3.
HRMS (APCI+): m/z Calcd. for $C_{20}H_{24}N_2O_2$ [M+H]$^+$: 325.1911; found: 325.1907.

(1*E*,3*E*)-1,4-Diphenylbuta-1,3-diene (27)

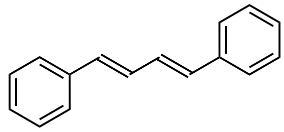

The general procedure (step 2, Fe(acac)$_3$ (10 mol%) was used) was applied to neopentyl chloride (96.5 mg, 0.91 mmol), sodium dispersion (26.1 wt%, 167.7 mg, 1.90 mmol), (*E*)-(2-bromovinyl)benzene (**27'**, 82.6 mg, 0.45 mmol), and Fe(acac)$_3$ (16.1 mg, 46 μmol). The crude product was purified by column chromatography on silica gel (hexane/chloroform = 20:1) and GPC (chloroform) to afford the title compound as a white solid (17.9 mg, 0.9 mmol, 39%). The spectral data of product **27** was in good agreement with the literature[10].
^{1}H NMR (500 MHz, CDCl$_3$): δ 7.43 (d, J = 7.5 Hz, 4H), 7.32 (dd, J = 7.7, 7.7 Hz, 4H), 7.22 (dd, J = 7.2, 7.2 Hz, 2H), 6.98–6.92 (m, 2H), 6.69–6.63 (m, 2H).
^{13}C NMR (126 MHz, CDCl$_3$): δ 137.3, 132.8, 129.2, 128.6, 127.5, 126.4.

7,7'-Di-*tert*-butyl-2,2'-bipyrene (28)

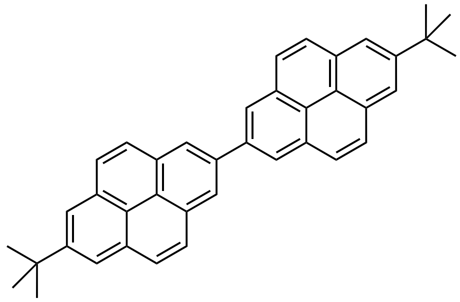

The general procedure was applied to neopentyl chloride (96.7 mg, 0.91 mmol), sodium dispersion (26.0 wt%, 167.2 mg, 1.89 mmol), 2-bromo-7-(*tert*-butyl)pyrene (**28'**, 152.0 mg, 0.45 mmol), and Fe(acac)$_3$ (8.0 mg, 23 μmol). The crude product was purified by column chromatography on silica gel (hexane/chloroform = 10:1) to afford the title compound **28** as a white solid (80.7 mg, 0.16 mmol, 70%).
Melting point: >300 °C.

^1H NMR (500 MHz, $CDCl_3$): δ 8.65 (s, 4H), 8.26 (s, 4H), 8.18 (d, J = 9.2 Hz, 4H), 8.12 (d, J = 8.6 Hz, 4H), 1.61 (s, 18H).
^{13}C NMR (126 MHz, $CDCl_3$): δ 149.2, 139.0, 131.5, 131.0, 128.1, 127.5, 124.4, 123.9, 122.9, 122.5, 35.3, 32.0.
HRMS (APCI+): m/z Calcd. for $C_{40}H_{34}$ $[M+H]^+$: 515.2733; found: 515.2730.

4,4'-Bis(2,2-diphenylvinyl)-1,1'-biphenyl (29)

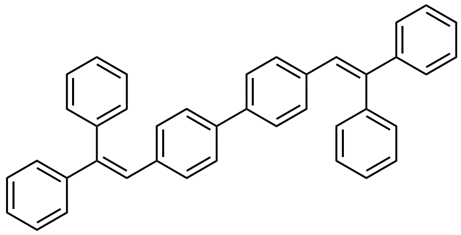

The general procedure was applied to neopentyl chloride (96.4 mg, 0.90 mmol), sodium dispersion (26.1 wt%, 167.2 mg, 1.90 mmol), (2-(4-bromophenyl)ethene-1,1-diyl)dibenzene (**29'**, 150.5 mg, 0.45 mmol), and $Fe(acac)_3$ (8.0 mg, 24 µmol). The crude product was purified by column chromatography on silica gel (hexane/chloroform/EtOAc = 100:5:2) to afford the title compound as a pale-yellow solid (79.5 mg, 0.16 mmol, 69%). The spectral data of product **29** was in good agreement with the literature[19].
^1H NMR (500 MHz, $CDCl_3$): δ 7.33–7.26 (m, 20H), 7.23–7.21 (m, 4H), 7.03 (d, J = 8.6 Hz, 4H), 6.97 (s, 2H).
^{13}C NMR (126 MHz, $CDCl_3$): δ 143.3, 142.6, 140.4, 138.6, 136.4, 130.3, 129.9, 128.7, 128.2, 127.7, 127.6, 127.5, 127.4, 126.2.

4,4'-Di(9*H*-carbazol-9-yl)-1,1'-biphenyl (30)

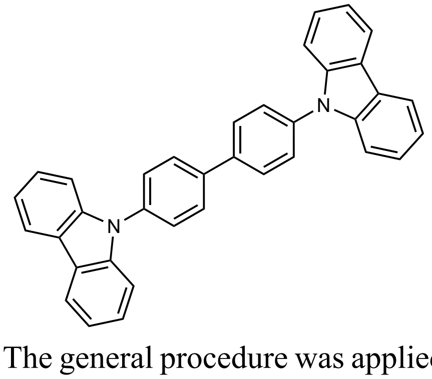

The general procedure was applied to neopentyl chloride (96.4 mg, 0.90 mmol), sodium dispersion (26.0 wt%, 168.3 mg, 1.90 mmol), 9-(4-bromophenyl)-9*H*-carbazole (**30'**, 145.4 mg, 0.45 mmol), and $Fe(acac)_3$ (7.9 mg, 22 µmol). The crude product was purified by column chromatography on silica gel (hexane/EtOAc/chloroform = 50:1:1) to afford the title compound as a white solid (80.6 mg, 0.17 mmol, 74%). The spectral data of product **30** was in good agreement with the literature[19].

^{1}H NMR (500 MHz, $CDCl_3$): δ 8.17 (d, *J* = 7.5 Hz, 4H), 7.87 (d, *J* = 8.6 Hz, 4H), 7.68 (d, *J* = 8.0 Hz, 4H), 7.50 (d, *J* = 8.0 Hz, 4H), 7.44 (ddd, *J* = 7.7, 7.7, 1.2 Hz, 4H), 7.31 (dd, *J* = 7.5, 7.5 Hz, 4H).

^{13}C NMR (126 MHz, $CDCl_3$): δ 140.8, 139.2, 137.2, 128.5, 127.4, 126.0, 123.5, 120.4, 120.1, 109.8.

6. Iron-catalysed cross coupling of arylsodium compounds with alkyl halides

General procedure for iron-catalysed cross coupling of arylsodium with alkyl halides

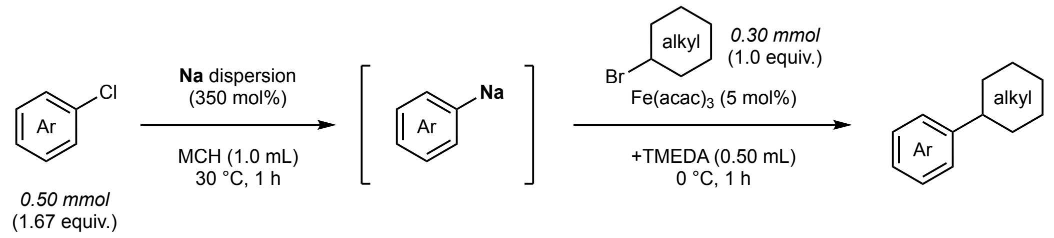

Step 1: preparation of the arylsodium compound.
In a dry Schlenk tube equipped with a glass-coated stirring bar, the aryl chloride (0.50 mmol, if liquid) was added to a mixture of MCH (1.0 mL) and sodium dispersion (ca. 26 wt%, 1.05 mmol, 350 mol% to alkyl halides; 210 mol% to aryl chlorides) under nitrogen. When the starting material is a solid, the sodium dispersion was added to a mixture of the aryl chloride (0.50 mmol) and MCH (1.0 mL). After stirring at 30 °C for the indicated time, the corresponding arylsodium was generated.

Step 2: iron-catalysed cross coupling of arylsodium compounds with alkyl halides.
$Fe(acac)_3$ (5.3 mg, 15 µmol, 5 mol%), TMEDA (0.50 mL), and alkyl halide (0.30 mmol, 1.0 equiv.) were added to the suspension of the arylsodium compound in MCH (1.0 mL) at 0 °C, and the reaction mixture was stirred at 0 °C for 1 h. The reaction was quenched with D_2O (0.1 mL) (to estimate the amount of active arylsodium remaining in the reaction mixture) and then with a saturated aqueous solution of NH_4Cl (1 mL) at 0 °C. After extraction with EtOAc, Et_2O, or CH_2Cl_2 for three times, the combined organic layers were passed through a plug of silica gel with EtOAc, Et_2O, or CH_2Cl_2 and concentrated under reduced pressure. The crude product was purified by column chromatography on silica gel using the indicated eluent to afford the desired compound.

1-(*tert*-Butyl)-4-cyclohexylbenzene (31)

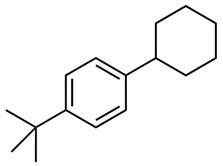

The general procedure was applied to 1-(*tert*-butyl)-4-chlorobenzene (**8'**, 84.2 mg, 0.50 mmol), sodium dispersion (26.0 wt%, 94.8 mg, 1.07 mmol), $Fe(acac)_3$ (5.3 mg, 15 µmol), and bromocyclohexane (37 µL, 0.30 mmol). The crude product was purified by column chromatography on silica gel (hexane only) to afford the title compound as a colorless oil (53.1 mg, 0.25 mmol, 82%). The spectral data of product **31** was in good agreement with the literature[20].
^{1}H NMR (500 MHz, $CDCl_3$): δ 7.31 (d, J = 8.0 Hz, 4H), 7.14 (d, J = 8.6 Hz, 4H), 2.50–2.44 (m, 1H), 1.89–1.82 (m, 4H), 1.76–1.72 (m, 1H), 1.45–1.34 (m, 4H), 1.31 (s, 9H), 1.28–1.20 (m, 1H).

^{13}C NMR (126 MHz, $CDCl_3$): δ 148.4, 145.0, 126.4, 125.1, 44.0, 34.5, 34.3, 31.4, 27.0, 26.2.

1-Cyclohexyl-4-methylbenzene (32)

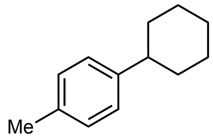

The general procedure (step 2, $Fe(acac)_3$ (10 mol%) was used) was applied to 1-chloro-4-methylbenzene (**2'**, 63.7 mg, 0.50 mmol), sodium dispersion (26.1 wt%, 92.9 mg, 1.05 mmol), $Fe(acac)_3$ (10.3 mg, 29 µmol), and bromocyclohexane (37 µL, 0.30 mmol). The crude product was purified by column chromatography on silica gel (pentane only) and GPC (chloroform) to afford the title compound as a colorless oil (28.3 mg, 0.16 mmol, 54%). The spectral data of product **32** was in good agreement with the literature[21].

^{1}H NMR (500 MHz, $CDCl_3$): δ 7.10 (brs, 4H), 2.48–2.42 (m, 1H), 2.31 (s, 3H), 1.86–1.81 (m, 4H), 1.75–1.72 (m, 1H), 1.44–1.33 (m, 4H), 1.28–1.22 (m, 1H).

^{13}C NMR (126 MHz, $CDCl_3$): δ 145.1, 133.1, 128.9, 126.7, 44.1, 34.6, 26.9, 26.2, 21.0.

1-Cyclohexyl-3-methylbenzene (33)

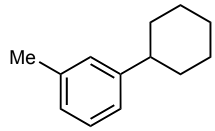

The general procedure was applied to 1-chloro-4-methylbenzene (**3'**, 63.8 mg, 0.50 mmol), sodium dispersion (25.9 wt%, 95.2 mg, 1.07 mmol), $Fe(acac)_3$ (5.2 mg, 15 µmol), and bromocyclohexane (37 µL, 0.30 mmol). The crude product was purified by column chromatography on silica gel (pentane only) to afford the title compound as a colorless oil (22.0 mg, 0.13 mmol, 42%). The spectral data of product **33** was in good agreement with the literature[22].

^{1}H NMR (500 MHz, $CDCl_3$): δ 7.18 (dd, J = 7.5, 7.5 Hz, 1H), 7.02–6.98 (m. 3H), 2.48–2.43 (m, 1H), 2.33 (s, 3H), 1.87–1.82 (m, 4H), 1.77–1.72 (m, 1H), 1.46–1.33 (m, 4H), 1.30–1.20 (m, 1H).

^{13}C NMR (126 MHz, $CDCl_3$): δ 148.1, 137.7, 128.2, 127.7, 126.5, 123.8, 44.6, 34.5, 26.9, 26.2, 21.5.

1-Cyclohexyl-4-ethylbenzene (34)

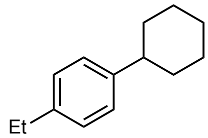

The general procedure was applied to 1-chloro-4-ethylbenzene (**5'**, 70.2 mg, 0.50 mmol), sodium dispersion (26.1 wt%, 94.0 mg, 1.07 mmol), $Fe(acac)_3$ (5.3 mg, 15 µmol), and bromocyclohexane (37 µL, 0.30 mmol). The crude product was purified by column chromatography on silica gel (pentane only) to afford the title compound as a colorless oil (45.9 mg, 0.24 mmol, 81%). The spectral data of product **34** was in good agreement with the literature[23].

^{1}H NMR (500 MHz, $CDCl_3$): δ 7.12 (brs, 4H), 2.61 (q, J = 7.5 Hz, 2H), 2.49–2.43 (m, 1H), 1.45–1.33 (m, 4H), 1.28–1.22 (m, 1H), 1.22 (t, J = 7.5 Hz, 3H).
^{13}C NMR (126 MHz, $CDCl_3$): δ 145.3, 141.5, 127.7, 126.7, 44.2, 34.5, 28.4, 27.0, 26.2, 15.5.

1-Cyclohexyl-3-ethylbenzene (35)

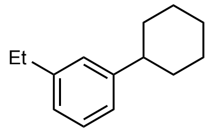

The general procedure was applied to 1-chloro-3-ethylbenzene (**6'**, 70.5 mg, 0.50 mmol), sodium dispersion (26.1 wt%, 93.8 mg, 1.06 mmol), Fe(acac)$_3$ (5.4 mg, 15 μmol), and bromocyclohexane (37 μL, 0.30 mmol.). The crude product was purified by column chromatography on silica gel (pentane only) to afford the title compound as a colorless oil (40.0 mg, 0.21 mmol, 71%). The spectral data of product **35** was in good agreement with the literature[23].
^{1}H NMR (500 MHz, $CDCl_3$): δ 7.20 (dd, J = 7.4, 7.4 Hz, 1H), 7.04–7.01 (m, 3H), 2.63 (q, J = 7.4 Hz, 2H), 2.50–2.44 (m, 1H), 1.47–1.34 (m, 4H), 1.29–1.23 (m, 1H), 1.23 (t, J = 7.8 Hz, 3H).
^{13}C NMR (126 MHz, $CDCl_3$): δ 148.1, 144.1, 128.2, 126.5, 125.3, 124.0, 44.6, 34.5, 28.9, 27.0, 26.2, 15.6.

1-Butyl-4-cyclohexylbenzene (36)

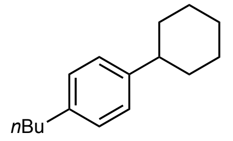

The general procedure was applied to 1-butyl-4-chlorobenzene (**7'**, 84.3 mg, 0.50 mmol), sodium dispersion (25.9 wt%, 94.7 mg, 1.07 mmol), Fe(acac)$_3$ (5.5 mg, 16 μmol), and bromocyclohexane (37 μL, 0.30 mmol). The crude product was purified by column chromatography on silica gel (hexane only) to afford the title compound as colorless oil (46.2 mg, 0.21 mmol, 71%). The spectral data of product **36** was in good agreement with the literature[23].
^{1}H NMR (500 MHz, $CDCl_3$): δ 7.12–7.08 (m, 4H), 2.57 (t, J = 7.8 Hz, 2H), 2.47–2.43 (m, 1H), 1.87–1.81 (m, 4H), 1.76–1.71 (m, 1H), 1.62–1.55 (m, 2H), 1.44–1.32 (m, 6H), 1.28–1.20 (m, 1H), 0.92 (t, J = 7.4 Hz, 3H).
^{13}C NMR (126 MHz, $CDCl_3$): δ 145.3, 140.2, 128.2, 126.6, 44.2, 35.2, 34.5, 33.7, 27.0, 26.2, 22.5, 14.0.

4-Cyclohexyl-1,2-dimethylbenzen (37)

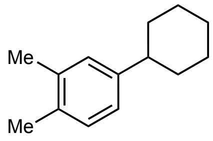

The general procedure (step 2, Fe(acac)$_3$ (10 mol%) was used) was applied to 4-chloro-1,2-dimethylbenzene (**15'**, 70.2 mg, 0.50 mmol), sodium dispersion (25.9 wt%, 93.5 mg, 1.05 mmol),

Fe(acac)$_3$ (10.4 mg, 29 μmol), and bromocyclohexane (37 μL, 0.30 mmol). The crude product was purified by column chromatography on silica gel (pentane only) to afford the title compound as a colorless oil (20.3 mg, 0.11 mmol, 36%).
^{1}H NMR (500 MHz, CDCl$_3$): δ 7.05 (d, J = 8.0 Hz, 1H), 6.98 (s, 1H), 6.95 (d, J = 7.5 Hz, 1H), 2.45–2.40 (m, 1H), 2.24 (s, 3H), 2.22 (s, 3H), 1.86–1.81 (m, 4H), 1.75–1.72 (m, 1H), 1.44–1.33 (m, 4H), 1.28–1.20 (m, 1H).
^{13}C NMR (126 MHz, CDCl$_3$): δ 145.7, 136.3, 133.8, 129.5, 128.2, 124.1, 44.2, 34.6, 27.0, 26.2, 19.8, 19.3.
HRMS (APCI+): m/z Calcd. for $C_{14}H_{20}$ $[M]^+$: 188.1560; found: 188.1556.

***tert*-Butyl(4-cyclohexylphenyl)dimethylsilane (38)**

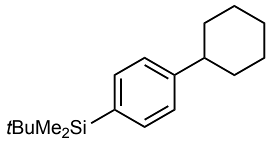

The general procedure (step 1, the reaction time was 3 h, step 2, Fe(acac)$_3$ (10 mol%) was used) was applied to *tert*-butyl(4-chlorophenyl)dimethylsilane (**10'**, 113.0 mg, 0.50 mmol), sodium dispersion (25.9 wt%, 94.7 mg, 1.07 mmol), Fe(acac)$_3$ (10.3 mg, 29 μmol), and bromocyclohexane (37 μL, 0.30 mmol). The crude product was purified by column chromatography on silica gel (hexane only) and GPC (chloroform) to afford the title compound **38** as a colorless crystalline solid (29.5 mg, 0.11 mmol, 36%).
Melting point: 60–61 °C.
^{1}H NMR (500 MHz, CDCl$_3$): δ 7.43 (d, J = 8.0 Hz, 2H), 7.18 (d, J = 8.0 Hz, 2H), 1.89–1.82 (m, 4H), 1.76–1.73 (m, 1H), 1.46–1.34 (m, 4H), 1.29–1.22 (m, 1H), 0.87 (s, 9H), 0.25 (s, 6H).
^{13}C NMR (126 MHz, CDCl$_3$): δ 148.6, 134.6, 134.5, 126.0, 44.5, 34.3, 26.9, 26.5, 26.2, 16.9, –6.1.
GC-MS (EI): m/z (relative intensity): 274 (M^+, 0.45), 218 (19), 217 (100), 73 (6), 59 (9).

1-Cyclohexyl-2-methoxybenzene (39)

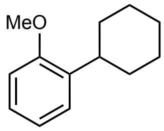

The general procedure (step 1, the reaction time was 2 h, step 2, Fe(acac)$_3$ (20 mol%) was used, and the reaction temperature was 30 °C) was applied to 1-chloro-2-methoxybenzene (**12'**, 71.7 mg, 0.50 mmol), sodium dispersion (26.1 wt%, 93.5 mg, 1.06 mmol), Fe(acac)$_3$ (21.4 mg, 61 μmol), and bromocyclohexane (37 μL, 0.30 mmol). The crude product was purified by column chromatography on silica gel (pentane only) to afford the title compound as a colorless oil (18.6 mg, 0.10 mmol, 33%). The spectral data of product **39** was in good agreement with the literature[21].
^{1}H NMR (500 MHz, CDCl$_3$): δ 7.19 (dd, J = 7.5, 1.8 Hz, 1H), 7.15 (ddd, J = 7.7, 7.7, 1.5 Hz, 1H), 6.92 (ddd, J = 7.5, 7.5, 1.2 Hz, 1H), 6.84 (dd, J = 8.1, 1.2 Hz, 1H), 3.81 (s, 3H), 2.95 (tt, J = 11.5, 2.9 Hz, 1H), 1.84–1.81 (m, 4H), 1.77–1.73 (m, 1H), 1.47–1.33 (m, 4H), 1.29–1.21 (m, 1H).

^{13}C NMR (126 MHz, $CDCl_3$): δ 156.7, 136.2, 126.5, 126.4, 120.5, 110.3, 55.3, 36.7, 33.2, 27.1, 26.4.

4-Cyclohexyl-*N*,*N*-dimethylaniline (40)

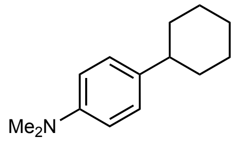

The general procedure (step 1, the reaction time was 2 h) was applied to 4-chloro-*N*,*N*-dimethylaniline (**13'**, 78.0 mg, 0.50 mmol), sodium dispersion (25.9 wt%, 94.8 mg, 1.07 mmol), $Fe(acac)_3$ (5.4 mg, 15 μmol), and bromocyclohexane (37 μL, 0.30 mmol). The crude product was purified by column chromatography on silica gel (hexane/EtOAc = 25:1) to afford the title compound as a colorless oil (34.0 mg, 0.17 mmol, 56%). The spectral data of product **40** was in good agreement with the literature[24].

^{1}H NMR (500 MHz, $CDCl_3$): δ 7.09 (d, *J* = 8.6 Hz, 2H), 6.70 (d, *J* = 8.6 Hz, 2H), 2.90 (s, 6H), 2.40 (tt, *J* = 11.5, 3.2 Hz, 1H), 1.86–1.80 (m, 4H), 1.73–1.71 (m, 1H), 1.42 (m, 4H), 1.26–1.21 (m, 1H).

^{13}C NMR (126 MHz, $CDCl_3$): δ 149.0, 136.5, 127.3, 112.9, 43.5, 40.9, 34.7, 27.0, 26.2.

3-Cyclohexyl-*N*,*N*-dimethylaniline (41)

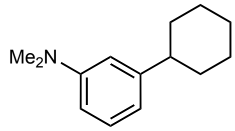

The general procedure (step 1, the reaction time was 2 h) was applied to 3-chloro-*N*,*N*-dimethylaniline (**14'**, 78.0 mg, 0.50 mmol), sodium dispersion (25.9 wt%, 94.6 mg, 1.07 mmol), $Fe(acac)_3$ (5.6 mg, 16 μmol), and bromocyclohexane (37 μL, 0.30 mmol). The crude product was purified by column chromatography on silica gel (hexane/EtOAc = 25:1) to afford the title compound as a colorless oil (25.4 mg, 0.12 mmol, 42%). The spectral data of product **41** was in good agreement with the literature[21].

^{1}H NMR (500 MHz, $CDCl_3$): δ 7.16 (dd, *J* = 8.0, 8.0 Hz, 1H), 6.60–6.57 (m, 3H), 2.93 (s, 6H), 2.45 (tt, *J* = 11.5, 3.4 Hz, 1H), 1.89–1.81 (m, 4H), 1.76–1.72 (m, 1H), 1.48–1.34 (m, 4H), 1.29–1.21 (m, 1H).

^{13}C NMR (126 MHz, $CDCl_3$): δ 150.7, 149.0, 128.9, 115.3, 111.5, 110.4, 45.2, 40.7, 34.5, 27.0, 26.2.

4-Cyclohexyl-*N*,*N*-diphenylaniline (42)

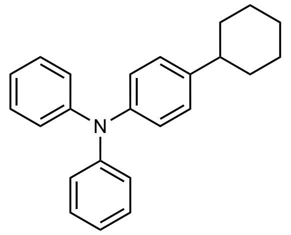

The general procedure (step 1, the reaction time was 5 h) was applied to 4-chloro-*N*,*N*-diphenylaniline (**20'**, 140.4 mg, 0.50 mmol), sodium dispersion (26.1 wt%, 93.5 mg, 1.06 mmol), $Fe(acac)_3$ (5.3 mg, 15 µmol), and bromocyclohexane (37 µL, 0.30 mmol). The crude product was purified by column chromatography on silica gel (hexane/EtOAc/chloroform = 50:1:1) and GPC (chloroform) to afford the title compound **42** as a white solid (69.7 mg, 0.21 mmol, 71%).
Melting point: 85–87 °C.
^{1}H NMR (500 MHz, $CDCl_3$): δ 7.20 (dd, *J* = 8.0, 8.0 Hz, 4H), 7.08–7.05 (m, 6H), 7.00 (d, *J* = 8.6 Hz, 2H), 6.95 (dd, *J* = 7.5, 7.5 Hz, 2H), 2.47–2.42 (m, 1H), 1.89–1.81 (m, 4H), 1.75–1.72 (m, 1H), 1.43–1.33 (m, 4H), 1.27–1.18 (m, 1H).
^{13}C NMR (126 MHz, $CDCl_3$): δ 148.0, 145.4, 142.9, 129.1, 127.5, 124.5, 123.7, 122.2, 43.9, 34.5, 26.9, 26.2.
HRMS (APCI+): *m*/*z* Calcd. for $C_{24}H_{25}N$ $[M+H]^+$: 328.2060; found: 328.2061.

1-(*tert*-Butyl)-4-cyclohexylbenzene (31)

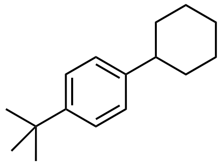

- Iodocyclohexane as an electrophile -
The general procedure was applied to 1-(*tert*-butyl)-4-chlorobenzene (**8'**, 84.8 mg, 0.50 mmol), sodium dispersion (26.1 wt%, 93.6 mg, 1.06 mmol), $Fe(acac)_3$ (5.4 mg, 15 µmol), and iodocyclohexane (39 µL, 0.30 mmol). The yield of the product was estimated by GC analysis with dodecane as an internal standard, after calibration of the response curves, to be 43%.

- Chlorocyclohexane as an electrophile -
The general procedure was applied to 1-(*tert*-butyl)-4-chlorobenzene (**8'**, 84.8 mg, 0.50 mmol), sodium dispersion (26.1 wt%, 93.1 mg, 1.06 mmol), $Fe(acac)_3$ (5.5 mg, 16 µmol), and chlorocyclohexane (36 µL, 0.30 mmol). The yield of the product was estimated by GC analysis with dodecane as an internal standard, after calibration of the response curves, to be 20%.

1-(*tert*-Butyl)-4-cyclopentylbenzene (43)

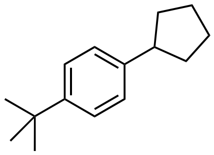

The general procedure was applied to 1-(*tert*-butyl)-4-chlorobenzene (**8’**, 84.5 mg, 0.50 mmol), sodium dispersion (26.1 wt%, 93.2 mg, 1.06 mmol), Fe(acac)$_3$ (5.5 mg, 16 μmol), and bromocyclopentane (32 μL, 0.30 mmol). The crude product was purified by column chromatography on silica gel (hexane only) to afford the title compound as a colorless oil (28.9 mg, 0.14 mmol, 48%). The spectral data of product **43** was in good agreement with the literature[20].
^{1}H NMR (500 MHz, CDCl$_3$): δ 7.31 (d, *J* = 8.1 Hz, 2H), 7.18 (d, *J* = 8.1 Hz, 2H), 2.99–2.92 (m, 2H), 2.08–2.02 (m, 2H), 1.83–1.76 (m, 2H), 1.71–1.64 (m, 2H), 1.62–1.54 (m, 2H), 1.31 (s, 9H).
^{13}C NMR (126 MHz, CDCl$_3$): δ 148.4, 143.4, 126.7, 125.1, 45.5, 34.6, 34.3, 31.4, 25.5.

1-(*tert*-Butyl)-4-cyclobutylbenzene (44)

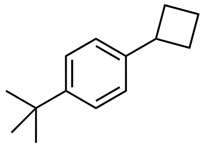

The general procedure was applied to 1-(*tert*-butyl)-4-chlorobenzene (**8’**, 84.9 mg, 0.50 mmol), sodium dispersion (26.1 wt%, 95.0 mg, 1.08 mmol), Fe(acac)$_3$ (5.5 mg, 16 μmol), and bromocyclobutane (28 μL, 0.30 mmol). The crude product was purified by column chromatography on silica gel (pentane only) to afford the title compound as a colorless oil (48.1 mg, 0.26 mmol, 86%). The spectral data of product **44** was in good agreement with the literature[20].
^{1}H NMR (500 MHz, CDCl$_3$): δ 7.32 (d, *J* = 8.0 Hz, 2H), 7.16 (d, *J* = 8.6 Hz, 2H), 3.51 (tt, *J* = 8.9, 8.9 Hz, 1H), 2.34–2.28 (m, 2H), 2.18–2.10 (m, 2H), 2.04–1.94 (m, 1H), 1.87–1.80 (m, 1H), 1.31 (s, 9H).
^{13}C NMR (126 MHz, CDCl$_3$): δ 148.4, 143.2, 126.0, 125.1, 40.0, 34.3, 31.4, 29.9, 18.3.

4-(4-(*tert*-Butyl)phenyl)tetrahydro-2*H*-pyran (45)

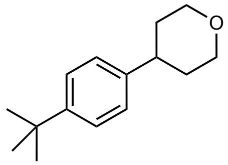

The general procedure was applied to 1-(*tert*-butyl)-4-chlorobenzene (**8’**, 84.6 mg, 0.50 mmol), sodium dispersion (26.1 wt%, 92.8 mg, 1.05 mmol), Fe(acac)$_3$ (5.3 mg, 15 μmol), and 4-bromotetrahydro-2*H*-pyran (33 μL, 0.30 mmol). The crude product was purified by column chromatography on silica gel (hexane/EtOAc = 20:1) to afford the title compound as a colorless oil (33.4 mg, 0.15 mmol, 52%). The spectral data of product **45** was in good agreement with the literature[20].

^{1}H NMR (500 MHz, $CDCl_3$): δ 7.34 (d, *J* = 8.1 Hz, 2H), 7.16 (d, *J* = 8.1 Hz, 2H), 4.09–4.06 (m, 2H), 3.52 (td, *J* = 11.6, 2.7 Hz, 2H), 2.73 (tt, *J* = 11.5, 4.3 Hz, 1H), 1.86–1.74 (m, 4H), 1.31 (s, 9H).
^{13}C NMR (126 MHz, $CDCl_3$): δ 149.0, 142.8, 126.3, 125.3, 68.4, 41.0, 34.3, 33.9, 31.4.

4-(4-(*tert*-Butyl)phenyl)-1-methylpiperidine (46)

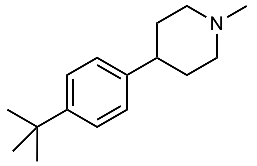

The general procedure was applied to 1-(*tert*-butyl)-4-chlorobenzene (**8'**, 84.6 mg, 0.50 mmol), sodium dispersion (26.1 wt%, 93.2 mg, 1.06 mmol), $Fe(acac)_3$ (5.4 mg, 15 µmol), and 4-bromo-1-methylpiperidine (41 µL, 0.30 mmol). The crude product was purified by column chromatography on silica gel (CH_2Cl_2/MeOH = 20:1) to afford the title compound **46** as a colorless solid (48.7 mg, 0.21 mmol, 70%).
Melting point: 68–70 °C.
^{1}H NMR (500 MHz, $CDCl_3$): δ 7.32 (d, *J* = 8.0 Hz, 2H), 7.16 (d, *J* = 8.1 Hz, 2H), 2.97 (d, *J* = 12.1 Hz, 2H), 2.45 (tt, *J* = 10.9, 5.4 Hz, 1H), 2.32 (s, 3H), 2.04 (td, *J* = 11.3, 3.7 Hz, 2H), 1.84–1.76 (m, 4H), 1.31 (s, 9H).
^{13}C NMR (126 MHz, $CDCl_3$): δ 148.8, 143.2, 126.4, 125.2, 56.4, 46.5, 41.4, 34.3, 33.5, 31.3.
HRMS (APCI+): *m/z* Calcd. for $C_{16}H_{25}N$ $[M+H]^+$: 232.2060; found: 232.2060.

1-(*tert*-Butyl)-4-(4,4-difluorocycloheyl)benzene (47)

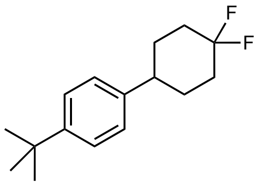

The general procedure was applied to 1-(*tert*-butyl)-4-chlorobenzene (**8'**, 84.4 mg, 0.50 mmol), sodium dispersion (26.1 wt%, 93.0 mg, 1.06 mmol), $Fe(acac)_3$ (5.4 mg, 15 µmol), and 4-bromo-1,1-difluorocyclohexane (39 µL, 0.30 mmol). The crude product was purified by column chromatography on silica gel (hexane/EtOAc = 20:1) to afford the title compound **47** as colorless solid (43.8 mg, 0.17 mmol, 58%).
Melting point: 63–65 °C.
^{1}H NMR (500 MHz, $CDCl_3$): δ 7.33 (d, *J* = 8.6 Hz, 2H), 7.15 (d, *J* = 8.0 Hz, 2H), 2.60–2.55 (m, 1H), 2.22–2.14 (m, 2H), 1.94–1.75 (m, 6H), 1.31 (s, 9H).
^{13}C NMR (126 MHz, $CDCl_3$): δ 149.2, 142.1, 126.4, 125.4, 123.3 (dd, J_{CF} = 240, 240 Hz), 41.9, 34.4, 34.1 (dd, J_{CF} = 26, 22 Hz), 31.4, 30.3 (d, J_{CF} = 10 Hz).
^{19}F NMR (470 MHz, $CDCl_3$): δ –94.7 (d, *J* = 234 Hz, 1F), –105.5 (dt, *J* = 234 Hz, J_{FH} = 29 Hz, 1F).

HRMS (APCI+): *m*/*z* Calcd. for $C_{16}H_{22}F_2$ $[M]^+$: 252.1684; found: 252.1681.

1-(*sec*-Butyl)-4-(*tert*-butyl)benzene (48)

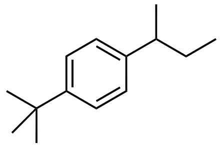

The general procedure (step 2, $Fe(acac)_3$ (10 mol%) was used) was applied to 1-(*tert*-butyl)-4-chlorobenzene (**8'**, 84.8 mg, 0.50 mmol), sodium dispersion (26.1 wt%, 93.4 mg, 1.06 mmol), $Fe(acac)_3$ (10.6 mg, 30 μmol), and 2-bromobutane (33 μL, 0.30 mmol). The crude product was purified by column chromatography on silica gel (pentane only) to afford the title compound as a colorless oil (32.0 mg, 0.17 mmol, 55%). The spectral data of product **48** was in good agreement with the literature[25].

^{1}H NMR (500 MHz, $CDCl_3$): δ 7.30 (d, J = 8.0 Hz, 2H), 7.11 (d, J = 8.0 Hz, 2H), 2.56 (qt, J = 7.1, 7.1 Hz, 1H), 1.64–1.52 (m, 2H), 1.31 (s, 9H), 1.22 (d, J = 6.9 Hz, 3H), 0.83 (t, J = 7.4 Hz, 3H).

^{13}C NMR (126 MHz, $CDCl_3$): δ 148.4, 144.6, 126.6, 125.0, 41.1, 34.3, 31.4, 31.2, 21.7, 12.3.

1-(*tert*-Butyl)-4-isobutylbenzene (49)

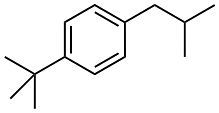

The general procedure was applied to 1-(*tert*-butyl)-4-chlorobenzene (**8'**, 84.6 mg, 0.50 mmol), sodium dispersion (26.1 wt%, 93.0 mg, 1.06 mmol), $Fe(acac)_3$ (5.5 mg, 16 μmol), and 1-bromo-2-methylpropane (32 μL, 0.30 mmol). The crude product was purified by column chromatography on silica gel (pentane only) to afford the title compound as a colorless oil (50.6 mg, 0.27 mmol, 90%). The spectral data of product **49** was in good agreement with the literature[24].

^{1}H NMR (500 MHz, $CDCl_3$): δ 7.28 (d, J = 8.1 Hz, 2H), 7.07 (d, J = 8.0 Hz, 2H), 2.44 (d, J = 7.5 Hz, 2H), 1.85 (sept-t, J = 6.7, 6.7 Hz, 1H), 1.31 (s, 9H), 0.90 (d, J = 6.3 Hz, 6H).

^{13}C NMR (126 MHz, $CDCl_3$): δ 148.3, 138.6, 128.7, 124.9, 44.9, 34.3, 31.4, 30.2, 22.5.

- Control experiment in the absence of $Fe(acac)_3$ catalyst -

The general procedure was applied to 1-(*tert*-butyl)-4-chlorobenzene (**8'**, 84.8 mg, 0.50 mmol), sodium dispersion (26.1 wt%, 93.1 mg, 1.06 mmol), and 1-bromo-2-methylpropane (32 μL, 0.30 mmol). The yield of the product was estimated by GC analysis with dodecane as an internal standard to be 2%.

1-(*tert*-Butyl)-4-(2-ethylhexyl)benzene (50)

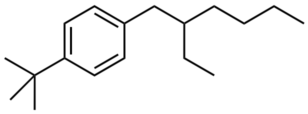

The general procedure was applied to 1-(*tert*-butyl)-4-chlorobenzene (**8'**, 84.8 mg, 0.50 mmol), sodium dispersion (26.1 wt%, 93.8 mg, 1.06 mmol), $Fe(acac)_3$ (5.4 mg, 15 μmol), and 3-(bromomethyl)heptane (52 μL, 0.30 mmol). The crude product was purified by column chromatography on silica gel (hexane only) to afford the title compound **50** as a colorless oil (58.1 mg, 0.24 mmol, 78%).

^{1}H NMR (500 MHz, $CDCl_3$): δ 7.27 (d, *J* = 8.6 Hz, 2H), 7.07 (d, *J* = 8.1 Hz, 2H), 2.53–2.44 (m, 2H), 1.59–1.51 (m, 1H), 1.34–1.23 (m, 8H), 1.31 (s, 9H), 0.89–0.85 (m, 6H).

^{13}C NMR (126 MHz, $CDCl_3$): δ 148.2, 138.7, 128.8, 124.9, 41.0, 39.5, 34.3, 32.4, 31.4, 28.9, 25.3, 23.1, 14.2, 10.7.

HRMS (APCI+): *m*/*z* Calcd. for $C_{18}H_{30}$ $[M]^+$: 246.2342; found: 246.2348.

- Control experiment in the absence of the $Fe(acac)_3$ catalyst -

The general procedure was applied to 1-(*tert*-butyl)-4-chlorobenzene (**8'**, 84.8 mg, 0.50 mmol), sodium dispersion (26.1 wt%, 93.4 mg, 1.06 mmol), and 3-(bromomethyl)heptane (52 μL, 0.30 mmol). The yield of the product was estimated by GC analysis with dodecane as an internal standard to be 3%.

1-(*tert*-Butyl)-4-(cyclohexylmethyl)benzene (51)

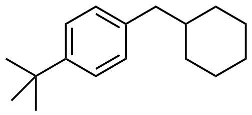

The general procedure was applied to 1-(*tert*-butyl)-4-chlorobenzene (**8'**, 84.6 mg, 0.50 mmol), sodium dispersion (26.1 wt%, 93.7 mg, 1.06 mmol), $Fe(acac)_3$ (5.2 mg, 14 μmol), and (bromomethyl)cyclohexane (42 μL, 0.30 mmol). The crude product was purified by column chromatography on silica gel (hexane only) to afford the title compound **51** as a colorless oil (49.2 mg, 0.21 mmol, 71%). The spectral data of product **51** was in good agreement with the literature[26].

^{1}H NMR (500 MHz, $CDCl_3$): δ 7.28 (d, *J* = 8.0 Hz, 2H), 7.06 (d, *J* = 8.0 Hz, 2H), 2.44 (d, *J* = 7.7 Hz, 2H), 1.69–1.62 (m, 5H), 1.54–1.46 (m, 1H), 1.31 (s, 9H), 1.23–1.10 (m, 3H), 0.96–0.87 (m, 2H).

^{13}C NMR (126 MHz, $CDCl_3$): δ 148.2, 138.2, 128.8, 124.9, 43.6, 39.7, 34.3, 33.2, 31.4, 26.6, 26.3.

1-(*tert*-Butyl)-4-(cyclopentylmethyl)benzene (52)

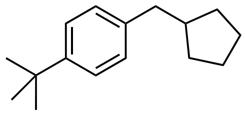

The general procedure was applied to 1-(*tert*-butyl)-4-chlorobenzene (**8'**, 84.7 mg, 0.50 mmol), sodium dispersion (26.1 wt%, 93.3 mg, 1.06 mmol), $Fe(acac)_3$ (5.4 mg, 15 μmol), and (bromomethyl)cyclopentane (37 μL, 0.30 mmol). The crude product was purified by column chromatography on silica gel (hexane only) to afford the title compound **52** as a colorless oil (51.8 mg, 0.24 mmol, 79%).

^{1}H NMR (500 MHz, $CDCl_3$): δ 7.28 (d, *J* = 8.6 Hz, 2H), 7.10 (d, *J* = 8.0 Hz, 2H), 2.57 (d, *J* = 7.5 Hz, 2H), 2.07 (tdd, *J* = 7.6, 7.6, 7.6 Hz, 1H), 1.73–1.67 (m, 2H), 1.67–1.59 (m, 2H), 1.55–1.46 (m, 2H), 1.31 (s, 9H), 1.23–1.15 (m, 2H).

^{13}C NMR (126 MHz, $CDCl_3$): δ 148.2, 139.3, 128.4, 125.0, 42.0, 41.6, 34.3, 32.6, 31.4, 24.9.

HRMS (APCI+): *m*/*z* Calcd. for $C_{16}H_{24}$ $[M]^+$: 216.1873; found: 216.1875.

1-(*tert*-Butyl)-4-(cyclobutylmethyl)benzene (53)

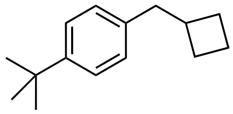

The general procedure was applied to 1-(*tert*-butyl)-4-chlorobenzene (**8'**, 84.5 mg, 0.50 mmol), sodium dispersion (26.1 wt%, 93.6 mg, 1.06 mmol), $Fe(acac)_3$ (5.4 mg, 15 μmol), and (bromomethyl)cyclobutane (33 μL, 0.30 mmol). The crude product was purified by column chromatography on silica gel (hexane only) to afford the title compound **53** as a colorless oil (50.1 mg, 25 mmol, 82%).

^{1}H NMR (500 MHz, $CDCl_3$): δ 7.28 (d, *J* = 8.6 Hz, 2H), 7.07 (d, *J* = 8.6 Hz, 2H), 2.67 (d, *J* = 7.5 Hz, 2H), 2.55 (tdd, *J* = 7.7, 7.7, 7.7 Hz, 1H), 2.07–2.00 (m, 2H), 1.87–1.79 (m, 2H), 1.75–1.68 (m, 2H), 1.30 (s, 9H).

^{13}C NMR (126 MHz, $CDCl_3$): δ 148.3, 138.3, 128.1, 125.0, 42.5, 37.2, 34.3, 31.4, 28.3, 18.4.

HRMS (APCI+): *m*/*z* Calcd. for $C_{15}H_{22}$ $[M]^+$: 202.1716; found: 202.1717.

(3*S*,8*S*,9*S*,10*R*,13*R*,14*S*,17*R*)-3-(4-(*tert*-Butyl)phenyl)-10,13-dimethyl-17-((*R*)-6-methylheptan-2-yl)-2,3,4,7,8,9,10,11,12,13,14,15,16,17-tetradecahydro-1*H*-cyclopenta[*a*]phenanthrene (55)

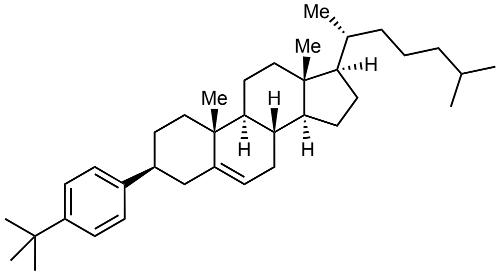

The general procedure was applied to 1-(*tert*-butyl)-4-chlorobenzene (**8'**, 84.4 mg, 0.50 mmol), sodium dispersion (26.1 wt%, 93.1 mg, 1.06 mmol), $Fe(acac)_3$ (5.1 mg, 14 μmol), and (3*S*,8*S*,9*S*,10*R*,13*R*,14*S*,17*R*)-3-bromo-10,13-dimethyl-17-((*R*)-6-methylheptan-2-yl)-2,3,4,7,8,9,10,11,12,13,14,15,16,17-tetradecahydro-1*H*-cyclopenta[*a*]phenanthrene (**54**, 135.3 mg, 0.30 mmol, from Tokyo Chemical Industry Co., Ltd. [code: C0695]). The crude product was purified by column chromatography on silica gel (hexane only) and GPC (chloroform) to afford the title compound **55** as a white solid (85.0 mg, 0.17 mmol, 56%).
Melting point: 177–180 °C.
^{1}H NMR (500 MHz, $CDCl_3$): δ 7.32 (d, *J* = 8.6 Hz, 2H), 7.17 (d, *J* = 8.6 Hz, 2H), 5.35–5.34 (m, 1H), 2.53–2.47 (m, 1H), 2.45–2.39 (m, 1H), 2.21–2.17 (m, 1H), 2.05–1.94 (m, 3H), 1.88–1.69 (m, 3H), 1.62–0.99 (m, 20H), 1.31 (s, 9H), 1.08 (s, 3H), 0.93 (d, *J* = 6.3 Hz, 3H), 0.87 (dd, *J* = 6.9, 2.3 Hz, 6H), 0.70 (s, 3H).
^{13}C NMR (126 MHz, $CDCl_3$): δ 148.6, 144.0, 143.1, 126.3, 125.2, 120.0, 56.9, 56.2, 50.5, 45.2, 42.3, 40.7, 39.93, 39.87, 39.5, 36.9, 36.2, 35.8, 34.3, 32.0, 31.9, 31.4, 30.0, 28.3, 28.0, 24.3, 23.9, 22.9, 22.6, 21.0, 19.6, 18.8, 11.9.
HRMS (APCI+): *m*/*z* Calcd. for $C_{37}H_{58}$ $[M]^+$: 502.4533; found: 502.4527.

7. Mechanistic investigation

General procedure for radical clock experiments

Step 1: preparation of organosodium compound $\mathbf{8_{Na}}$.

In a dry Schlenk tube equipped with a glass-coated stirring bar, 1-(*tert*-butyl)-4-chlorobenzene (**8'**, 84.3 mg, 0.50 mmol, 1.67 equiv.) was added to a mixture of MCH (1.0 mL) and sodium dispersion (ca. 26 wt%, ca. 92.8 mg, 1.05 mmol, 350 mol% to alkyl bromide probes; 210 mol% to 1-(*tert*-butyl)-4-chlorobenzene) under nitrogen. After stirring at 30 °C for 1 h, the corresponding arylsodium $\mathbf{8_{Na}}$ was generated.

Step 2: iron-catalysed cross coupling of organosodium compound $\mathbf{8_{Na}}$ with alkyl bromide probes. $Fe(acac)_3$ (5.3 mg, 15 μmol, 5 mol%), TMEDA (0.50 mL), and the alkyl bromide radical probes (0.30 mmol, 1.0 equiv.) were added to the suspension of arylsodium in MCH (1.0 mL) at 0 °C, and the reaction mixture was stirred at the same temperature for 1 h. The reaction was quenched with D_2O (0.1 mL) (to estimate the amount of active arylsodium remaining in the reaction mixture) and then with a saturated aqueous solution of NH_4Cl (1 mL) at 0 °C. The reaction mixture was diluted with ethyl acetate and passed through a plug of silica gel with ethyl acetate for GC and GC-MS analysis. The yields were estimated by GC analysis with dodecane as an internal standard.

- Reaction with (bromomethyl)cyclopropane

The general procedure was applied to 1-(*tert*-butyl)-4-chlorobenzene (**8'**, 84.9 mg, 0.50 mmol), sodium dispersion (25.9 wt%, 95.5 mg, 1.08 mmol), $Fe(acac)_3$ (5.4 mg, 15 μmol), and (bromomethyl)cyclopropane (29 μL, 0.30 mmol). The yields of products **56** and **57** were estimated by GC analysis with dodecane as an internal standard to be n.d. and 11%, respectively.

- Control experiment in the absence of $Fe(acac)_3$ catalyst -

The general procedure was applied to 1-(*tert*-butyl)-4-chlorobenzene (**8'**, 84.6 mg, 0.50 mmol), sodium dispersion (25.9 wt%, 93.9 mg, 1.06 mmol), and (bromomethyl)cyclopropane (29 μL, 0.30 mmol). The yields of the product **56** and **57** were estimated by GC analysis with dodecane as an internal standard to be n.d. and n.d., respectively.

Supplementary Table 4. Radical clock experiment with (bromomethyl)cyclopropane.

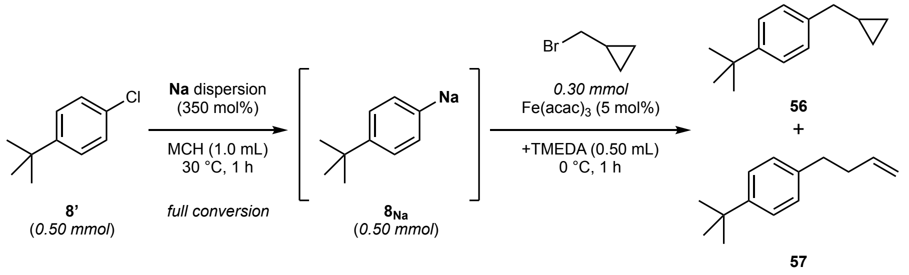

entry	$Fe(acac)_3$	**56** (%)	**57** (%)
1	w/	n.d.	11
2	w/o	n.d.	n.d.

GC yields using dodecane as an internal standard.

- Reaction with 6-bromohex-1-ene

The general procedure was applied to 1-(*tert*-butyl)-4-chlorobenzene (**8'**, 84.0 mg, 0.50 mmol), sodium dispersion (25.9 wt%, 94.8 mg, 1.07 mmol), $Fe(acac)_3$ (5.4 mg, 15 μmol), and 6-bromohex-1-ene (40 μL, 0.30 mmol). The yields of products **58** and **52** were estimated by GC analysis with dodecane as an internal standard to be 15% and 30%, respectively. The crude product was purified by column chromatography on silica gel (hexane only) to afford the compound **58** as a colorless oil (10.6 mg, 0.05 mmol, 16%) and the compound **52** as a colorless oil (17.5 mg, 0.08 mmol, 27%).

1-(*tert*-Butyl)-4-(hex-5-en-1-yl)benzene (58)

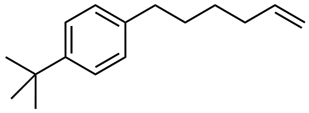

^{1}H NMR (500 MHz, $CDCl_3$): δ 7.30 (d, J = 8.6 Hz, 2H), 7.11 (d, J = 8.6 Hz, 2H), 5.81 (ddt, J = 17.0, 10.2, 6.9 Hz, 1H), 5.00 (dtd, J = 17.0, 1.7, 1.7 Hz, 1H), 4.93 (ddt, J = 10.2, 2.3, 1.2 Hz), 2.58 (t, J = 7.7 Hz, 2H), 2.11–2.06 (m, 2H), 1.66–1.60 (m, 2H), 1.47–1.41 (m, 2H), 1.31 (s, 9H).
^{13}C NMR (126 MHz, $CDCl_3$): δ 148.4, 139.6, 138.9, 128.0, 125.1, 114.4, 35.2, 34.3, 33.7, 31.4, 30.9, 28.7.
HRMS (APCI+): *m*/*z* Calcd. for $C_{16}H_{24}$ $[M]^+$: 216.1873; found: 216.1873.

1-(*tert*-Butyl)-4-(cyclopentylmethyl)benzene (52)

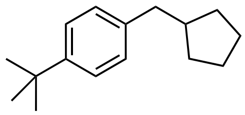

^{1}H NMR (500 MHz, $CDCl_3$): δ 7.28 (d, J = 8.6 Hz, 2H), 7.10 (d, J = 8.0 Hz, 2H), 2.57 (d, J = 7.5 Hz, 2H), 2.07 (tdd, J = 7.6, 7.6, 7.6 Hz, 1H), 1.73–1.67 (m, 2H), 1.67–1.59 (m, 2H), 1.55–1.46 (m, 2H), 1.31 (s, 9H), 1.23–1.15 (m, 2H).
^{13}C NMR (126 MHz, $CDCl_3$): δ 148.2, 139.3, 128.4, 125.0, 42.0, 41.6, 34.3, 32.6, 31.4, 24.9.
HRMS (APCI+): *m*/*z* Calcd. for $C_{16}H_{24}$ $[M]^+$: 216.1873; found: 216.1875.

- Control experiment in the absence of $Fe(acac)_3$ catalyst -

The general procedure was applied to 1-(*tert*-butyl)-4-chlorobenzene (**8'**, 84.7 mg, 0.50 mmol), sodium dispersion (25.9 wt%, 94.6 mg, 1.07 mmol), and 6-bromohex-1-ene (40 μL, 0.30 mmol). The yields of products **58** and **52** were estimated by GC analysis with dodecane as an internal standard to be 4% and n.d., respectively.

Supplementary Table 5. Radical clock experiment with 6-bromohex-1-ene.

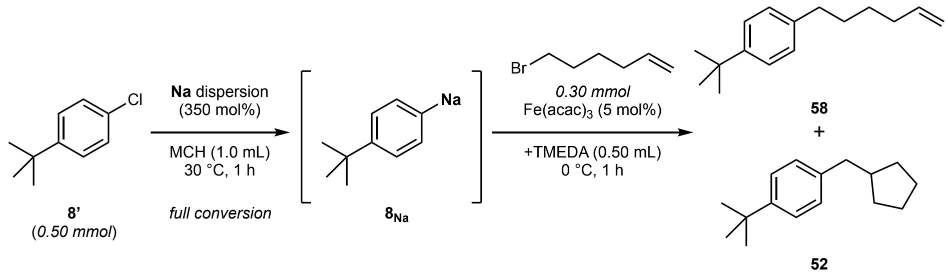

entry	$Fe(acac)_3$	**58** (%)	**52** (%)
1	w/	15 (16)	30 (27)
2	w/o	4	n.d.

GC yields using dodecane as an internal standard. Isolated yields are shown in parentheses.

8. Synthesis and characterisation of organometallic complexes

Synthesis of [($C_6H_4OMeNa)_4(TMEDA)_2$]

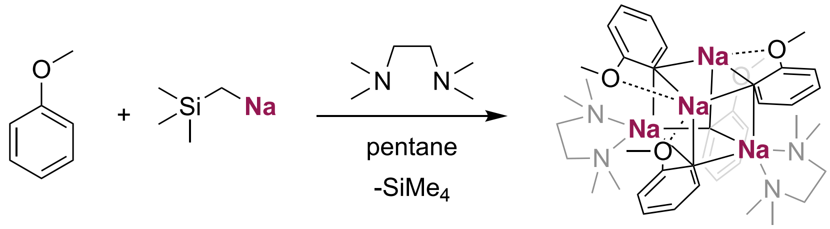

In a glovebox, $NaCH_2SiMe_3$ (2 mmol, 220 mg) was suspended in pentane (3 mL), and TMEDA (4 mmol, 600 μL) and anisole (2 mmol, 224 μL) were added at room temperature to give a light-yellow solution. It was stirred for 1 hour and then placed in a freezer at –30 ºC for 16 hours, to form a yellow crystalline solid. Removal of the supernatant and washing with cold pentane (x2) afforded the pure compound as a light-yellow solid (200.2 mg, 53% yield).

^{1}H NMR (300 MHz, C_6D_6): δ 8.11 (dd, J = 6.1, 1.9 Hz, 1H), 7.29 (ddd, J = 8.0, 7.1, 1.9 Hz, 1H), 7.22–7.13 (m, 2H), 6.67 (d, J = 7.6 Hz, 1H), 3.43 (s, 3H), 1.78 (s, 2H), 1.65 (s, 6H).

^{13}C NMR (75 MHz, C_6D_6): δ 169.6, 142.7, 126.0, 121.7, 106.2, 57.8, 54.2, 45.6. *Note: The signal corresponding to the quaternary sodiated carbon cannot be detected in the ^{13}C NMR spectrum or in the HMBC experiments despite several attempts.

Elemental analysis: calculated for $C_{40}H_{60}N_4Na_4O_4$: C, 63.81; H, 8.03; N, 7.44, found: C, 63.84; H, 8.03; N, 7.41.

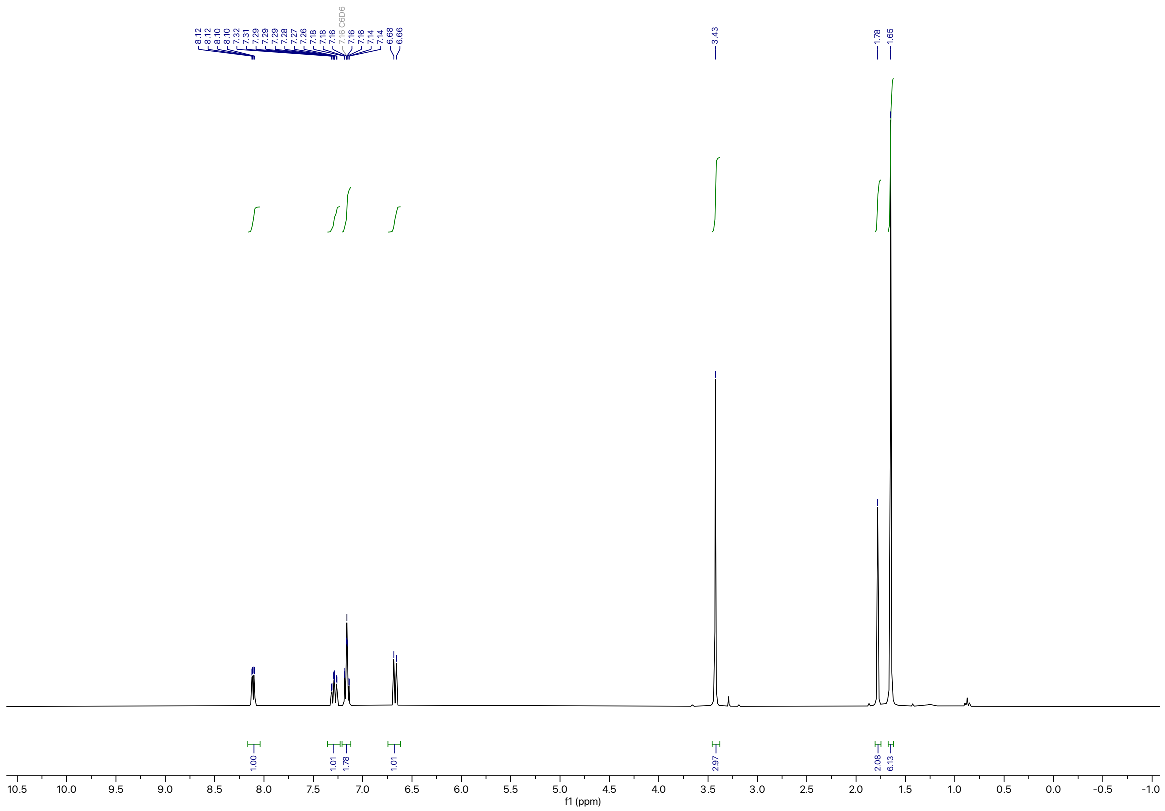

Supplementary Figure 2. ^{1}H NMR spectrum of [($C_6H_4OMeNa)_4(TMEDA)_2$] in C_6D_6.

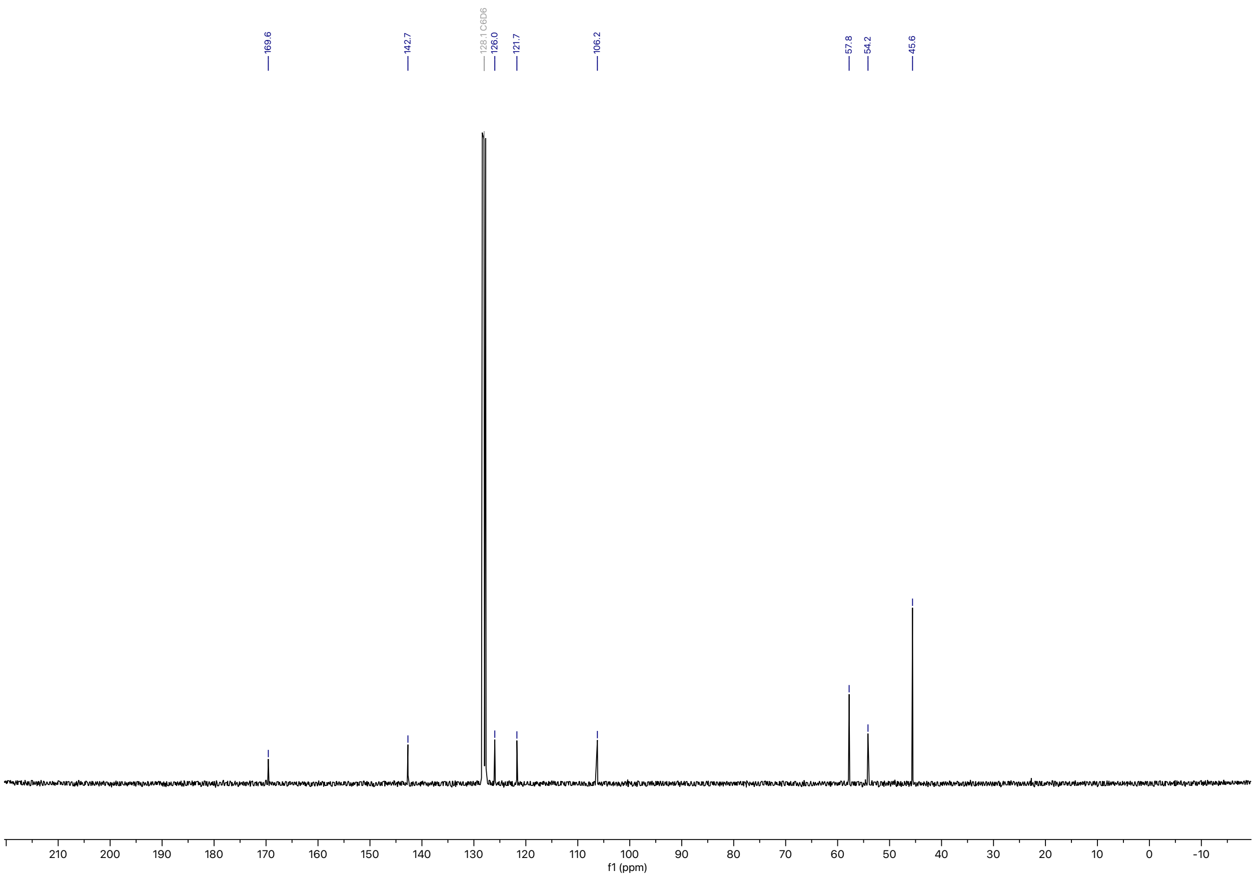

Supplementary Figure 3. ^{13}C NMR spectrum of $[(C_6H_4OMeNa)_4(TMEDA)_2]$ in C_6D_6.

Synthesis of $[(TMEDA)FeBr(C_6H_4OMe)]$ (Fe-1)

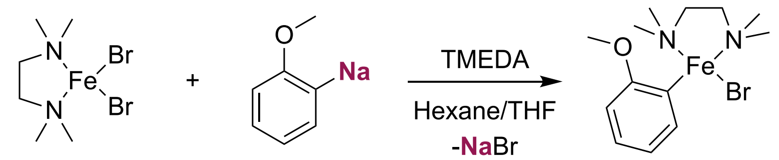

In a glove box, $FeBr_2$ (1 mmol, 215.6 mg) was suspended in THF (3 mL) and TMEDA (1 mmol, 150 µL) was added to form a brown suspension. In a separate vial, anisole (1 mmol, 112 µL), $NaCH_2SiMe_3$ (1 mmol, 110 mg), and TMEDA (1 mmol,150 µL) were mixed in hexane (3 mL) and stirred for 1 hour. The second solution (metalated anisole) was added dropwise at room temperature into the $(TMEDA)FeBr_2$ solution. A white solid formed, which was filtered using a Pasteur pipette and a glass filter pad to give a light brown solution. It was stored at –30 ºC for 3 days to give a light brown crystalline solid. Removal of the supernatant, washing with cold pentane and filtration delivered $[(TMEDA)FeBr(C_6H_4OMe)]$ (**Fe-1**) as a light brown solid (98.0 mg, 27% yield). It was stored at –30 ºC in the glovebox freezer, due to fast decomposition at room temperature.

The solution magnetic moment was calculated by the Evans' Method in C_6D_6 (and using a capillary of the same solvent) to obtain a value of 5.25 µB, which agrees with being a high spin Fe(II) centre.

Elemental analysis was attempted, but due to the high reactivity of the complex, unsatisfactory results were obtained despite multiple attempts. The best results were: calculated for $C_{13}H_{23}BrFeN_2O$: C, 43.48; H, 7.15; N, 7.80, found: C, 37.76; H, 6.46; N, 7.77.

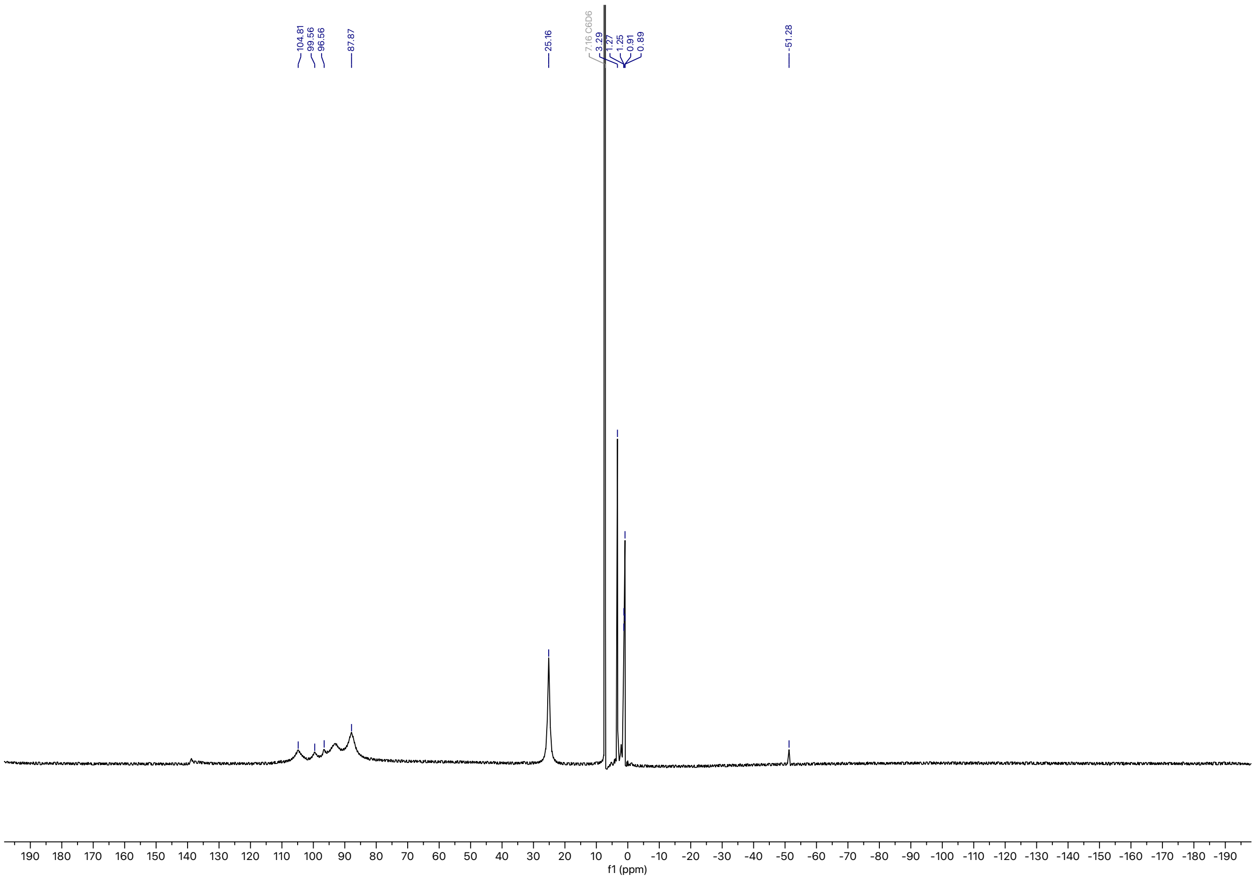

Supplementary Figure 4. ^{1}H NMR spectrum of [(TMEDA)FeBr(C_6H_4OMe)] **(Fe-1)** in C_6D_6.

Synthesis of [(TMEDA)Fe(C_6H_4OMe)$_2$] (Fe-2)

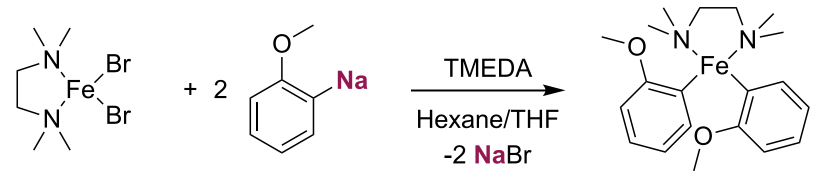

In a glove box, $FeBr_2$ (1 mmol, 215.6 mg) was suspended in THF (3 mL) and TMEDA (1 mmol, 150 µL) was added to form a brown suspension. In a separate vial, anisole (2 mmol, 224 µL), $NaCH_2SiMe_3$ (2 mmol, 220 mg), and TMEDA (2 mmol, 300 µL) were mixed in hexane (3 mL) and stirred for 1 hour. The second solution (metalated anisole) was added dropwise at room temperature into the (TMEDA)$FeBr_2$ solution. A white solid formed, which was filtered using a Pasteur pipette and a glass filter pad to give a light brown solution. It was stored at –30 ºC for 3 days to give a light brown crystalline solid. Removal of the supernatant, washing with cold pentane and filtration delivered [(TMEDA)Fe(C_6H_4OMe)$_2$] (**Fe-2**) as a pale yellow solid (26.0 mg, 7%

yield). It was stored at –30 ºC in the glovebox freezer, due to fast decomposition at room temperature.

The solution magnetic moment was calculated by the Evans' Method in C_6D_6 (and using a capillary of the same solvent) to obtain a value of 5.01 μB, which agrees with being a high spin Fe(II) centre. Elemental analysis was attempted, but due to the high reactivity of the complex, unsatisfactory results were obtained despite multiple attempts. The best results were: calculated for $C_{20}H_{30}FeN_2O_2$: C, 62.18; H, 7.83; N, 7.25, found: C, 63.67; H, 7.53; N, 5.69.

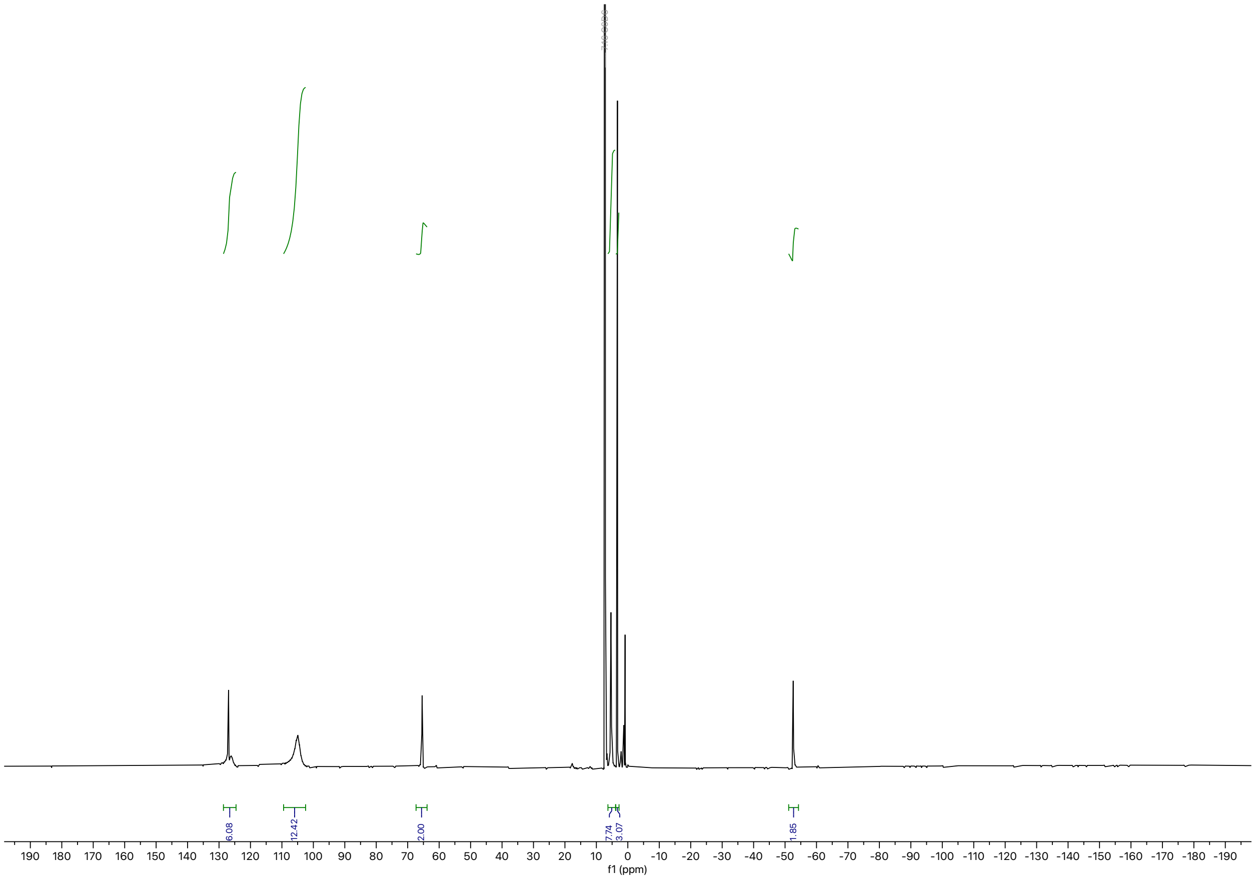

Supplementary Figure 5. ^{1}H NMR spectrum of [(TMEDA)Fe(C_6H_4OMe)$_2$] **(Fe-2)** in C_6D_6.

Reaction of Fe(acac)$_3$ with 12$_{Na}$.

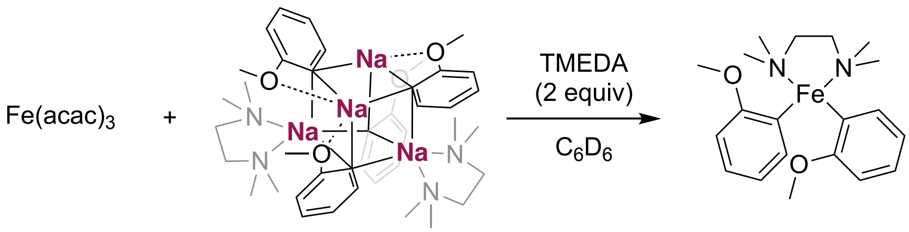

In a glovebox, Fe(acac)$_3$ (3.5 mg, 0.01 mmol) was added into an NMR tube. TMEDA (3 µL, 0.02 mmol) and C_6D_6 were added. [(C_6H_4OMeNa)$_4$(TMEDA)$_2$] (7.6 mg, 0.01 mmol) were added to the tube, and mixed by inversion multiple times. After 10 minutes, a ^{1}H NMR spectrum was recorded, showing the formation of **Fe-2** along with other unidentified diamagnetic species.

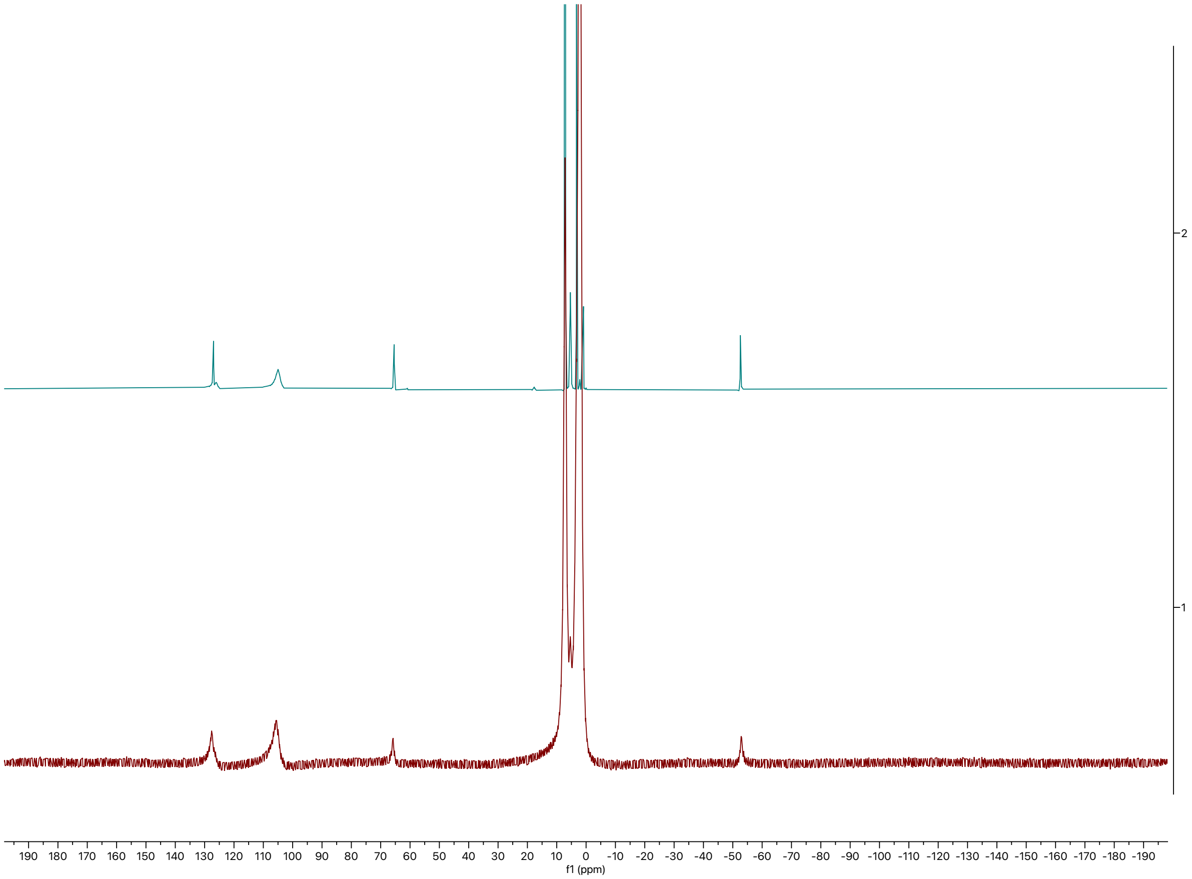

Supplementary Figure 6. Top: ^{1}H NMR spectrum of [(TMEDA)Fe(C_6H_4OMe)$_2$] (**Fe-2**) in C_6D_6; bottom: in situ formation of **Fe-2** with Fe(acac)$_3$ and **12$_{Na}$**.

Reaction of Fe(acac)$_3$ with [(C_6H_4OMeNa)$_4$(TMEDA)$_2$].

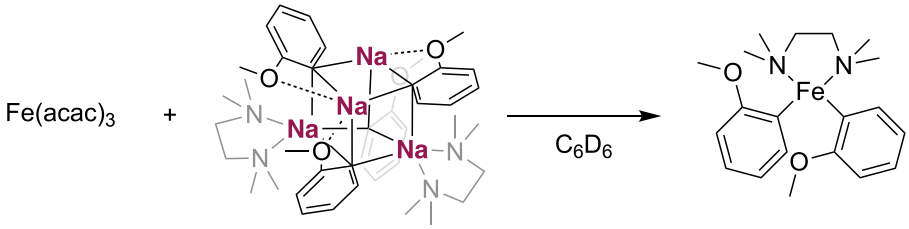

In a glovebox, Fe(acac)$_3$ (3.5 mg, 0.01 mmol) was added into an NMR tube and dissolved in C_6D_6. [(C_6H_4OMeNa)$_4$(TMEDA)$_2$] (7.6 mg, 0.01 mmol) were added to the tube, and mixed by inversion multiple times. After 10 minutes, the solution was filtered and transferred to a new NMR tube, where a ^{1}H NMR spectrum was recorded, showing the formation of **Fe-2** along with other unidentified diamagnetic species.

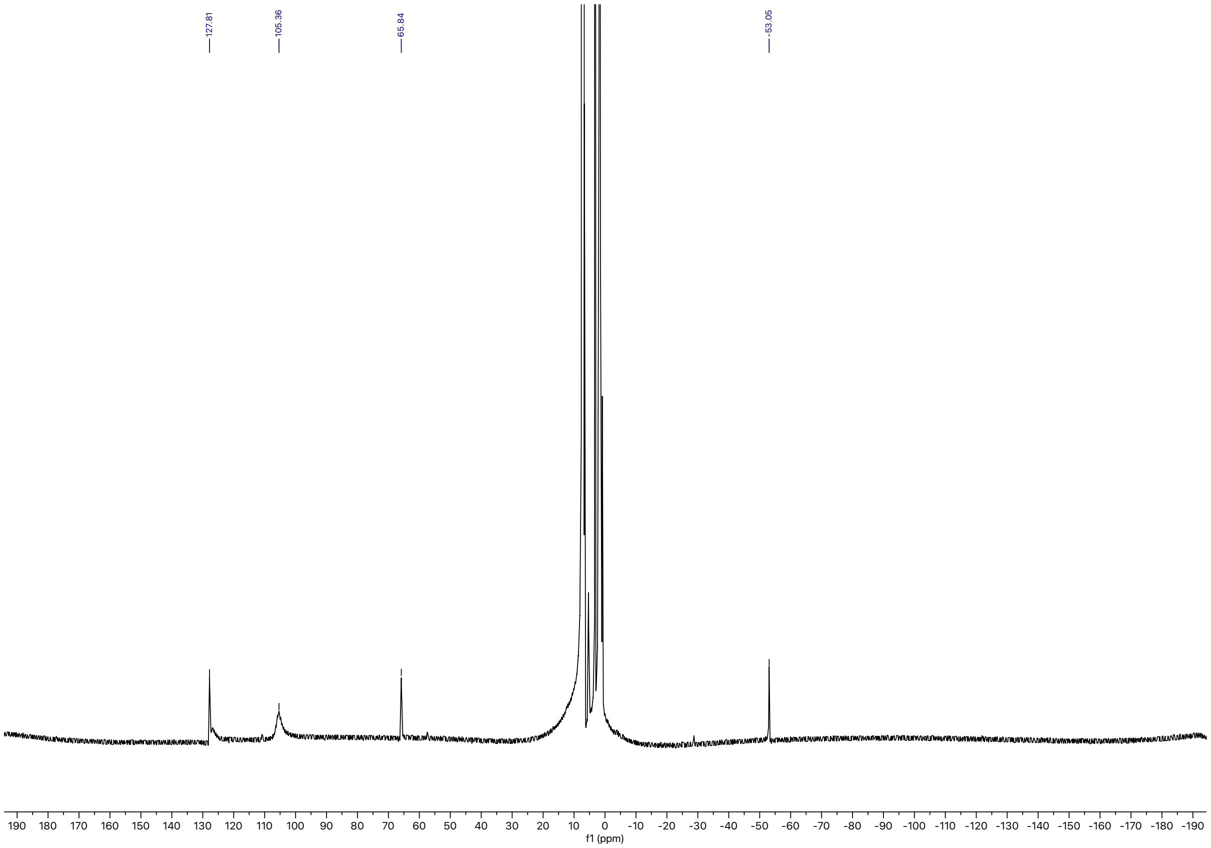

Supplementary Figure 7. In situ formation of **Fe-2** with Fe(acac)$_3$ and [(C_6H_4OMeNa)$_4$(TMEDA)$_2$].

EPR experiment with frozen reaction mixture.

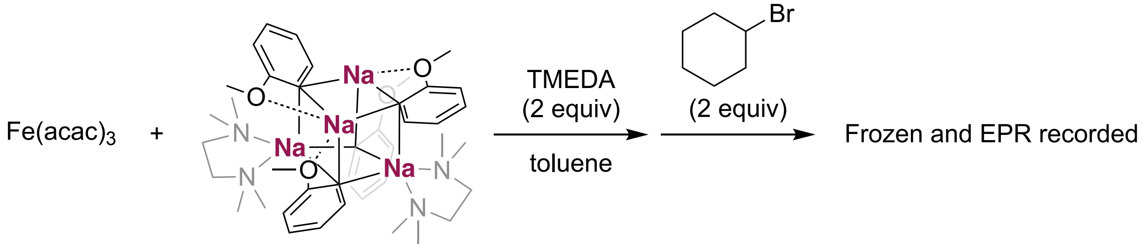

In a glovebox, $Fe(acac)_3$ (3.5 mg, 0.01 mmol) was added to a vial and dissolved in toluene. TMEDA (3 µL, 0.02 mmol) and $[(C_6H_4OMeNa)_4(TMEDA)_2]$ (7.6 mg, 0.01 mmol) were added and reacted for 10 minutes. After this amount of time, bromocyclohexane (2.6 µL, 0.02 mmol) was added, reacted for 1 minute and filtered through a glass filter. The solution was added to an EPR tube, frozen in liquid nitrogen and measured immediately. No EPR signals were detected, indicating that no EPR active species were formed.

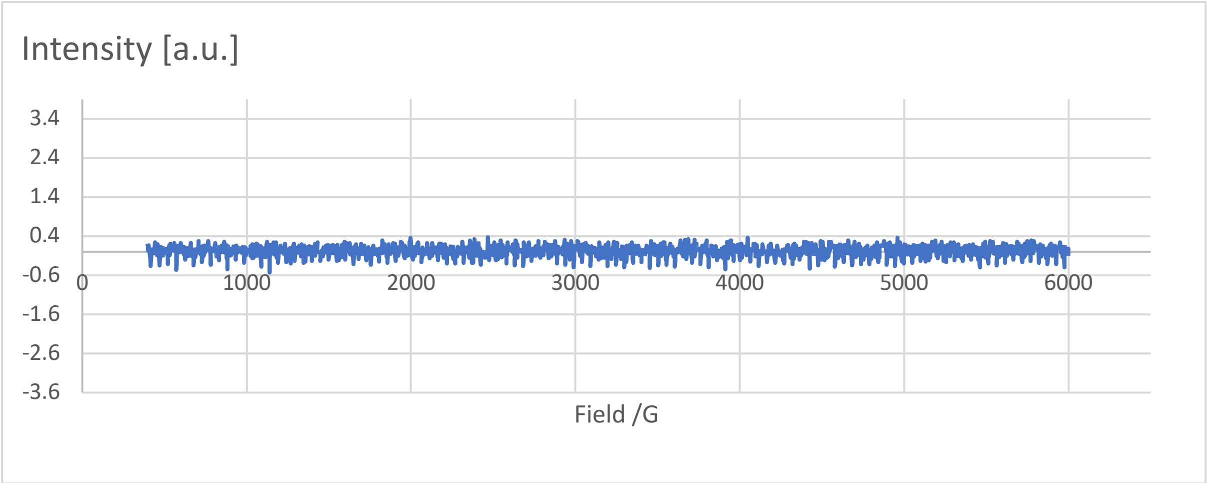

Supplementary Figure 8. EPR spectrum of frozen reaction mixture.

9. ^{1}H DOSY NMR studies

Diffusion Ordered Spectroscopy (DOSY) experiment was conducted by NMR using the External Calibration Curve (ECC) method at 15 mM in C_6D_{12}[27]. Data was accumulated by linearly varying the diffusion encoding gradients over a range of 2% to 95% for 32 gradient values. The signal decay dimension on the pseudo-2D data was generated by Fourier transformation of the time-domain data. The diffusion profile and coefficients were ascertained by use of the DOSY processing features of TopSpin software. The peak of residual proteosolvent was used as an internal standard.

^{1}H DOSY NMR of [(4-$C_6H_4{}^t$BuNa)TMEDA]

Using C_6D_{12} as an internal standard, we calculated the estimated molecular weight of the [(4-$C_6H_4{}^t$BuNa)TMEDA] aggregate to be 709 g mol^{-1}.

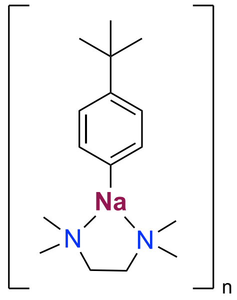

n = 1 : mw = 272 g mol^{-1}
n = 2 : mw = 545 g mol^{-1}
n = 3 : mw = 817 g mol^{-1}

Average diffusion coefficient = 3.541 x 10^{-10} m^2 s^{-1}

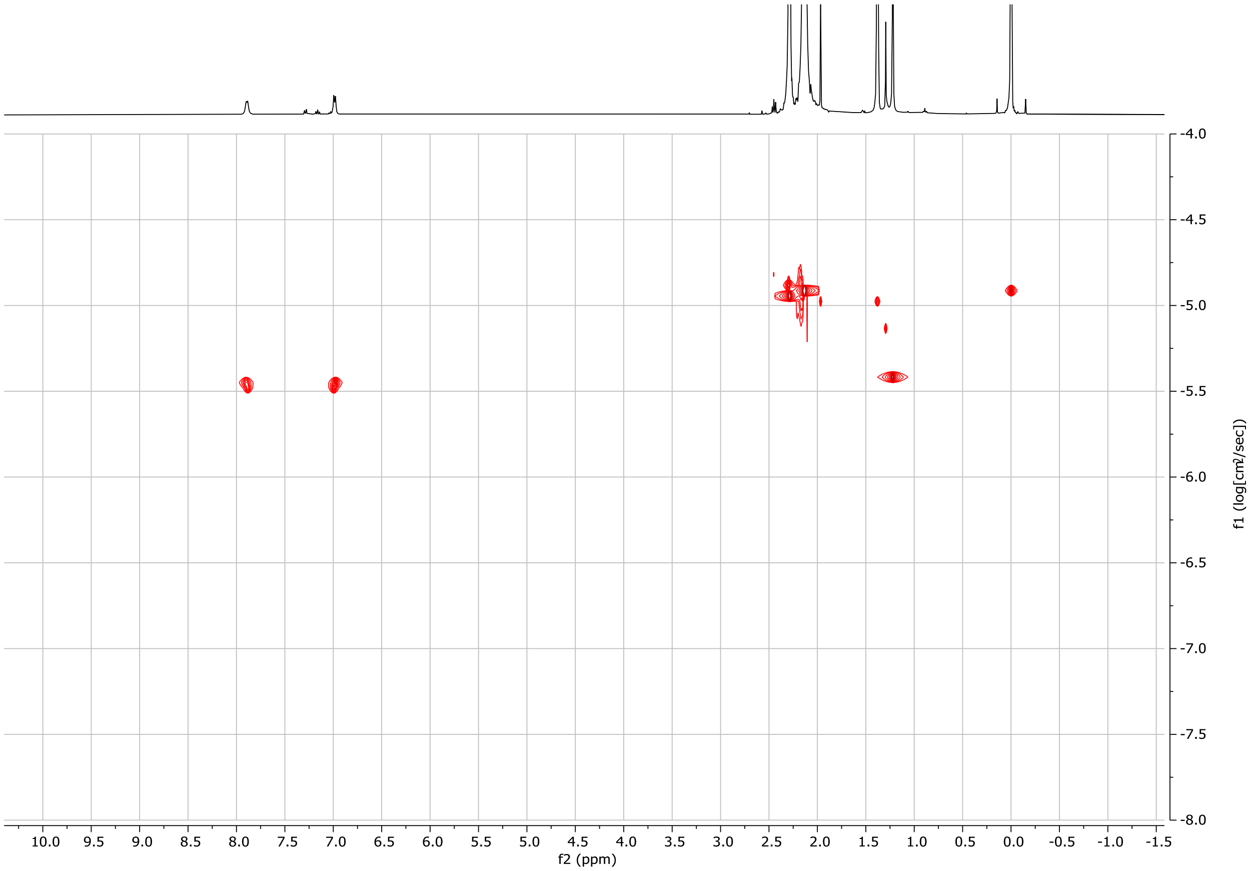

Supplementary Figure 9. ^{1}H DOSY NMR spectrum of [(4-$C_6H_4{}^t$BuNa)(TMEDA)] in C_6D_{12}.

^{1}H DOSY NMR of 1:1 (C_6H_4OMeNa):(TMEDA)

Using C_6D_{12} as an internal standard, we calculated the estimated molecular weight of the formed aggregate from a 1:1 mixture of C_6H_4OMeNa and TMEDA to be 816 g mol^{-1}.

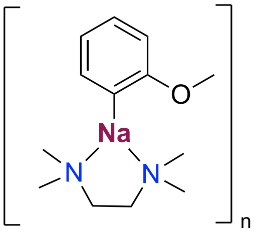

n = 1 : mw = 246 g mol^{-1}
n = 2 : mw = 493 g mol^{-1}
n = 3 : mw = 739 g mol^{-1}

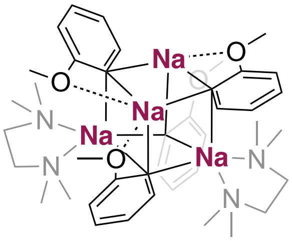

mw = 753 g mol^{-1}

Average diffusion coefficient = 3.360 x 10^{-10} m^2 s^{-1}

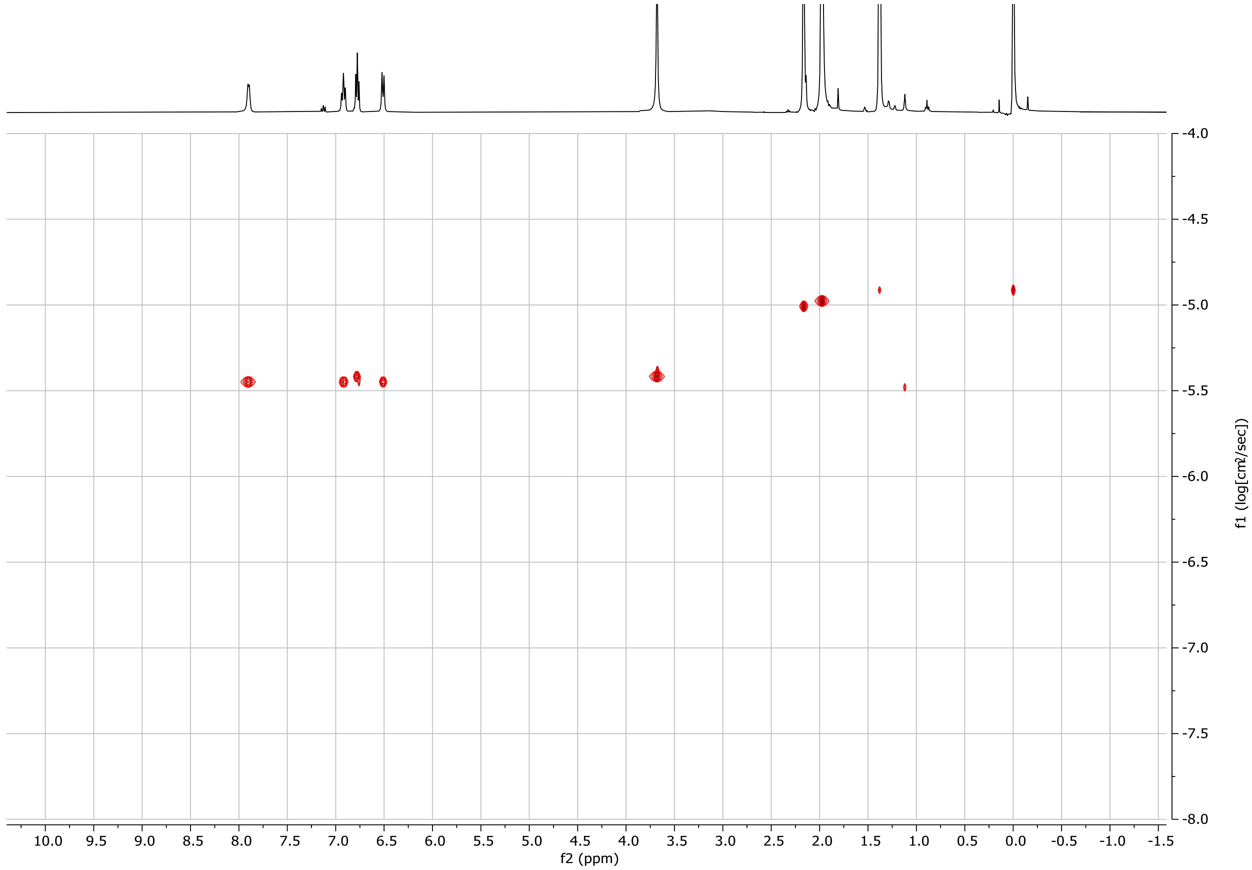

Supplementary Figure 10 ^{1}H DOSY NMR spectrum of 1:1 (2-C_6H_4OMeNa):(TMEDA) in C_6-D_{12}.

^{1}H DOSY NMR of 1:4 (C_6H_4OMeNa):(TMEDA)

Using C_6D_{12} as an internal standard, we calculated the estimated molecular weight of the formed aggregate from a 1:4 mixture of C_6H_4OMeNa and TMEDA to be 609 g mol^{-1}.

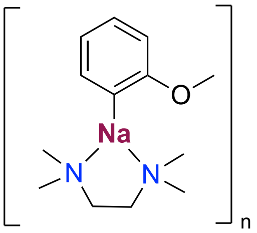

n = 1 : mw = 246 g mol^{-1}
n = 2 : mw = 493 g mol^{-1}
n = 3 : mw = 739 g mol^{-1}

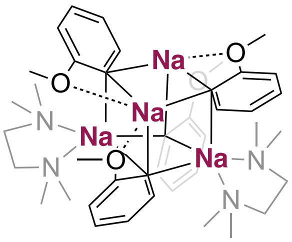

mw = 753 g mol^{-1}

Average diffusion coefficient = 3.634 x 10^{-10} m^2 s^{-1}

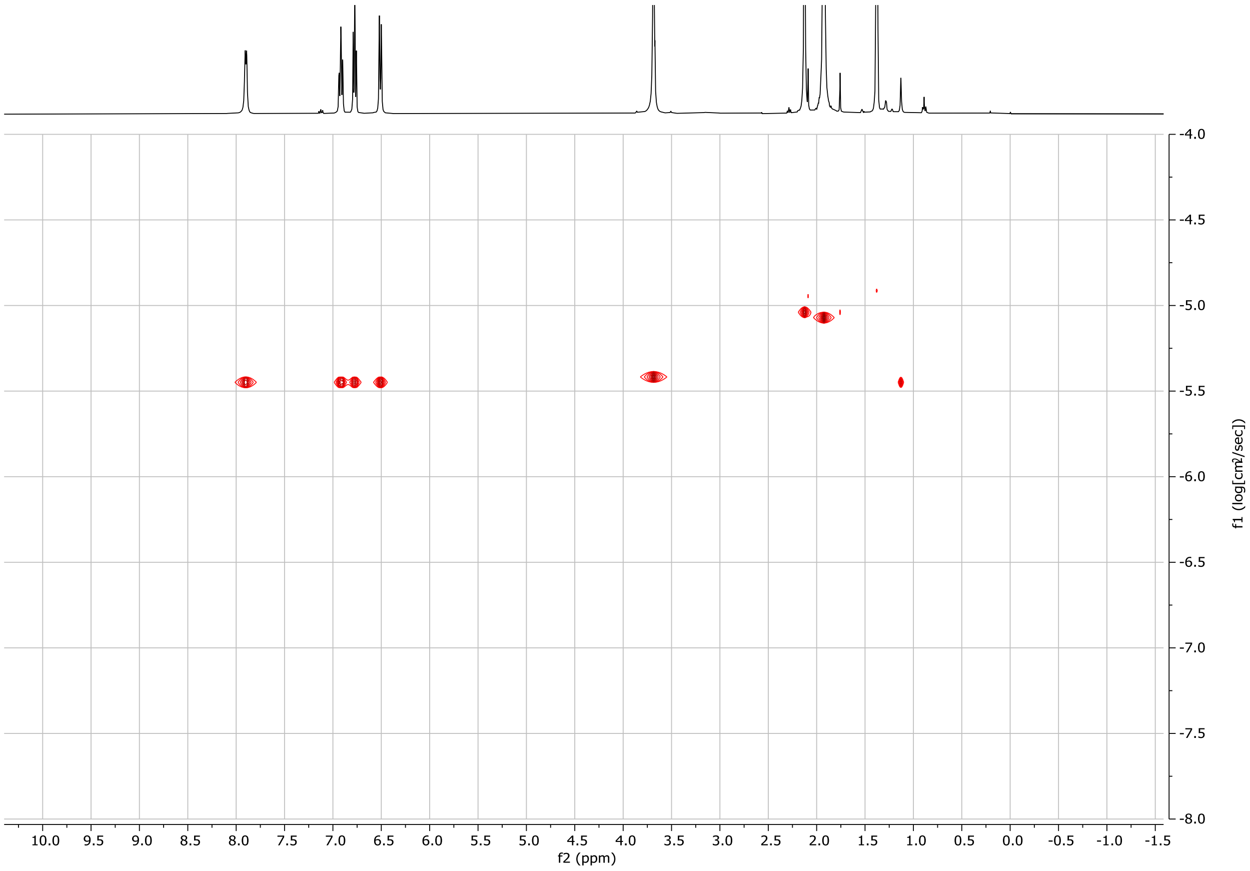

Supplementary Figure 11. ^{1}H DOSY NMR spectrum of 1:4 (2-C_6H_4OMeNa):(TMEDA) in C_6D_{12}.

^{1}H DOSY NMR of 1:1 (C_6H_4OMeNa):(PMDETA)

Using C_6D_{12} as an internal standard, we calculated the estimated molecular weight of the formed aggregate from a 1:1 mixture of C_6H_4OMeNa and PMDETA to be 1245 g mol^{-1}.

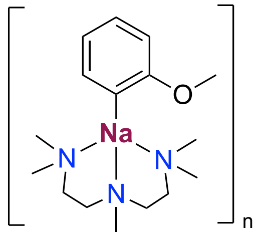

n = 1 : mw = 303 g mol^{-1}
n = 2 : mw = 606 g mol^{-1}
n = 3 : mw = 909 g mol^{-1}
n = 4 : mw = 1212 g mol^{-1}

Average diffusion coefficient = 3.461 x 10^{-10} m^2 s^{-1}

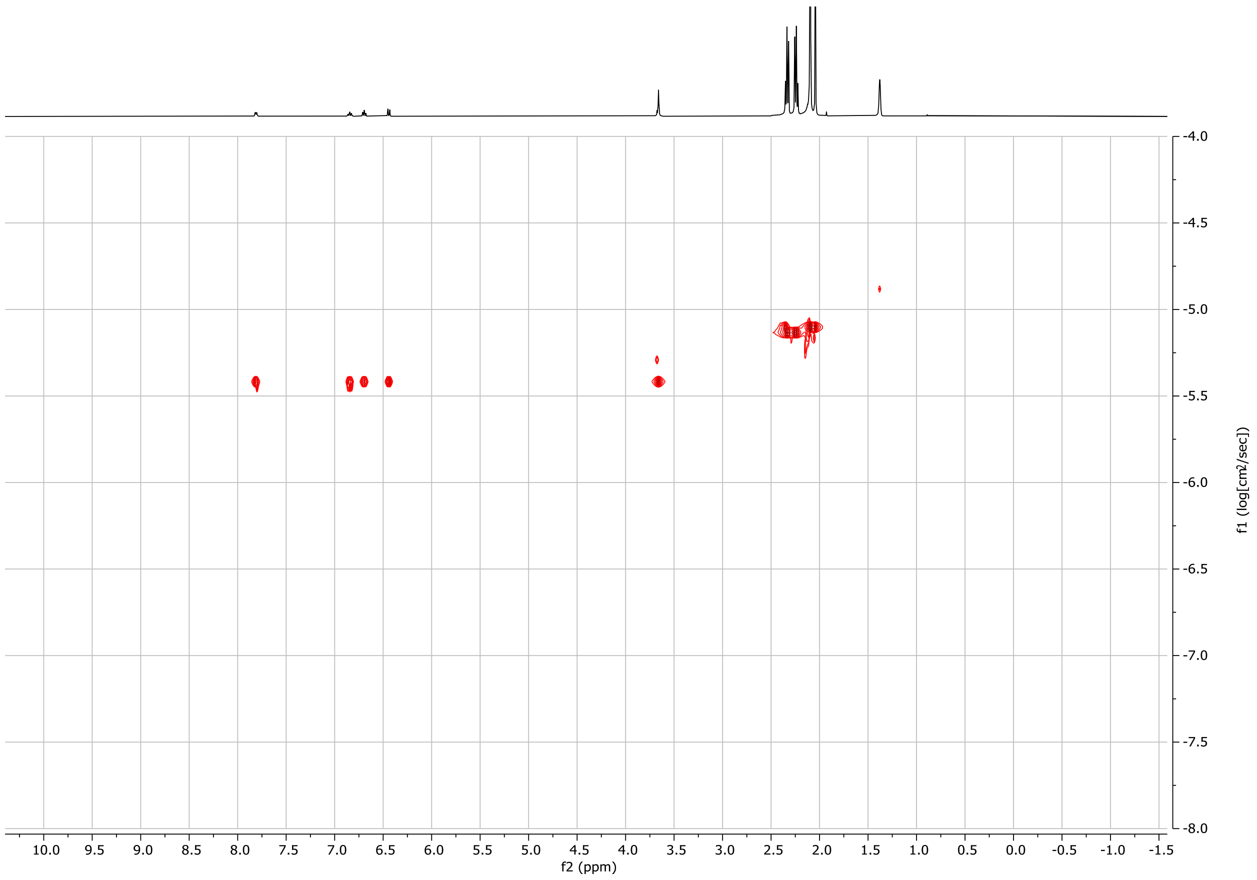

Supplementary Figure 12. ^{1}H DOSY NMR spectrum of 1:1 (2-C_6H_4OMeNa):(PMDETA) in C_6D_{12}.

^{1}H DOSY NMR of 1:4 (C_6H_4OMeNa):(THF)

Using C_6D_{12} as an internal standard, we calculated the estimated molecular weight of the formed aggregate from a 1:4 mixture of C_6H_4OMeNa and THF to be 631 g mol^{-1}.

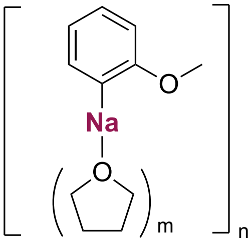

n = 1, m = 1 : mw = 202 g mol^{-1}
n = 1, m = 3 : mw = 346 g mol^{-1}
n = 2, m = 2 : mw = 548 g mol^{-1}
n = 2, m = 3 : mw = 692 g mol^{-1}

Average diffusion coefficient = 3.647 x 10^{-10} m^2 s^{-1}

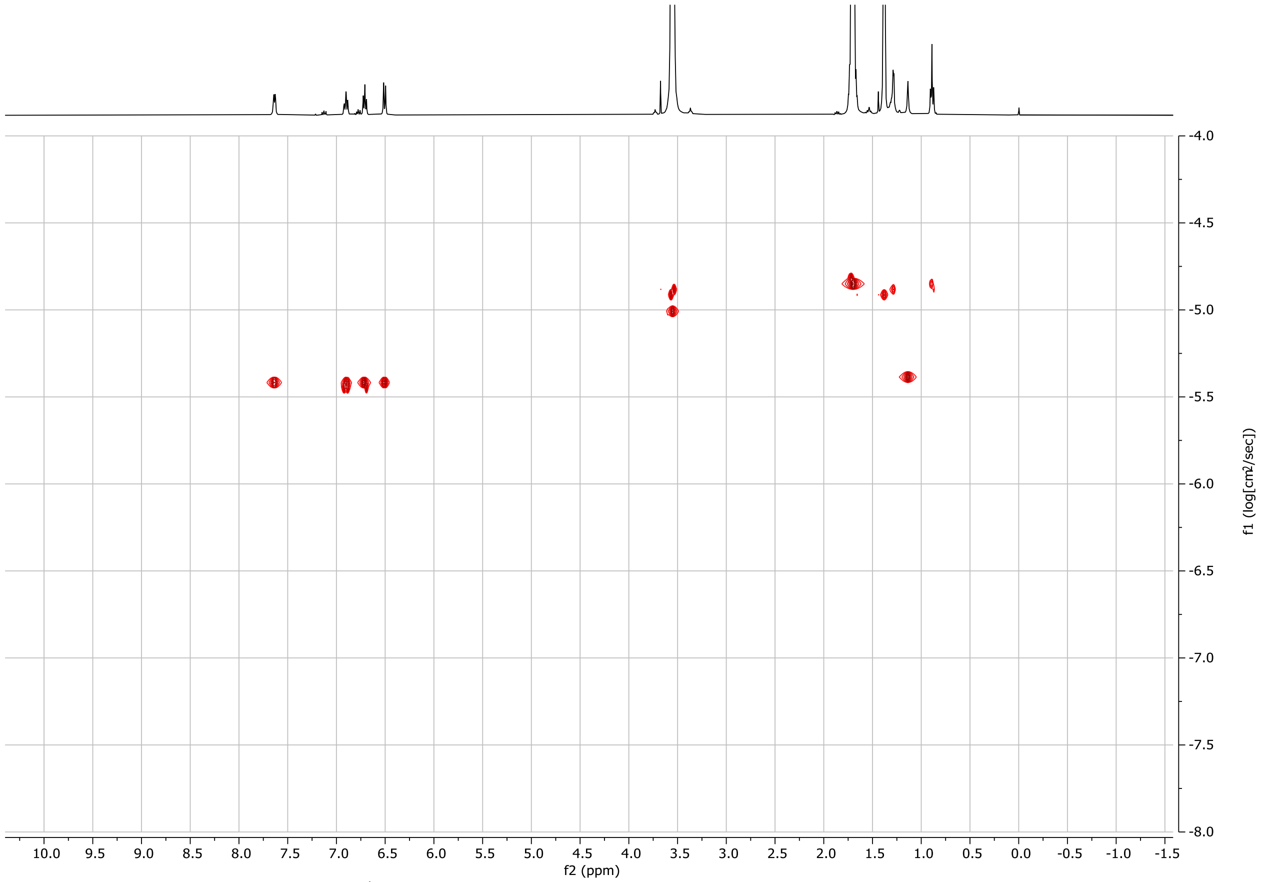

Supplementary Figure 13. ^{1}H DOSY NMR spectrum of 1:4 (2-C_6H_4OMeNa):(THF) in C_6D_{12}.

^{1}H NMR of 1:1 (C_6H_4OMeNa):(15-crown-5)

^{1}H NMR of 1:1 mixture of C_6H_4OMeNa and 15-crown-5 resulted in rapid decomposition of the donor and hydrolysis of the aryl sodium to form anisole.

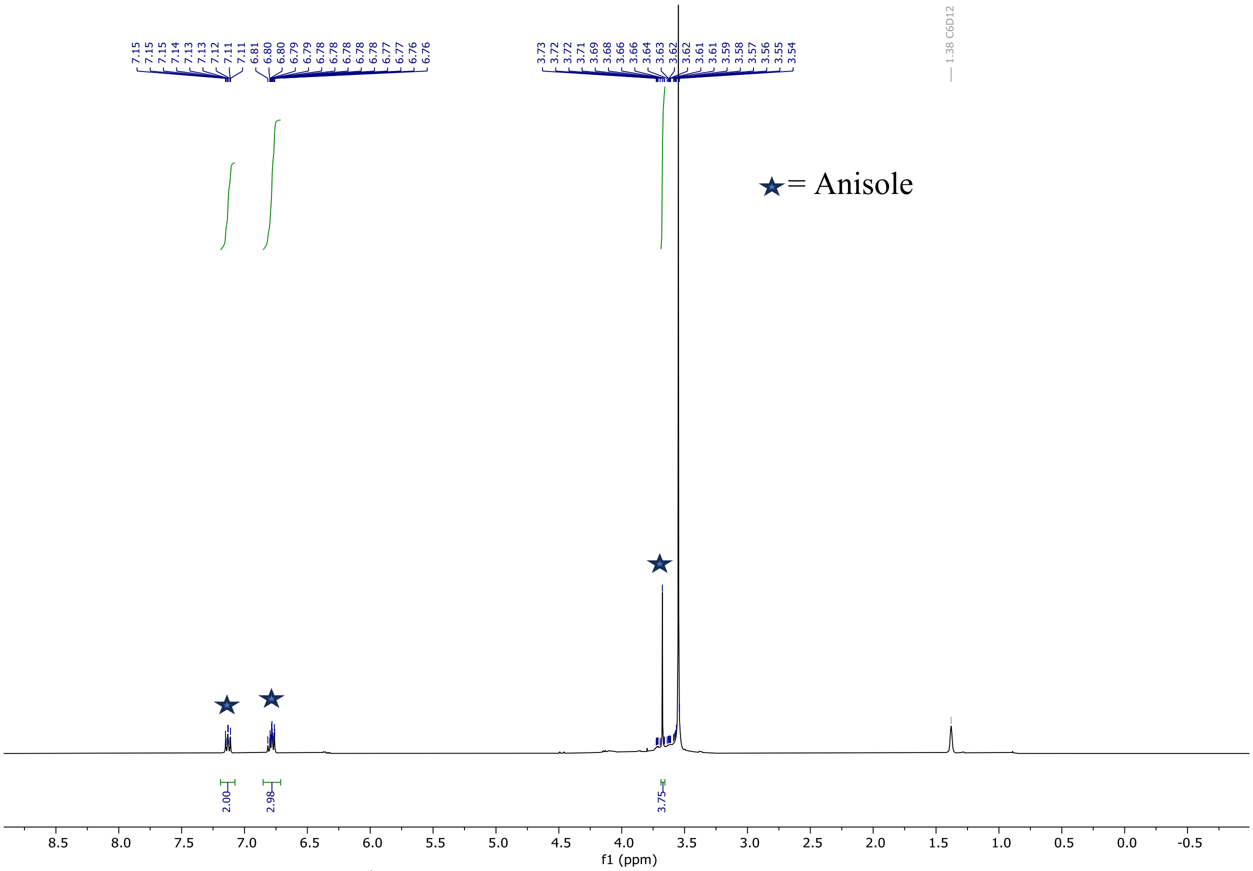

Supplementary Figure 14. ^{1}H NMR spectrum of 1:1 (2-C_6H_4OMeNa):(15-crown-5) in C_6D_{12}.

10. Stoichiometric reactions with Fe-1 and Fe-2

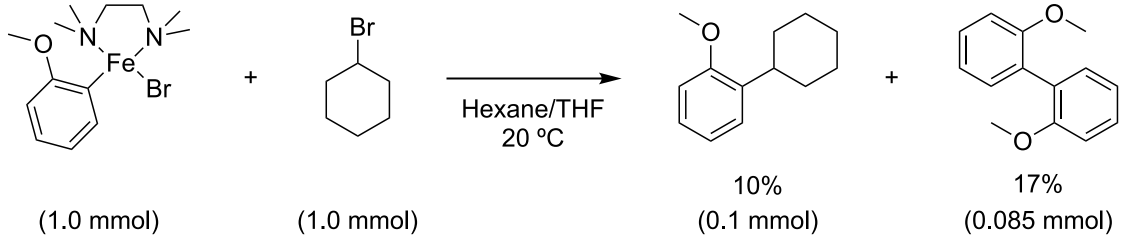

Complex [(TMEDA)FeBr(C_6H_4OMe)] (**Fe-1**) was prepared in solution, as described in section 8. Instead of placing it in the freezer for crystallisation, the reaction was transferred into an ampule and on the Schlenk line bromocyclohexane (1 mmol, 123 μL) was added and the reaction stirred for an hour. After that time, the reaction was quenched with water, extracted with ethyl acetate and hexamethylbenzene was added as an internal standard. An aliquot was evaporated under vacuum and a ^{1}H NMR spectrum was recorded, showing the formation of the cross-coupled product in 10% yield and 2,2’-dimethoxybiphenyl (**12**) in 17% yield.

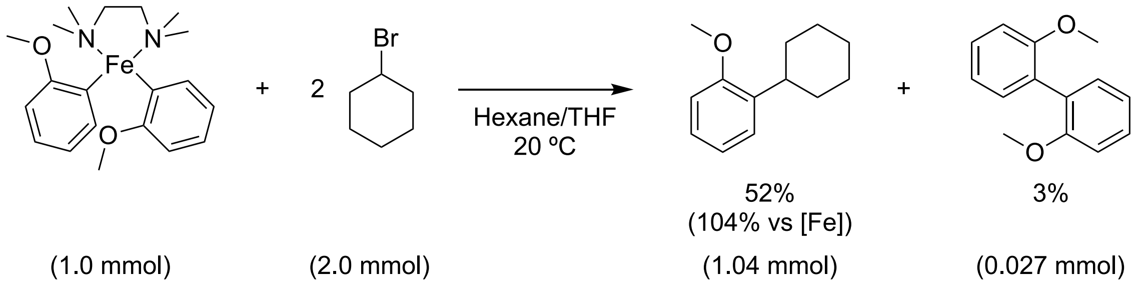

Complex [(TMEDA)Fe(C_6H_4OMe)$_2$] (**Fe-2**) was prepared in solution, as described in section 8. Instead of placing it in the freezer for crystallisation, the reaction was transferred into an ampule and on the Schlenk line bromocyclohexane (2 mmol, 246 μL) was added and the reaction stirred for an hour. After that time, the reaction was quenched with water, extracted with ethyl acetate and hexamethylbenzene was added as an internal standard. An aliquot was evaporated under vacuum and a ^{1}H NMR spectrum was recorded, showing the formation of the cross-coupled product in 52% yield and 2,2’-dimethoxybiphenyl (**12**) in 3% yield.

Stoichiometric reactions with different Fe:12_{Na} ratios

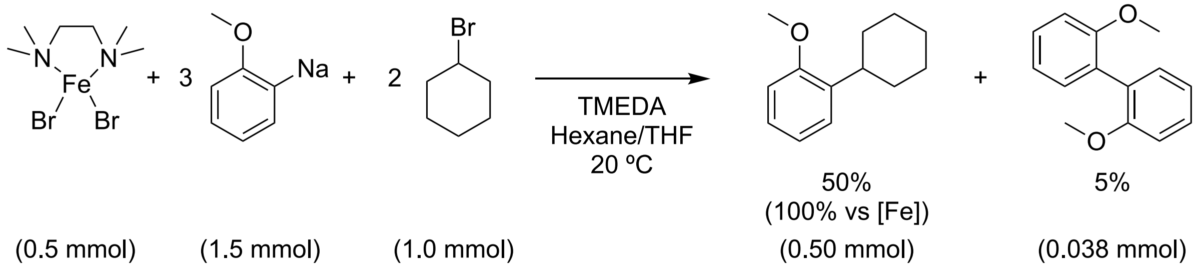

In a glove box, $FeBr_2$ (0.5 mmol, 108 mg) was suspended in THF (3 mL) and TMEDA (0.5 mmol, 75 μL) was added to form a brown suspension. In a separate vial, anisole (1.5 mmol, 168 μL), $NaCH_2SiMe_3$ (1.5 mmol, 165 mg), and TMEDA (2 mmol, 300 μL) were mixed in hexane (3 mL) and stirred for 1 hour. The second solution (metalated anisole) was added dropwise at room temperature into the (TMEDA)$FeBr_2$ solution. A white solid formed and instead of placing it in the freezer for crystallisation, the reaction mixture was transferred into an ampule, bromocyclohexane (1 mmol, 123 μL) was added on the Schlenk line, and the reaction was stirred for an hour. The reaction was then quenched with water, extracted with ethyl acetate and hexamethylbenzene was added as an internal standard. An aliquot was evaporated under vacuum and a ^{1}H NMR spectrum was recorded, showing the formation of the cross-coupled product in 50% yield and 2,2'-dimethoxybiphenyl (**12**) in 5% yield.

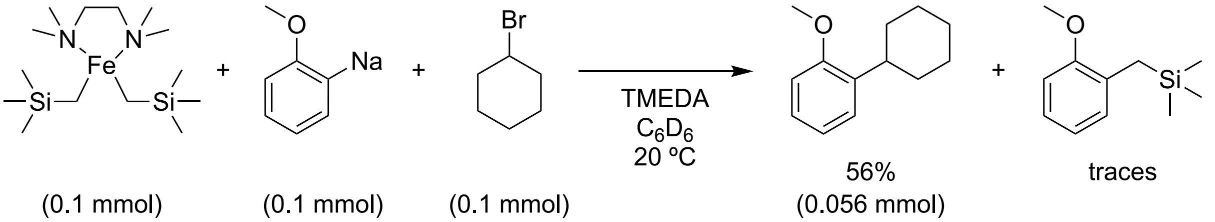

In a glove box, (TMEDA)$Fe(CH_2SiMe_3)_2$ (0.1 mmol, 35 mg) and 2-C_6H_4OMeNa (0.1 mmol, 13 mg) were dissolved in C_6D_6, TMEDA (0.1 mmol, 15 μL) was added, and the dark red reaction mixture was monitored by ^{1}H NMR. After 1 hour, bromocyclohexane (0.1 mmol, 12 μL) was added and the reaction was monitored for another hour. After this time, the reaction was quenched with MeOD and filtered through a plug of silica. A ^{1}H NMR spectrum of the resulting reaction mixture was recorded, showing the formation of cross-coupled product in 56% yield and traces of (2-methoxybenzyl)trimethylsilane.

11. Crystallographic details

Supplementary Table 6. Main crystallographic parameters.

Compound	[($C_6H_4OMeNa)_4(TMEDA)_2$]	Fe-1	Fe-2
**CCDC Number**	2375563	2375564	2375562
**Empirical formula**	$C_{40}H_{60}N_4Na_4O_4$	$C_{13}H_{23}N_2OFeBr$	$C_{20}H_{30}FeN_2O_2$
**Mol. Mass**	752.88	359.09	386.31
**Temperature/K**	173.00(10)	173.01(10)	173.00(10)
**Crystal system**	triclinic	monoclinic	monoclinic
**Space group**	P-1	$P2_1/c$	$P2_1/n$
**a/Å**	11.54138(7)	9.8964(2)	7.22488(12)
**b/Å**	12.84046(9)	10.9650(2)	15.0195(3)
**c/Å**	17.51910(12)	14.9610(2)	18.1718(3)
**α/°**	106.7950(6)	90	90
**β/°**	93.7123(5)	98.6550(10)	90.9642(14)
**γ/°**	91.0038(5)	90	90
**$V/Å^3$**	2478.53(3)	1604.99(5)	1971.61(6)
**Z**	2	4	4
**λ/Å**	1.54184	0.71073	0.71073
**2Θ range for data collection/°**	5.284 to 148.998	4.624 to 61.012	4.484 to 61.016
**$\rho_{calc}g/cm^3$**	0.972	1.486	1.301
**μ/mm^{-1}**	0.815	3.421	0.780
**F(000)**	808.0	736.0	824.0
**$Crystal\ size/mm^3$**	0.346 × 0.258 × 0.183	0.25 × 0.103 × 0.085	0.371 × 0.067 × 0.046
**Reflection collected**	102402	47417	52988
**Unique reflections**	4248	4904	6021
***R*int**	0.0205	0.0269	0.0301
**Goof**	1.036	1.061	1.038
**Final R indexes [I>=2σ (I)]**	R_1 = 0.0340, wR_2 = 0.1022	R_1 = 0.0229, wR_2 = 0.0556	R_1 = 0.0314, wR_2 = 0.0739
**Final R indexes [all data]**	R_1 = 0.0352, wR_2 = 0.1033	R_1 = 0.0305, wR_2 = 0.0577	R_1 = 0.0417, wR_2 = 0.0773
**Largest diff. peak/hole / e $Å^{-3}$**	0.16/-0.17	0.76/-0.45	0.40/-0.16

- [$(C_6H_4OMeNa)_4(TMEDA)_2$]

Crystal-Structure Determination. A crystal of $C_{40}H_{60}N_4Na_4O_4$ immersed in parabar oil was mounted at 173K using the X-TEMP2[28,29] cold temperature device under the microscope, carried to the diffractometer inside a dewar containing liquid nitrogen and then transferred into a stream of gaseous nitrogen (173 K). All measurements were made on a *RIGAKU Synergy S* area-detector diffractometer[30] using mirror optics monochromated Cu *K*α radiation (λ = 1.54184 Å). The unit cell constants and an orientation matrix for data collection were obtained from a least-squares refinement of the setting angles of reflections in the range 2.640° < θ < 79.881°. A total of 11540 frames were collected using ω scans, with 0.3 second exposure time (1.2 s for high-angle reflections), a rotation angle of 0.5° per frame, a crystal-detector distance of 31.0 mm, at T = 173.00(10) K.

- [(TMEDA)FeBr(C_6H_4OMe)] (Fe-1)

Crystal-Structure Determination. A crystal of $C_{13}H_{23}N_2OFeBr$ immersed in parabar oil was mounted at –30°C using the X-TEMP2[28,29] cold temperature device under the microscope, carried to the diffractometer inside a dewar containing liquid nitrogen and then transferred into a stream of gaseous nitrogen (173 K). All measurements were carried out on a *RIGAKU XtaLAB Synergy R*, HyPix-Arc 100 area-detector diffractometer[28] using mirror optics monochromated Mo *K*α radiation (λ = 0.71073 Å). The unit cell constants and an orientation matrix for data collection were obtained from a least-squares refinement of the setting angles of reflections in the range 2.300° < θ < 32.504°. A total of 1720 frames were collected using ω scans, with 2.0 seconds exposure time, a rotation angle of 0.5° per frame, a crystal-detector distance of 43.0 mm, at T = 173.0(1) K.

- [(TMEDA)Fe$(C_6H_4OMe)_2$] (Fe-2)

Crystal-Structure Determination. A crystal of $C_{20}H_{30}FeN_2O_2$ immersed in parabar oil was mounted at 173K using the X-TEMP2[28,29] cold temperature device under the microscope, carried to the diffractometer inside a dewar containing liquid nitrogen and then transferred into a stream of gaseous nitrogen (173 K). All measurements were made on a *RIGAKU XtaLAB Synergy R*, HyPix-Arc 100 area-detector diffractometer[30] using mirror optics monochromated Mo *K*α radiation (λ = 0.71073 Å). The unit cell constants and an orientation matrix for data collection were obtained from a least-squares refinement of the setting angles of reflections in the range 2.248° < θ < 32.576°. A total of 1740 frames were collected using ω scans, with 8 seconds exposure time, a rotation angle of 0.5° per frame, a crystal-detector distance of 43.0 mm, at T = 173.00(10) K.

Data reduction was performed using the *CrysAlisPro*[30] program. The intensities were corrected for Lorentz and polarisation effects, and a numerical absorption correction based on gaussian integration over a multifaceted crystal model with additional empirical absorption correction using spherical harmonics using SCALE3 ABSPACK in *CrysAlisPro*[30] was applied. Data collection and refinement parameters are given in Supplementary Table 6.

The structure was solved by intrinsic phasing using *SHELXT*[31], which revealed the positions of all non-hydrogen atoms of the title compound. All non-hydrogen atoms were refined anisotropically. H-atoms were assigned in geometrically calculated positions and refined using a riding model where each H-atom was assigned a fixed isotropic displacement parameter with a value equal to 1.2Ueq of its parent atom (1.5Ueq for methyl groups).

Refinement of the structure was carried out on F^2 using full-matrix least-squares procedures, which minimised the function $\Sigma w(F_o^2 - F_c^2)^2$. The weighting scheme was based on counting statistics and included a factor to downweight the intense reflections. All calculations were performed using the *SHELXL-2014/7*[32] program in OLEX2[33].

For **[(C$_6$H$_4$OMeNa)$_4$(TMEDA)$_2$]** disorder model was used for parts of the structure where the occupancies of each disorder component were refined through the use of a free variable. The sum of equivalent components was constrained to 1, i.e. 100%. Areas containing disorder solvents (pentane) were found where a satisfactory solvent model could not be achieved, therefore, a solvent mask was used to include the contribution of electron density found in void areas into the calculated structure factor.

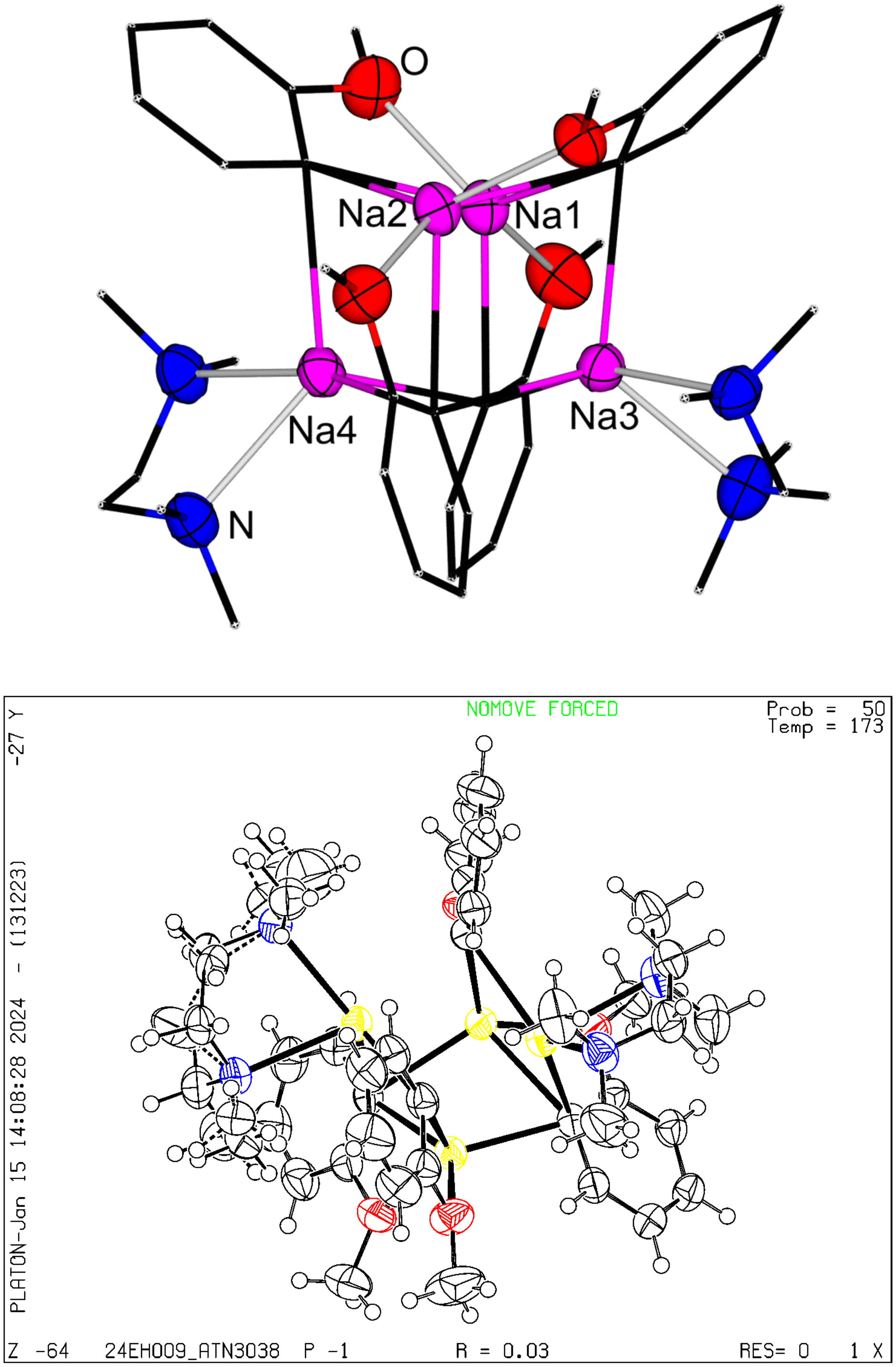

Supplementary Figure 15. Top: Molecular structure of [$(C_6H_4OMeNa)_4(TMEDA)_2$] (CCDC deposition number: 2375563), using a mixture of ellipsoids and sticks for its representation. All H atoms and disorder in a TMEDA fragment have been omitted for clarity. Bottom: ORTEP representation.

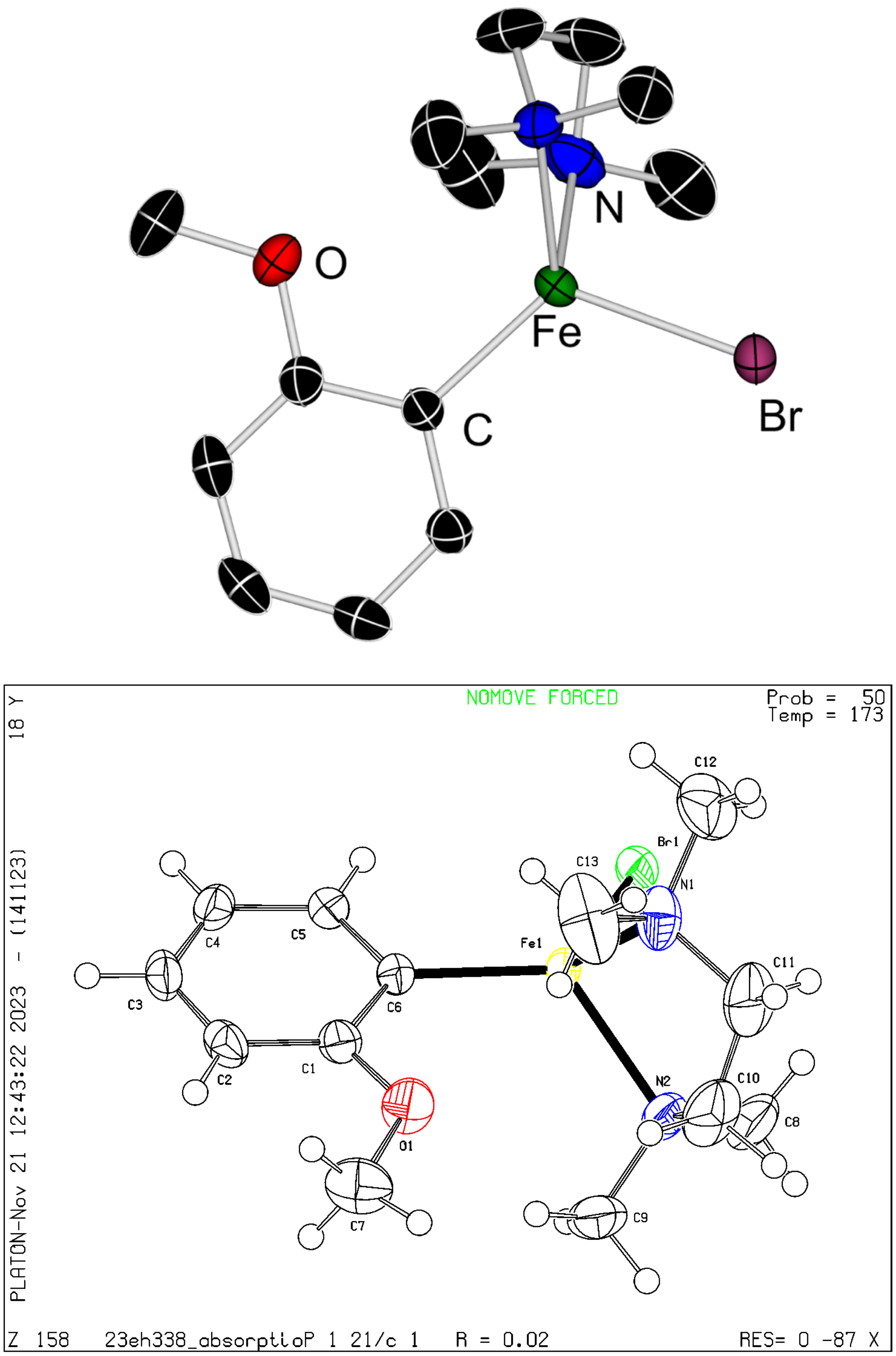

Supplementary Figure 16. Top: Molecular structure of [(TMEDA)FeBr(C_6H_4OMe)] (**Fe-1**) (CCDC deposition number: 2375564). Ellipsoids are shown at 50% probability and all H atoms have been omitted for clarity. Bottom: ORTEP representation.

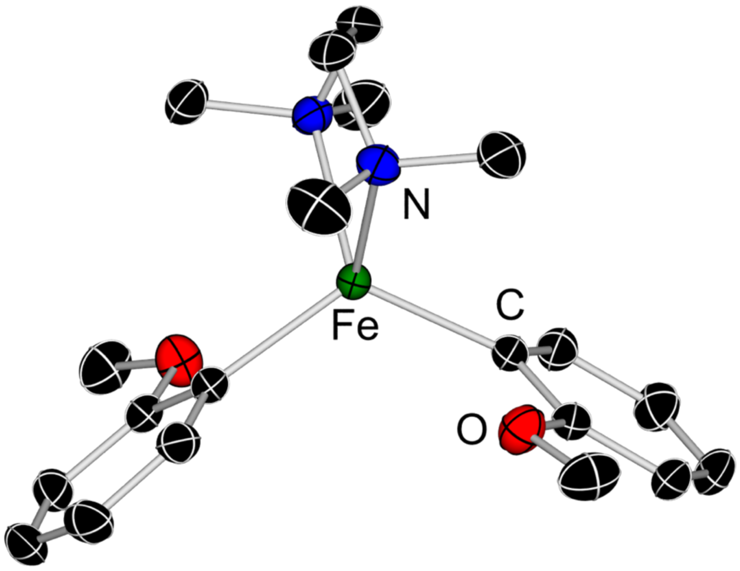

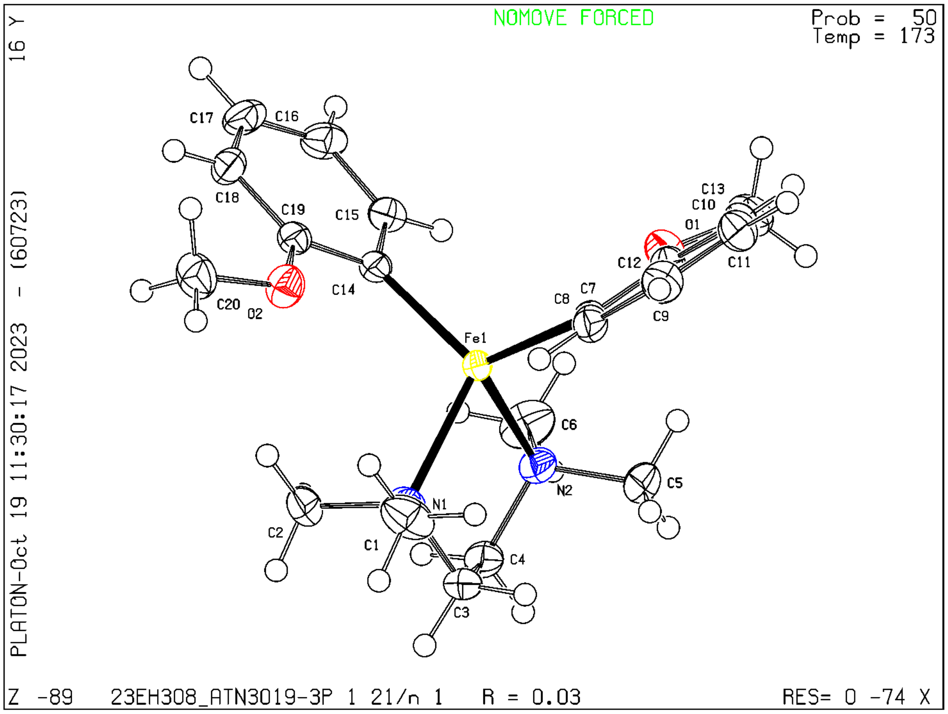

Supplementary Figure 17. Top: Molecular structure of [(TMEDA)Fe(C_6H_4OMe)$_2$] (**Fe-2**) (CCDC deposition number: 2375562). Ellipsoids are shown at 50% probability and all H atoms have been omitted for clarity. Bottom: ORTEP representation.

II. Supplementary References

1. Still, W. C., Kahn, M. & Mitra, A. Rapid chromatography technique for preparative separations with moderate resolution. *J. Org. Chem.* **43**, 2923–2925 (1978).
2. Pangborn, A. B., Giardello, M. A., Grubbs, R. H., Rosen, R. K. & Timmers, F. J. Safe and convenient procedure for solvent purification. *Organometallics* **15**, 1518–1520 (1996).
3. Mita, T., Tanaka, H., Michigami, K. & Sato Y. Ruthenium-catalyzed C–H silylation of 1-arylpyrazole derivatives and fluoride-mediated carboxylation: Use of two nitrogen atoms of the pyrazole group. *Synthesis* **25**, 1291–1294 (2014).
4. Monguchi, Y., Kitamoto, K., Ikawa, T., Maegawa, T. & Sajiki, H. Evaluation of aromatic amination catalyzed by palladium on carbon: A practical synthesis of triarylamines. *Adv. Synth. Catal.* **350**, 2767–2777 (2008).
5. Bull, J. A., Mousseau, J. J. & Charette, A. B. Convenient one-pot synthesis of (*E*)-*β*-aryl vinyl halides from benzyl bromides and dihalomethanes. *Org. Lett.* **10**, 5485–5488 (2008).
6. Murugan, K., Nainamalai, D., Kanagaraj, P., Nagappan, S. G. & Palaniswamy, S. Green-synthesized nickel nanoparticles on reduced graphene oxide as an active and selective catalyst for Suzuki and Glaser-Hay coupling reactions. *Appl. Organomet. Chem.* **34**, e5778 (2020).
7. Liu, W. & Lei, A. Efficient Fe-catalyzed homo-coupling of aryl Grignard reagents using O_2 as the oxidant. *Tetrahedron Lett.* **49**, 610–613 (2008).
8. Raul, P. K., Mahanta, A., Bora, U., Thakur, A. J. & Veer, V. In water homocoupling of arylboronic acids using nano-rod shaped and reusable copper oxide(II) catalyst at room temperature. *Tetrahedron Lett.* **56**, 7069–7073 (2015).
9. Pu, F., Zhang, L.-Y., Liu, Z.-W. & Shi, X.-Y. Palladium (II)-catalyzed decarboxylative cross dehydrogenative coupling: Direct synthesis of *meta*-substituted biaryls from aromatic acids. *Adv. Synth. Catal.* **360**, 2644–2649 (2018).
10. Lee, P. H., Seomoon, D. & Lee, K. Palladium-catalyzed inter- and intramolecular coupling reactions of aryl and vinyl halides mediated by indium. *Org. Lett.* **7**, 343–345 (2005).
11. Wiensch, E. M., Todd, D. P. & Montgomery, J. Silyloxyarenes as versatile coupling substrates enabled by nickel-catalyzed C–O bond cleavage. *ACS Catal.* **6**, 5568–5571 (2009).
12. Lv, D., Zhang, Y.-Y. & Li, J.-H. One-pot, nickel triflate-catalysed homocoupling of aryl chlorides in the presence of metallic magnesium. *J. Chem. Res.* **6**, 397–399 (2009).
13. Liu, Y., Bergès J., Zaid, Y., Chahdi, F. O., Lee, A. V. D. Harakat, D., Clot, E., Jaroschik, F. & Taillefer, M. Aerobic and ligand-free manganese-catalyzed homocoupling of arenes or aryl halides via in situ formation of aryllithiums. *J. Org. Chem.* **84**, 4413–4420 (2019).
14. Minus, M. B., Moor, S. R., Pary, F. F., Nirmani, L. P. T., Chwatko, M., Okeke, B., Singleton, J. E., Nelson, T. L., Lynd, N. A. & Anslyn, E. V. "Benchtop" biaryl coupling using Pd/Cu cocatalysis: Application to the synthesis of conjugated polymers. *Org. Lett.* **23**, 2873–2877 (2021).

15. Gragert, M. M., Tomov, A. K., Bettonville, S., Pannier, G., White, A. J. P. & Britovsek, G. J. P. Biaryl group 4 metal complexes as non-metallocene catalysts for polyethylene with long chain branching. *Eur. J. Inorg. Chem.* 4088–4092 (2020).
16. Cadge, J. A., Gates, P. J., Bower, J. F. & Russell, C. A. Migratory insertion of CO into a Au–C bond. *J. Am. Chem. Soc.* **144**, 19719–19725 (2022).
17. Kubota, K., Shizukuishi, N., Kudo, S. & Ito, H. Solid-state nickel(0)-mediated Yamamoto coupling enabled by mechanochemistry. *Chem. Lett.* **53**, upae056 (2024).
18. Itami, K., Tonogaki, K., Ohashi, Y. & Yoshida, J.-i. Rapid construction of multisubstituted olefin structures using vinylboronate ester platform leading to highly fluorescent materials. *Org. Lett.* **6**, 4093–4096 (2004).
19. Bortoluzzi, M., Marchetti, F., Pampaloni, G., Pinzino, C. & Zacchini, S. Oxidative dimerization of triarylamines promoted by WCl_6, including the solid state isolation and the crystallographic characterization of a triphenylammonium salt. *Inorg. Chem.* **55**, 887–893 (2016).
20. Chen, H., Hu, L., Ji., W., Yao, L. & Liao, X. Nickel-catalyzed decarboxylative alkylation of aryl iodides with anhydrides. *ACS Catal.* **8**, 10479–10485 (2018).
21. Wei, X.-J., Abdiaj, I., Sambiagio, C., Li, C., Zysman-Colman, Eli., Alcázar, J. & Noël, T. Visible-light-promoted iron-catalyzed $C(sp^2)$–$C(sp^3)$ Kumada cross-coupling in flow. *Angew. Chem. Int. Ed.* **58**, 13030–13034 (2019).
22. Liu, D., Li, Y., Qi, X., Liu, C., Lan, Y. & Lei, A. Nickel-catalyzed selective oxidative radical cross-coupling: An effective strategy for inert Csp3–H functionalization. *Org. Lett.* **17**, 998–1001 (2015).
23. Kato, T. & Maruoka, K. Design of bowl-shaped *N*-hydroxyimide derivatives as new organoradical catalysts for site-selective $C(sp^3)$–H bond functionalization reactions. *Angew. Chem. Int. Ed.* **59**, 14261–14264 (2020).
24. Czaplik, W. M., Mayer, M. & von Wangelin, A. J. Domino iron catalysis: direct aryl–alkyl cross-coupling. *Angew. Chem. Int. Ed.* **48**, 607–610 (2009).
25. Zhou, Y., Qiu, L., Li, J. & Xie, W. A general copper catalytic system for Suzuki–Miyaura cross-coupling of unactivated secondary and primary alkyl halides with arylborons. *J. Am. Chem. Soc.* **145**, 28146–28155 (2023).
26. Pilli, R., Selvam, K., Balamurugan, B. S. S., Jose, V. & Rasappan, R. $C(sp^3)$–$C(sp^3)$ Coupling of cycloalkanes and alkyl halides via dual photocatalytic hydrogen atom transfer and nickel catalysis. *Org. Lett.* **26**, 2993–2998 (2024).
27. Bachmann, S., Gernert, B. & Stalke, D. Solution structure of alkali metal cyclopentadienides in THF estimated by ECC-DOSY NMR-spectroscopy (incl. software). *Chem. Commun.* **52**, 12861–12864 (2016).
28. Kottke, T. & Stalke, D. Crystal handling at low temperatures. *J. Appl. Cryst.* **26**, 615–619 (1993).

29. Stalke, D. Cryo crystal structure determination and application to intermediates. *Chem. Soc. Rev.* **27**, 171–178 (1998).
30. Oxford Diffraction (2018). *CrysAlisPro* (Version 1.171.40.37a). Oxford Diffraction Ltd., Yarnton, Oxfordshire, UK.
31. Sheldrick, G. M. *SHELXT* – Integrated space-group and crystal-structure determination. *Acta Cryst.* **A71**, 3–8 (2015).
32. Sheldrick, G. M. Crystal structure refinement with *SHELXL*. *Acta Cryst.* **C71**, 3–8 (2015).
33. Dolomanov, O. V., Bourhis, L. J., Gildea, R. J., Howard, J. A. K. & Puschmann, H. *OLEX2*: a complete structure solution, refinement and analysis program. *J. Appl. Cryst.* **42**, 339–341 (2009).

III. Supplementary Figures

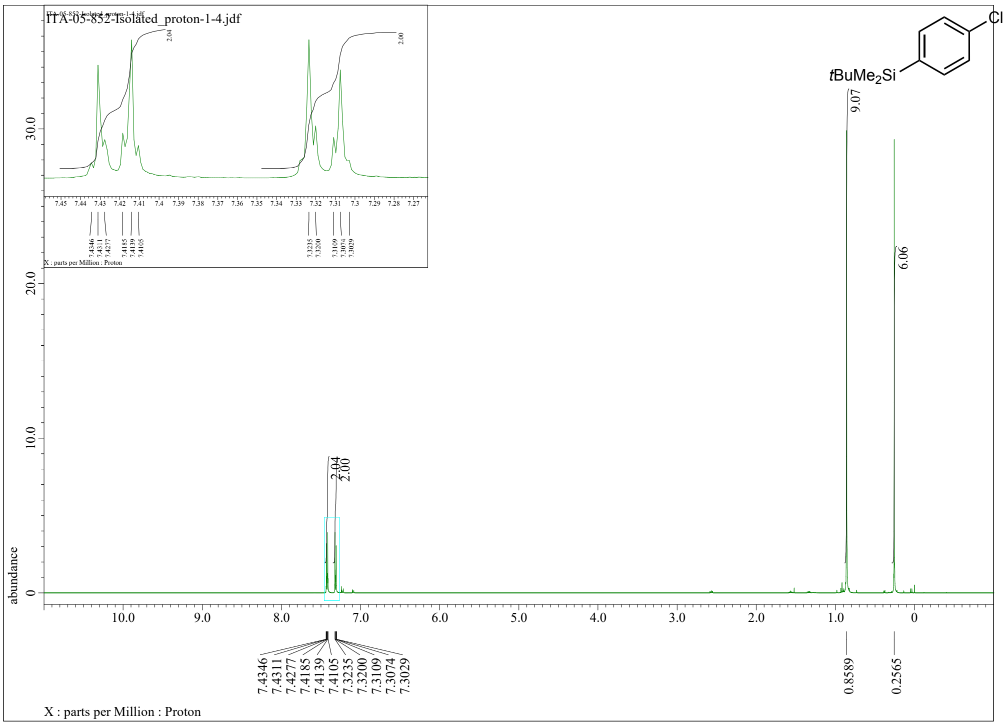

Supplementary Figure 18. ^{1}H NMR spectrum of **10'** (500 MHz, $CDCl_3$).

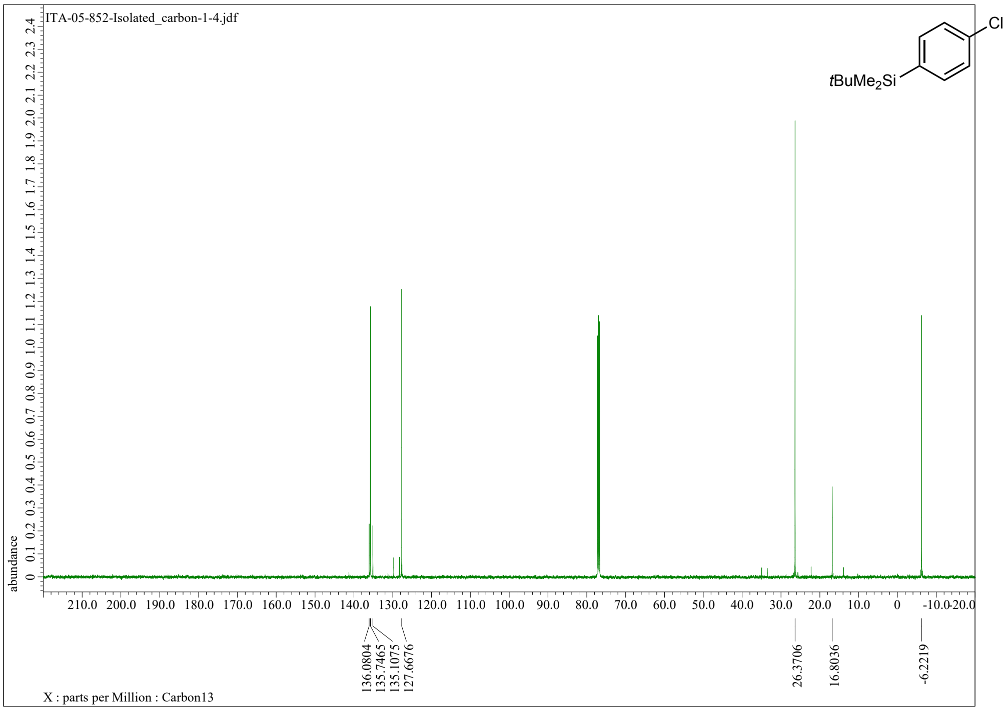

Supplementary Figure 19. ^{13}C NMR spectrum of **10'** (126 MHz, $CDCl_3$).

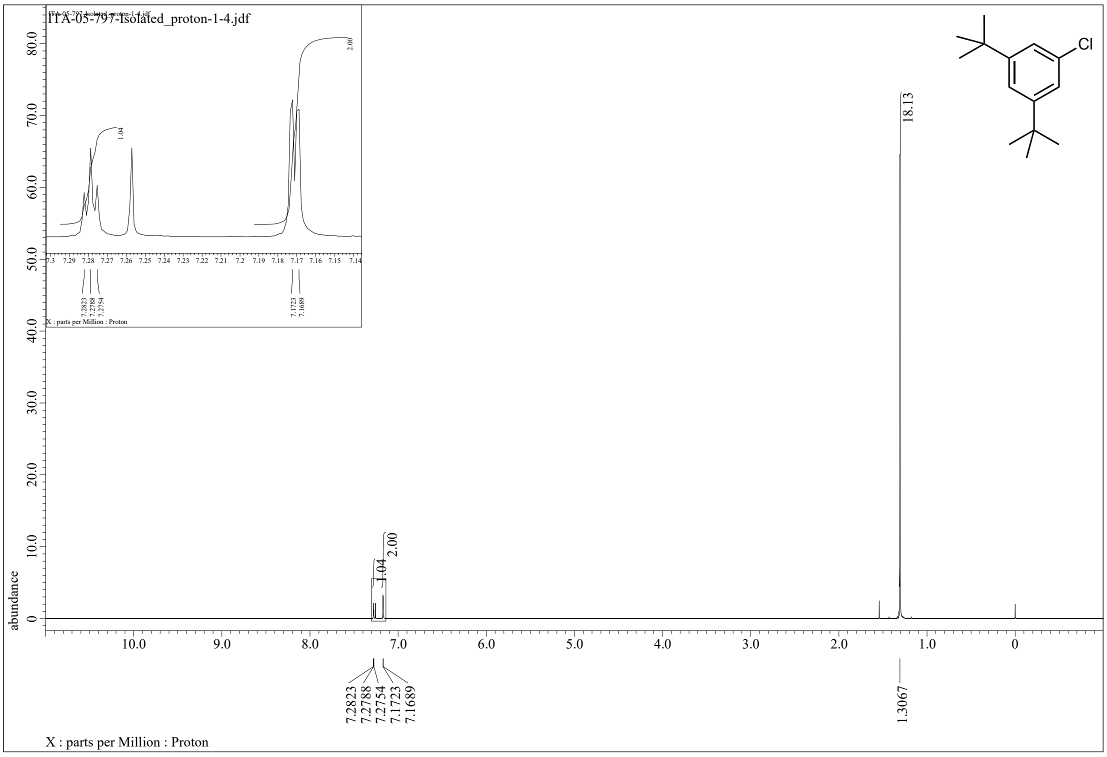

Supplementary Figure 20. ^{1}H NMR spectrum of **16’** (500 MHz, $CDCl_3$).

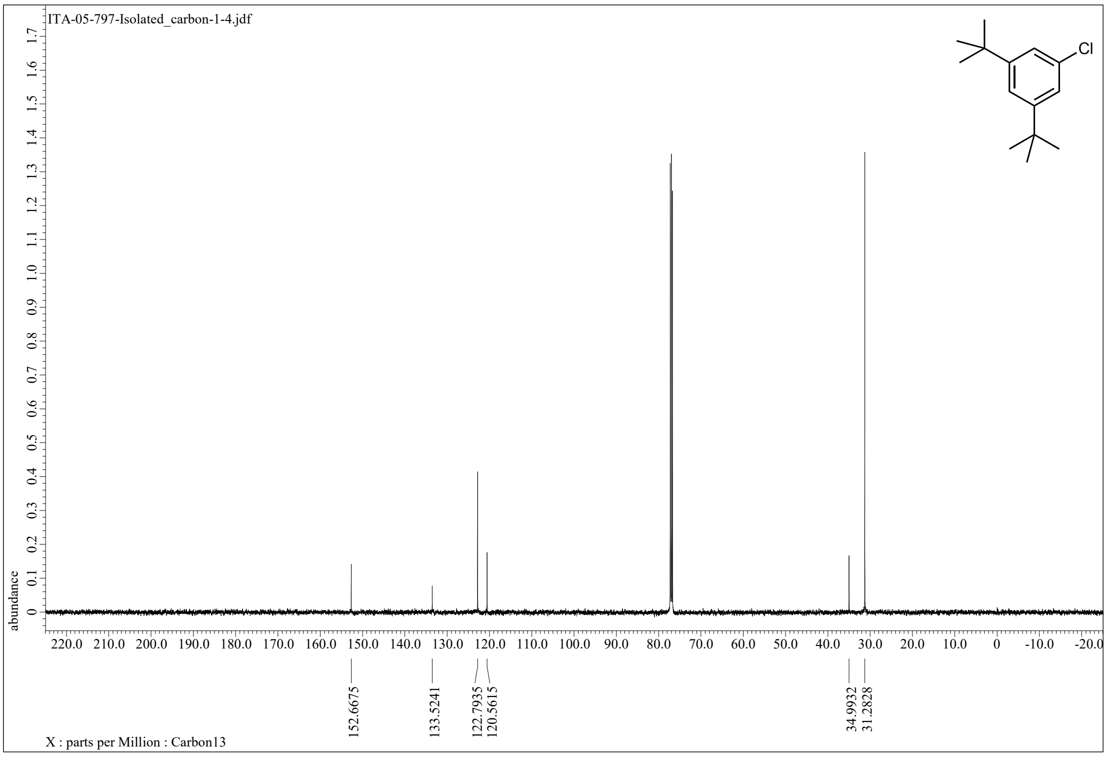

Supplementary Figure 21. ^{13}C NMR spectrum of **16’** (126 MHz, $CDCl_3$).

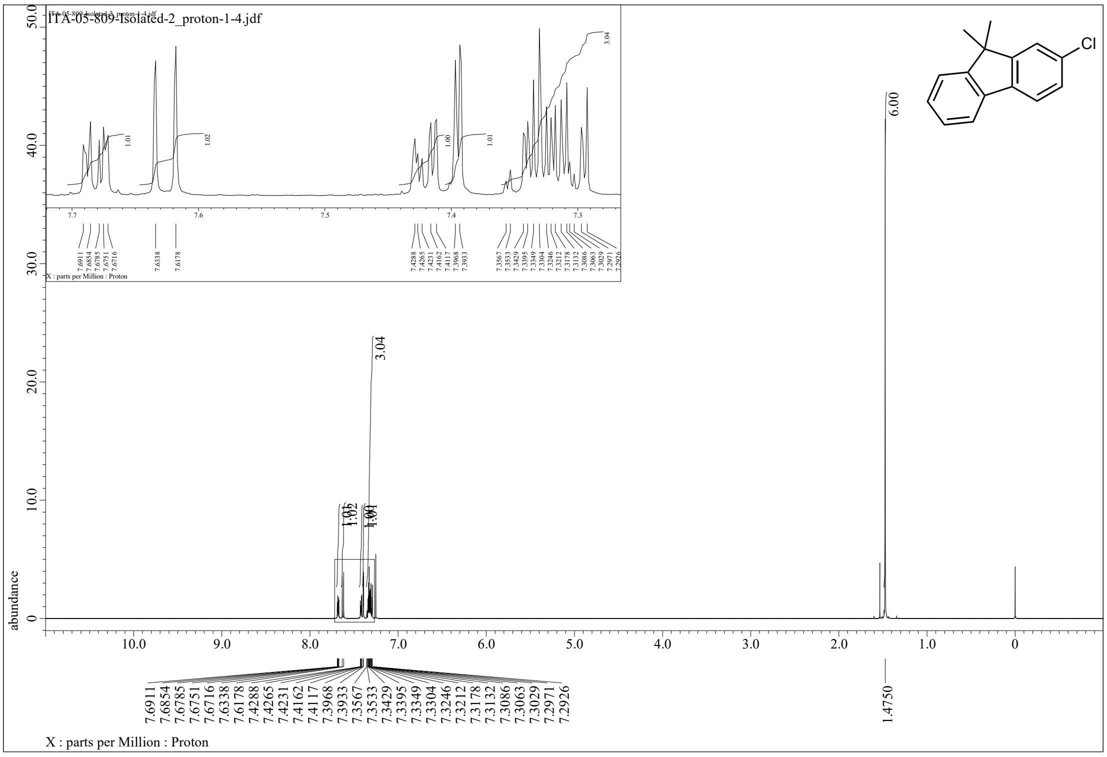

Supplementary Figure 22. ^{1}H NMR spectrum of **18’** (500 MHz, $CDCl_3$).

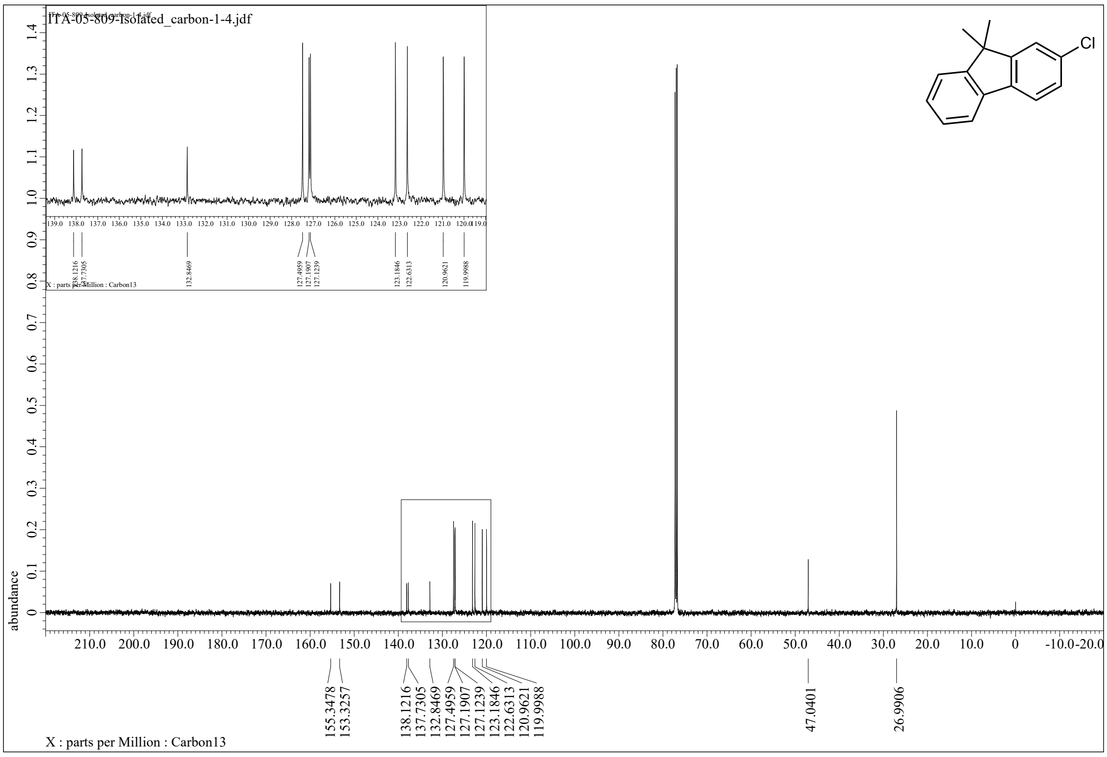

Supplementary Figure 23. ^{13}C NMR spectrum of **18’** (126 MHz, $CDCl_3$).

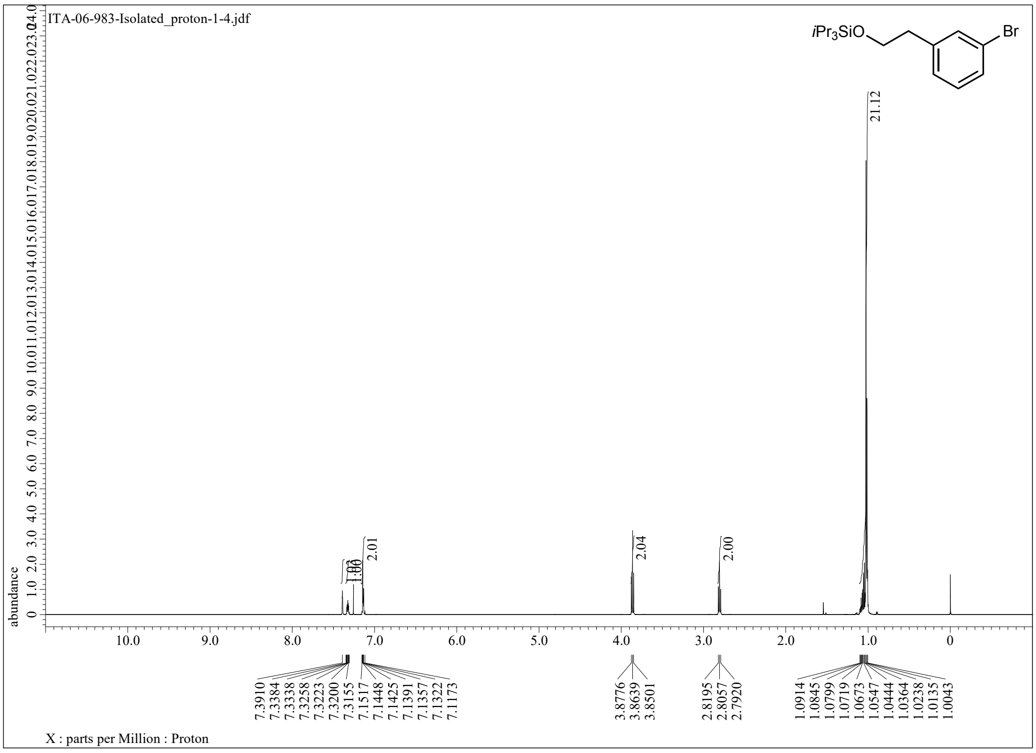

Supplementary Figure 24. ^{1}H NMR spectrum of **25'** (500 MHz, $CDCl_3$).

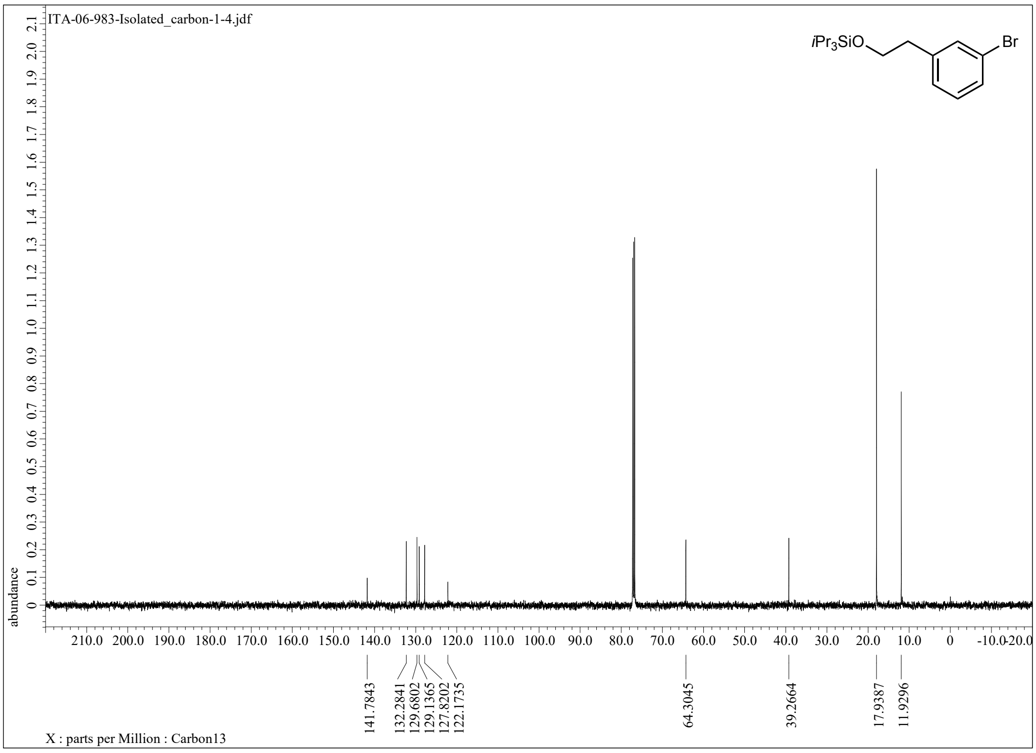

Supplementary Figure 25. ^{13}C NMR spectrum of **25'** (126 MHz, $CDCl_3$).

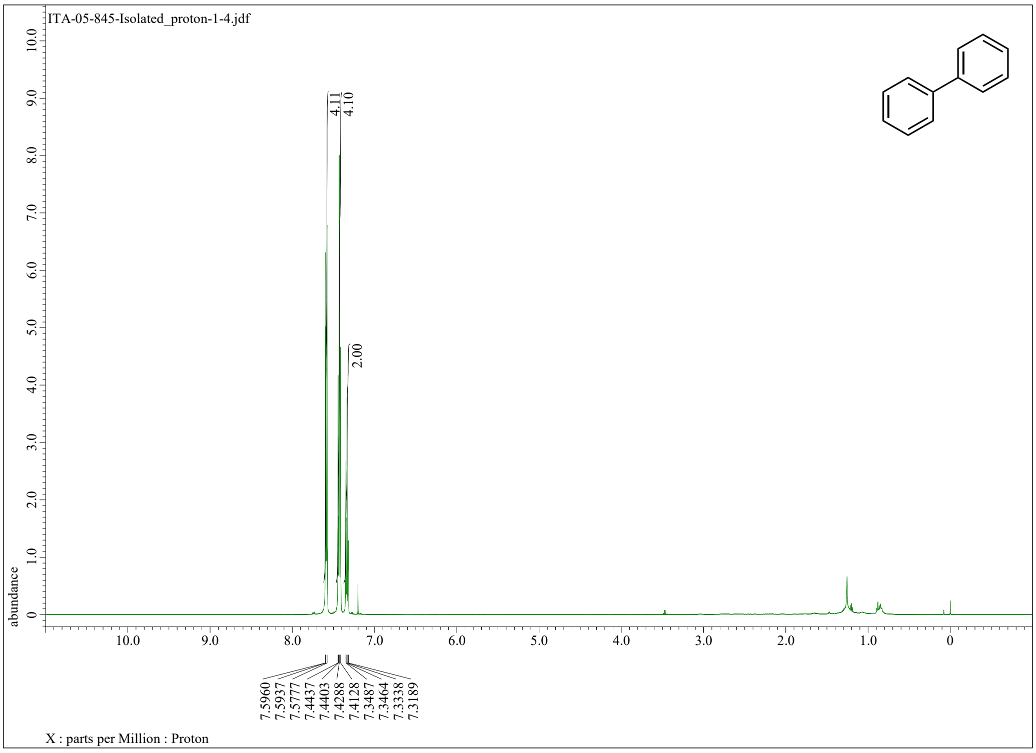

Supplementary Figure 26. ^{1}H NMR spectrum of **1** (500 MHz, $CDCl_3$).

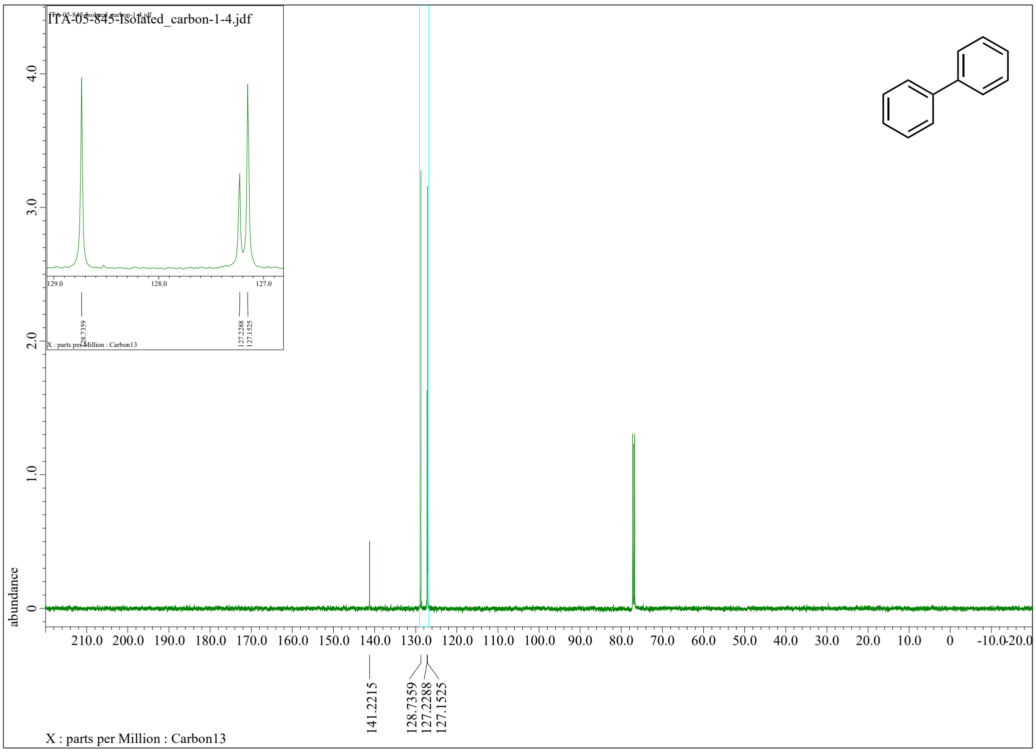

Supplementary Figure 27. ^{13}C NMR spectrum of **1** (126 MHz, $CDCl_3$).

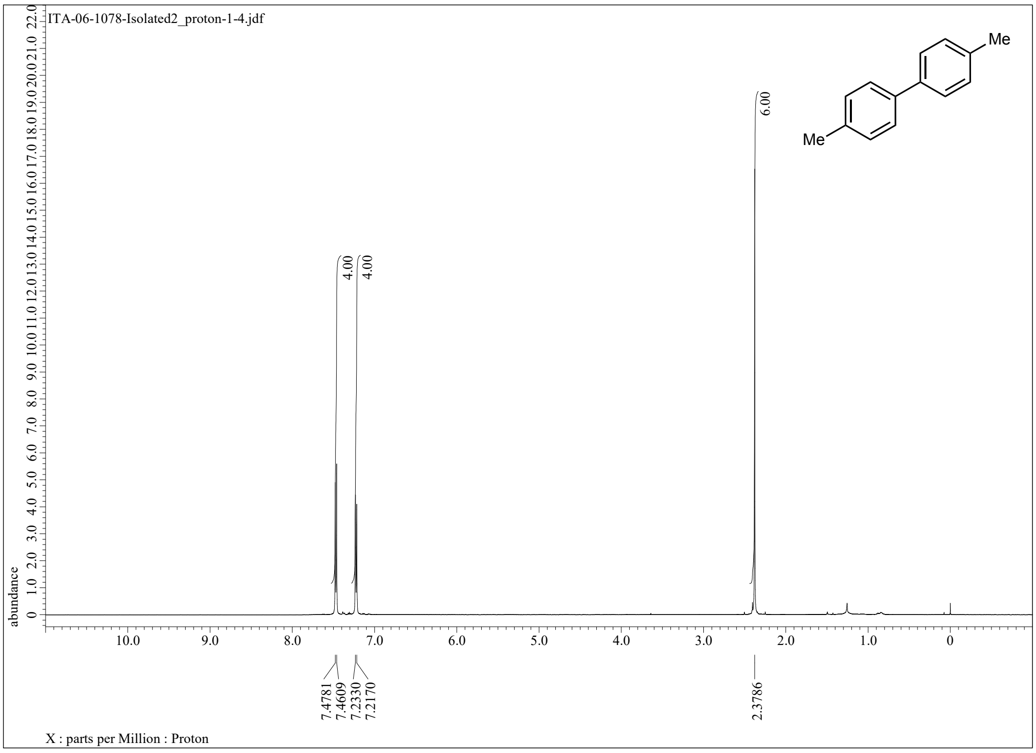

Supplementary Figure 28. ^{1}H NMR spectrum of **2** (500 MHz, $CDCl_3$).

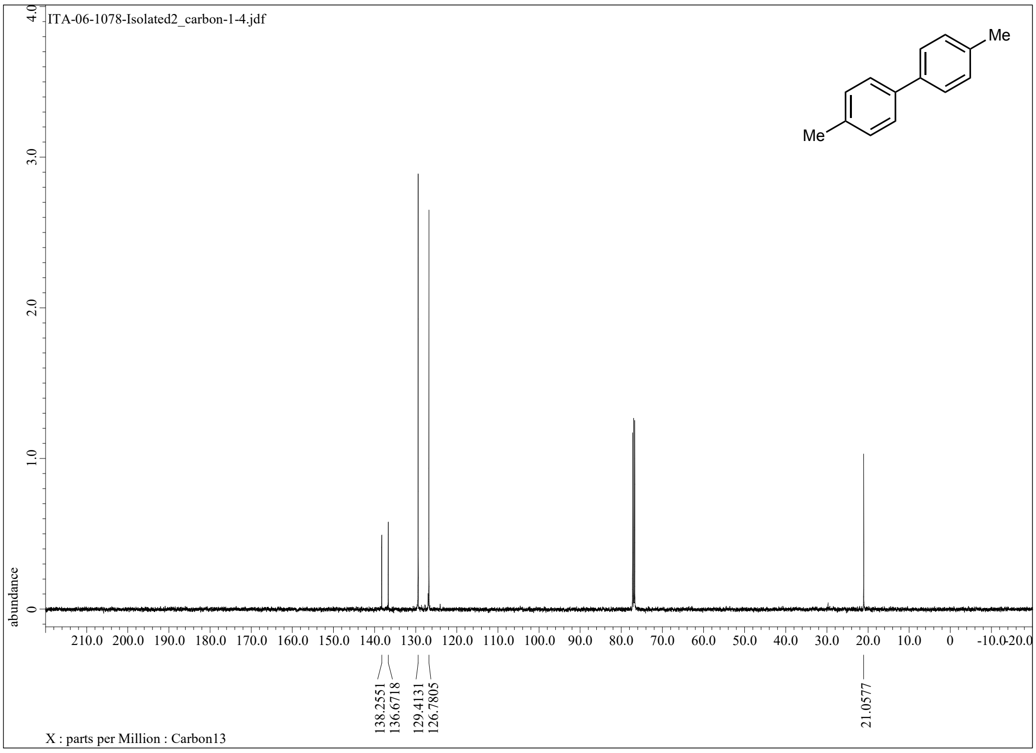

Supplementary Figure 29. ^{13}C NMR spectrum of **2** (126 MHz, $CDCl_3$).

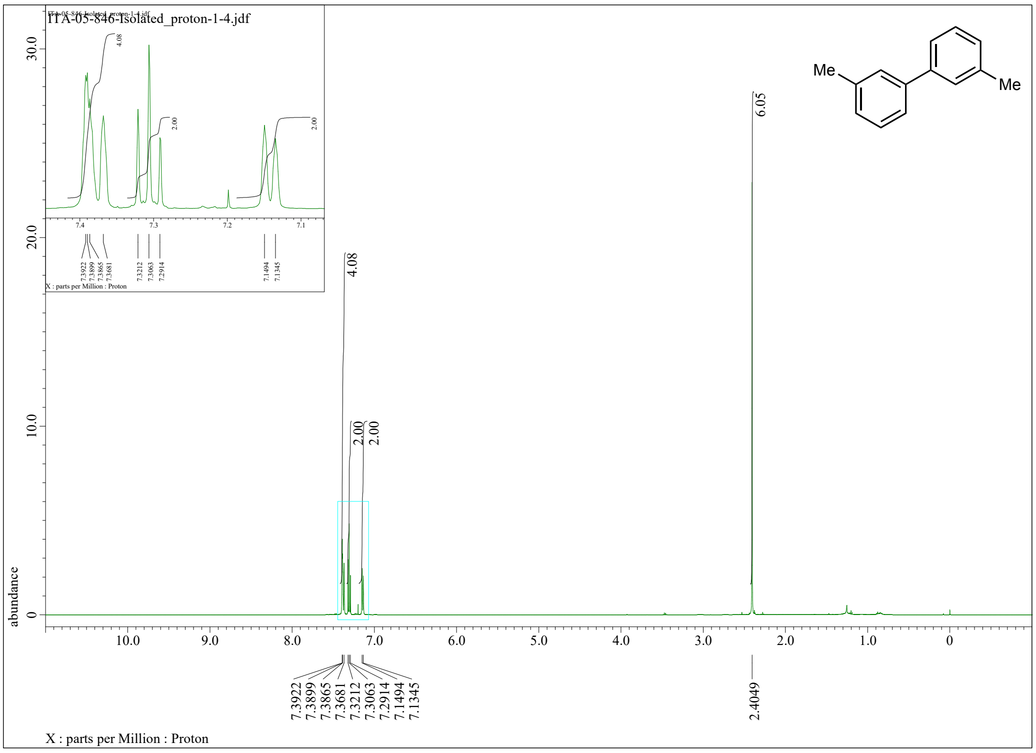

Supplementary Figure 30. ^{1}H NMR spectrum of **3** (500 MHz, $CDCl_3$).

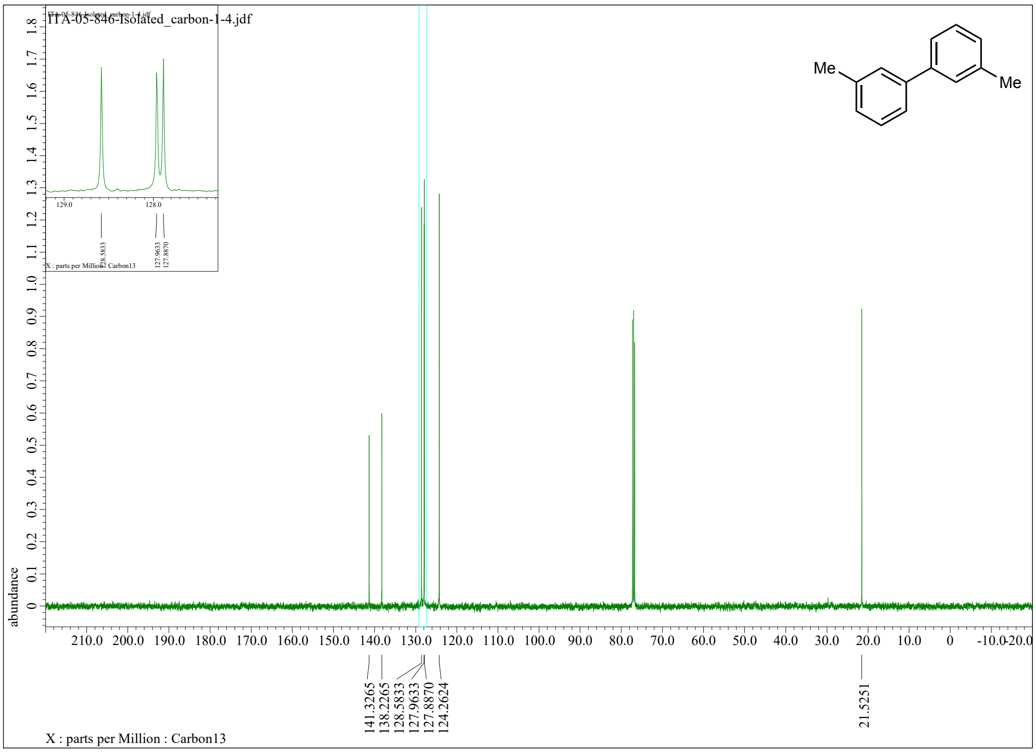

Supplementary Figure 31. ^{13}C NMR spectrum of **3** (126 MHz, $CDCl_3$).

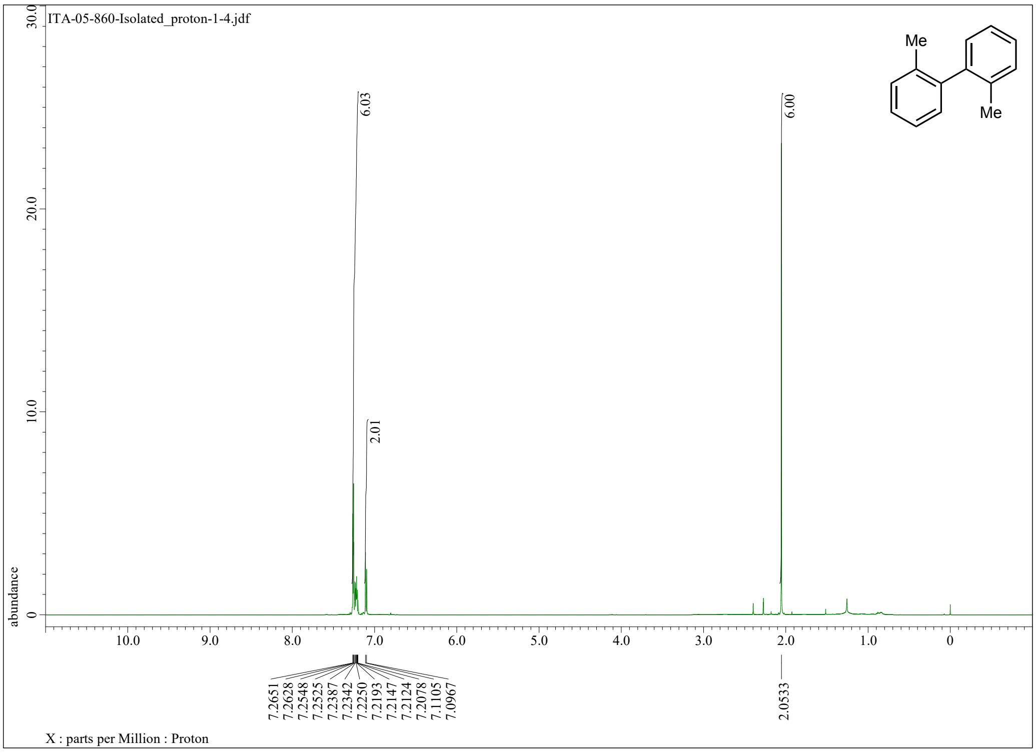

Supplementary Figure 32. ^{1}H NMR spectrum of **4** (500 MHz, $CDCl_3$).

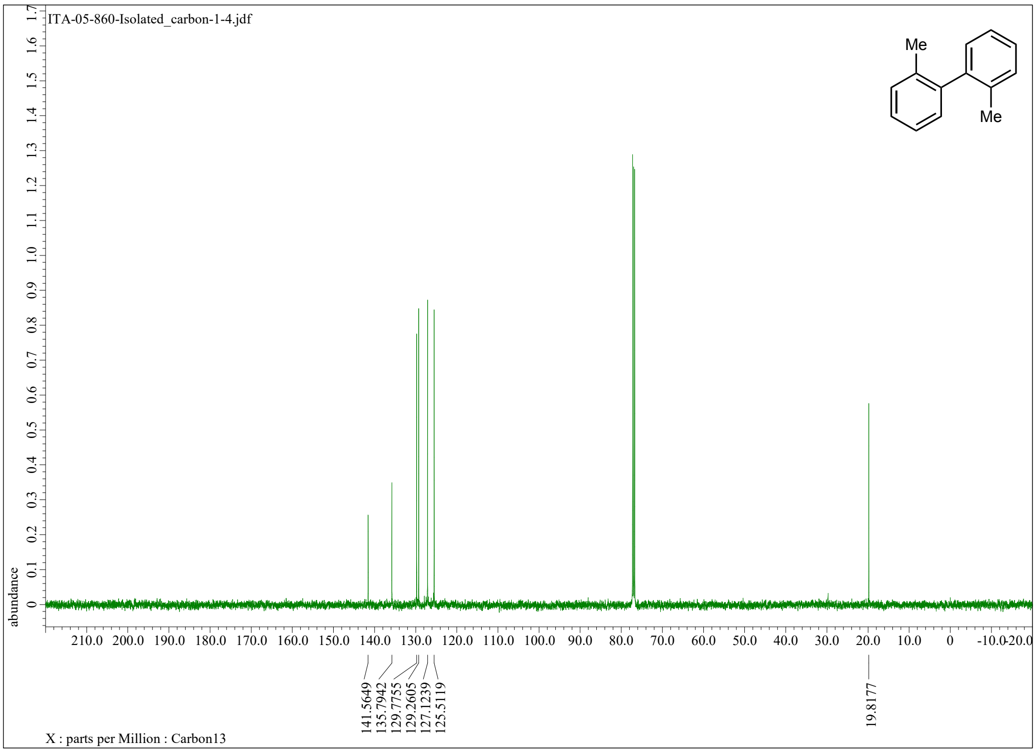

Supplementary Figure 33. ^{13}C NMR spectrum of **4** (126 MHz, $CDCl_3$).

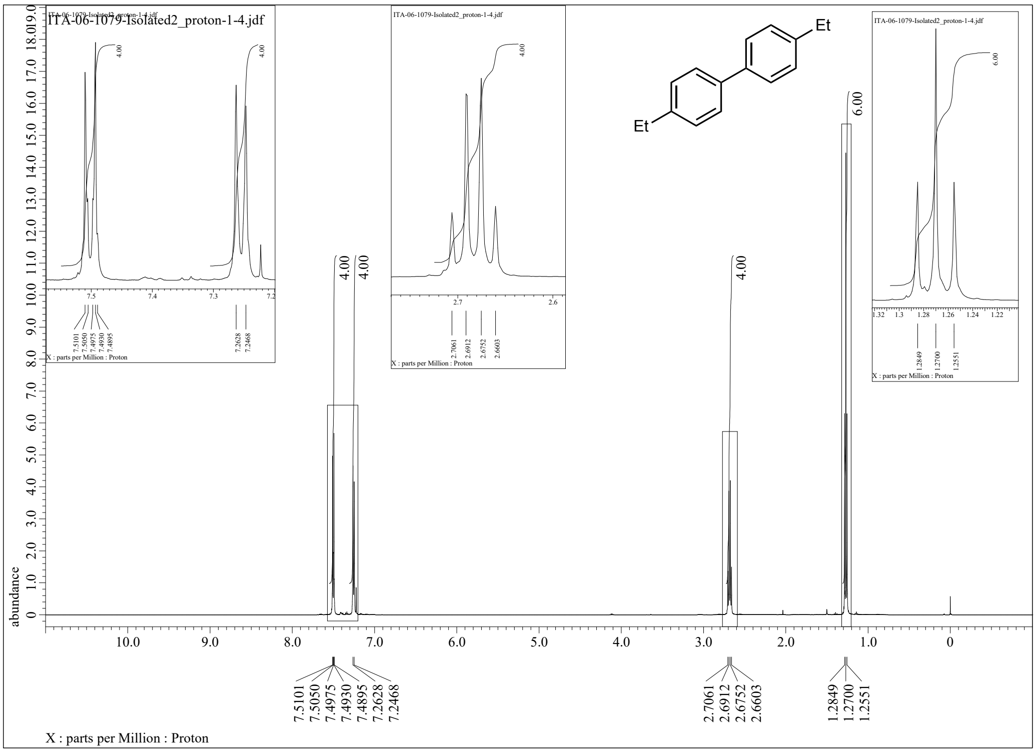

Supplementary Figure 34. ^{1}H NMR spectrum of **5** (500 MHz, $CDCl_3$).

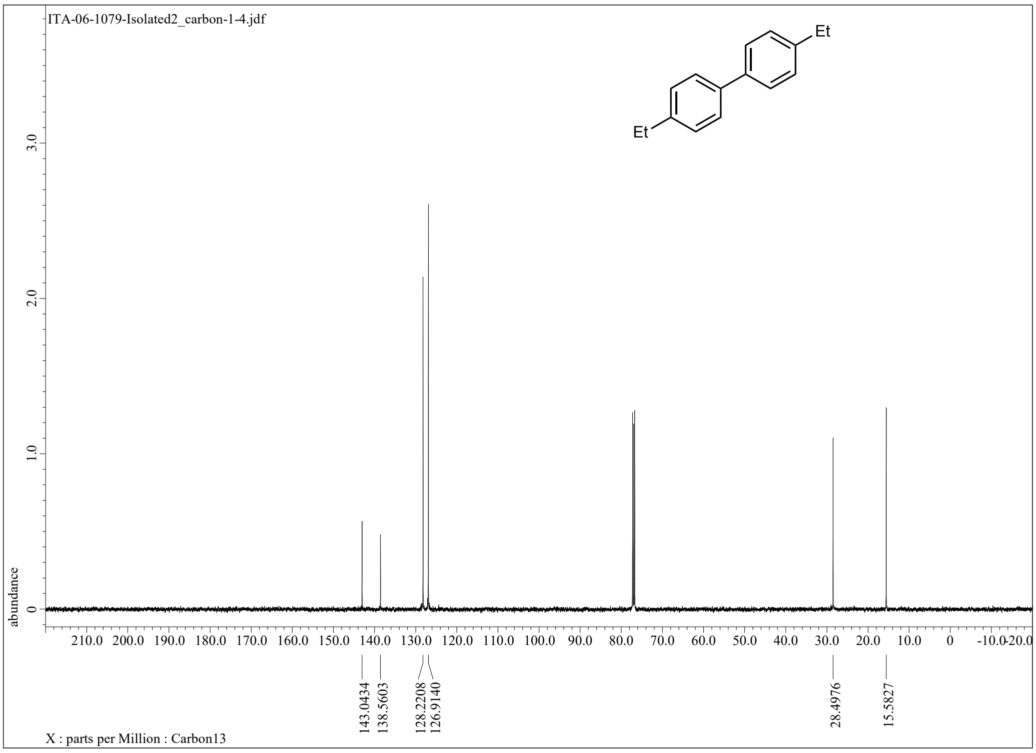

Supplementary Figure 35. ^{13}C NMR spectrum of **5** (126 MHz, $CDCl_3$).

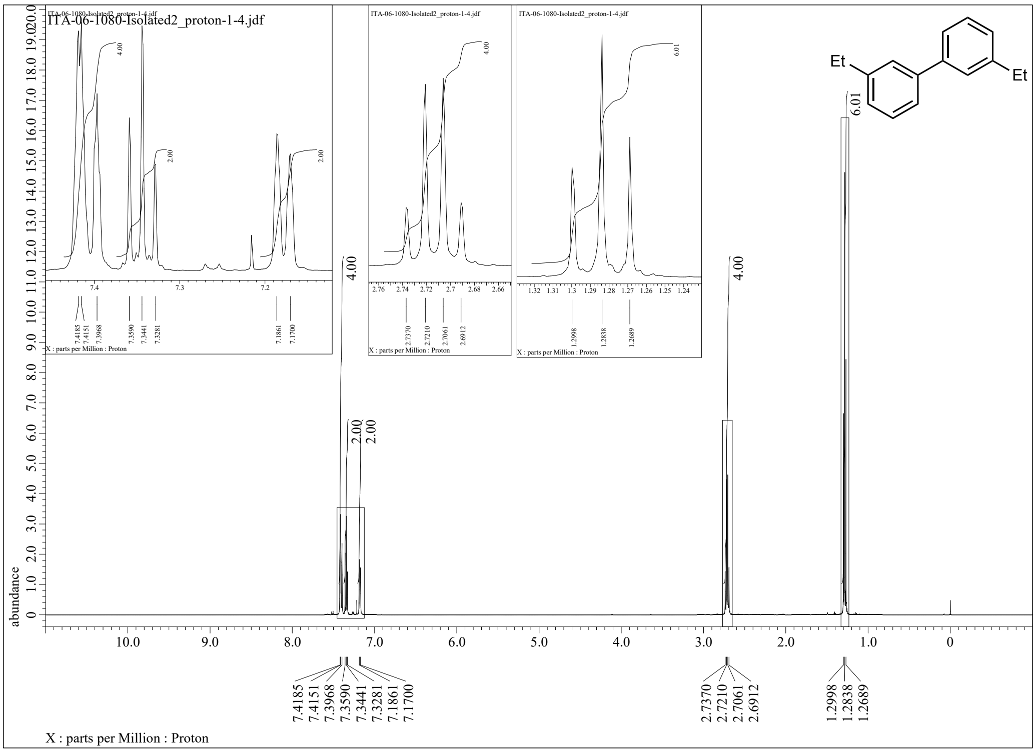

Supplementary Figure 36. ^{1}H NMR spectrum of **6** (500 MHz, $CDCl_3$).

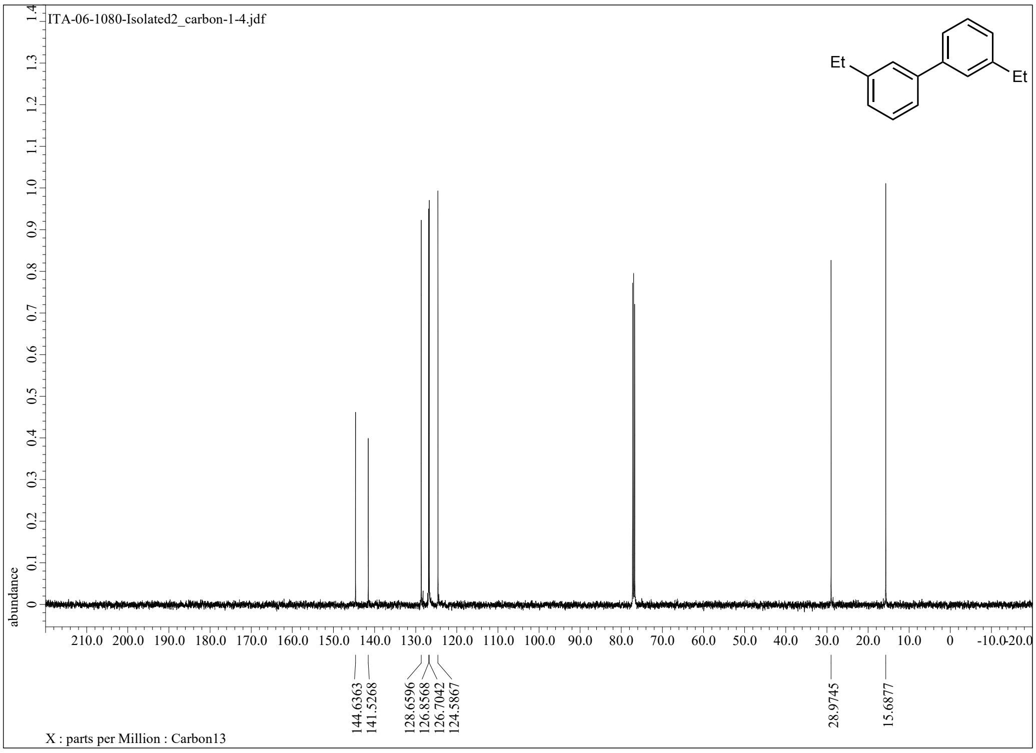

Supplementary Figure 37. ^{13}C NMR spectrum of **6** (126 MHz, $CDCl_3$).

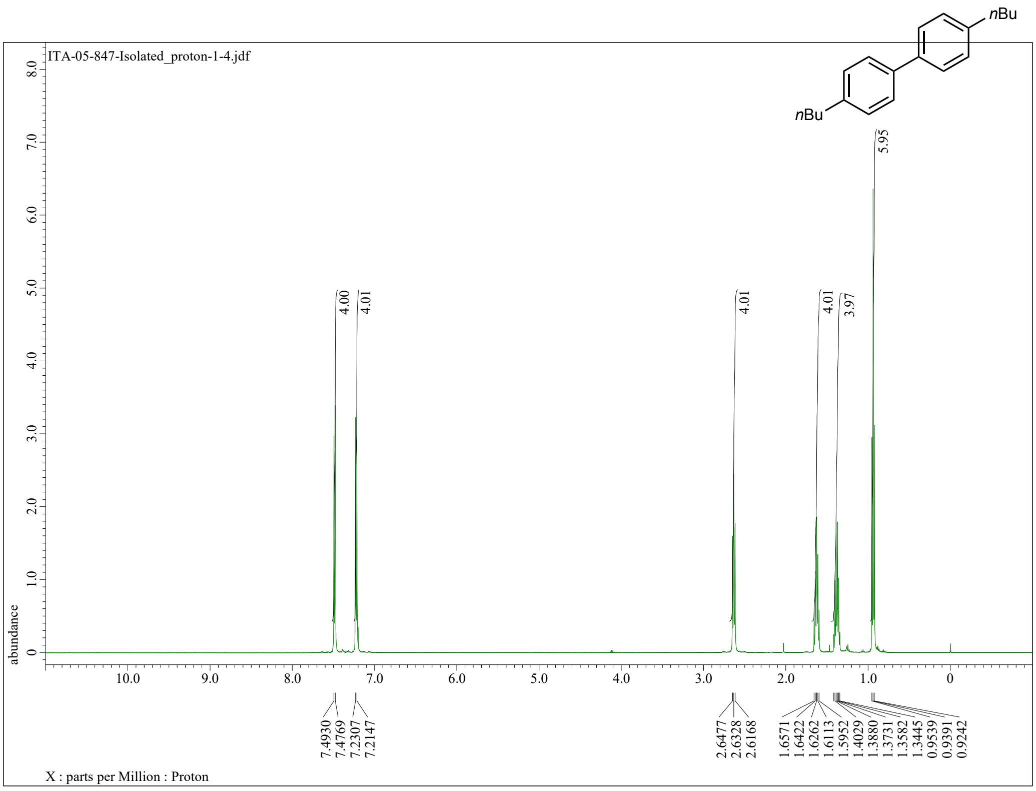

Supplementary Figure 38. ^{1}H NMR spectrum of **7** (500 MHz, $CDCl_3$).

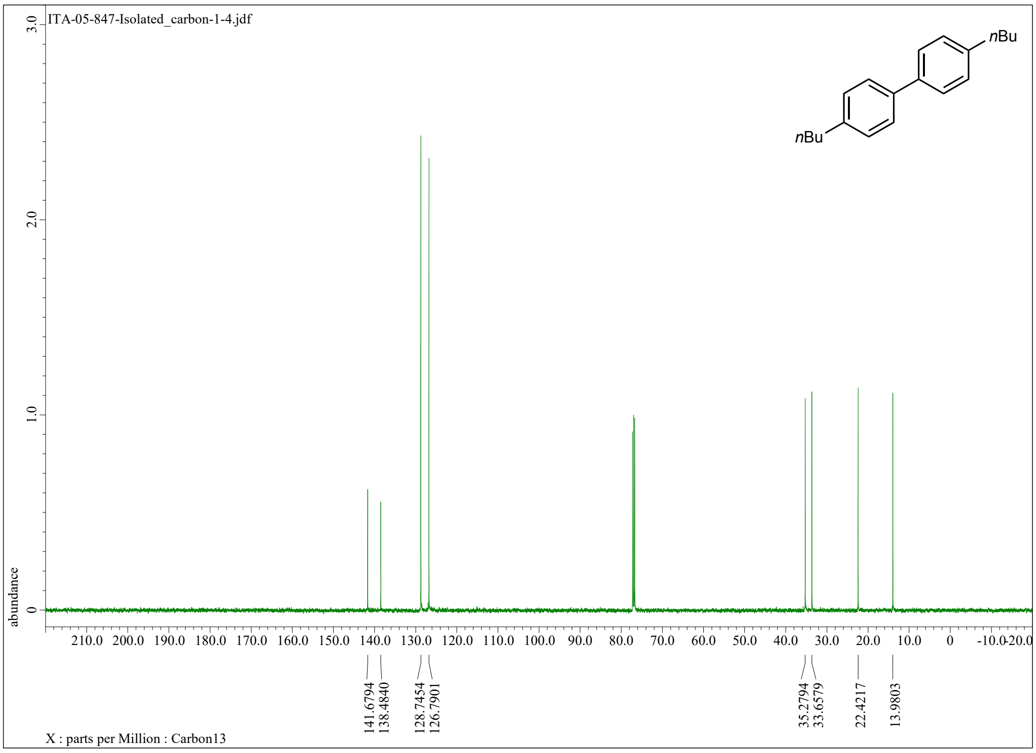

Supplementary Figure 39. ^{13}C NMR spectrum of **7** (126 MHz, $CDCl_3$).

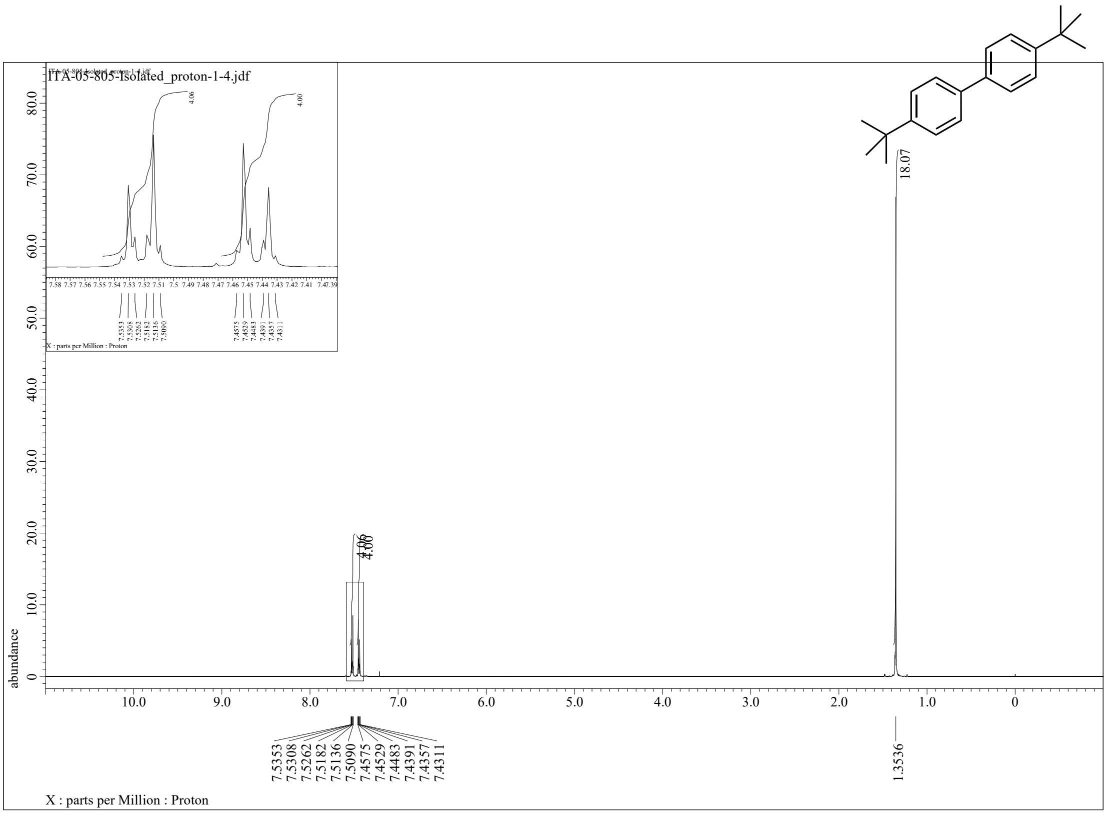

Supplementary Figure 40. ^{1}H NMR spectrum of **8** (500 MHz, $CDCl_3$).

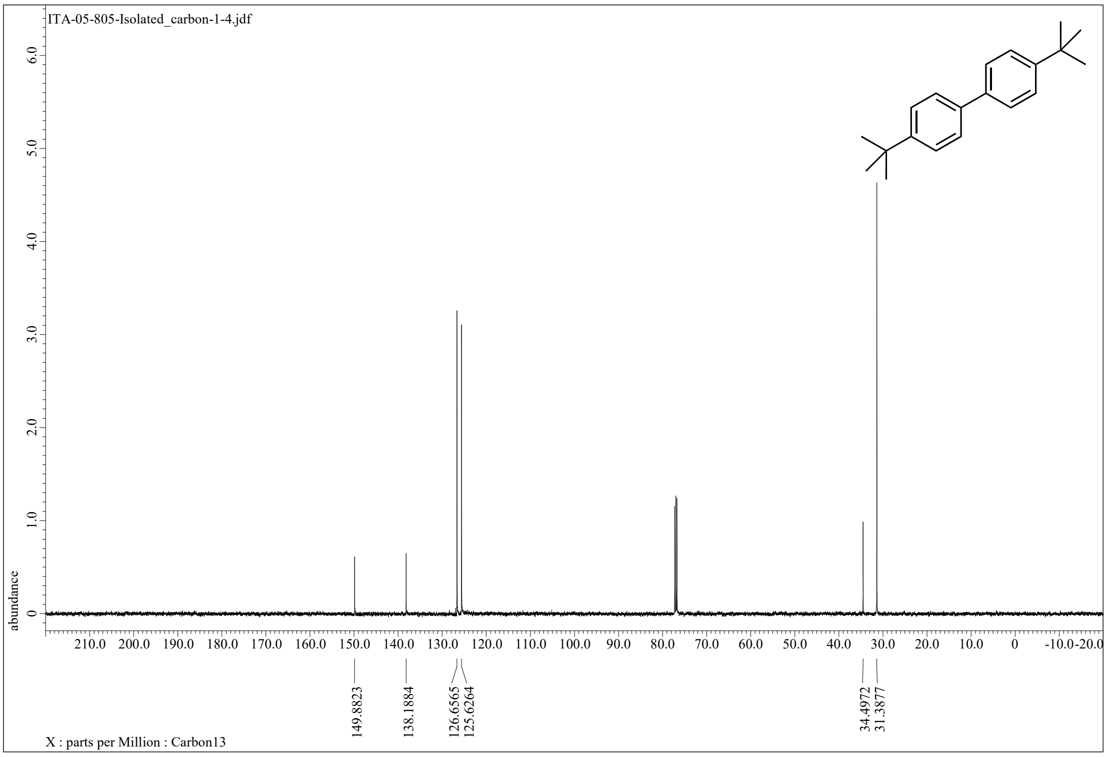

Supplementary Figure 41. ^{13}C NMR spectrum of **8** (126 MHz, $CDCl_3$).

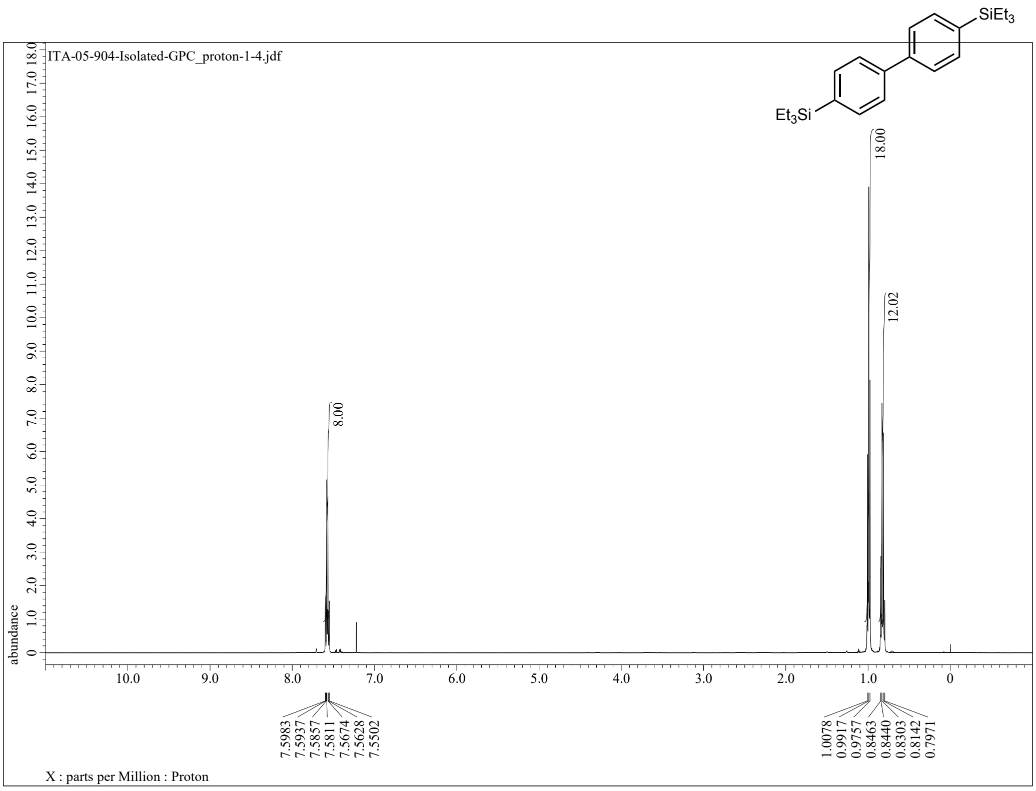

Supplementary Figure 42. ^{1}H NMR spectrum of **9** (500 MHz, $CDCl_3$).

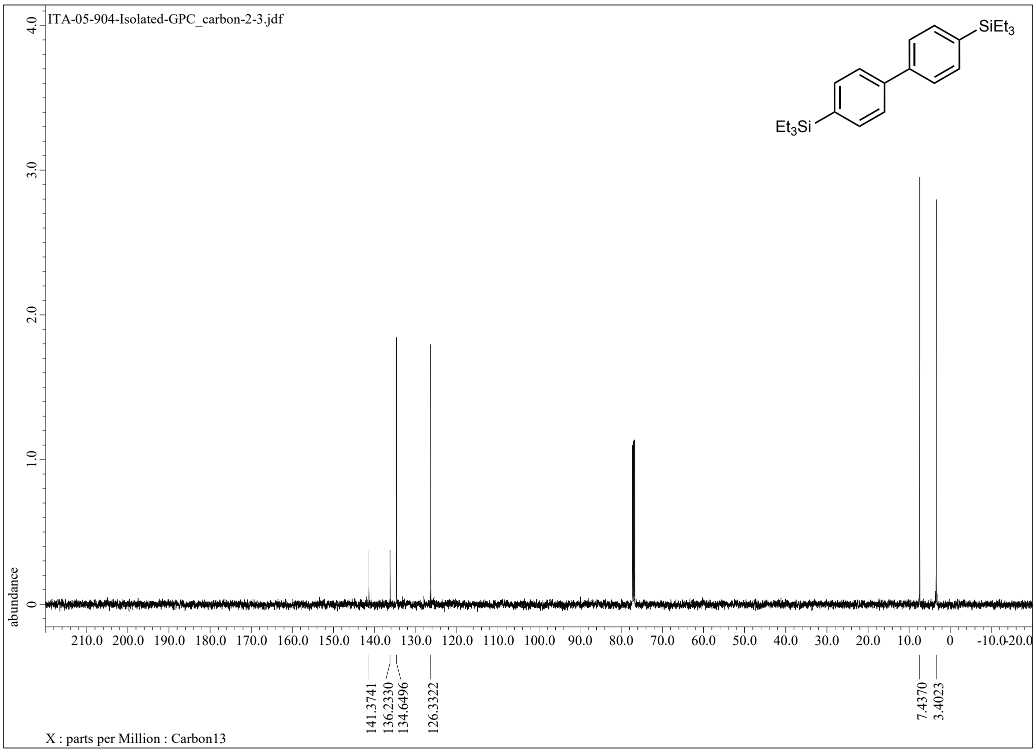

Supplementary Figure 43. ^{13}C NMR spectrum of **9** (126 MHz, $CDCl_3$).

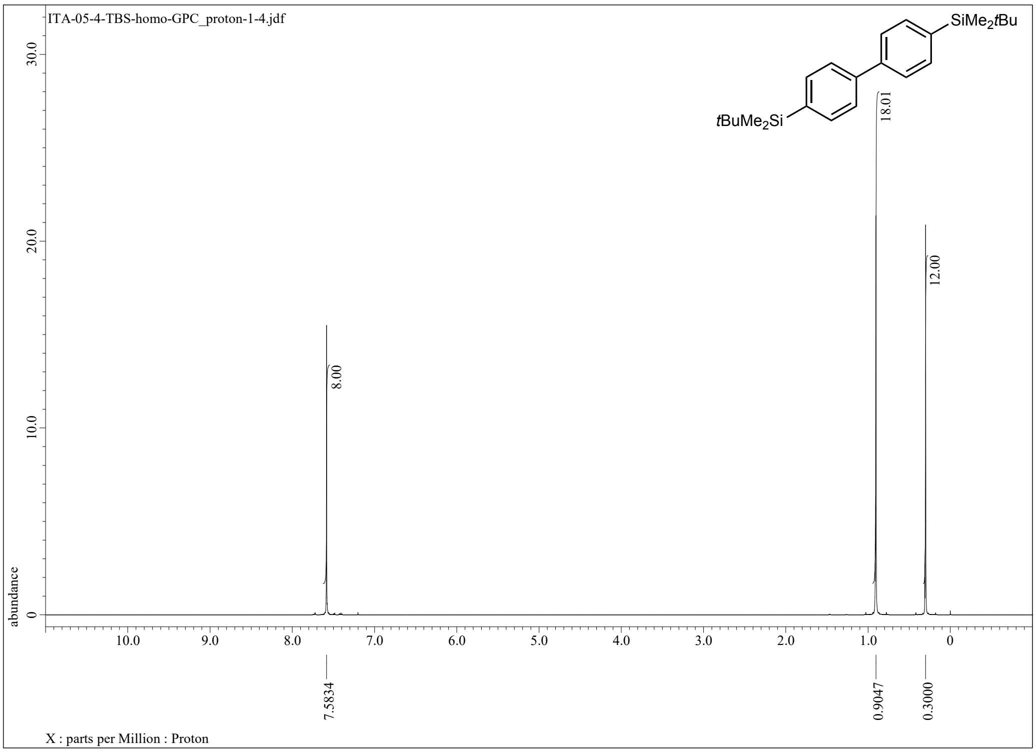

Supplementary Figure 44. ^{1}H NMR spectrum of **10** (500 MHz, $CDCl_3$).

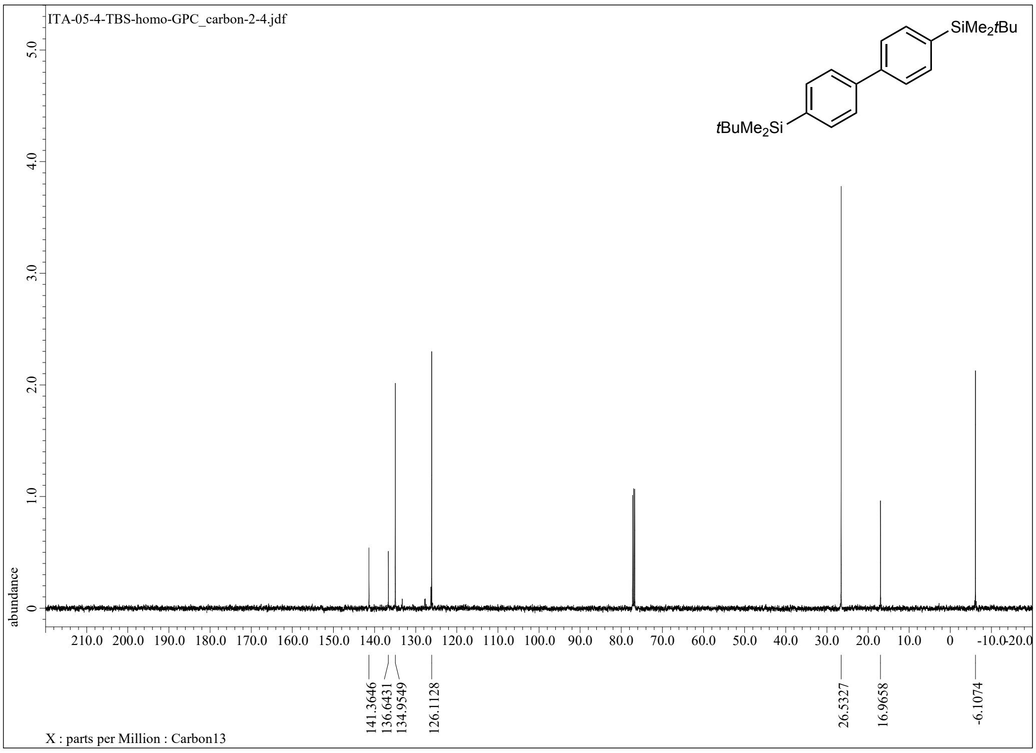

Supplementary Figure 45. ^{13}C NMR spectrum of **10** (126 MHz, $CDCl_3$).

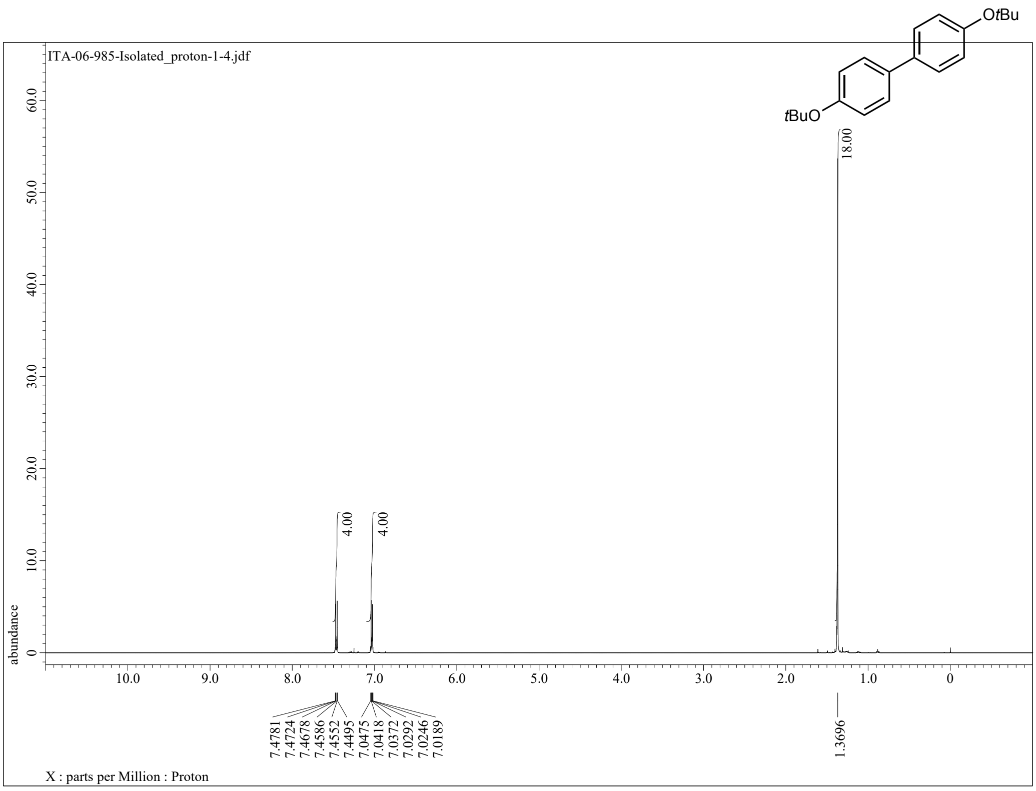

Supplementary Figure 46. ^{1}H NMR spectrum of **11** (500 MHz, $CDCl_3$).

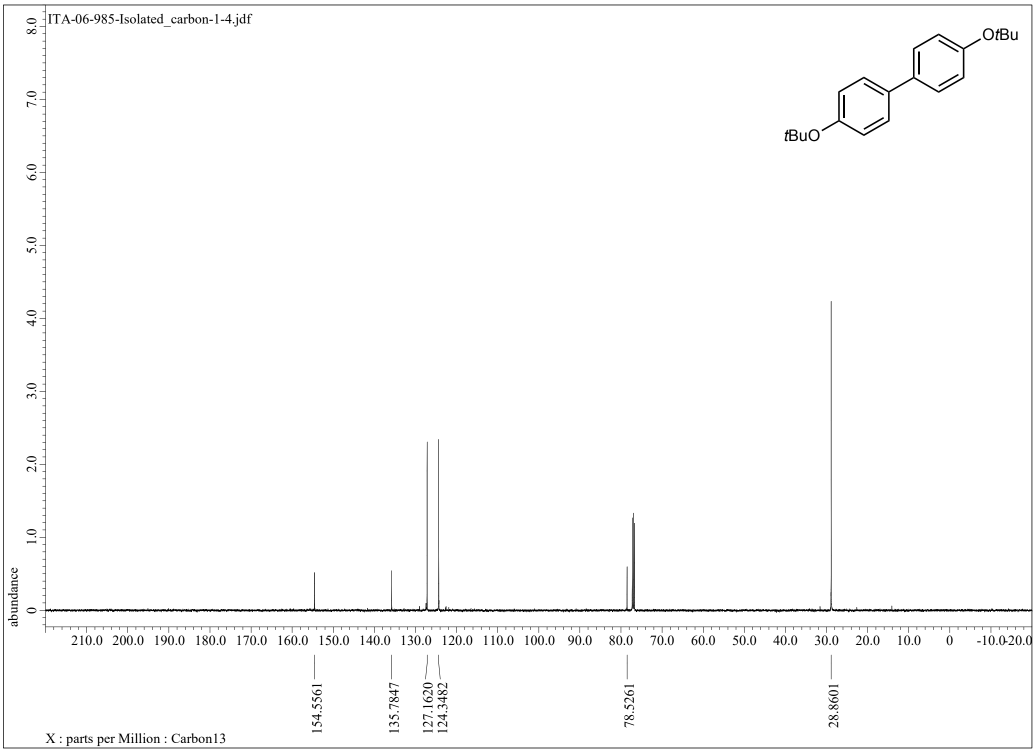

Supplementary Figure 47. ^{13}C NMR spectrum of **11** (126 MHz, $CDCl_3$).

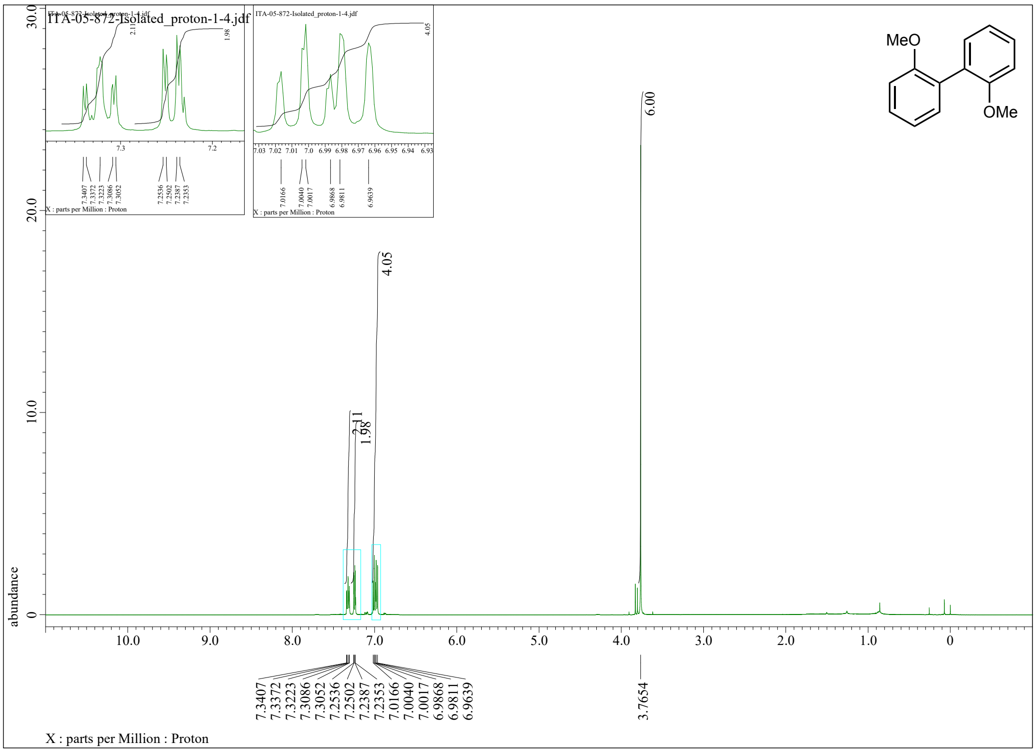

Supplementary Figure 48. ^{1}H NMR spectrum of **12** (500 MHz, $CDCl_3$).

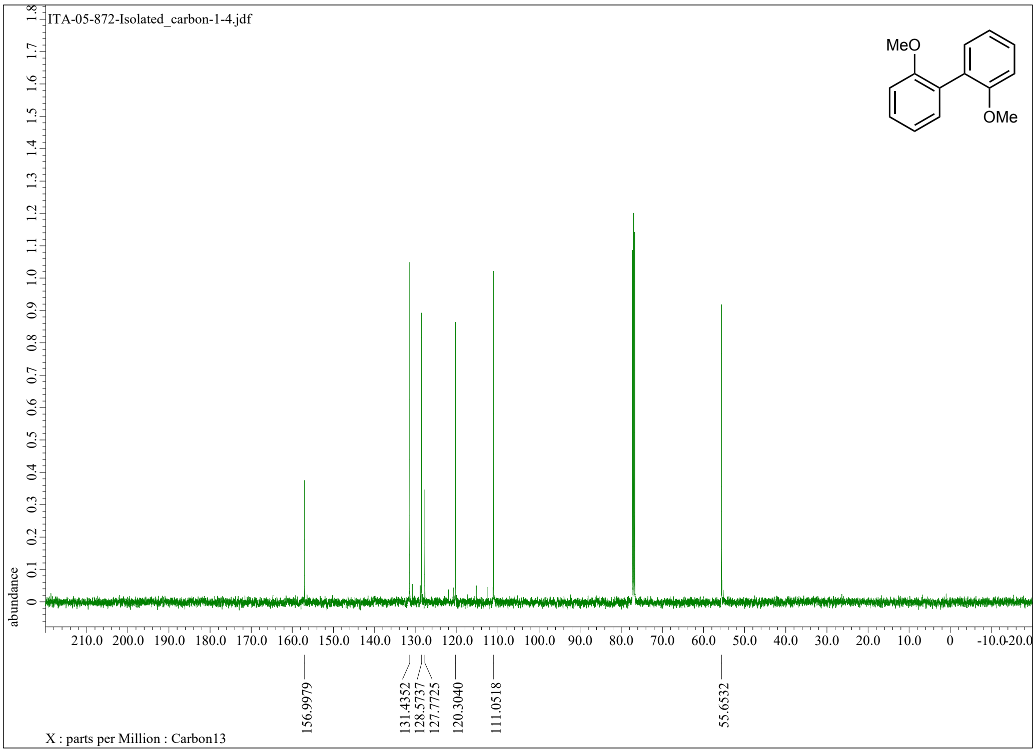

Supplementary Figure 49. ^{13}C NMR spectrum of **12** (126 MHz, $CDCl_3$).

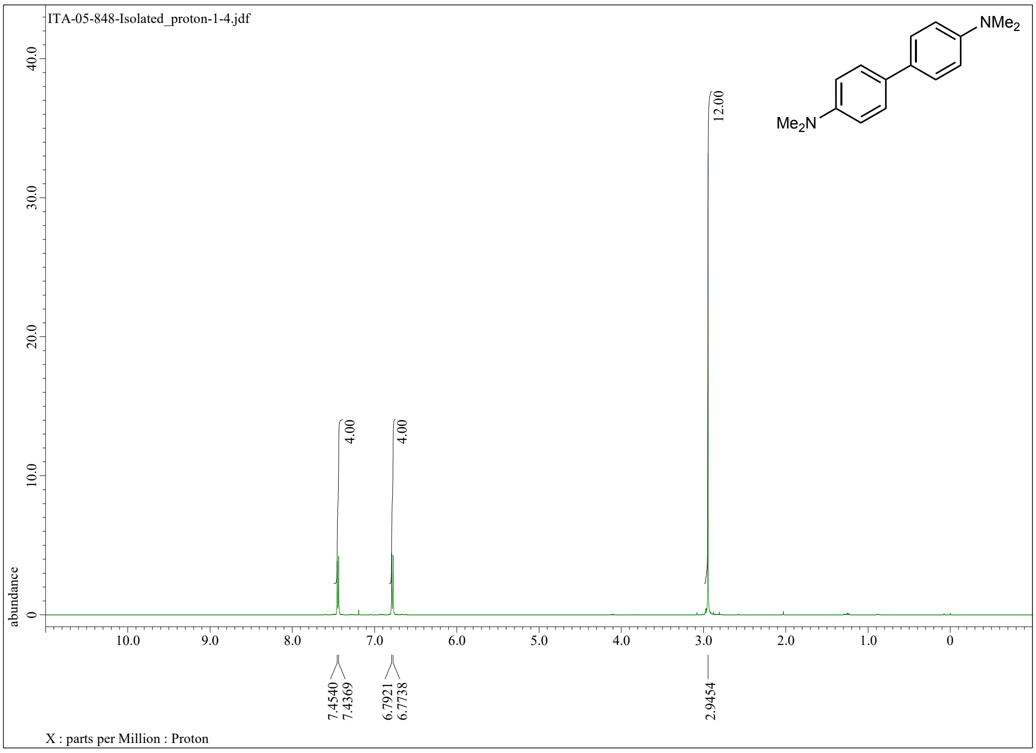

Supplementary Figure 50. ^{1}H NMR spectrum of **13** (500 MHz, $CDCl_3$).

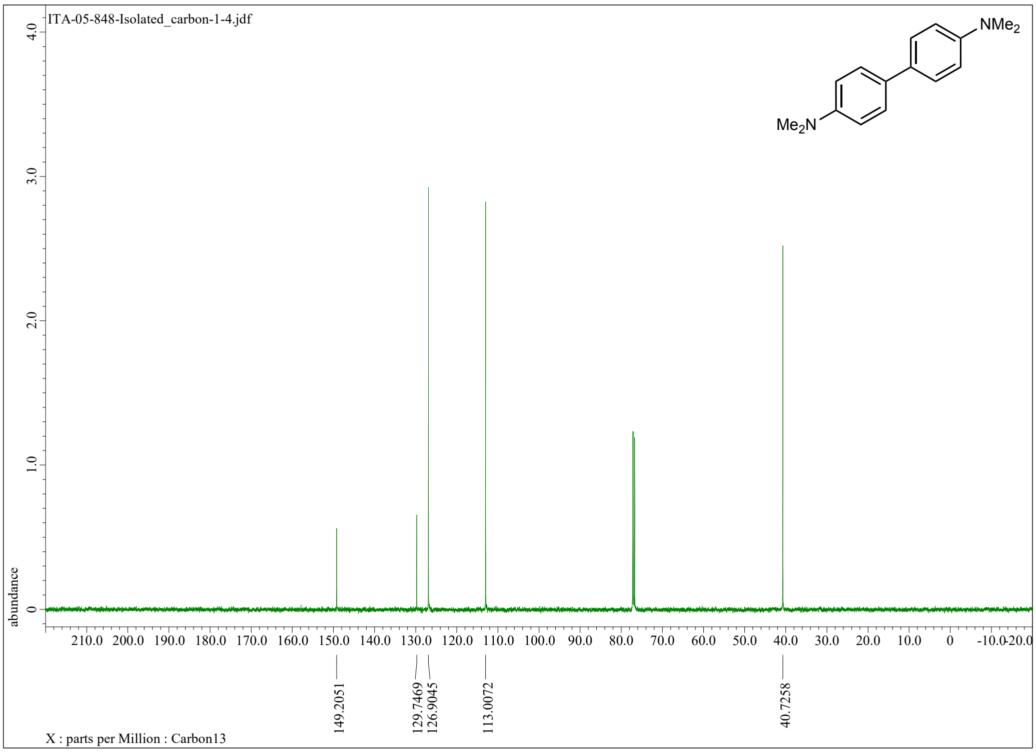

Supplementary Figure 51. ^{13}C NMR spectrum of **13** (126 MHz, $CDCl_3$).

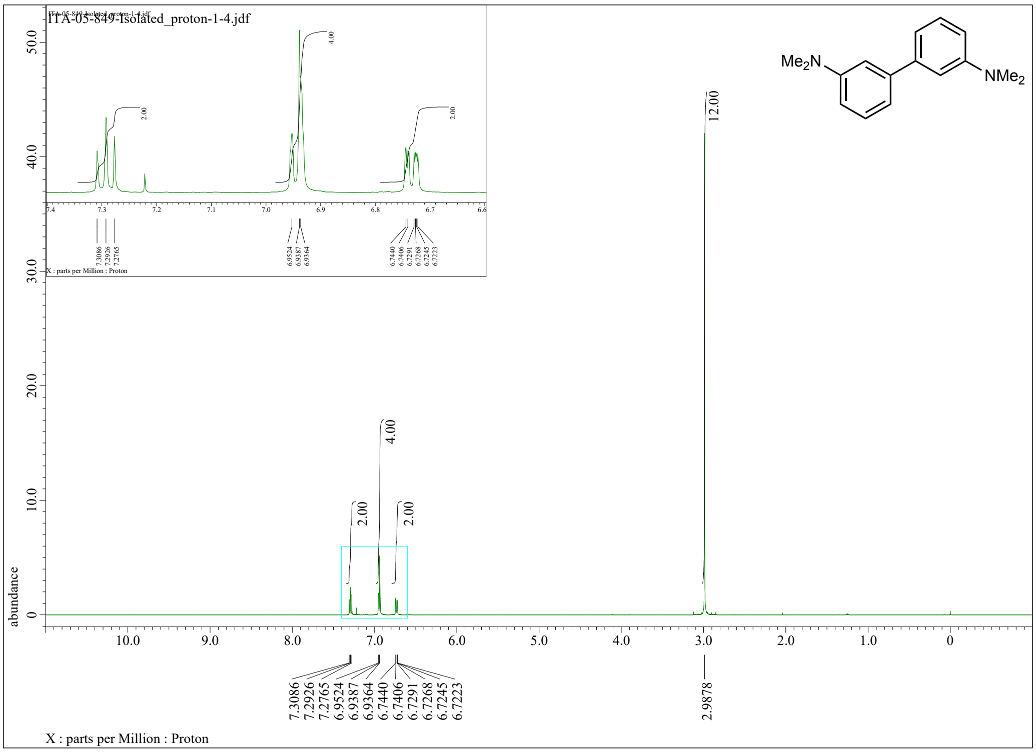

Supplementary Figure 52. ^{1}H NMR spectrum of **14** (500 MHz, $CDCl_3$).

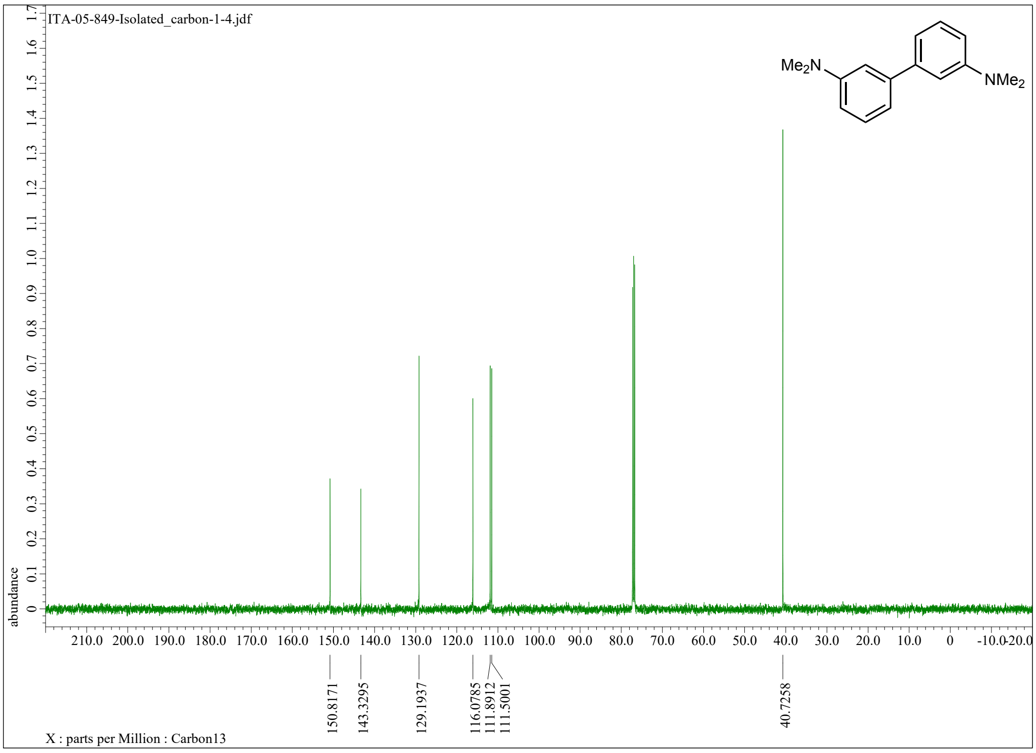

Supplementary Figure 53. ^{13}C NMR spectrum of **14** (126 MHz, $CDCl_3$).

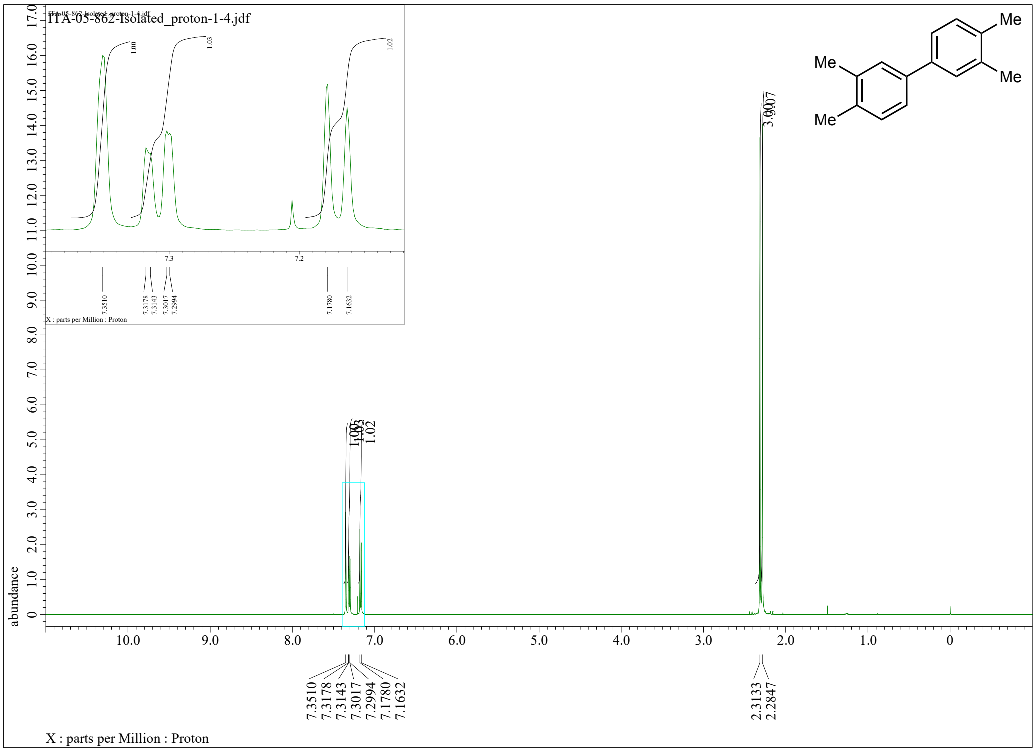

Supplementary Figure 54. ^{1}H NMR spectrum of **15** (500 MHz, $CDCl_3$).

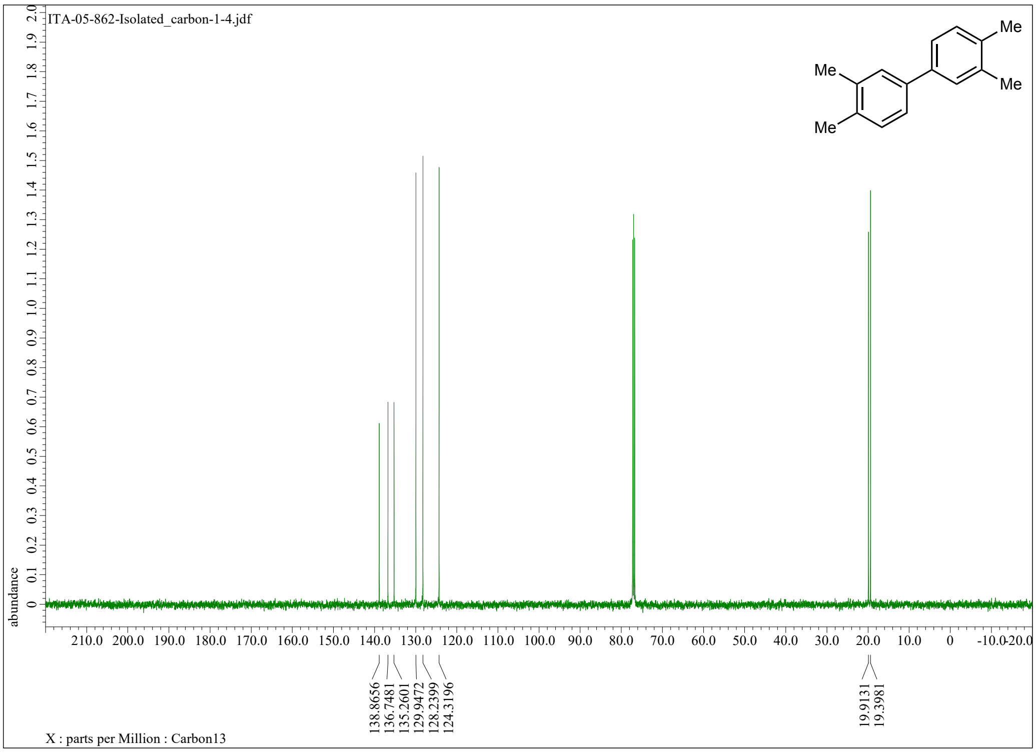

Supplementary Figure 55. ^{13}C NMR spectrum of **15** (126 MHz, $CDCl_3$).

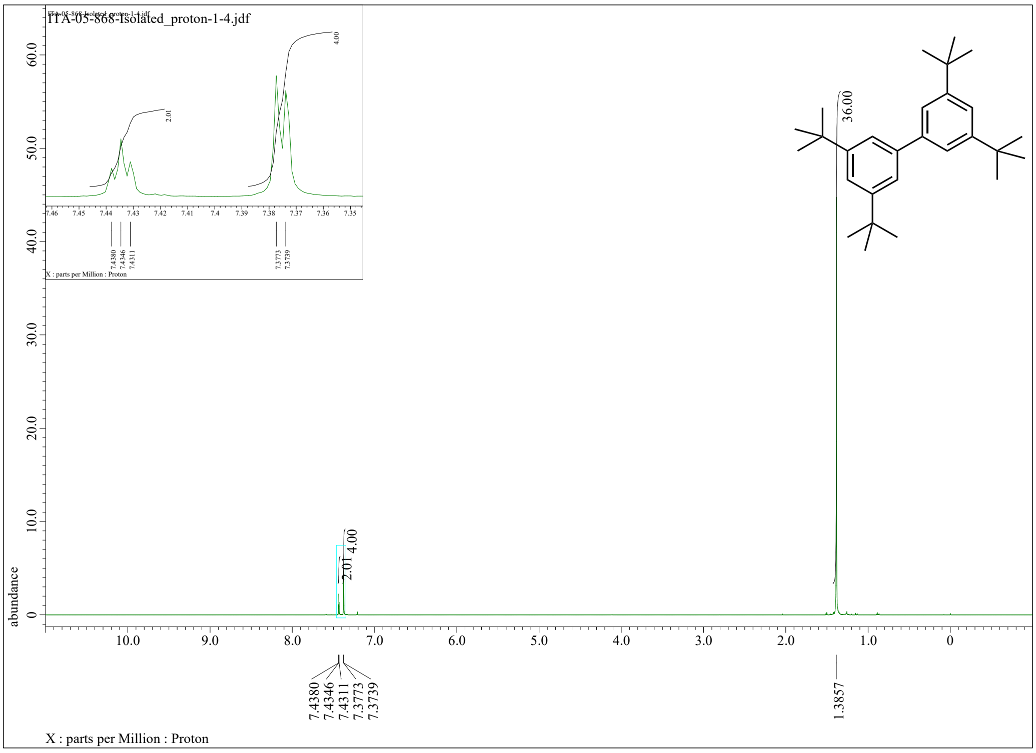

Supplementary Figure 56. ^{1}H NMR spectrum of **16** (500 MHz, $CDCl_3$).

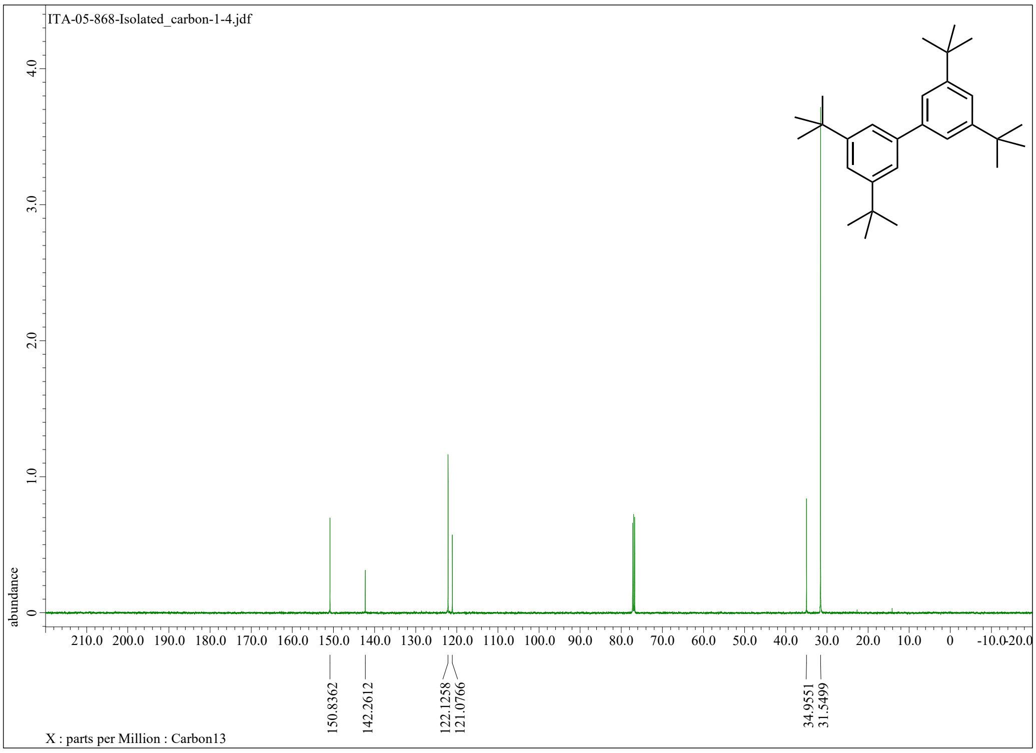

Supplementary Figure 57. ^{13}C NMR spectrum of **16** (126 MHz, $CDCl_3$).

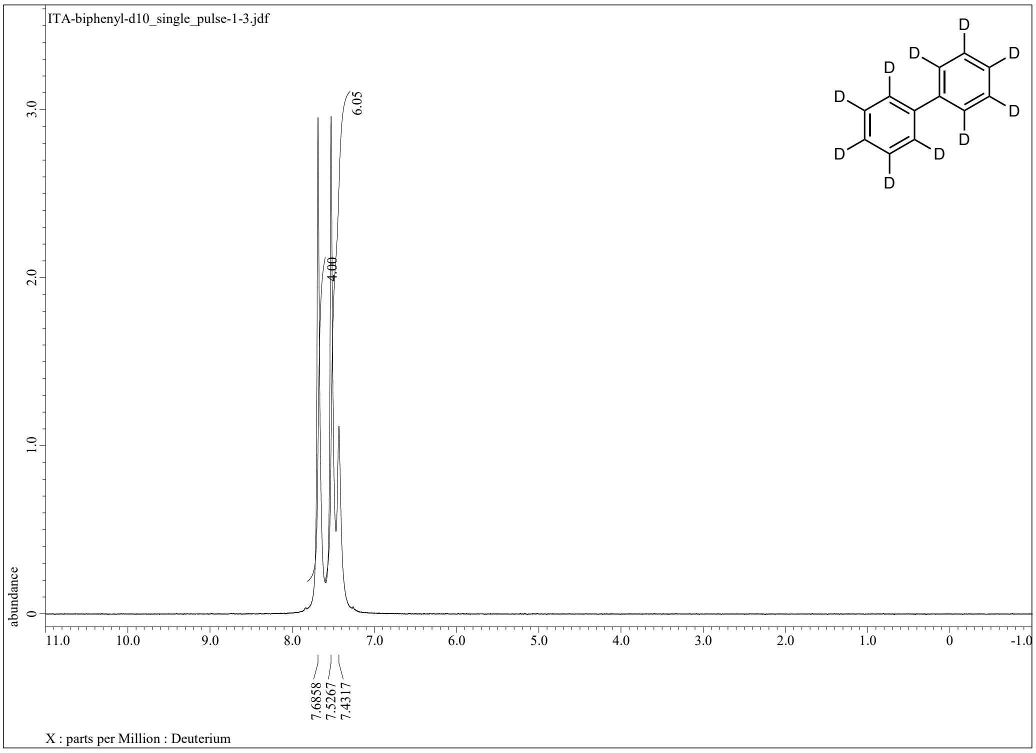

Supplementary Figure 58. ^{2}H NMR spectrum of **17** (77 MHz, $CHCl_3$).

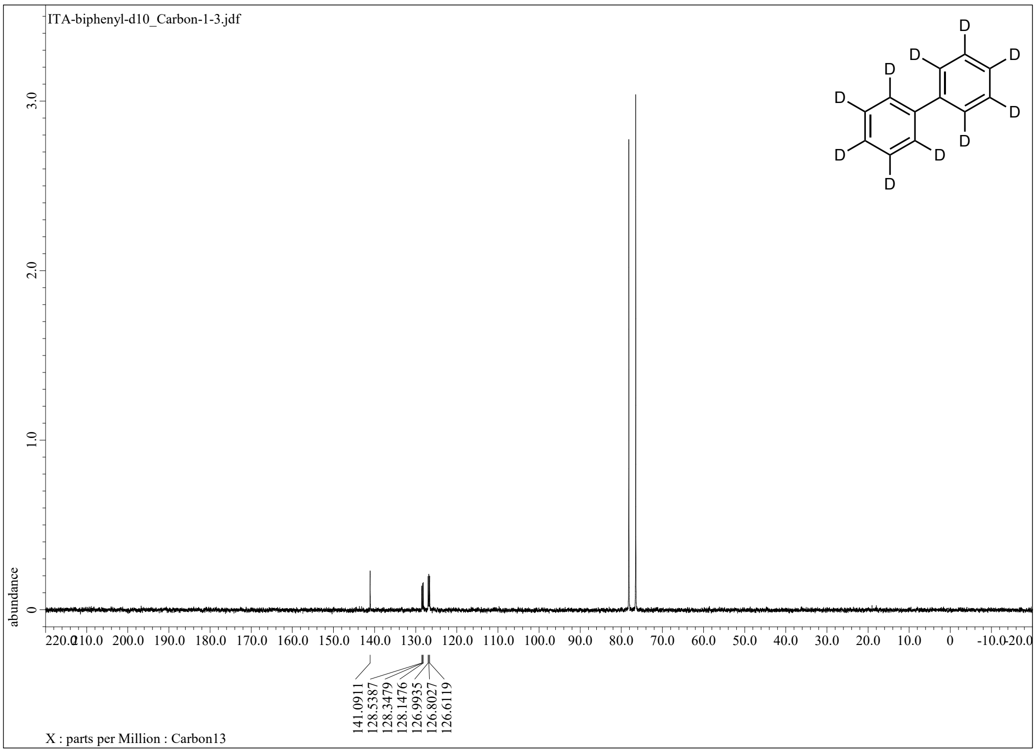

Supplementary Figure 59. ^{13}C NMR spectrum of **17** (126 MHz, $CDCl_3$).

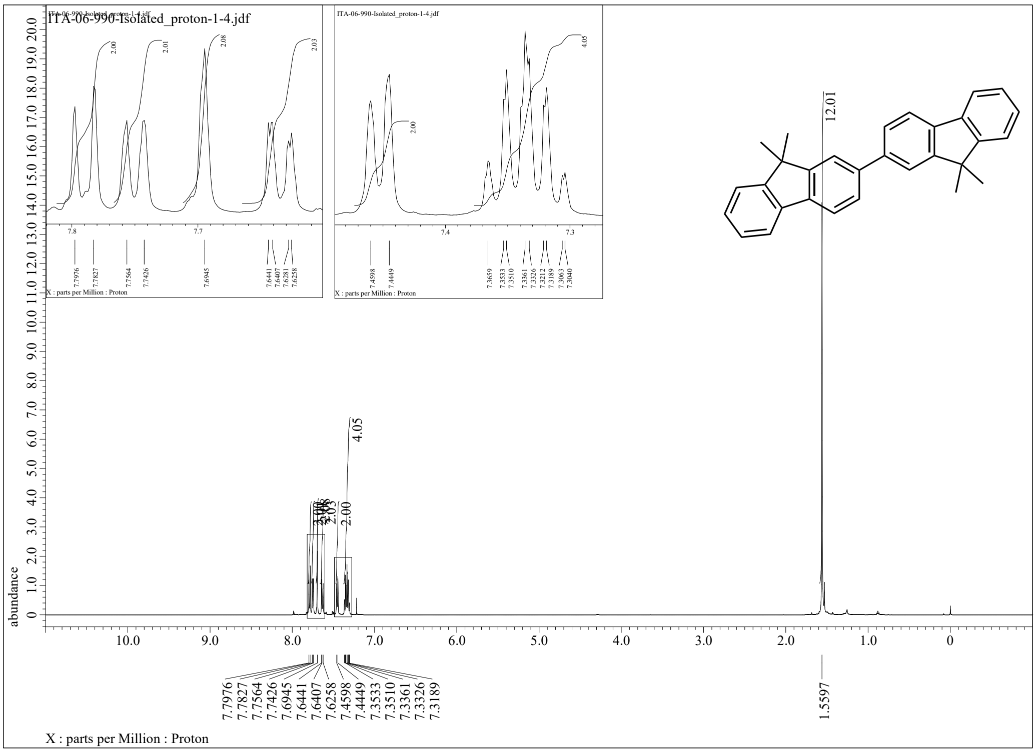

Supplementary Figure 60. ^{1}H NMR spectrum of **18** (500 MHz, $CDCl_3$).

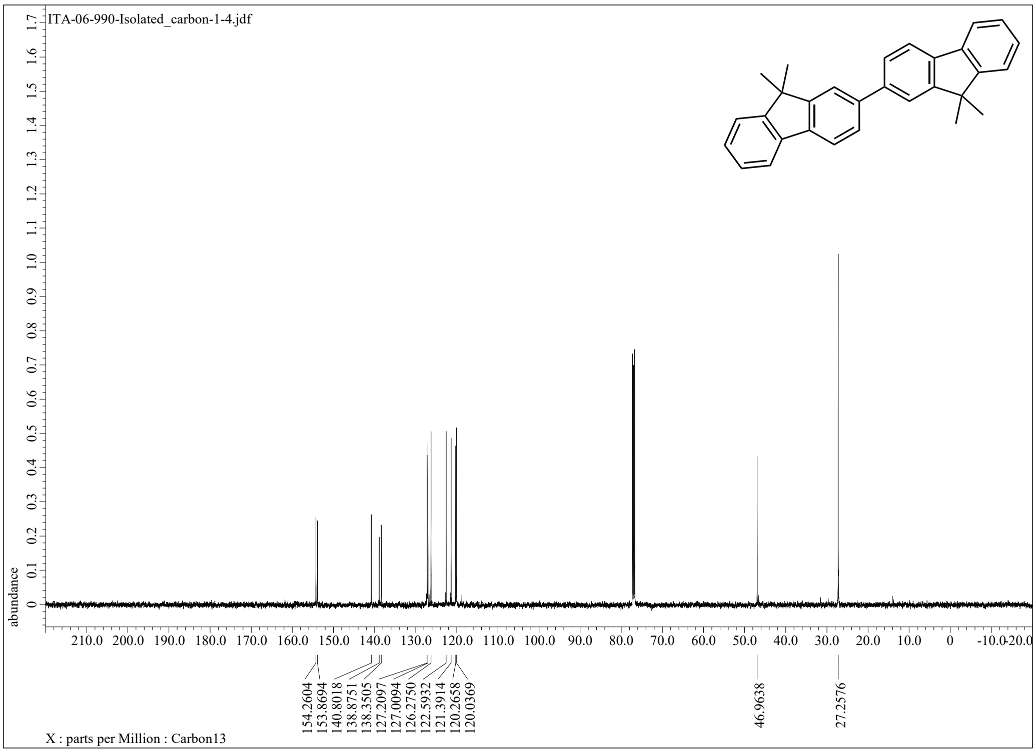

Supplementary Figure 61. ^{13}C NMR spectrum of **18** (126 MHz, $CDCl_3$).

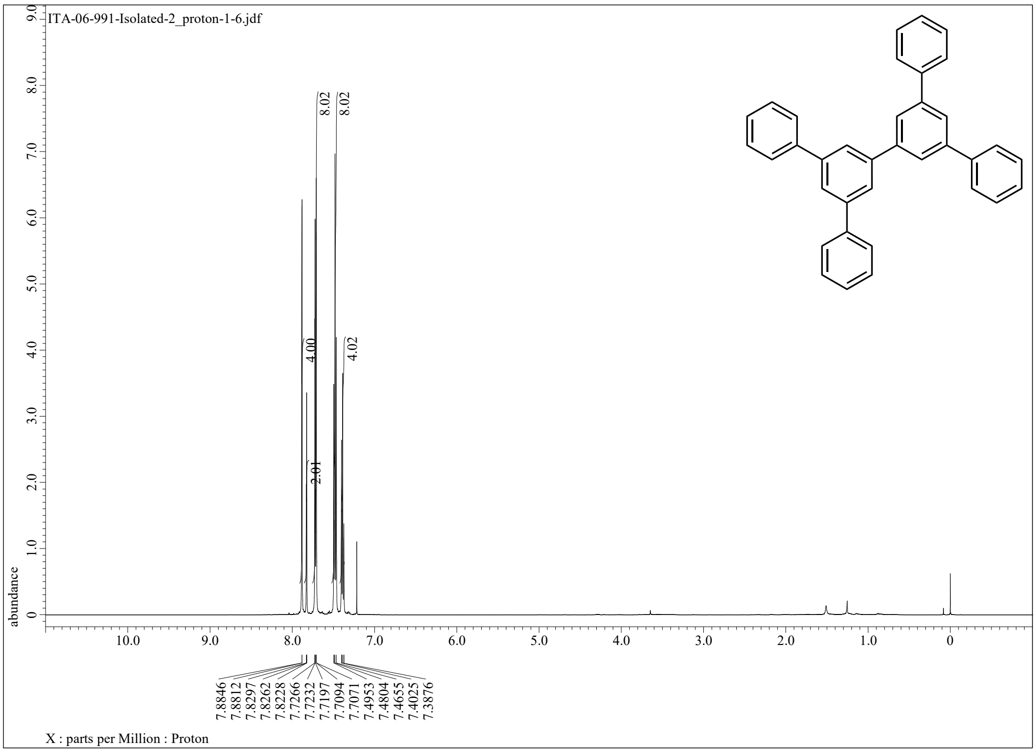

Supplementary Figure 62. ^{1}H NMR spectrum of **19** (500 MHz, $CDCl_3$).

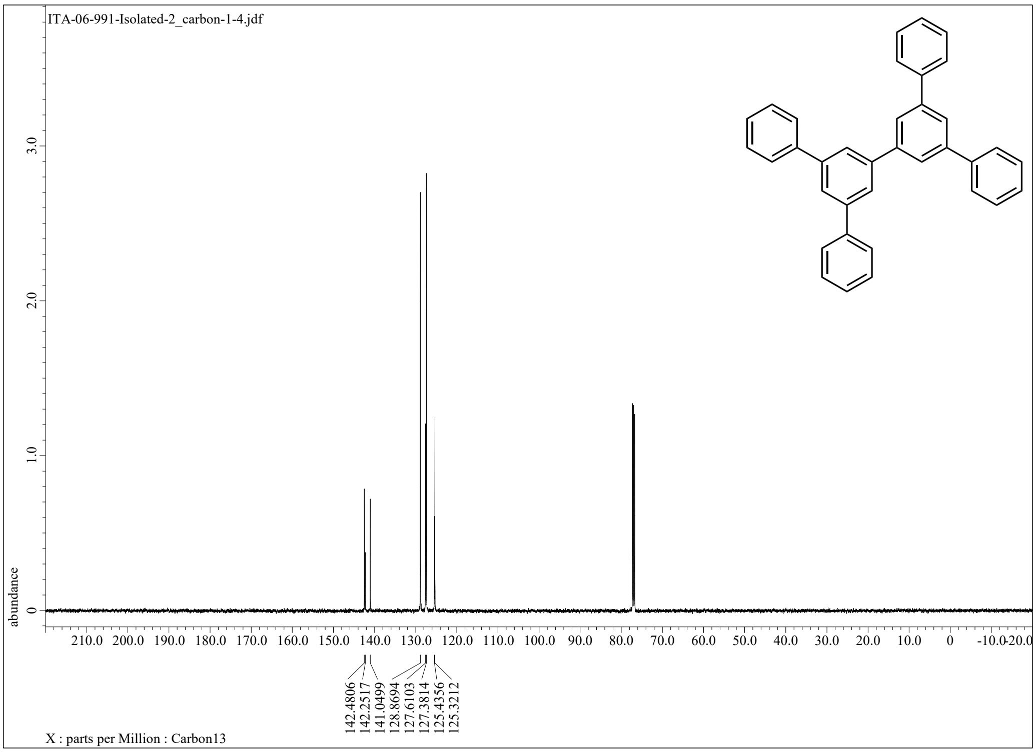

Supplementary Figure 63. ^{13}C NMR spectrum of **19** (126 MHz, $CDCl_3$).

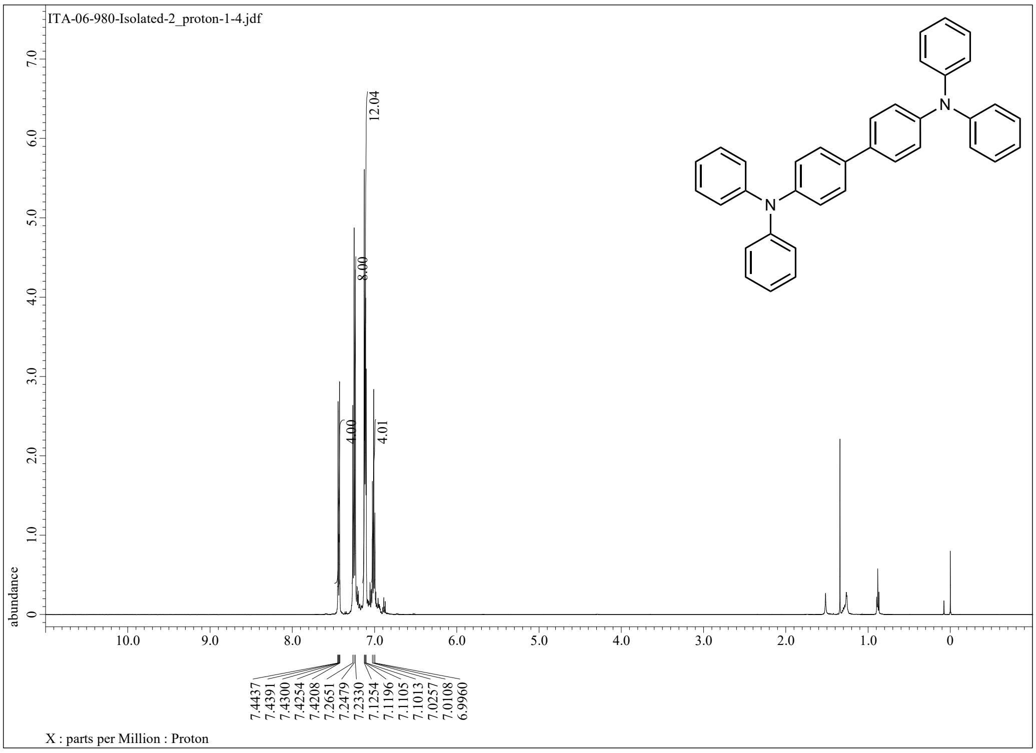

Supplementary Figure 64. ^{1}H NMR spectrum of **20** (500 MHz, $CDCl_3$).

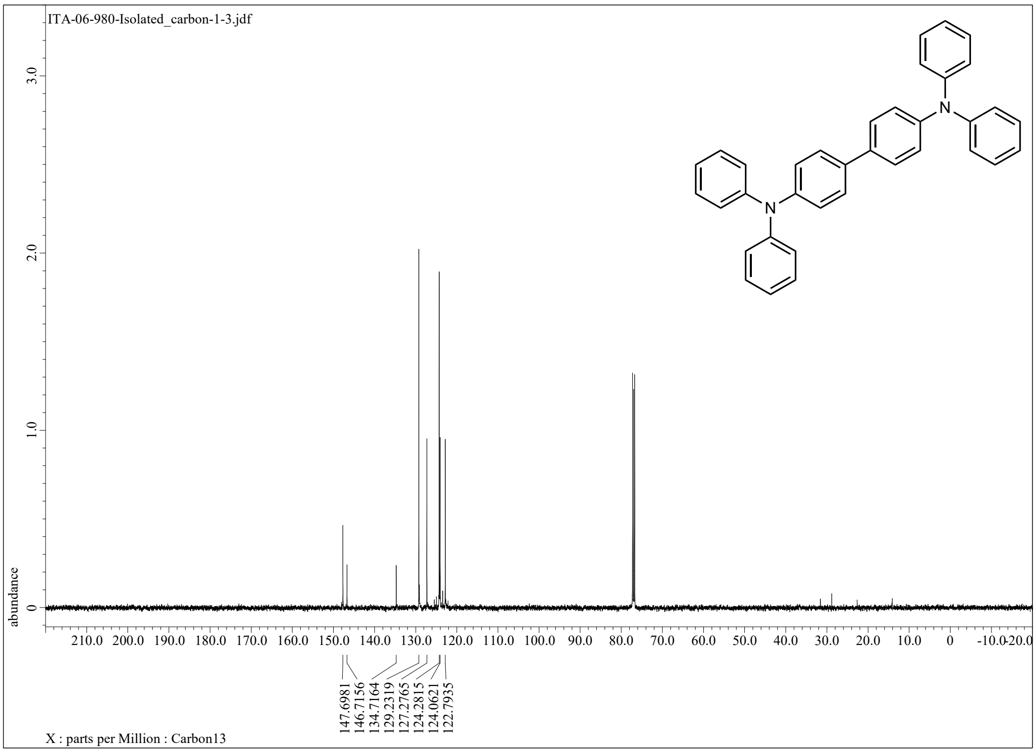

Supplementary Figure 65. ^{13}C NMR spectrum of **20** (126 MHz, $CDCl_3$).

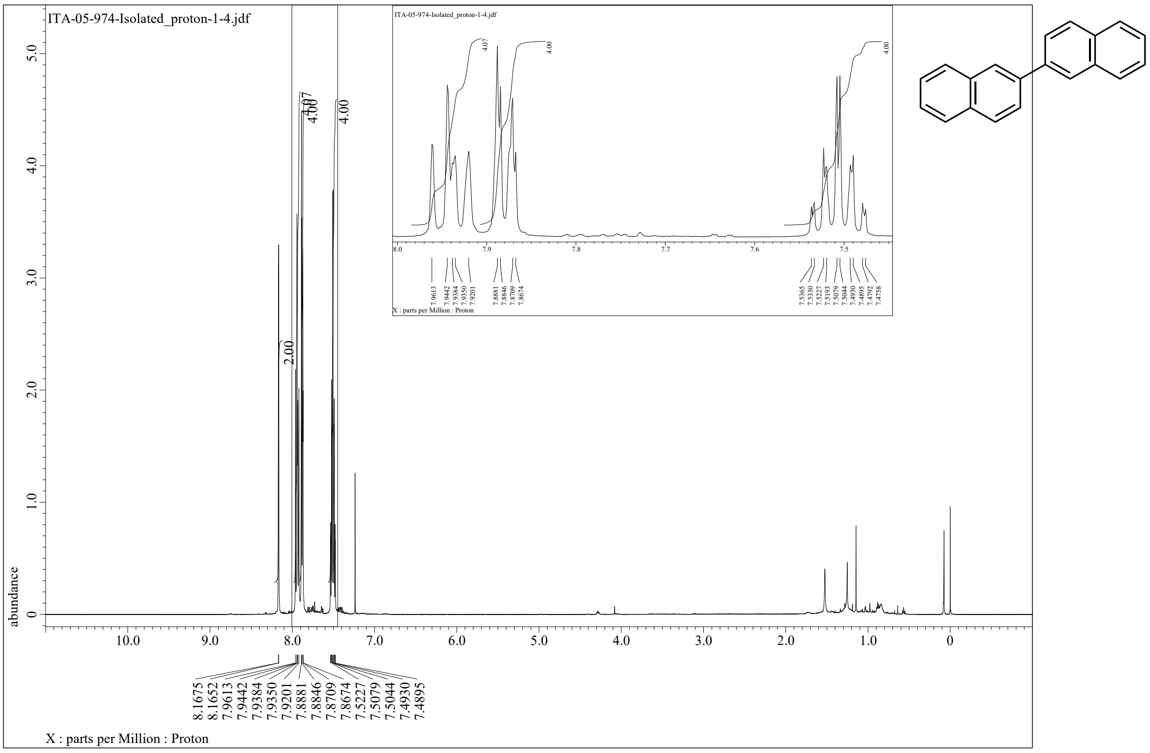

Supplementary Figure 66. ^{1}H NMR spectrum of **21** (500 MHz, $CDCl_3$).

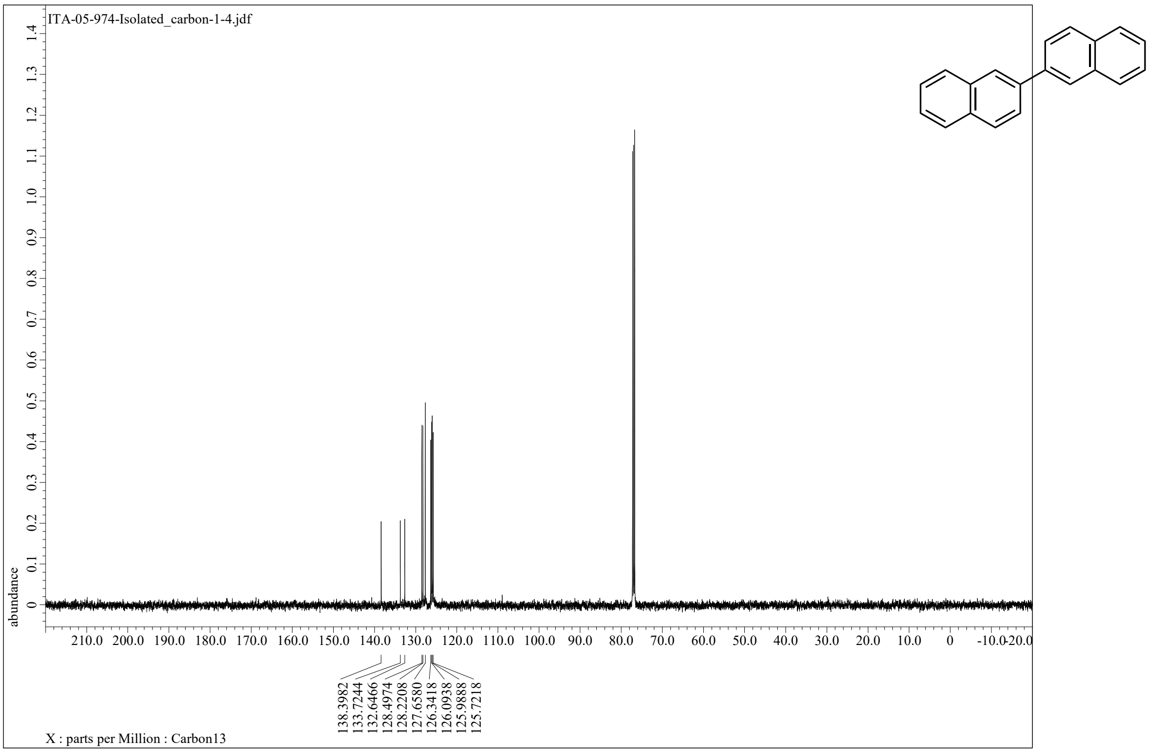

Supplementary Figure 67. ^{13}C NMR spectrum of **21** (126 MHz, $CDCl_3$).

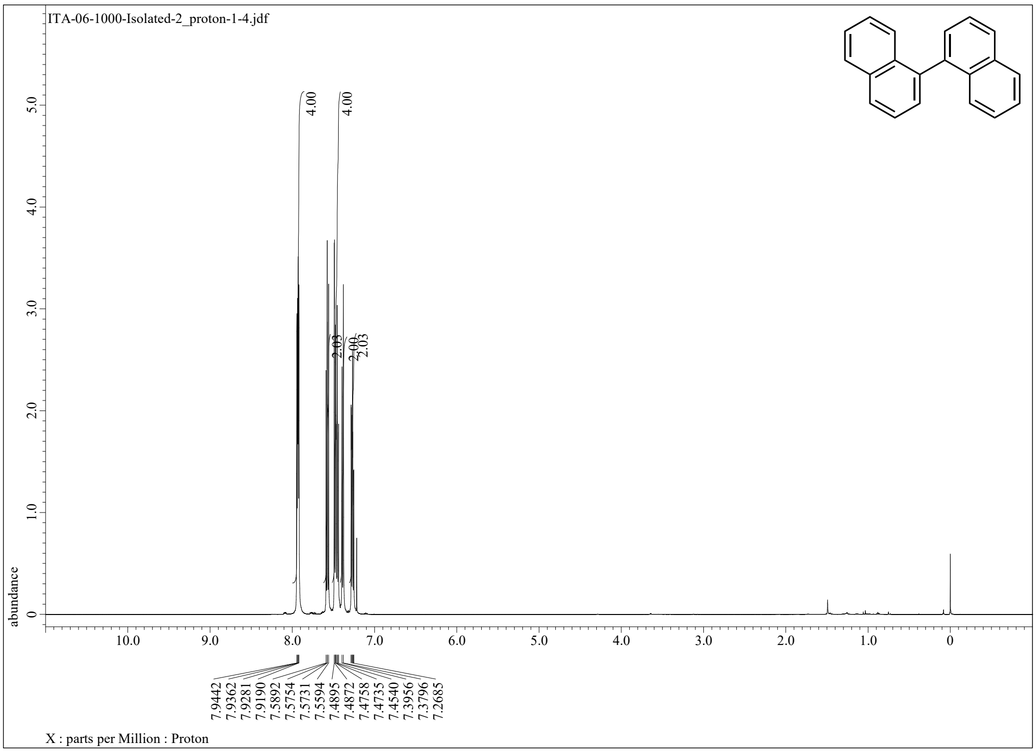

Supplementary Figure 68. ^{1}H NMR spectrum of **22** (500 MHz, $CDCl_3$).

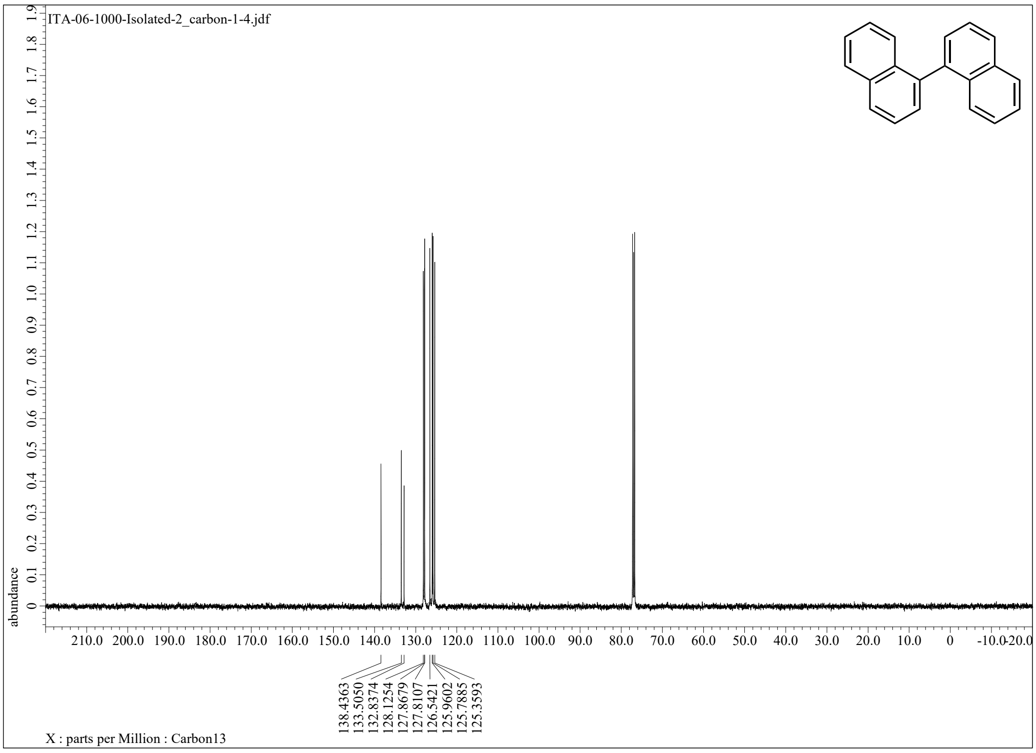

Supplementary Figure 69. ^{13}C NMR spectrum of **22** (126 MHz, $CDCl_3$).

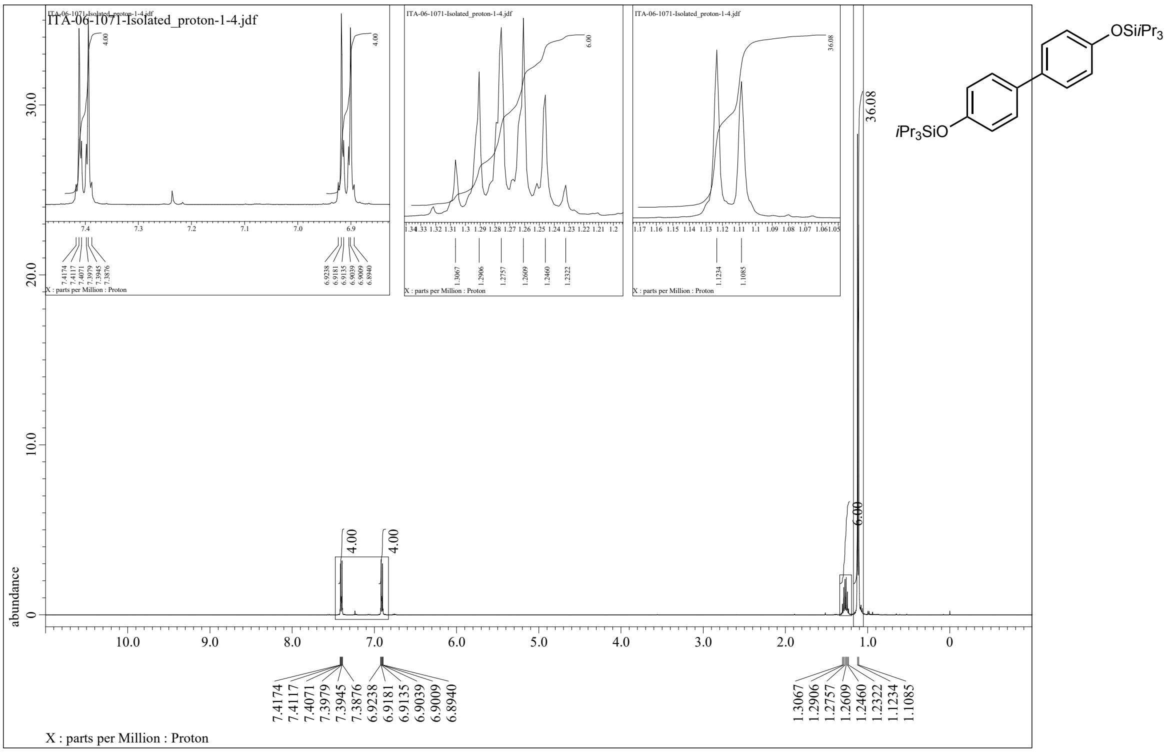

Supplementary Figure 70. ^{1}H NMR spectrum of **23** (500 MHz, $CDCl_3$).

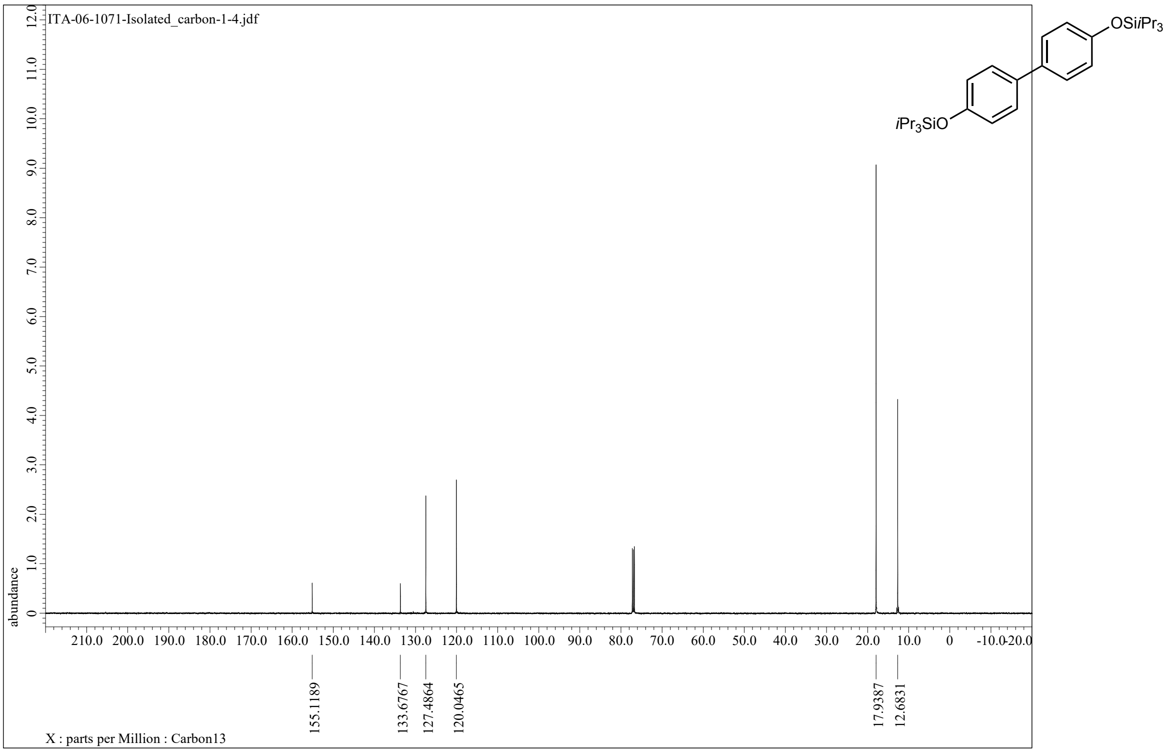

Supplementary Figure 71. ^{13}C NMR spectrum of **23** (126 MHz, $CDCl_3$).

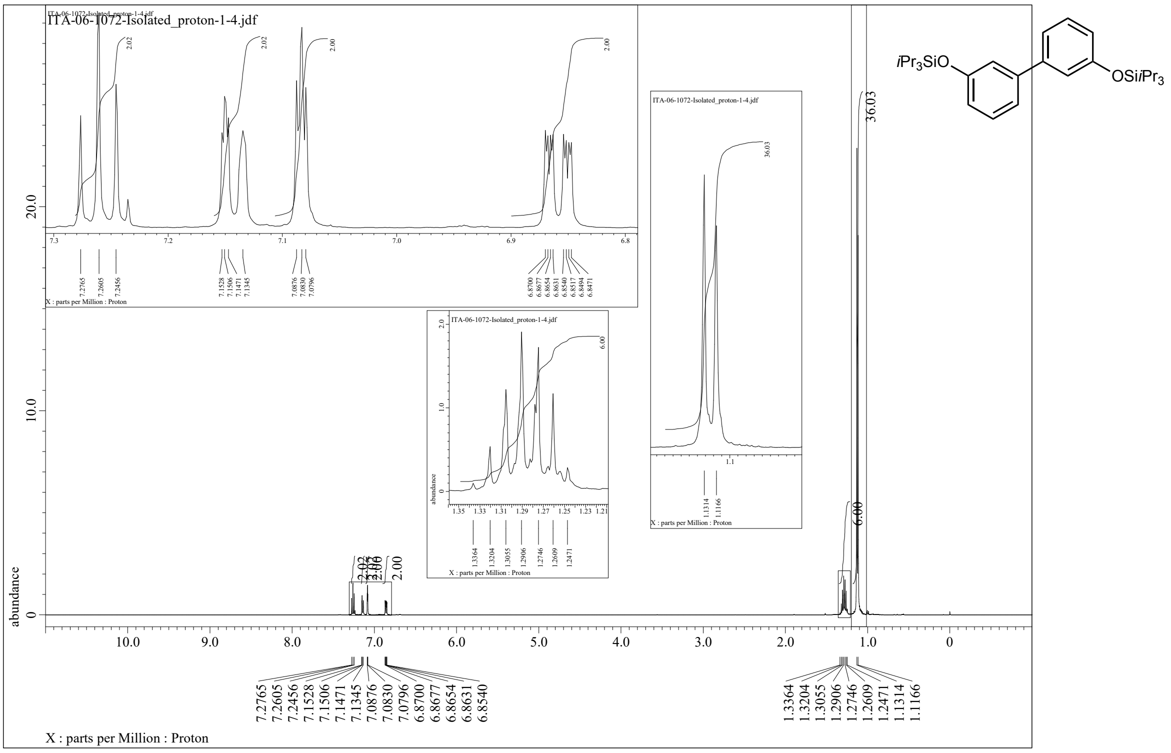

Supplementary Figure 72. ^{1}H NMR spectrum of **24** (500 MHz, $CDCl_3$).

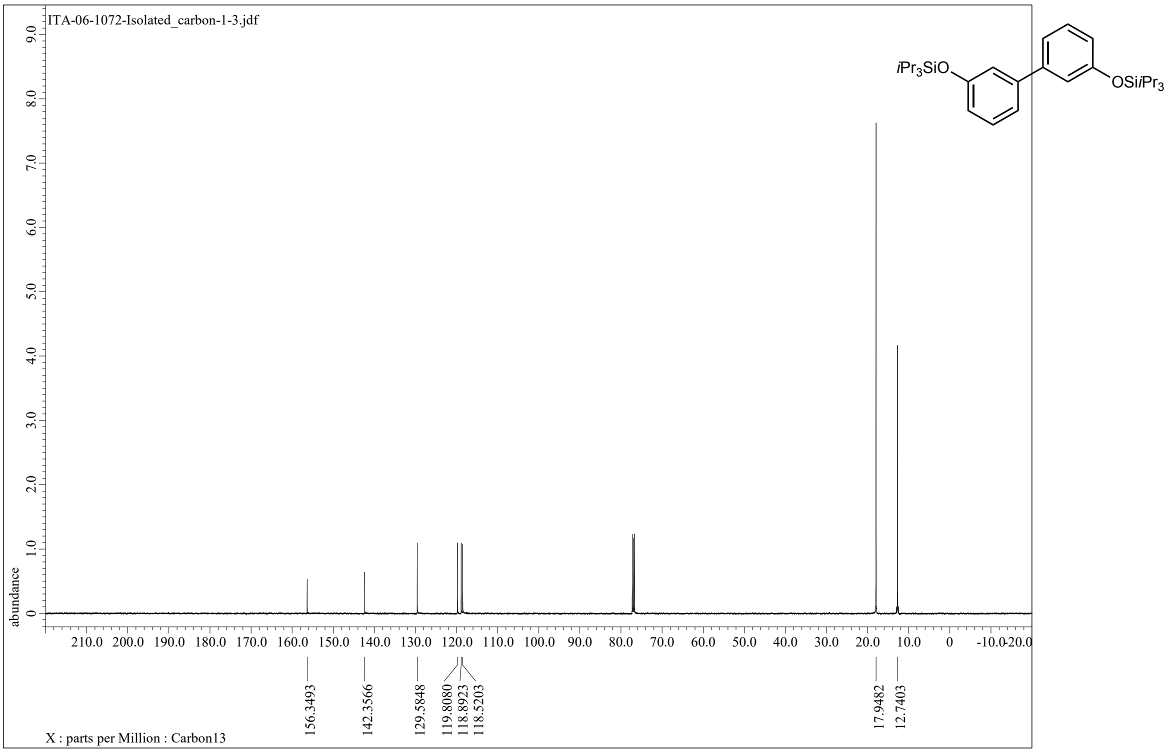

Supplementary Figure 73. ^{13}C NMR spectrum of **24** (126 MHz, $CDCl_3$).

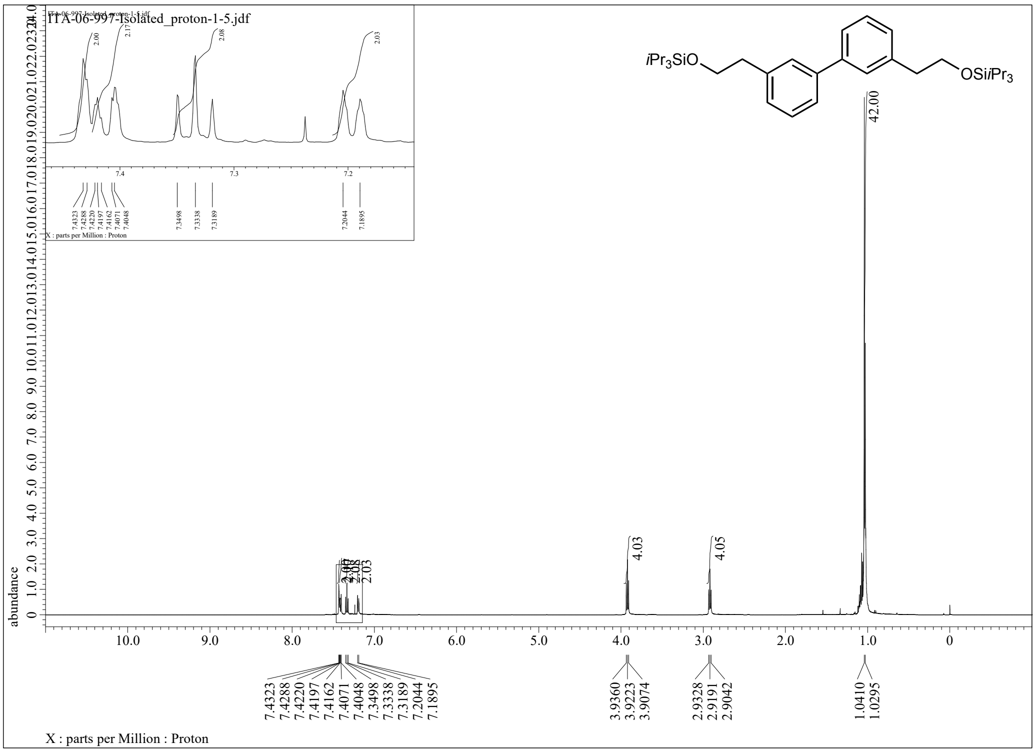

Supplementary Figure 74. ^{1}H NMR spectrum of **25** (500 MHz, $CDCl_3$).

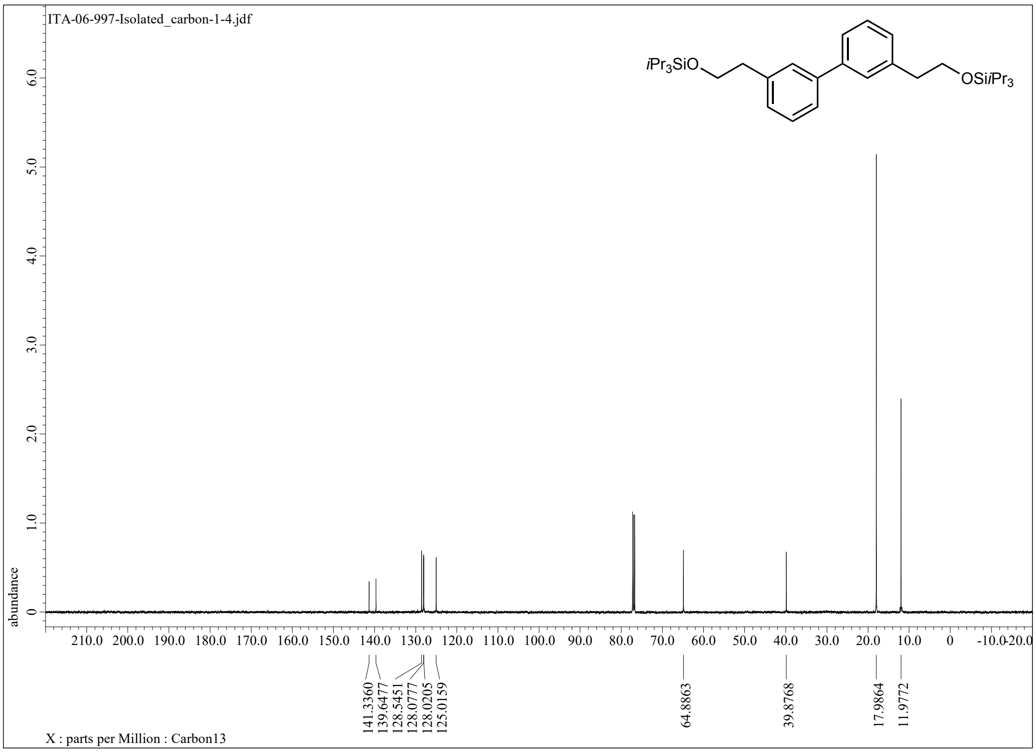

Supplementary Figure 75. ^{13}C NMR spectrum of **25** (126 MHz, $CDCl_3$).

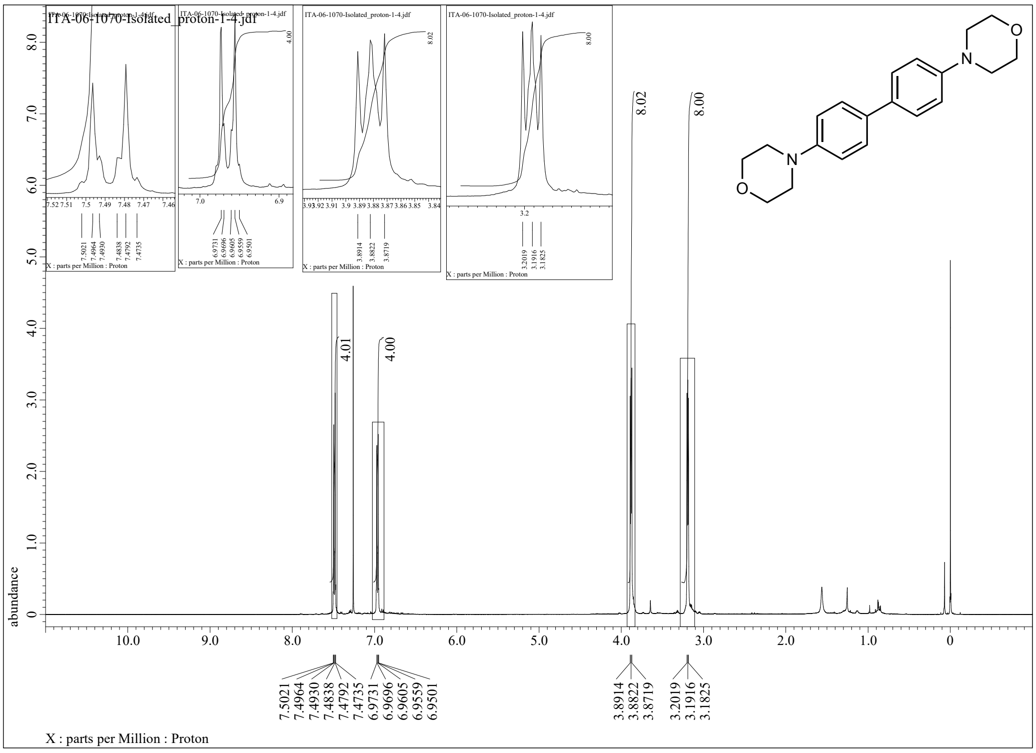

Supplementary Figure 76. ^{1}H NMR spectrum of **26** (500 MHz, $CDCl_3$).

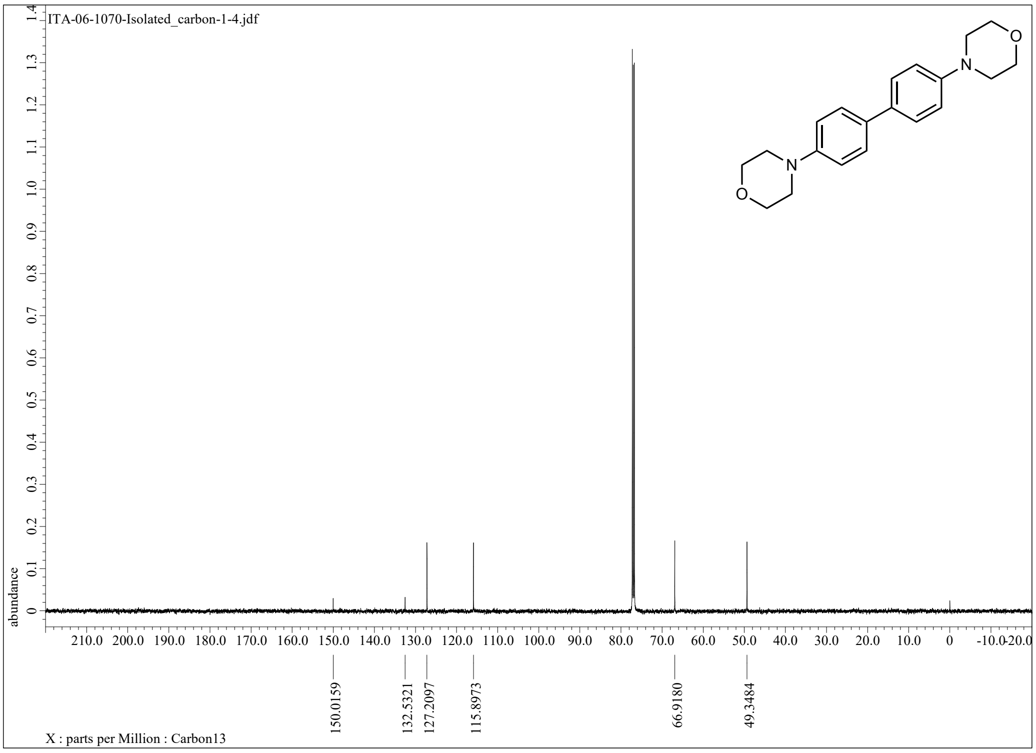

Supplementary Figure 77. ^{13}C NMR spectrum of **26** (126 MHz, $CDCl_3$).

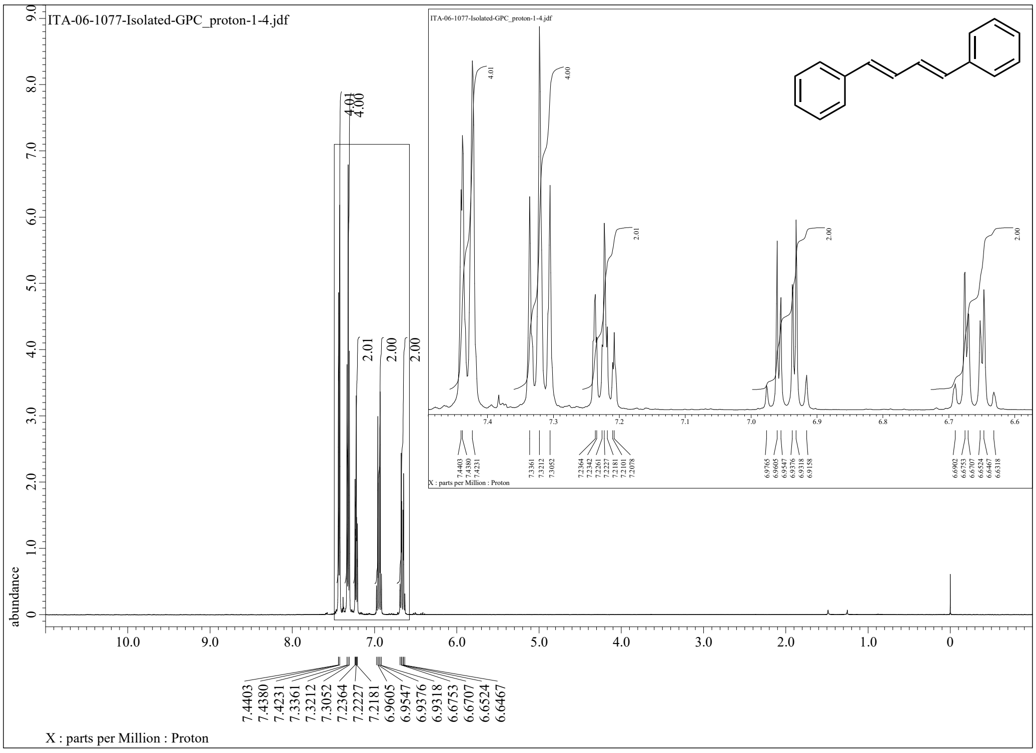

Supplementary Figure 78. ^{1}H NMR spectrum of **27** (500 MHz, $CDCl_3$).

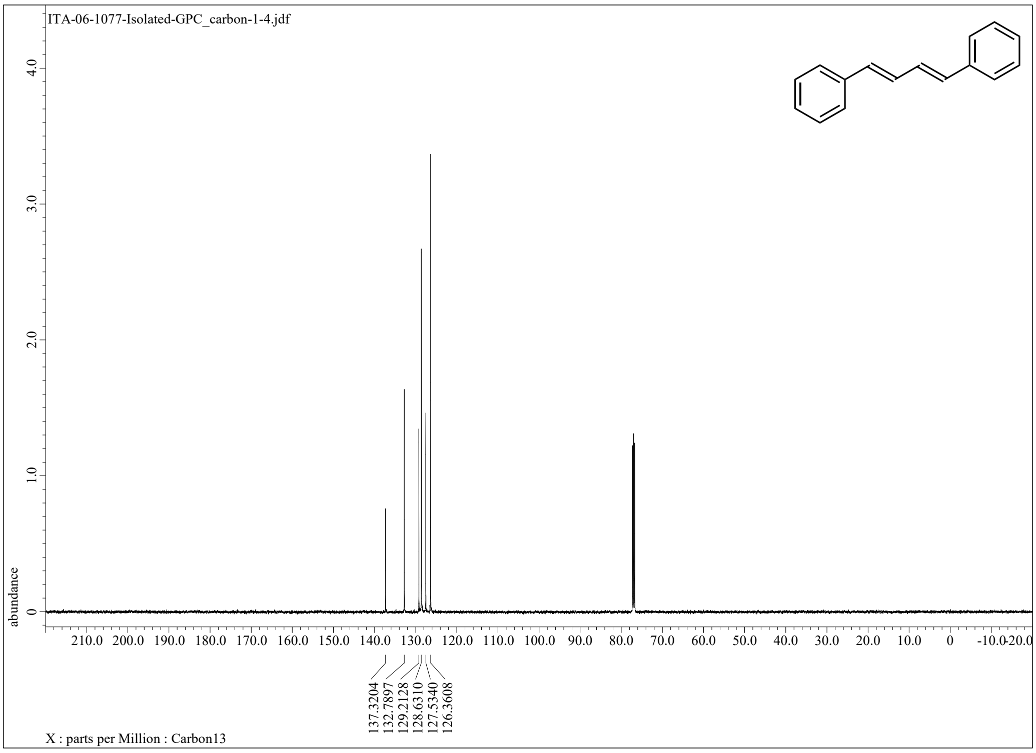

Supplementary Figure 79. ^{13}C NMR spectrum of **27** (126 MHz, $CDCl_3$).

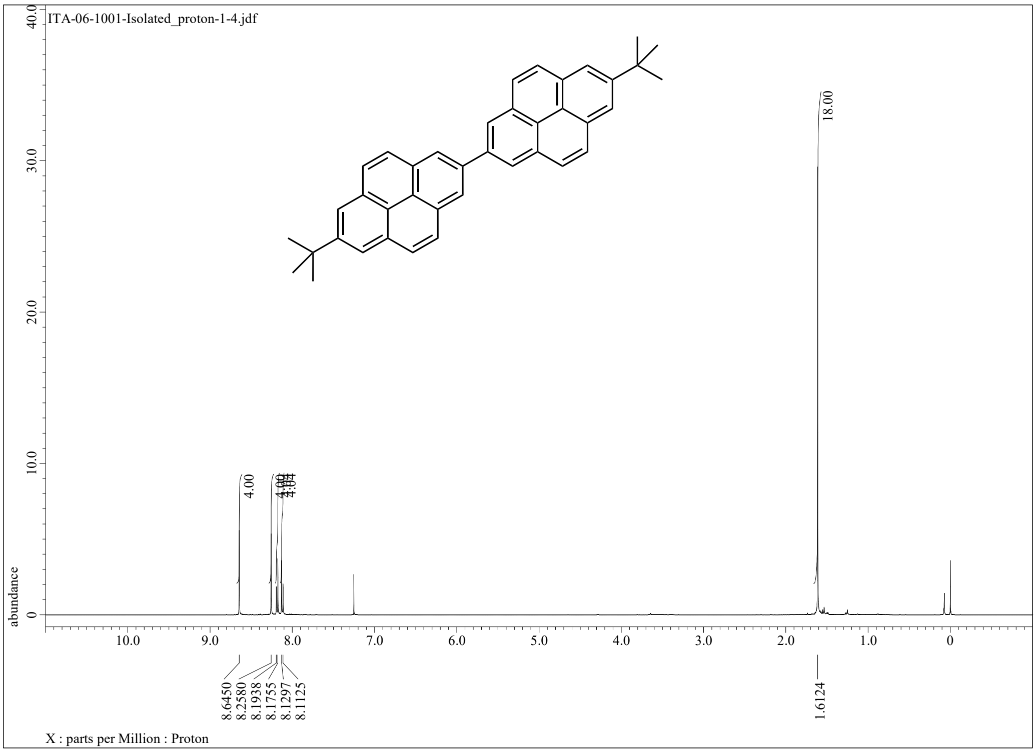

Supplementary Figure 80. ^{1}H NMR spectrum of **28** (500 MHz, $CDCl_3$).

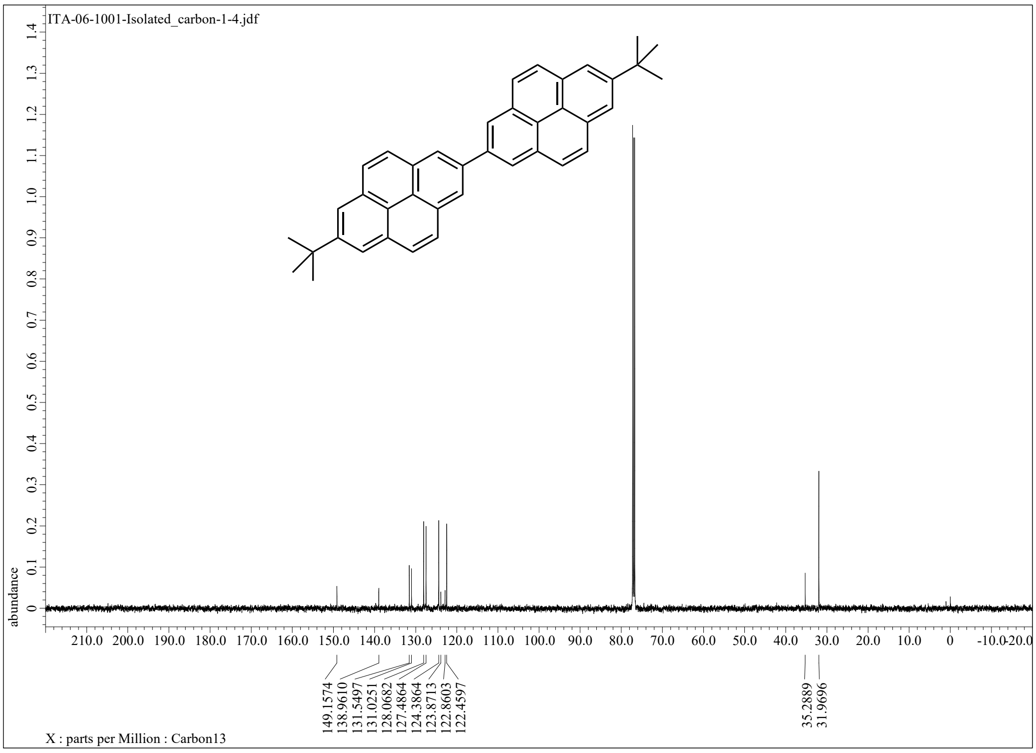

Supplementary Figure 81. ^{13}C NMR spectrum of **28** (126 MHz, $CDCl_3$).

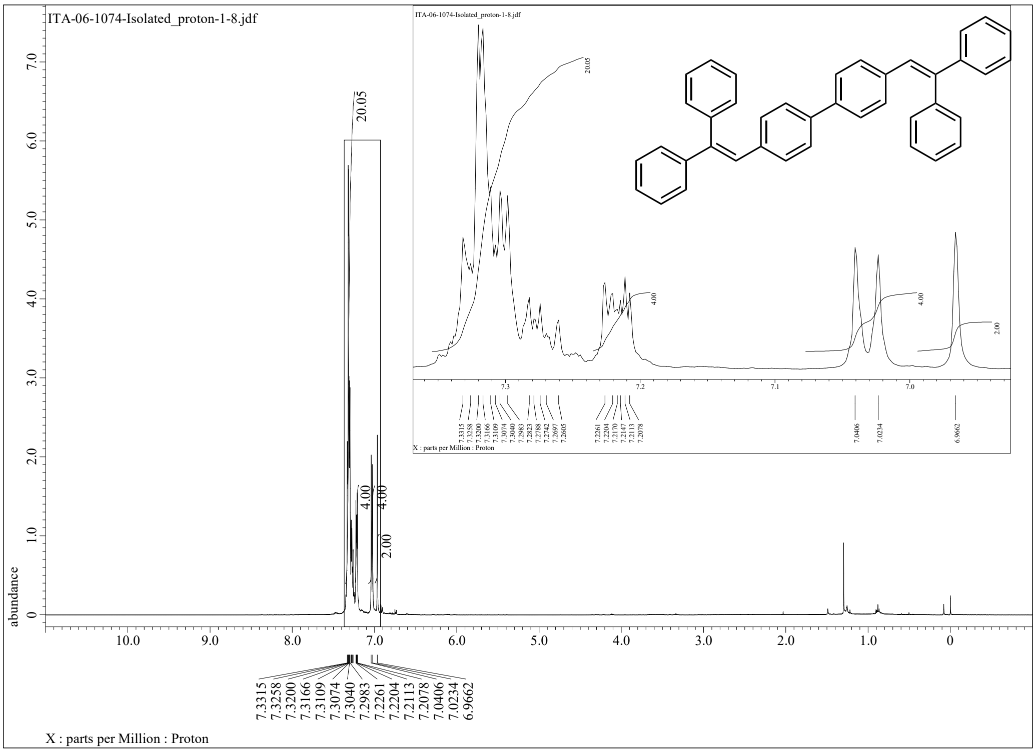

Supplementary Figure 82. ^{1}H NMR spectrum of **29** (500 MHz, $CDCl_3$).

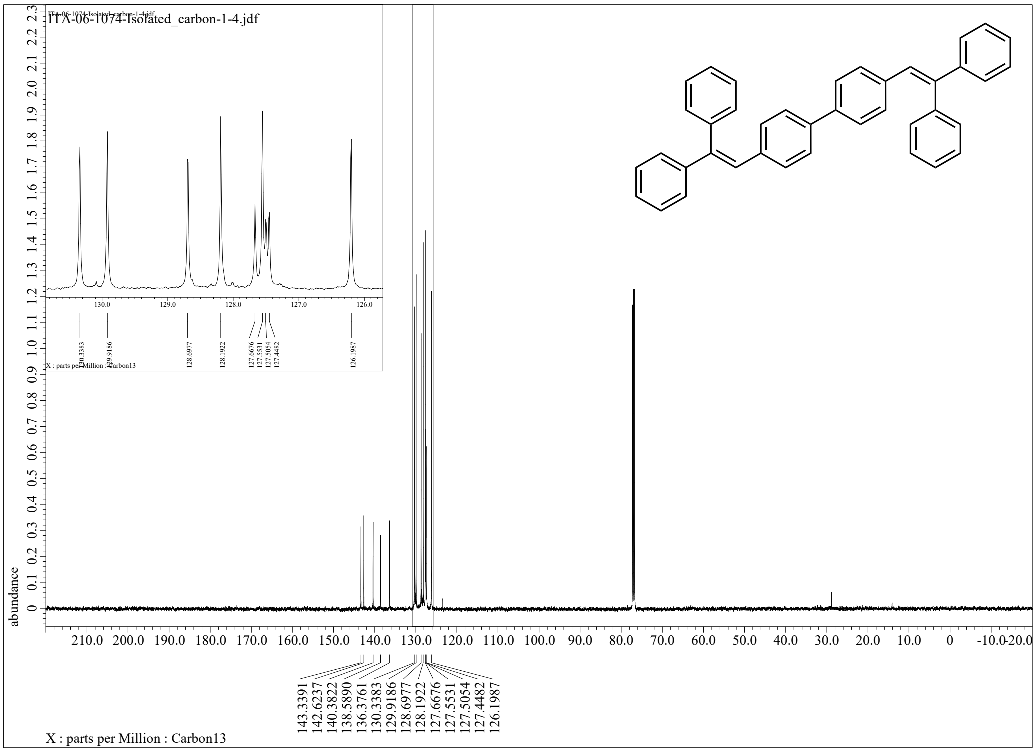

Supplementary Figure 83. ^{13}C NMR spectrum of **29** (126 MHz, $CDCl_3$).

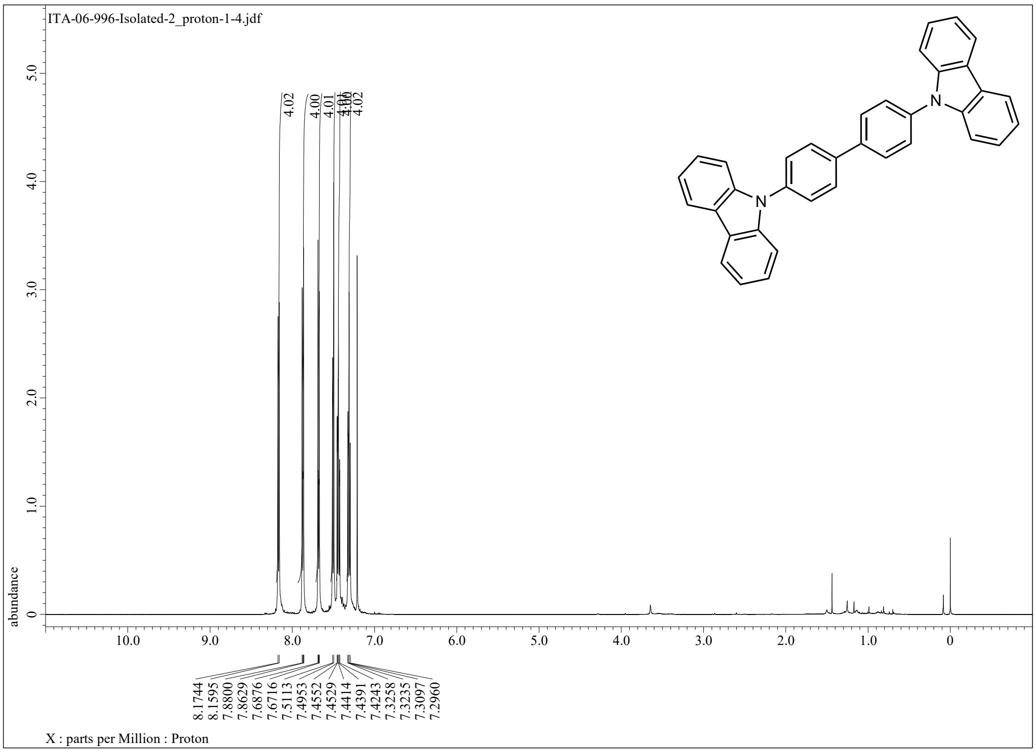

Supplementary Figure 84. ^{1}H NMR spectrum of **30** (500 MHz, $CDCl_3$).

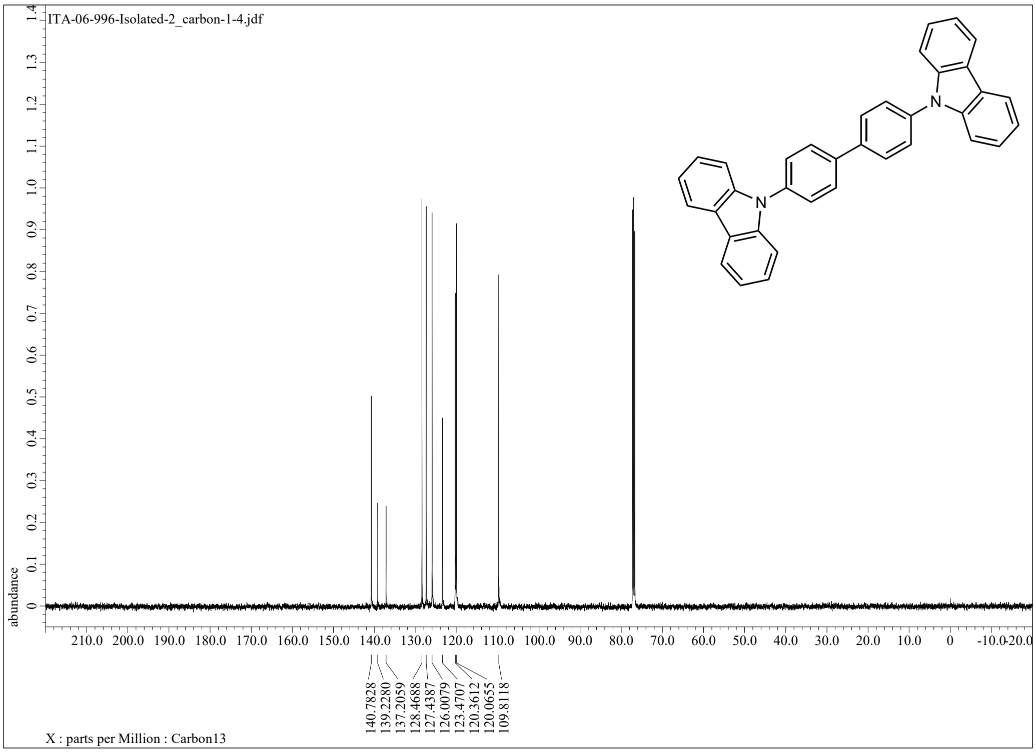

Supplementary Figure 85. ^{13}C NMR spectrum of **30** (126 MHz, $CDCl_3$).

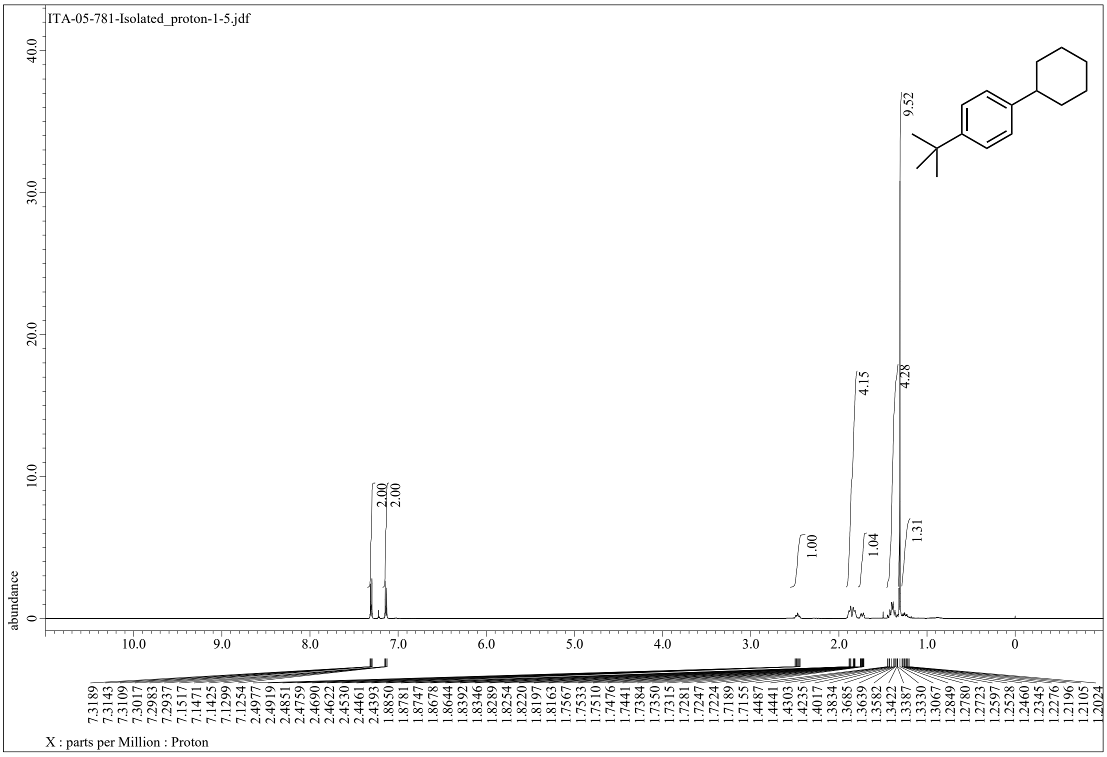

Supplementary Figure 86. ^{1}H NMR spectrum of **31** (500 MHz, $CDCl_3$).

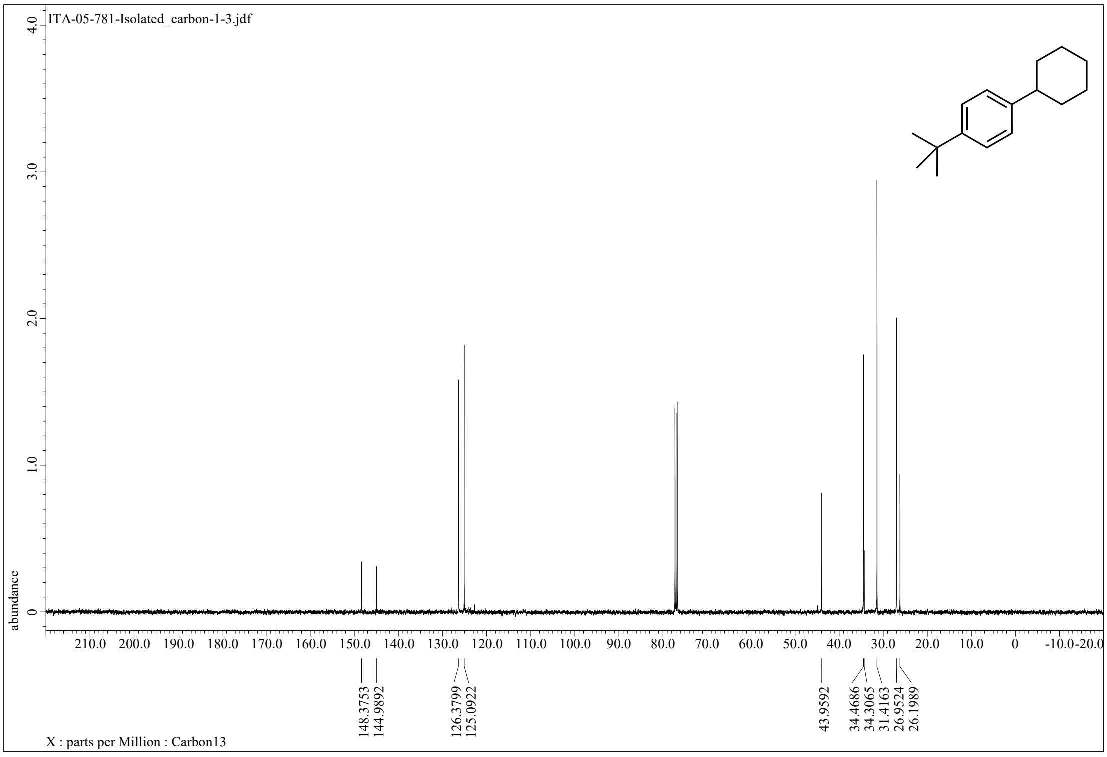

Supplementary Figure 87. ^{13}C NMR spectrum of **31** (126 MHz, $CDCl_3$).

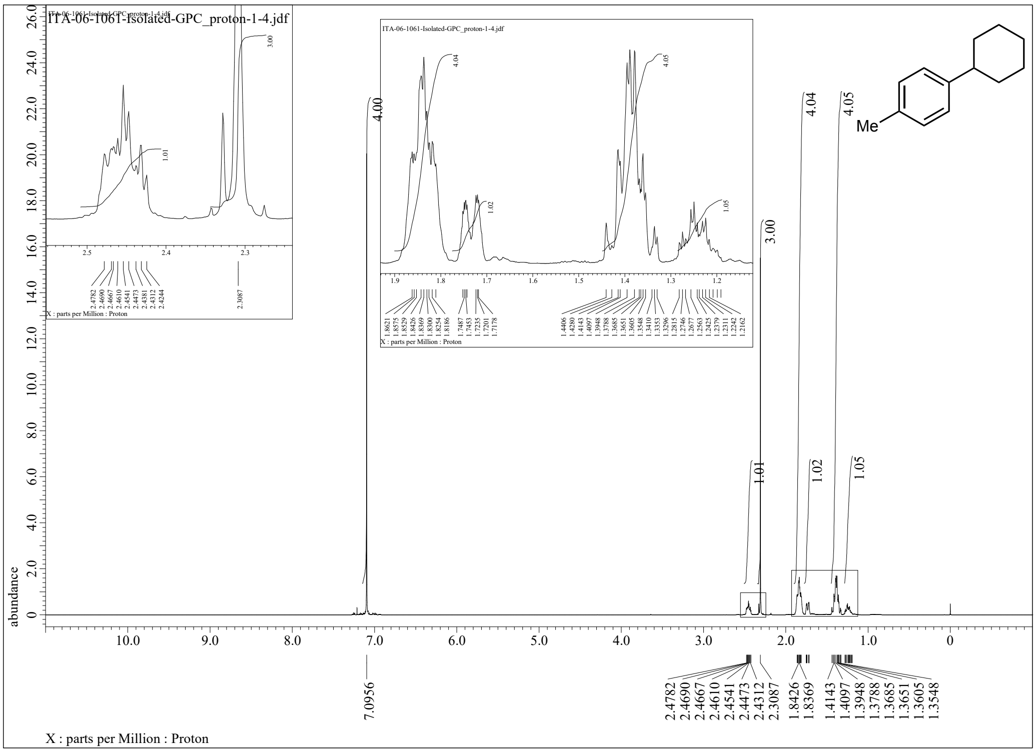

Supplementary Figure 88. ^{1}H NMR spectrum of **32** (500 MHz, $CDCl_3$).

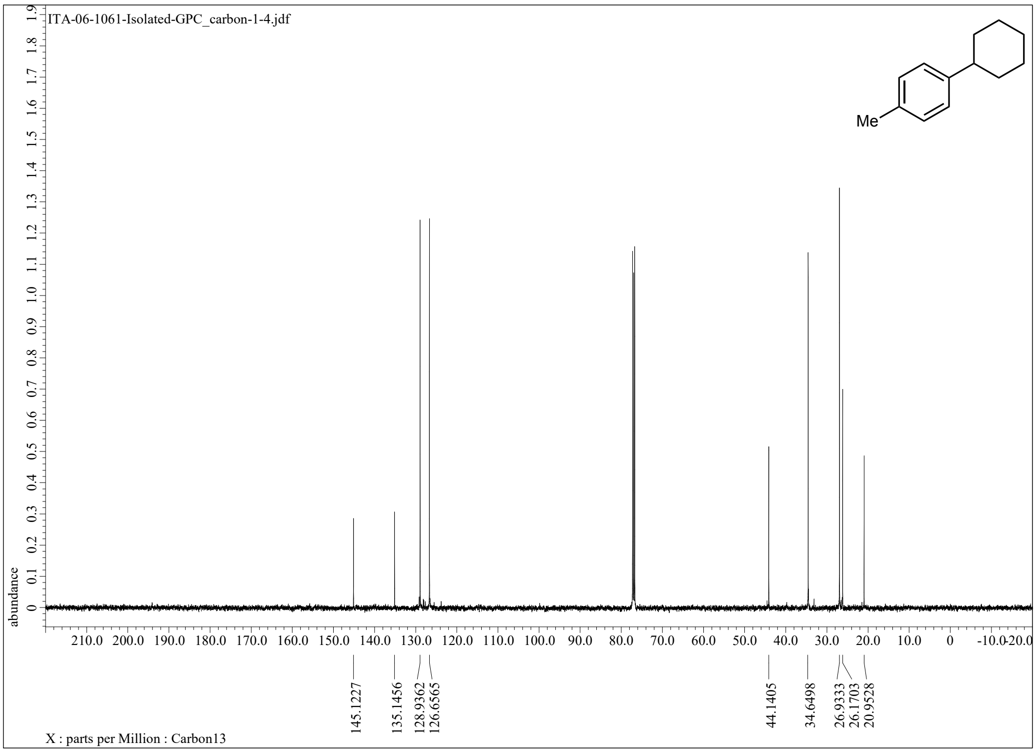

Supplementary Figure 89. ^{13}C NMR spectrum of **32** (126 MHz, $CDCl_3$).

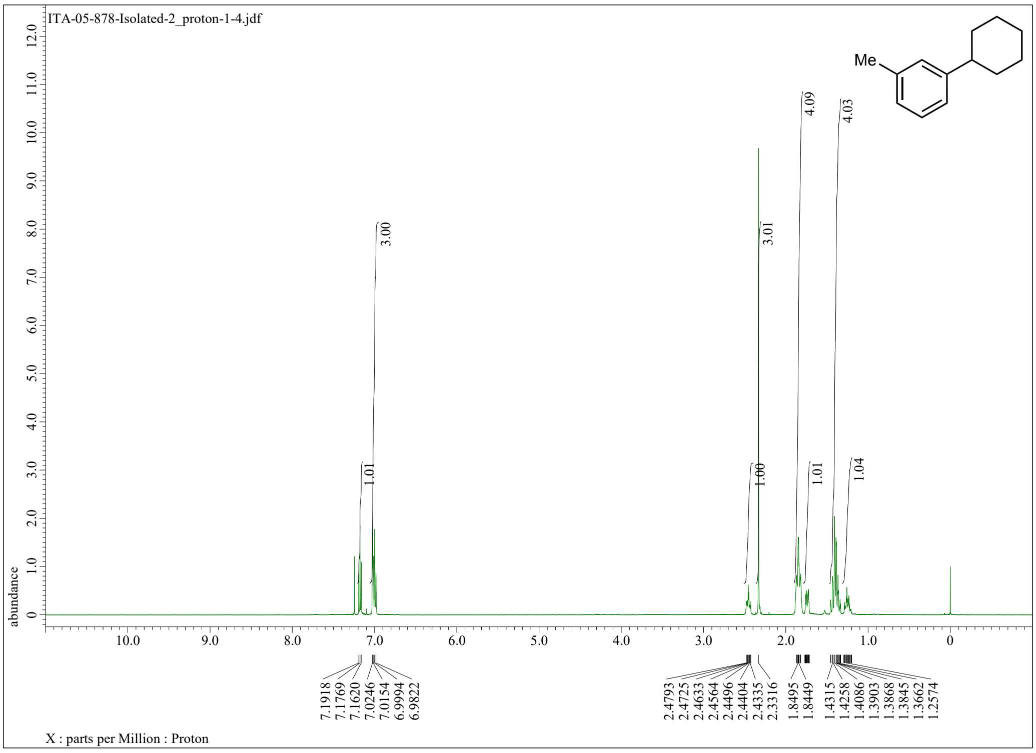

Supplementary Figure 90. ^{1}H NMR spectrum of **33** (500 MHz, $CDCl_3$).

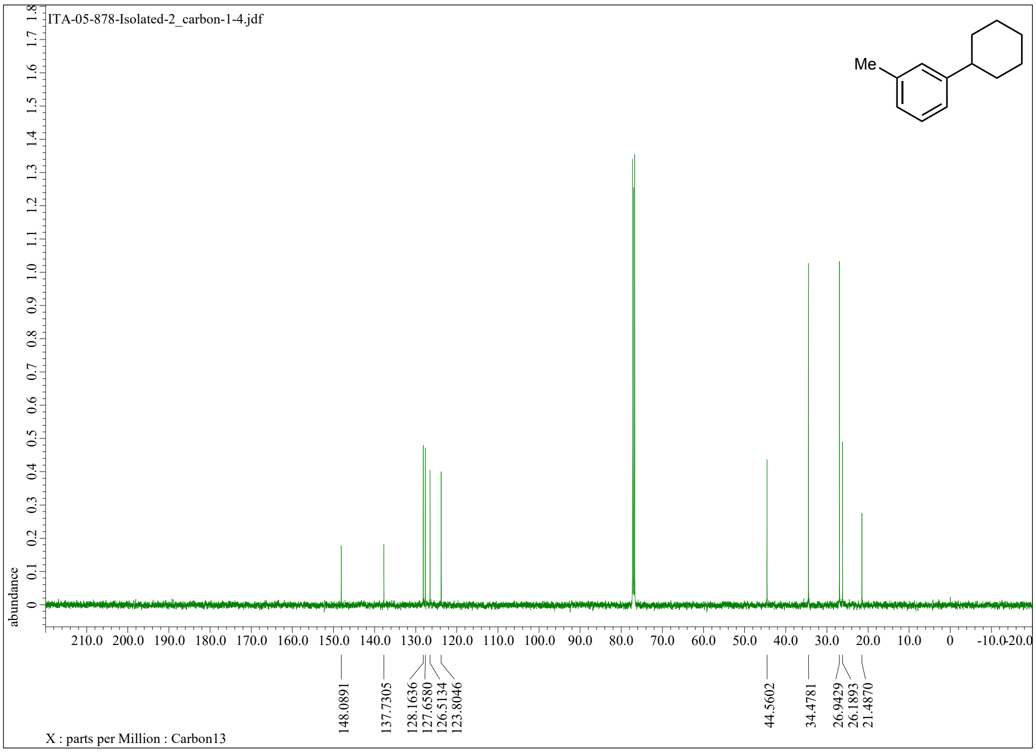

Supplementary Figure 91. ^{13}C NMR spectrum of **33** (126 MHz, $CDCl_3$).

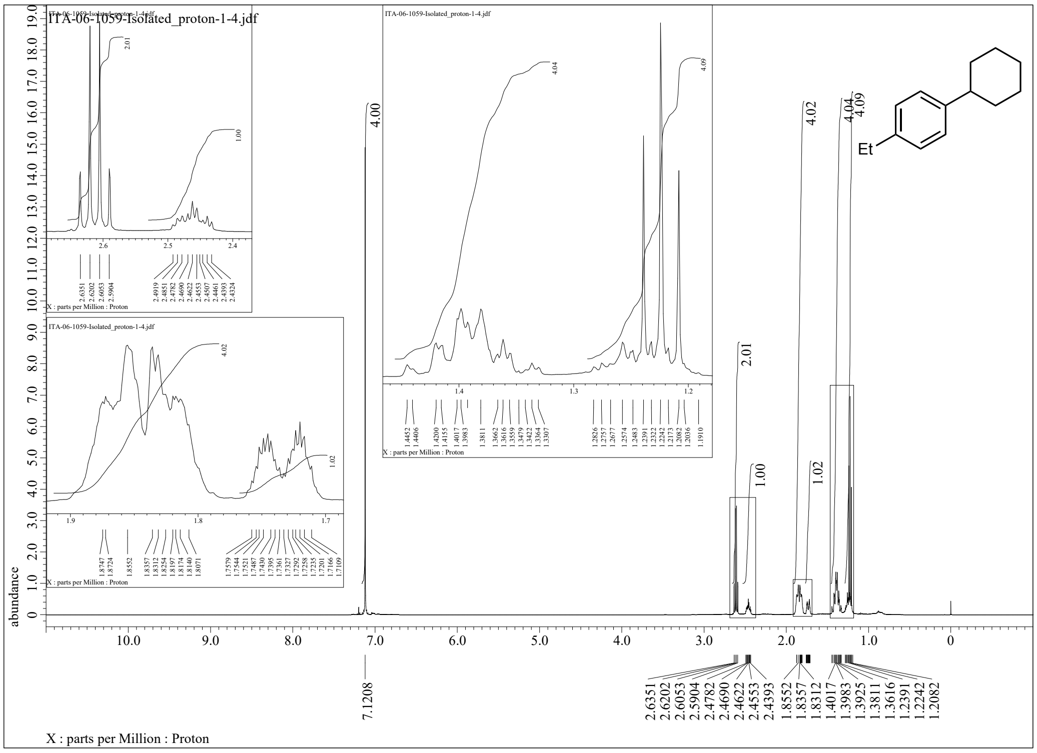

Supplementary Figure 92. ^{1}H NMR spectrum of **34** (500 MHz, $CDCl_3$).

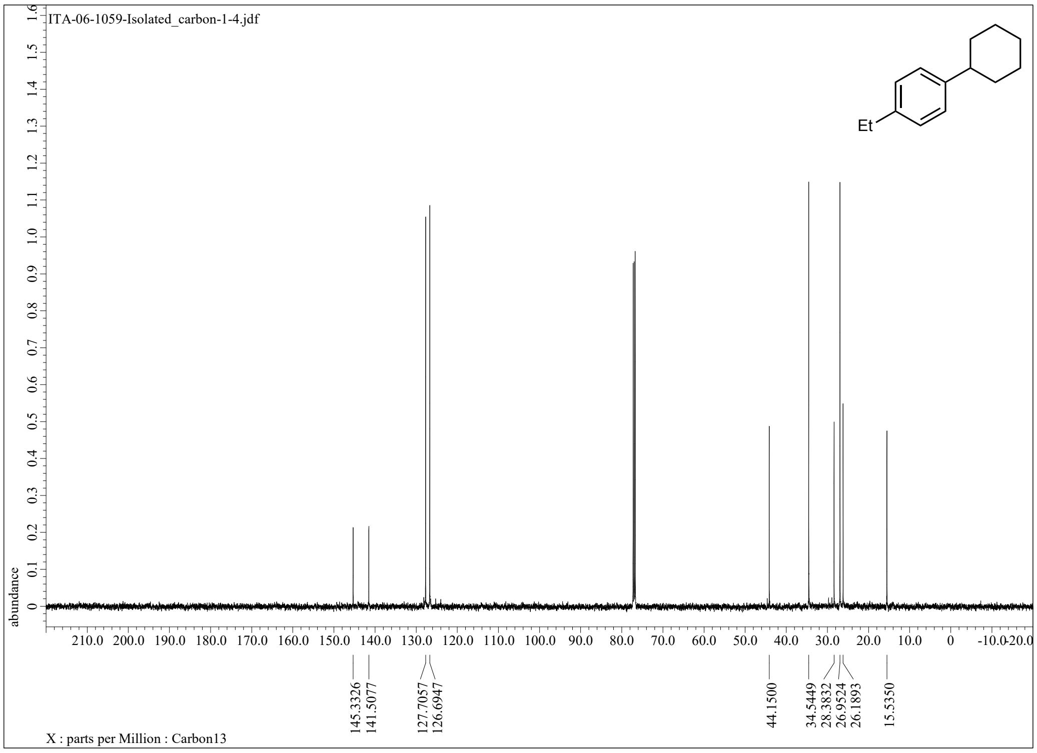

Supplementary Figure 93. ^{13}C NMR spectrum of **34** (126 MHz, $CDCl_3$).

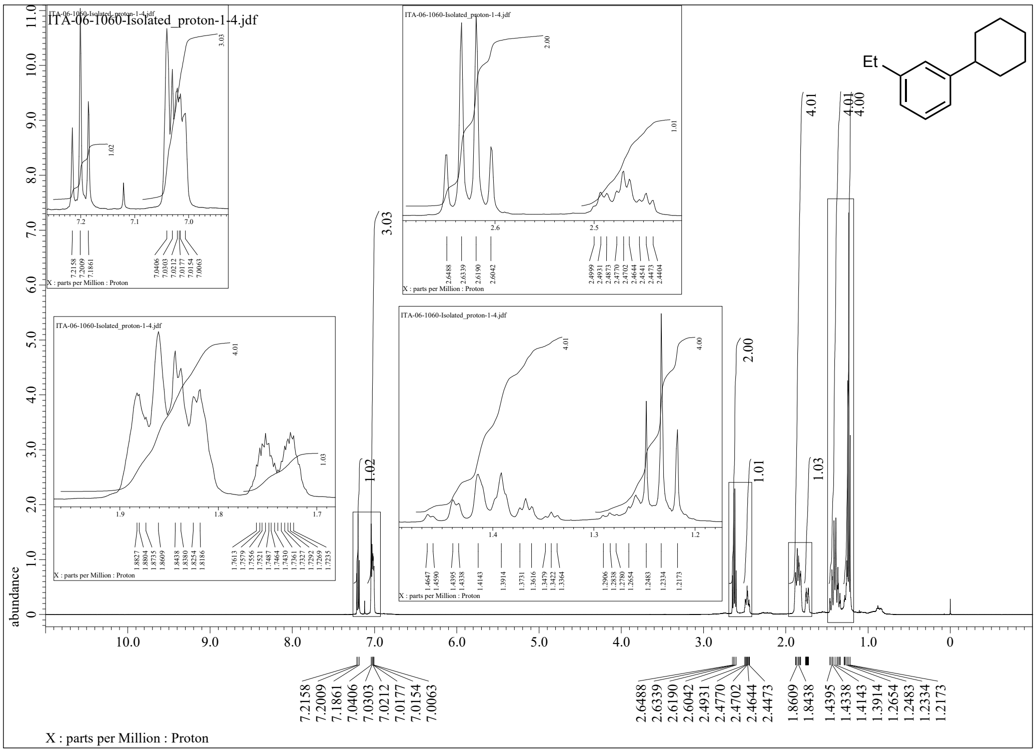

Supplementary Figure 94. ^{1}H NMR spectrum of **35** (500 MHz, $CDCl_3$).

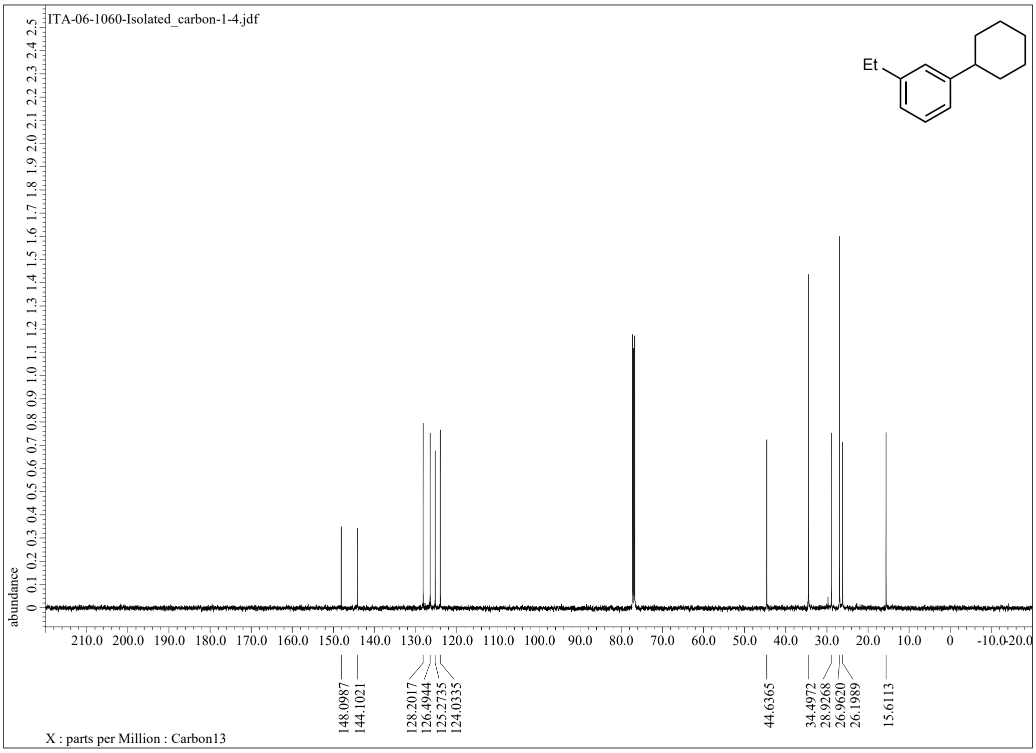

Supplementary Figure 95. ^{13}C NMR spectrum of **35** (126 MHz, $CDCl_3$).

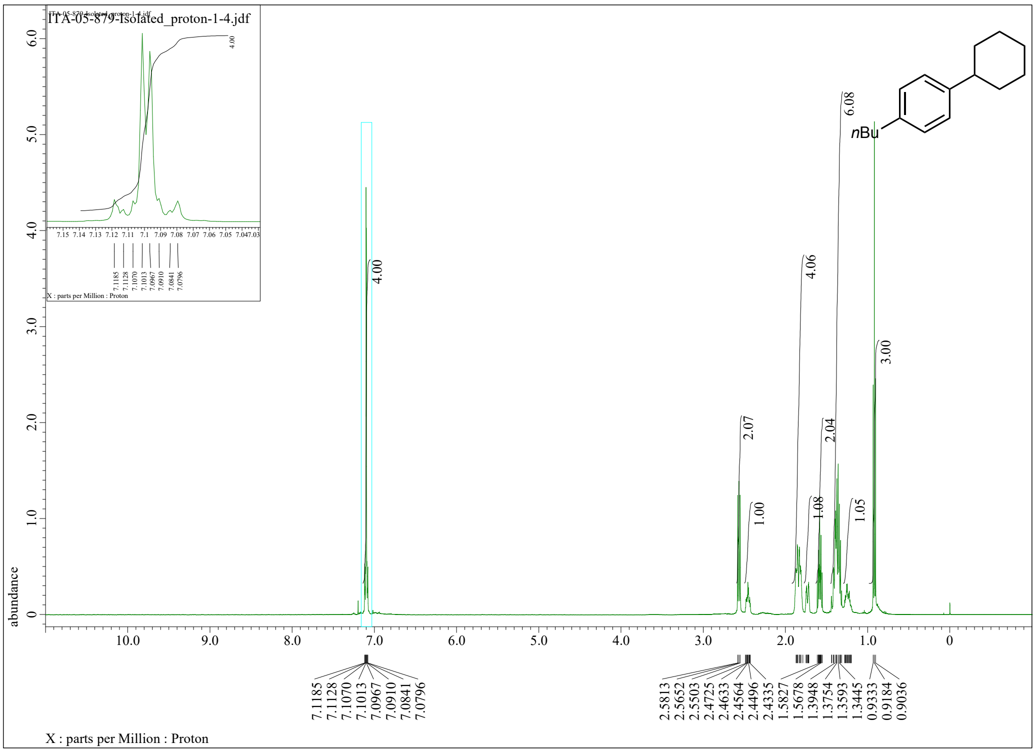

Supplementary Figure 96. ^{1}H NMR spectrum of **36** (500 MHz, $CDCl_3$).

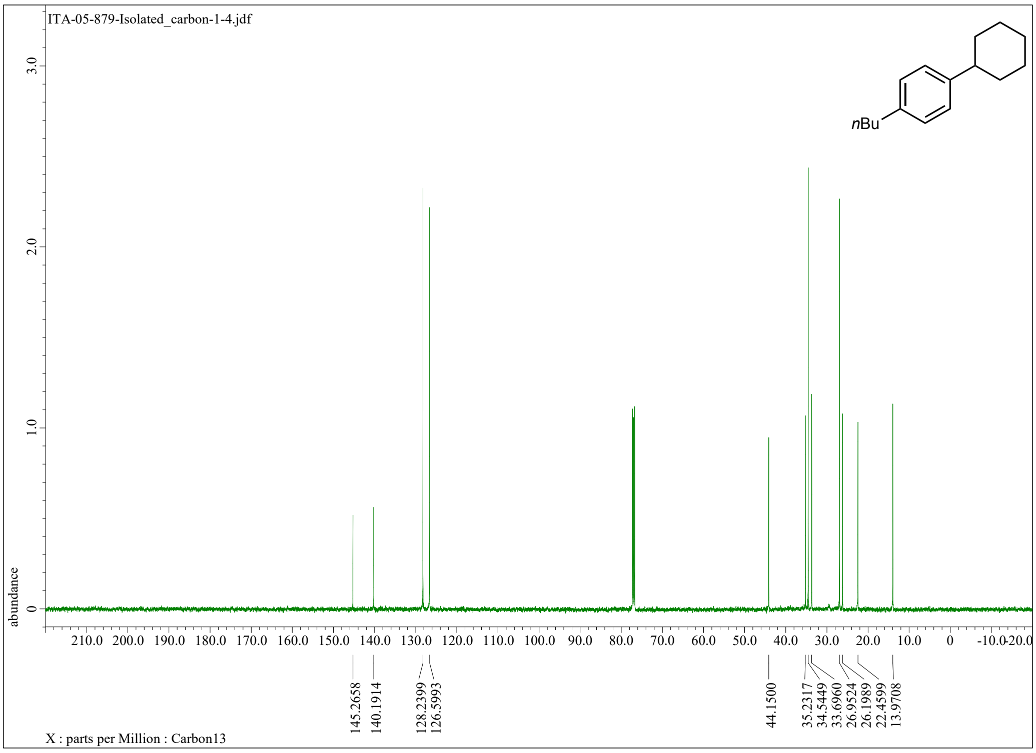

Supplementary Figure 97. ^{13}C NMR spectrum of **36** (126 MHz, $CDCl_3$).

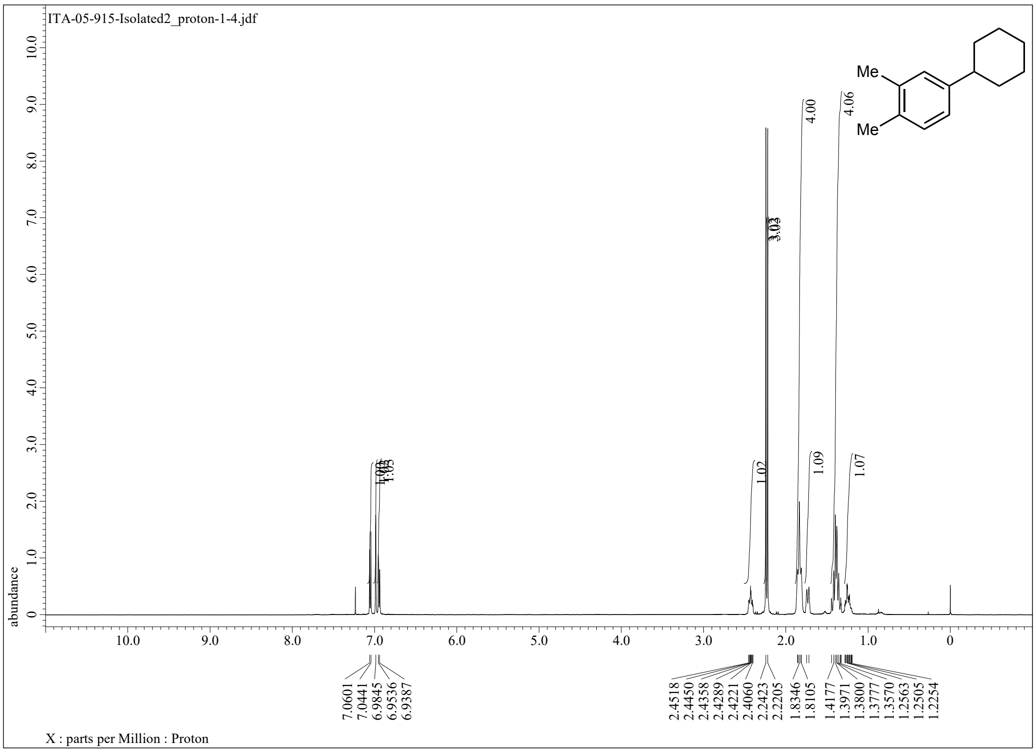

Supplementary Figure 98. ^{1}H NMR spectrum of **37** (500 MHz, $CDCl_3$).

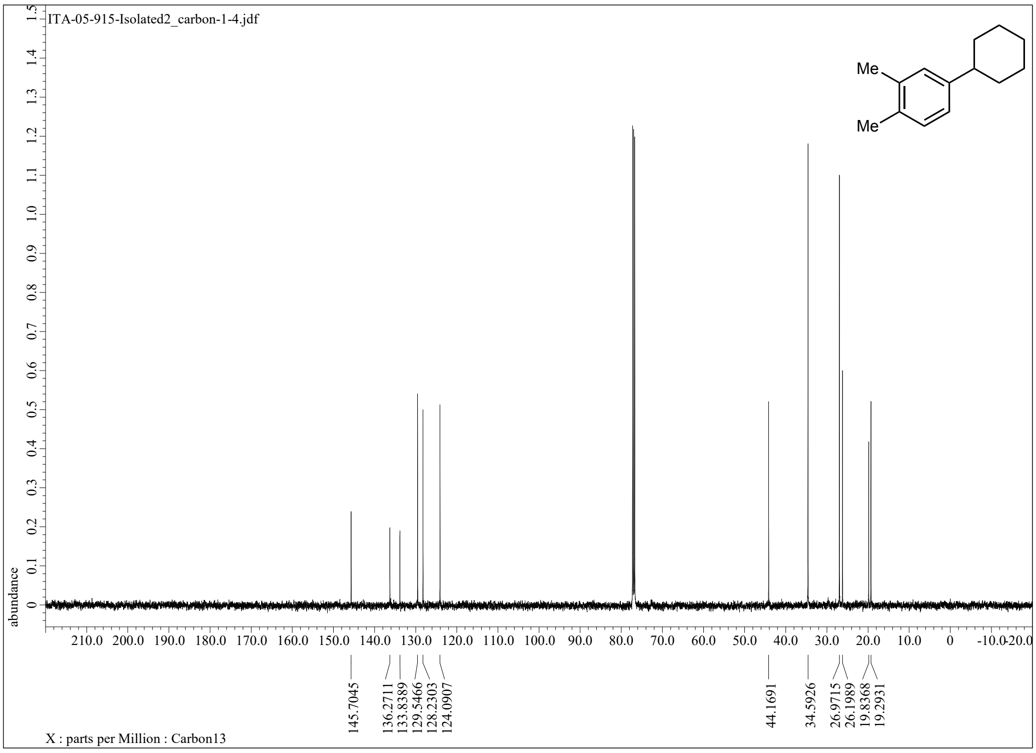

Supplementary Figure 99. ^{13}C NMR spectrum of **37** (126 MHz, $CDCl_3$).

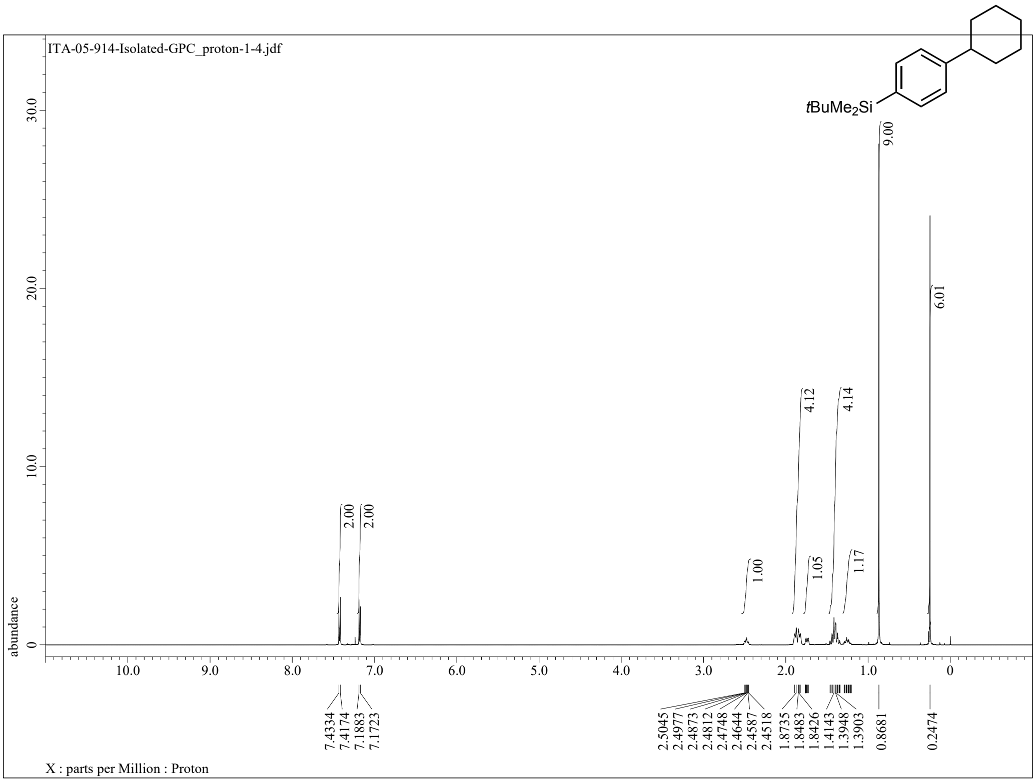

Supplementary Figure 100. ^{1}H NMR spectrum of **38** (500 MHz, $CDCl_3$).

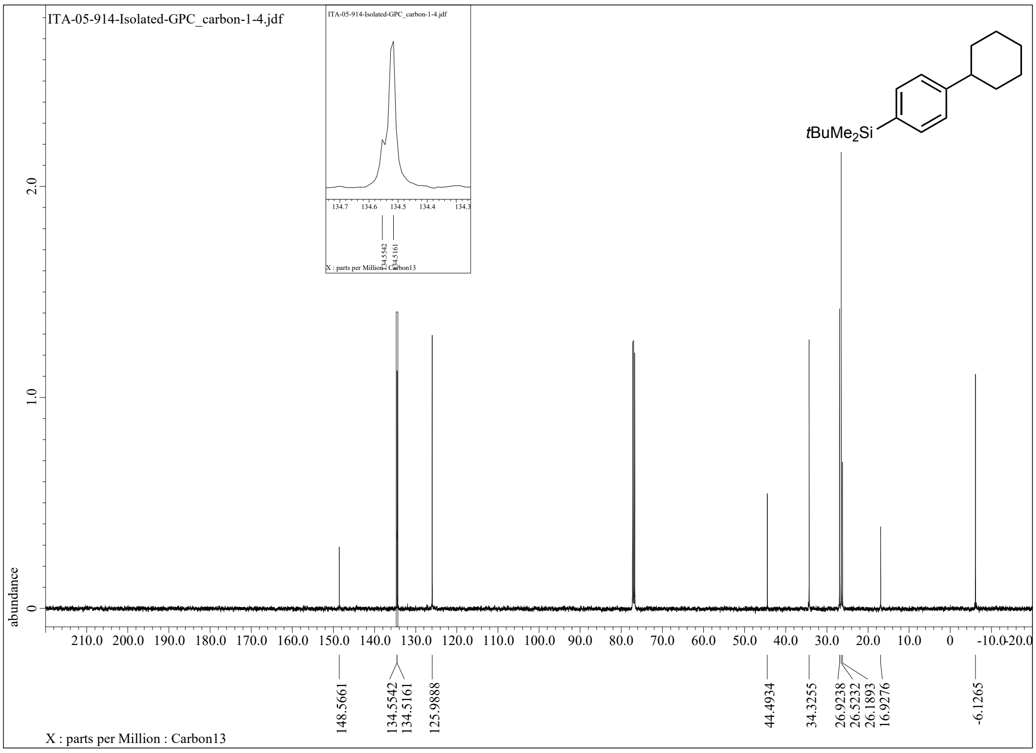

Supplementary Figure 101. ^{13}C NMR spectrum of **38** (126 MHz, $CDCl_3$).

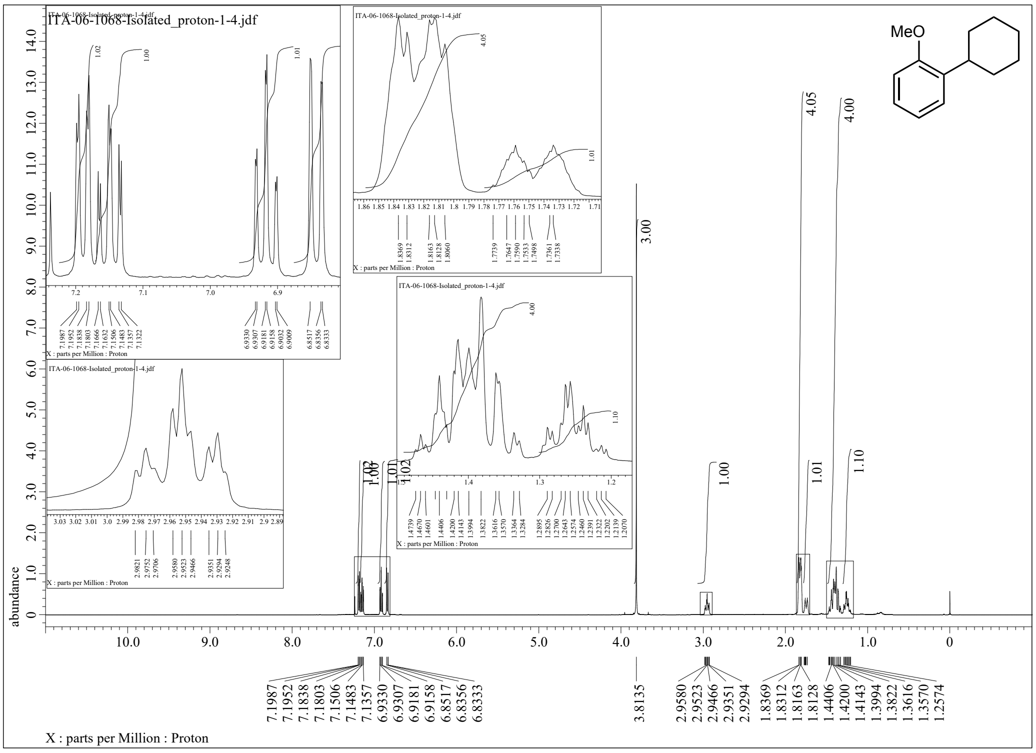

Supplementary Figure 102. ^{1}H NMR spectrum of **39** (500 MHz, $CDCl_3$).

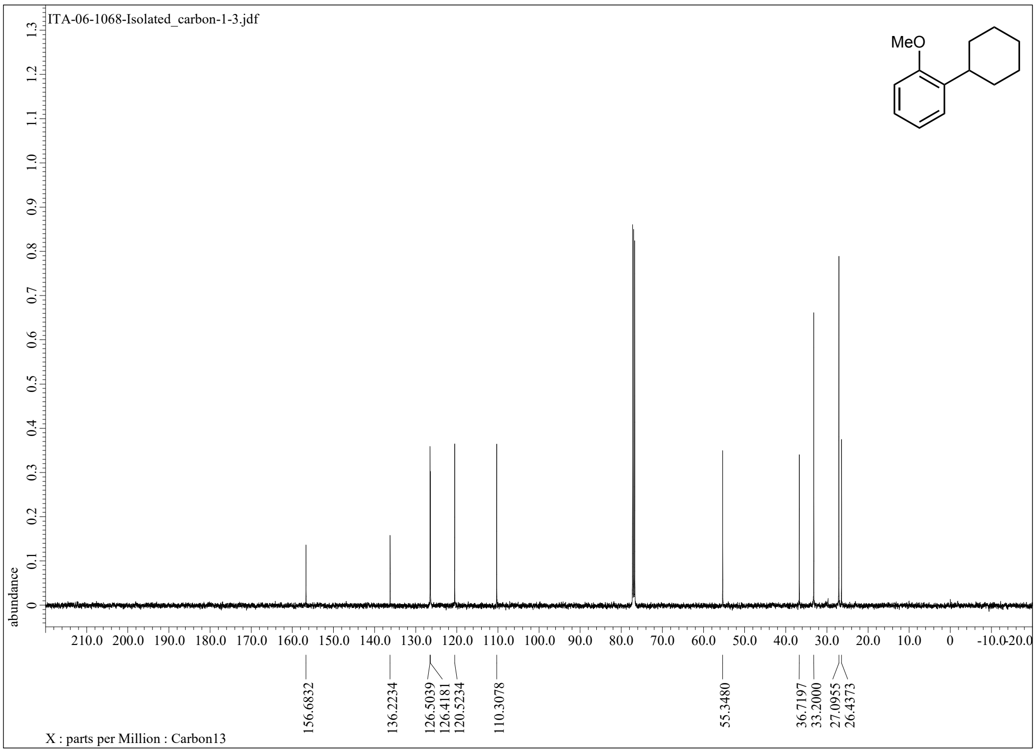

Supplementary Figure 103. ^{13}C NMR spectrum of **39** (126 MHz, $CDCl_3$).

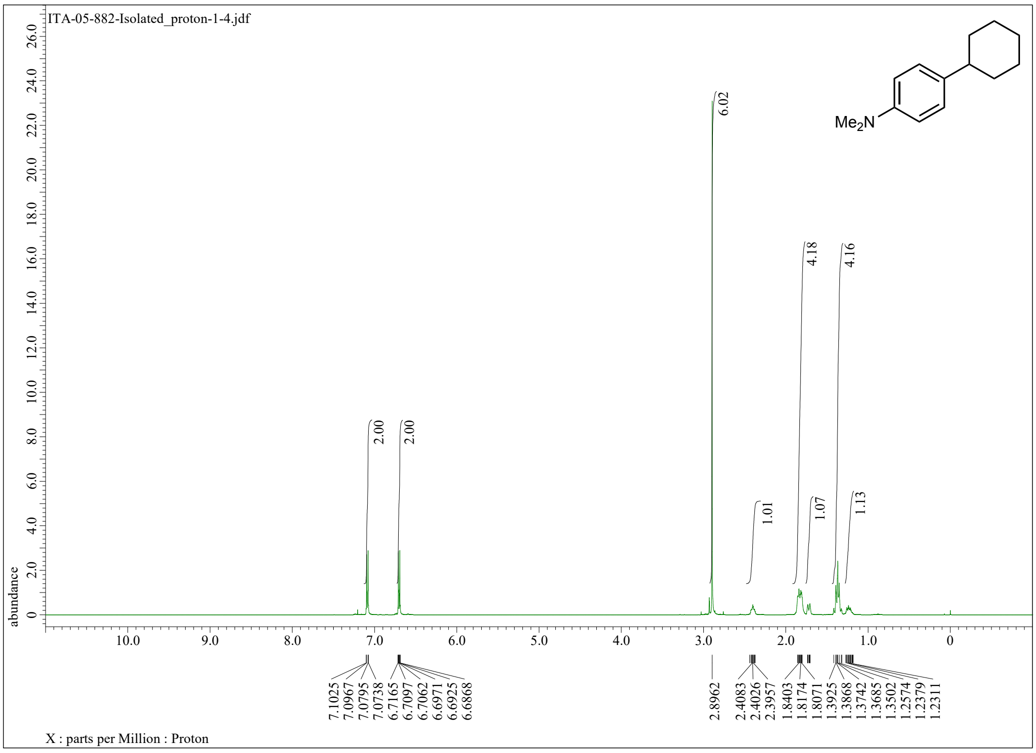

Supplementary Figure 104. ^{1}H NMR spectrum of **40** (500 MHz, $CDCl_3$).

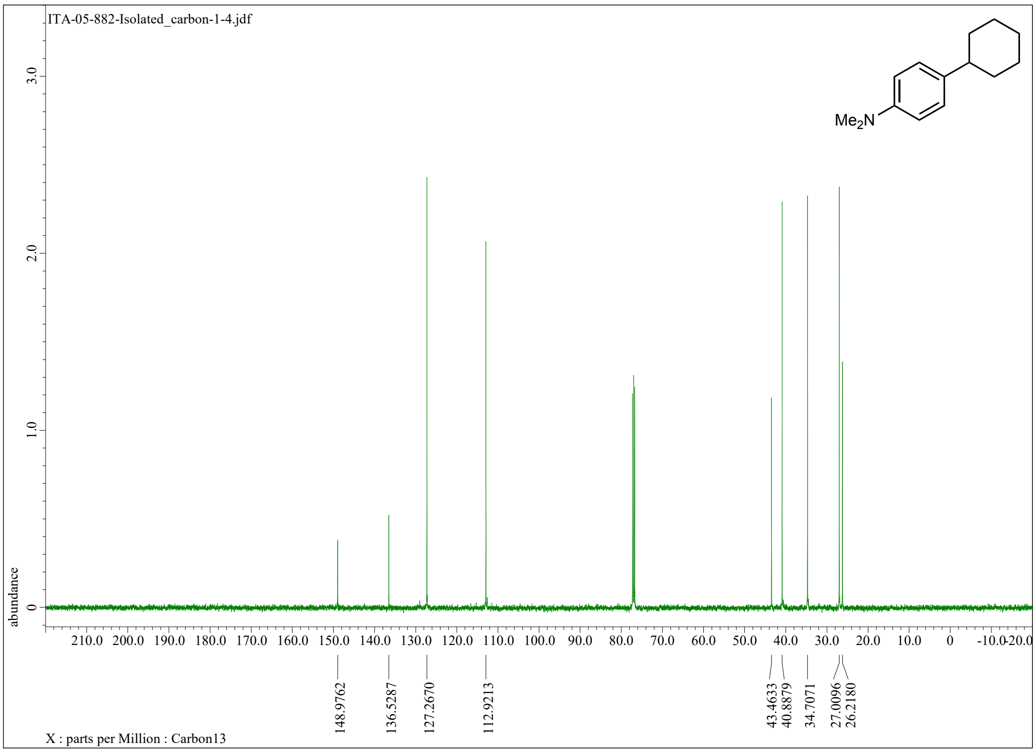

Supplementary Figure 105. ^{13}C NMR spectrum of **40** (126 MHz, $CDCl_3$).

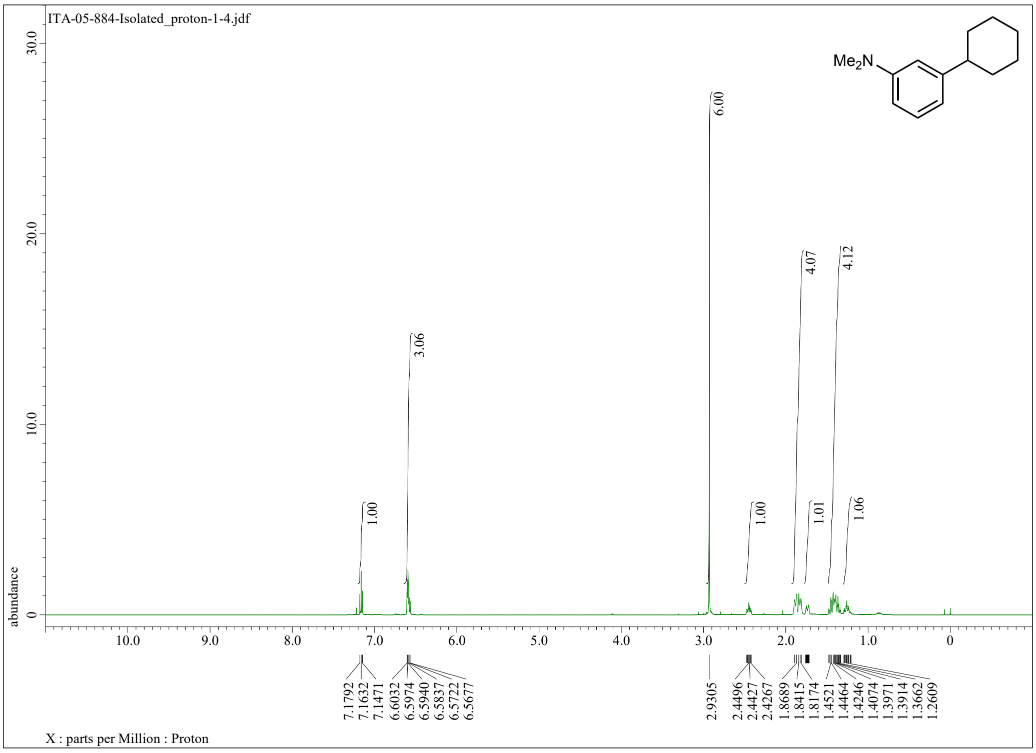

Supplementary Figure 106. ^{1}H NMR spectrum of **41** (500 MHz, $CDCl_3$).

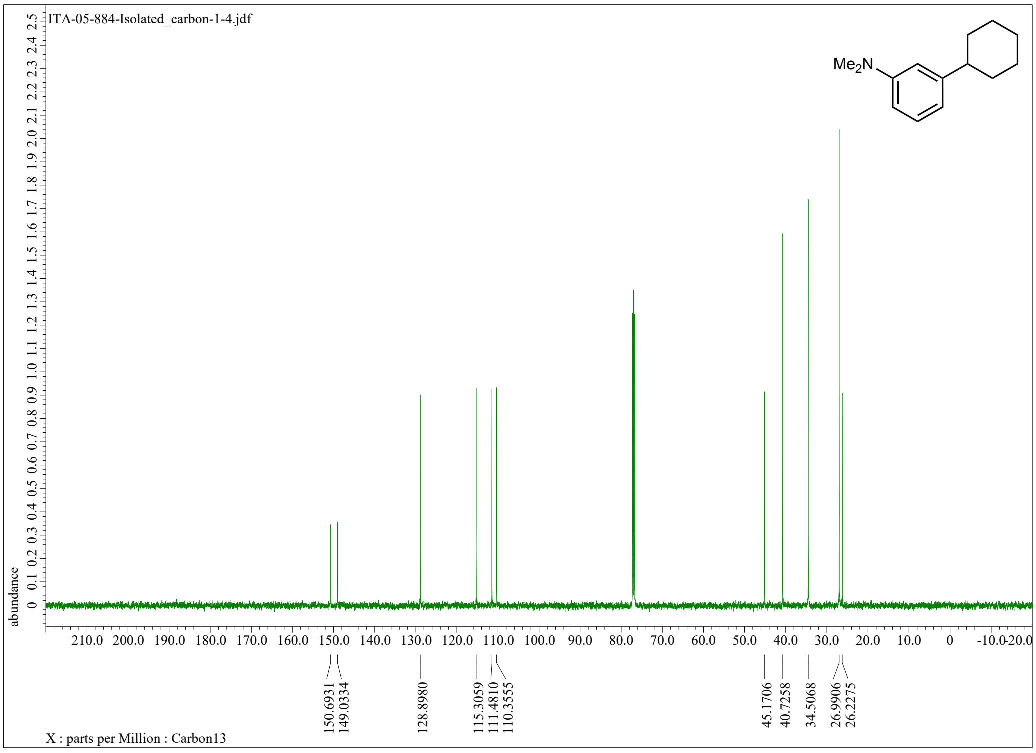

Supplementary Figure 107. ^{13}C NMR spectrum of **41** (126 MHz, $CDCl_3$).

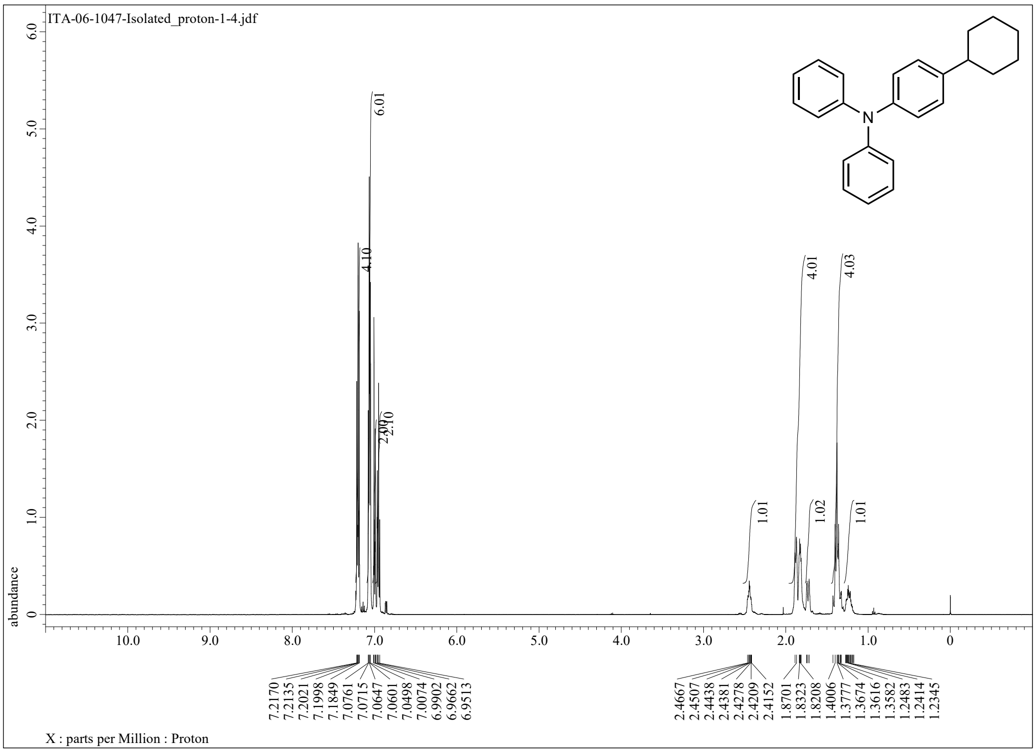

Supplementary Figure 108. ^{1}H NMR spectrum of **42** (500 MHz, $CDCl_3$).

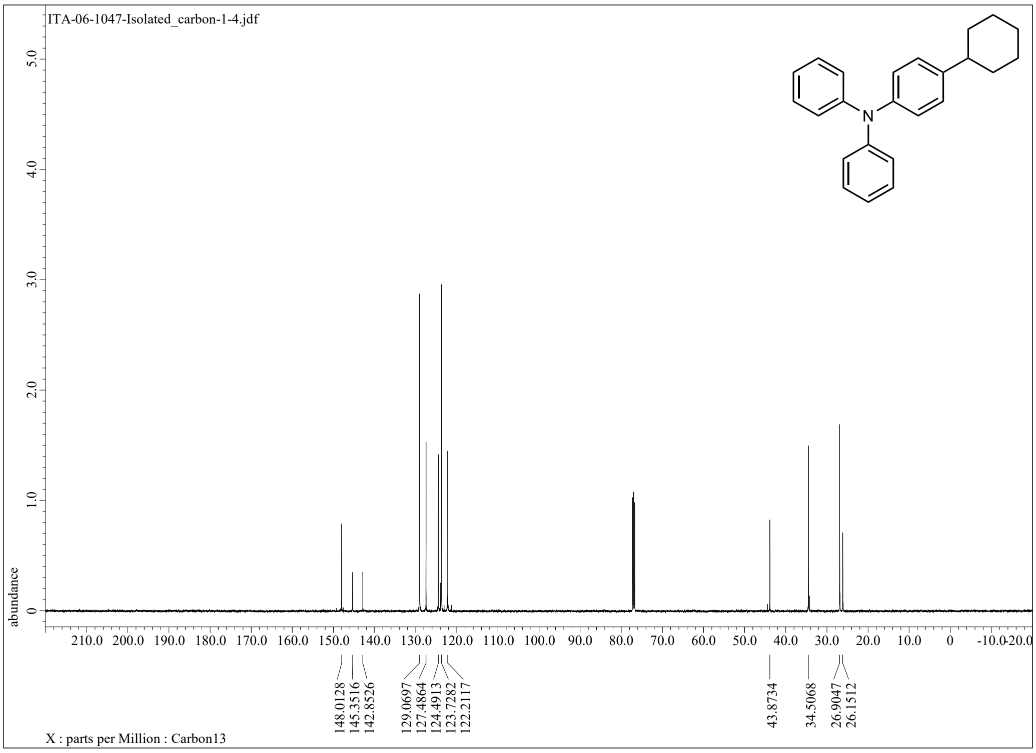

Supplementary Figure 109. ^{13}C NMR spectrum of **42** (126 MHz, $CDCl_3$).

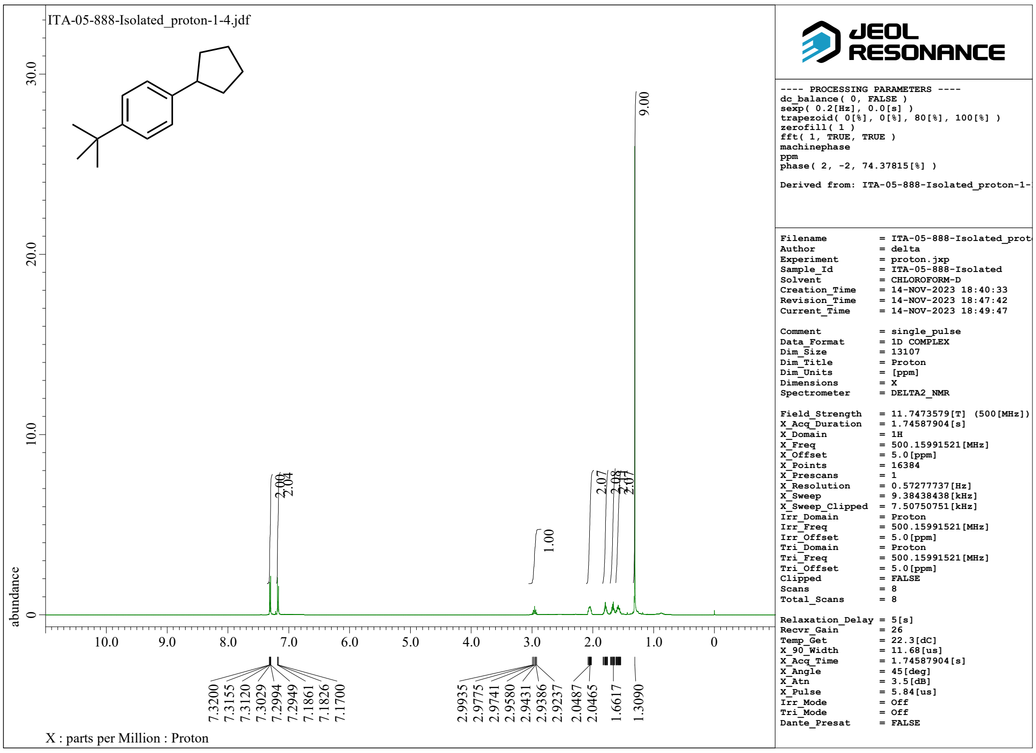

Supplementary Figure 110. ^{1}H NMR spectrum of **43** (500 MHz, $CDCl_3$).

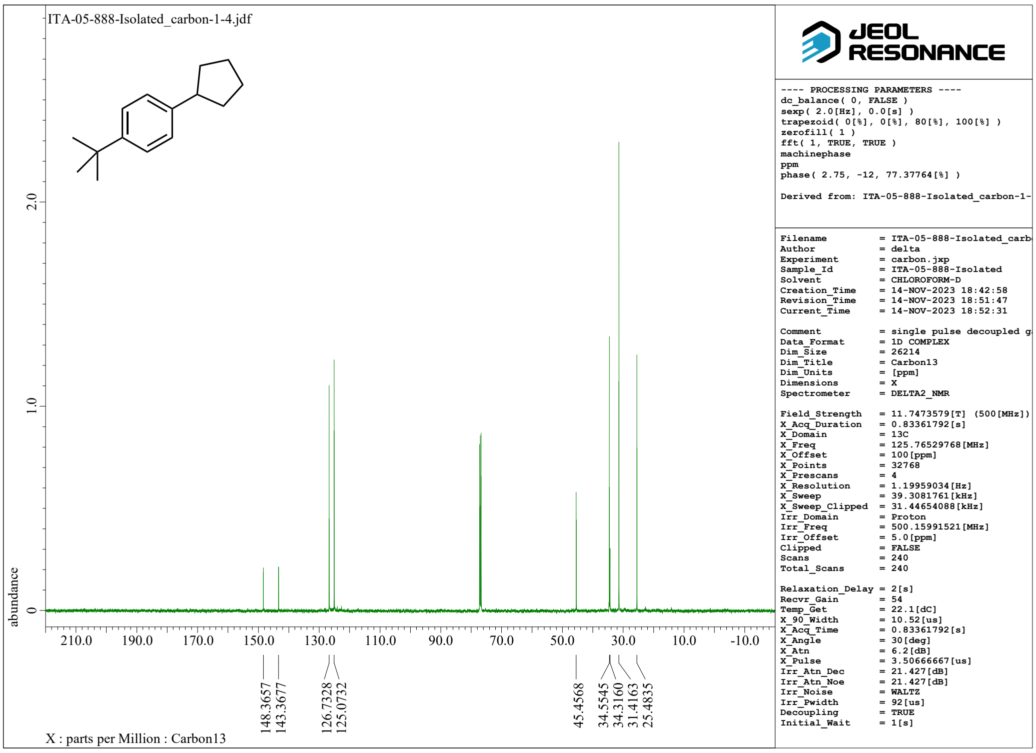

Supplementary Figure 111. ^{13}C NMR spectrum of **43** (126 MHz, $CDCl_3$).

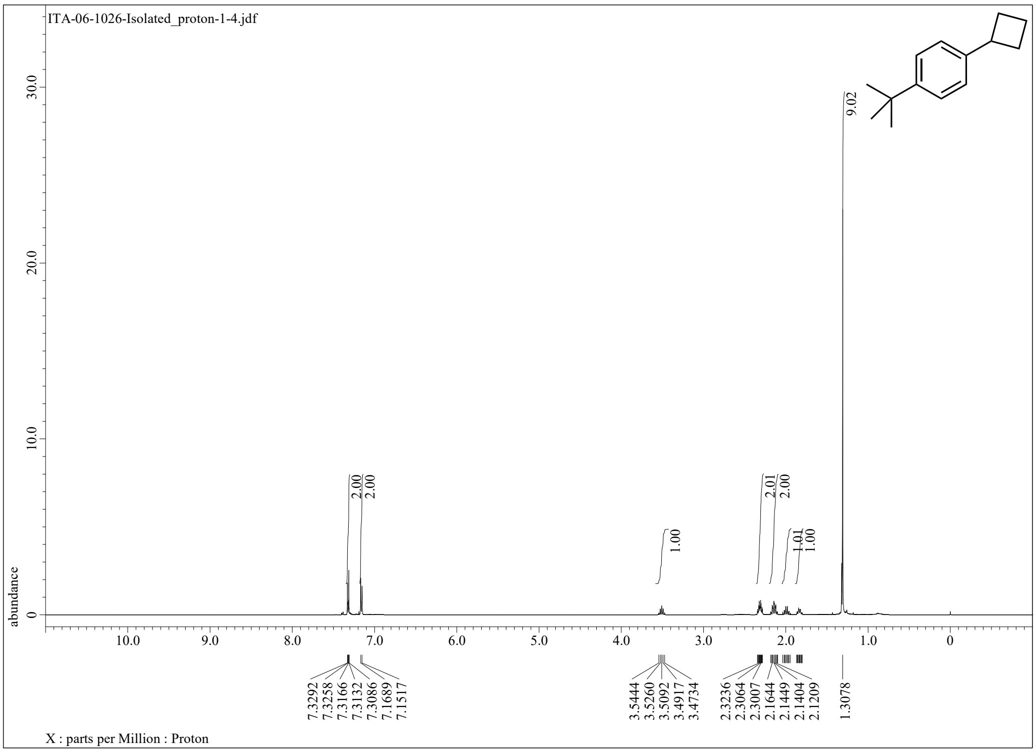

Supplementary Figure 112. ^{1}H NMR spectrum of **44** (500 MHz, $CDCl_3$).

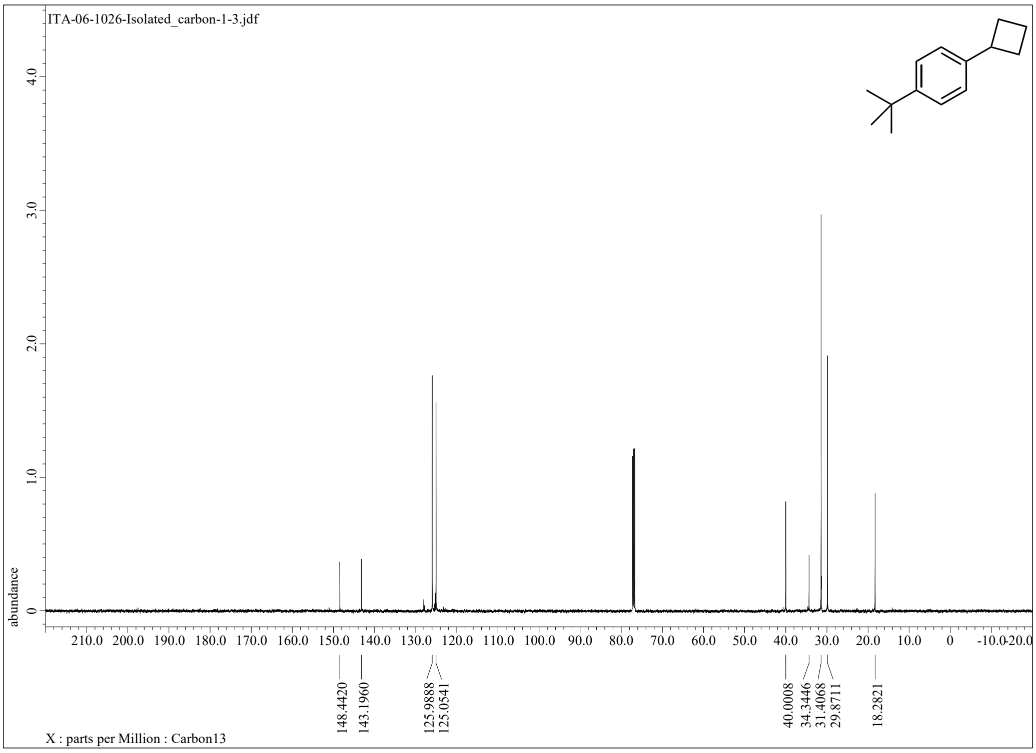

Supplementary Figure 113. ^{13}C NMR spectrum of **44** (126 MHz, $CDCl_3$).

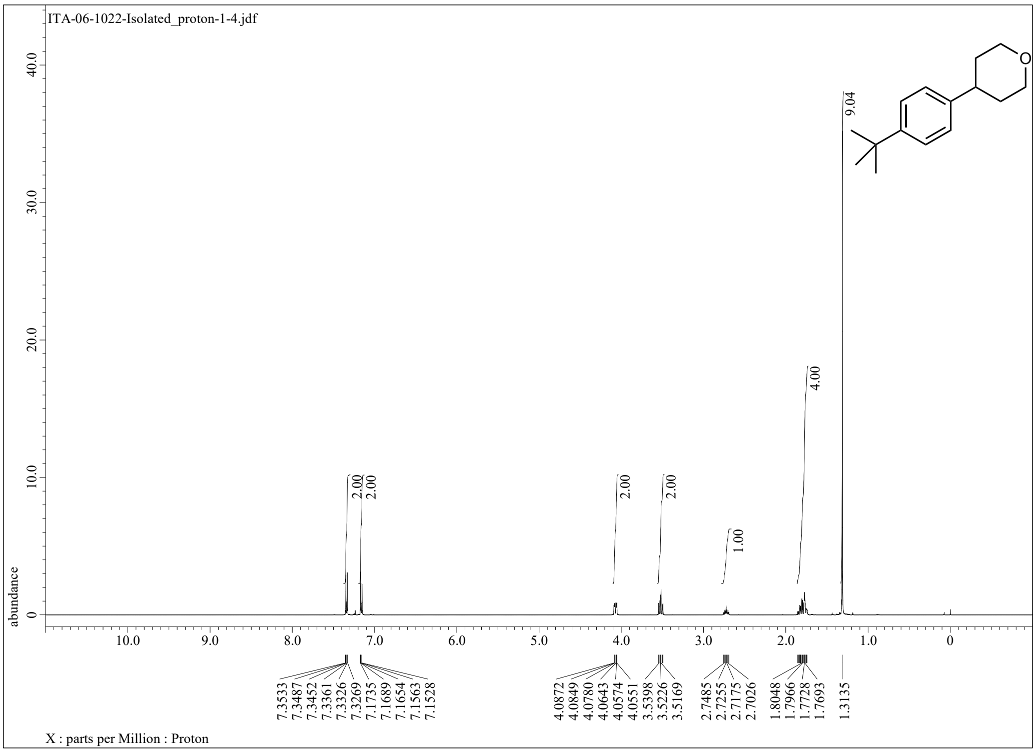

Supplementary Figure 114. ^{1}H NMR spectrum of **45** (500 MHz, $CDCl_3$).

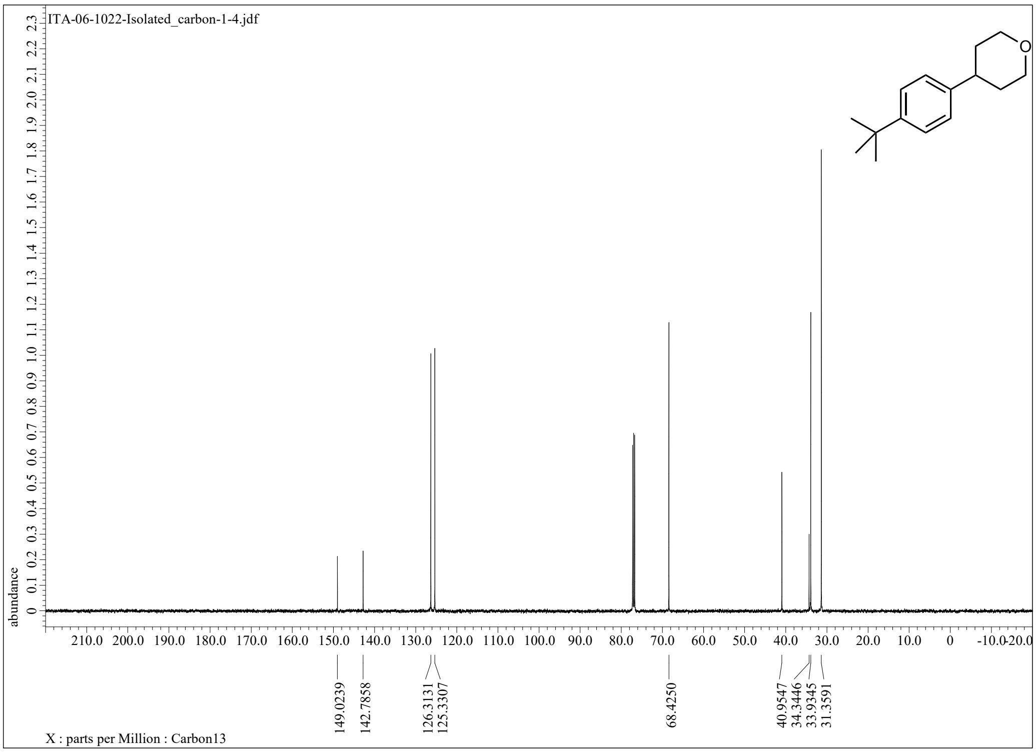

Supplementary Figure 115. ^{13}C NMR spectrum of **45** (126 MHz, $CDCl_3$).

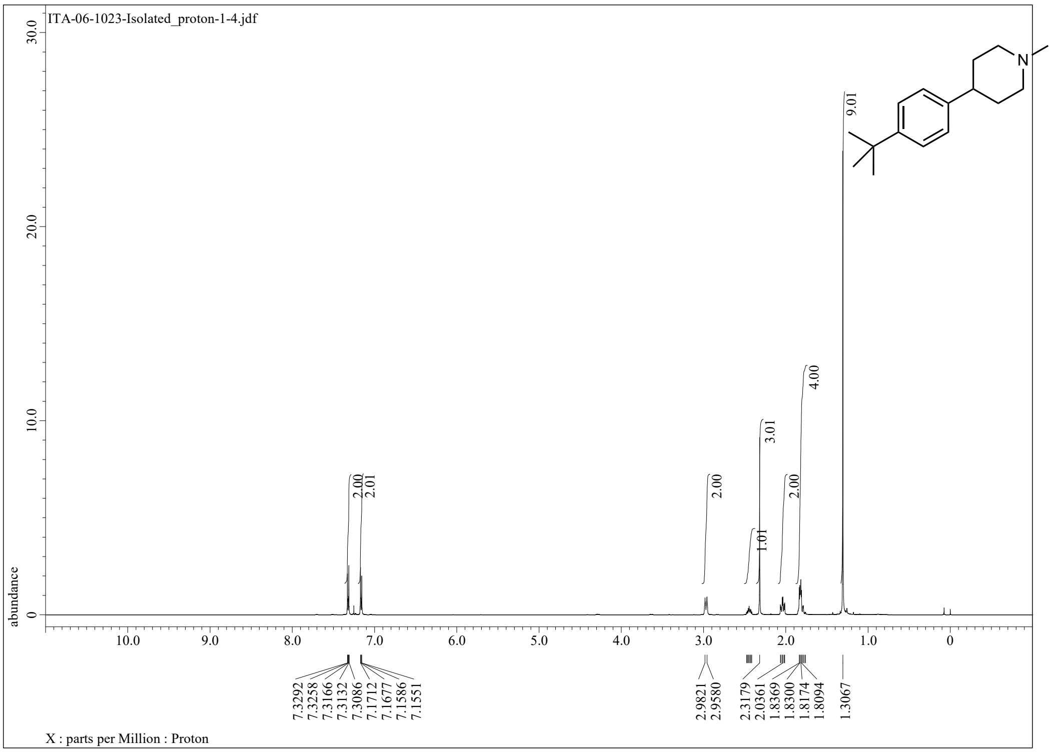

Supplementary Figure 116. ^{1}H NMR spectrum of **46** (500 MHz, $CDCl_3$).

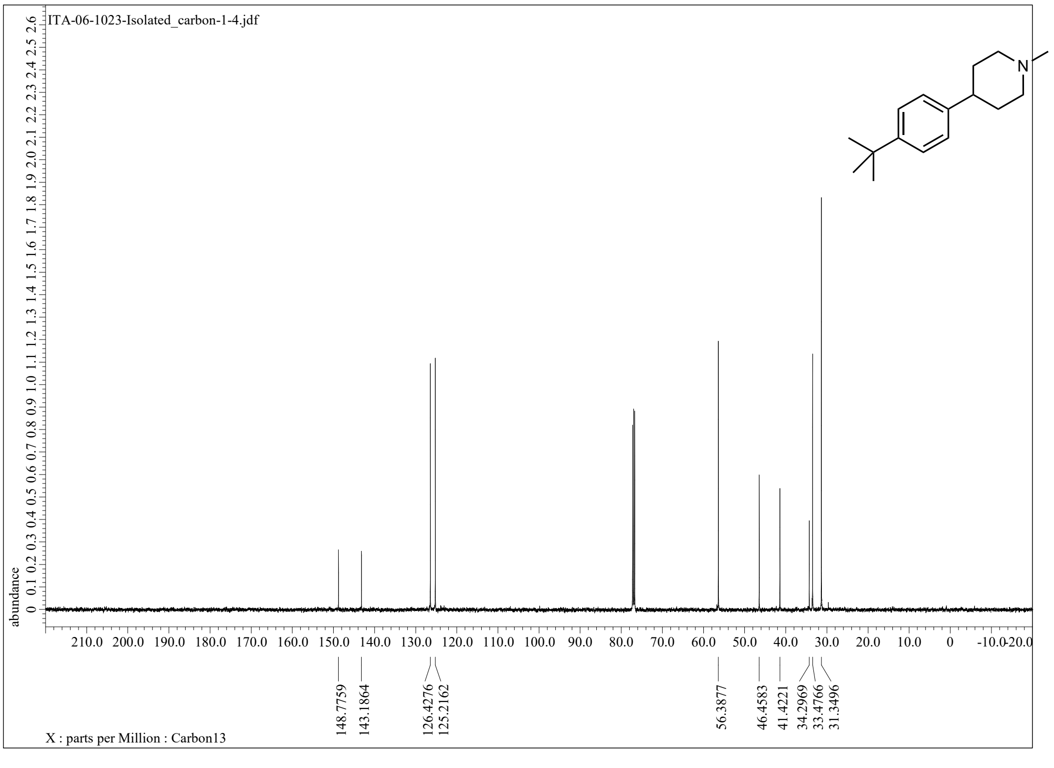

Supplementary Figure 117. ^{13}C NMR spectrum of **46** (126 MHz, $CDCl_3$).

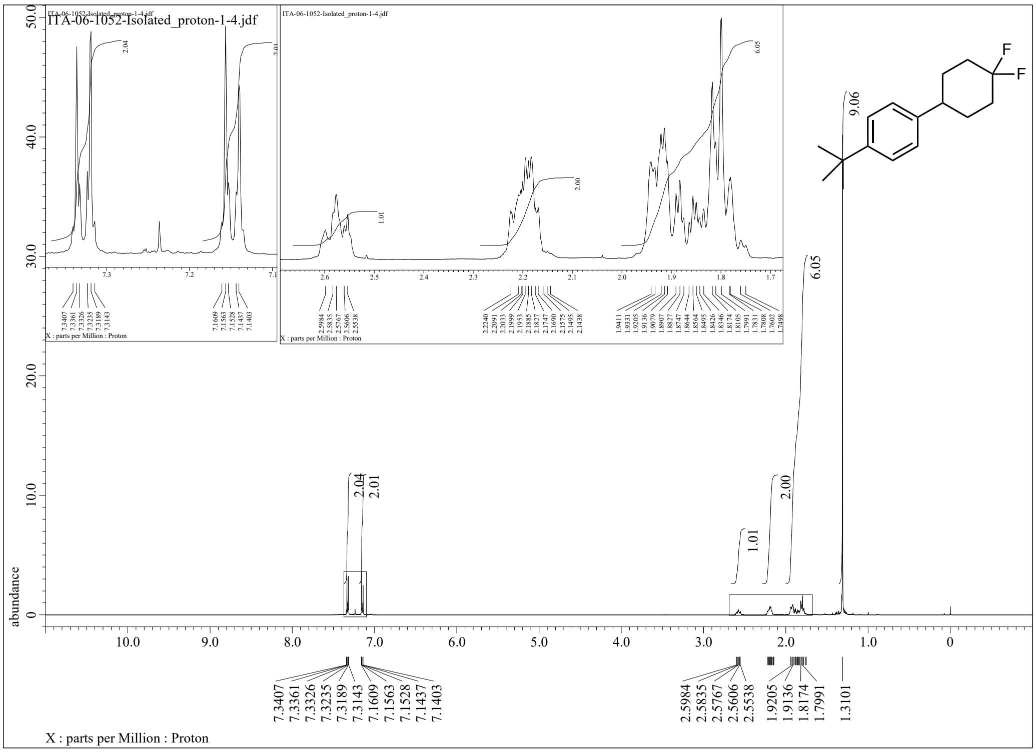

Supplementary Figure 118. ^{1}H NMR spectrum of **47** (500 MHz, $CDCl_3$).

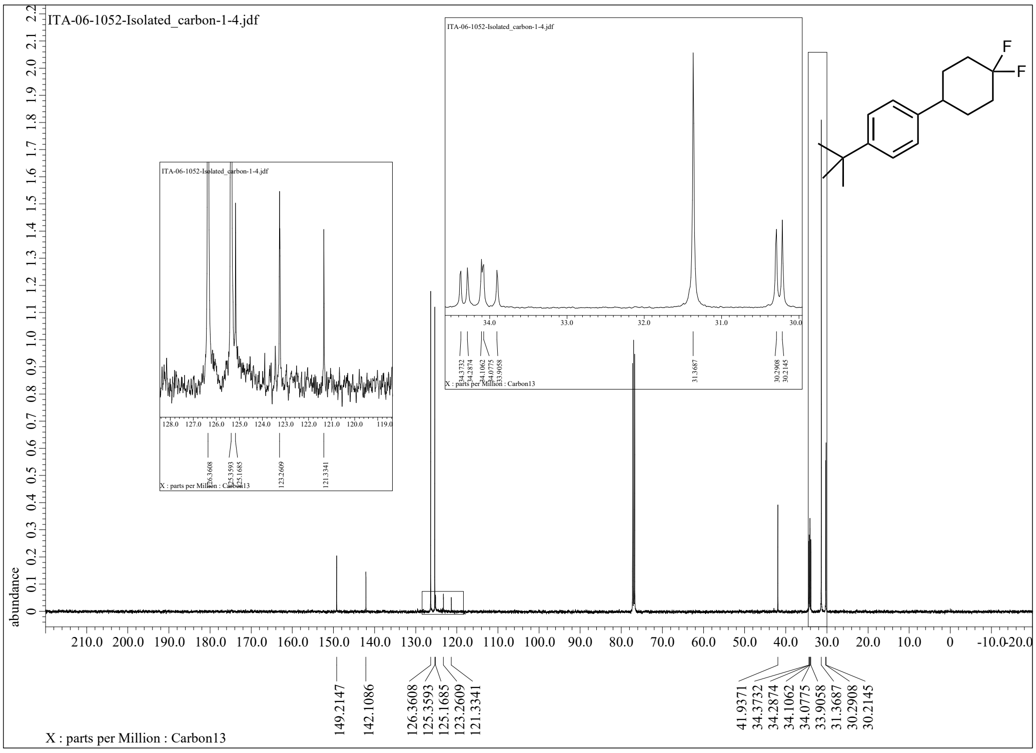

Supplementary Figure 119. ^{13}C NMR spectrum of **47** (126 MHz, $CDCl_3$).

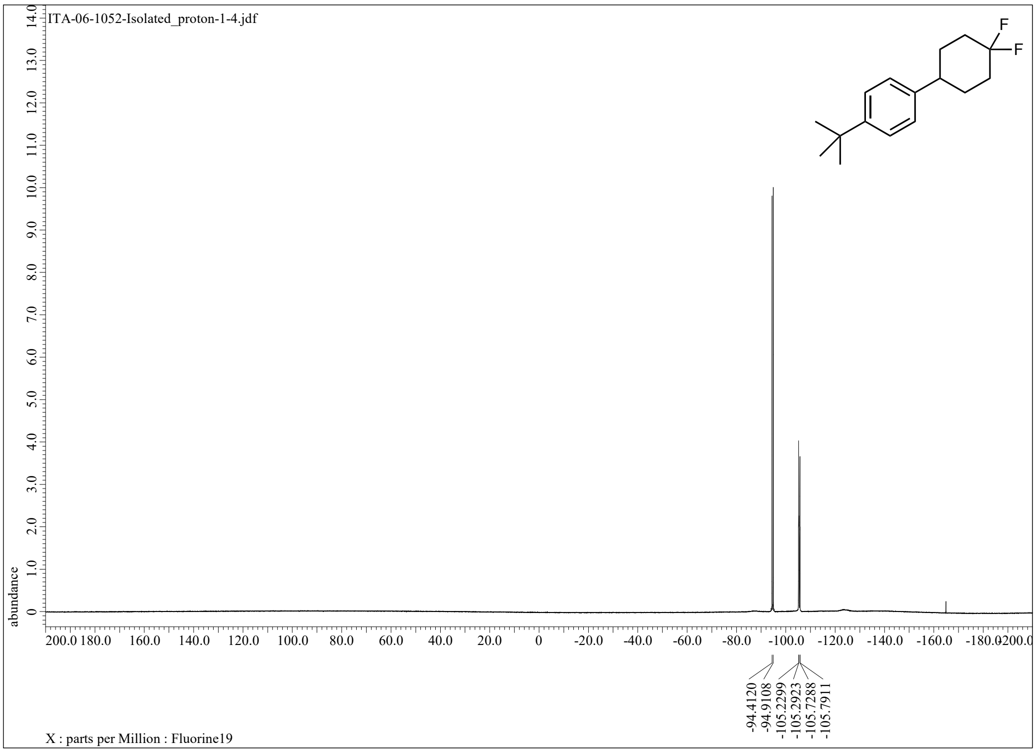

Supplementary Figure 120. ^{19}F NMR spectrum of **47** (470 MHz, $CDCl_3$).

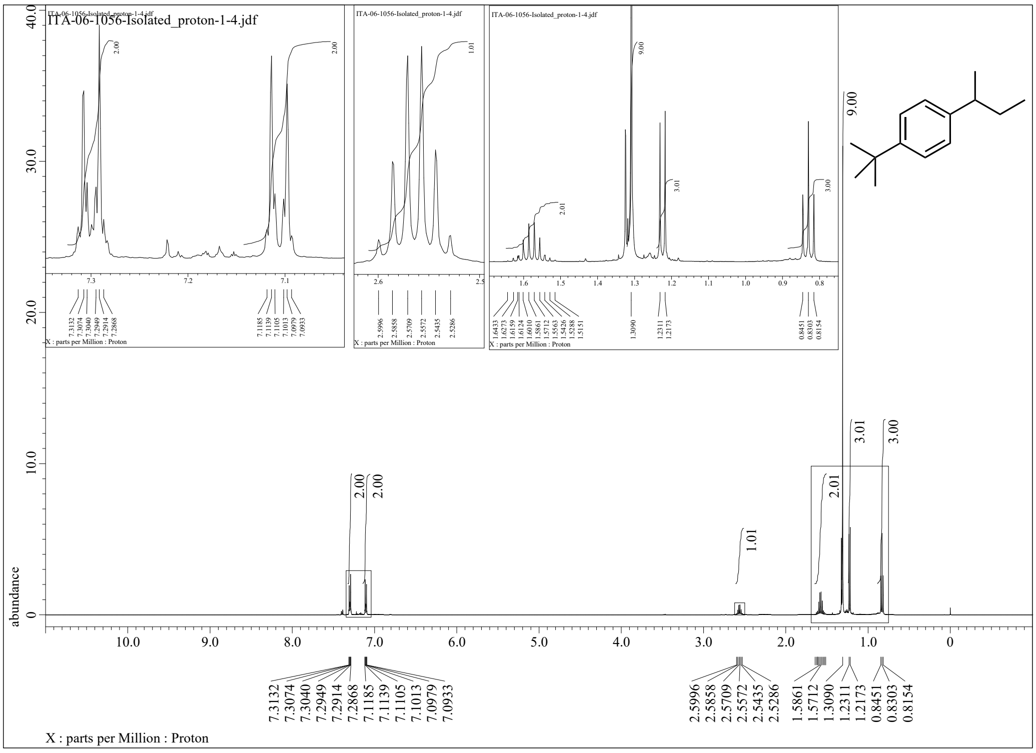

Supplementary Figure 121. ^{1}H NMR spectrum of **48** (500 MHz, $CDCl_3$).

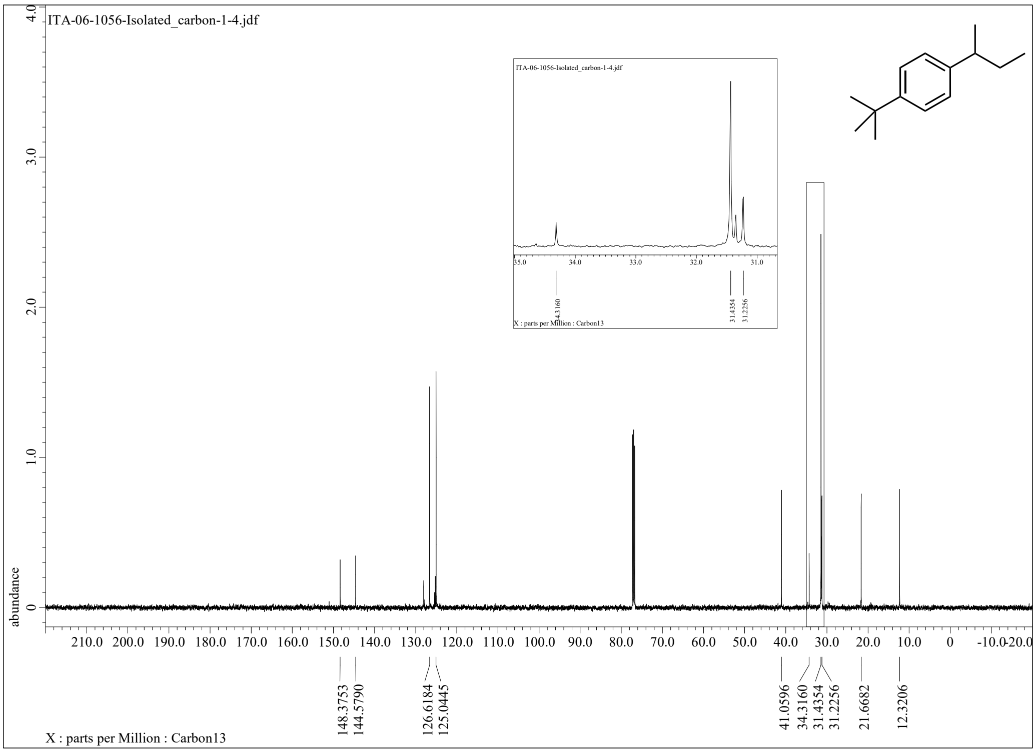

Supplementary Figure 122. ^{13}C NMR spectrum of **48** (126 MHz, $CDCl_3$).

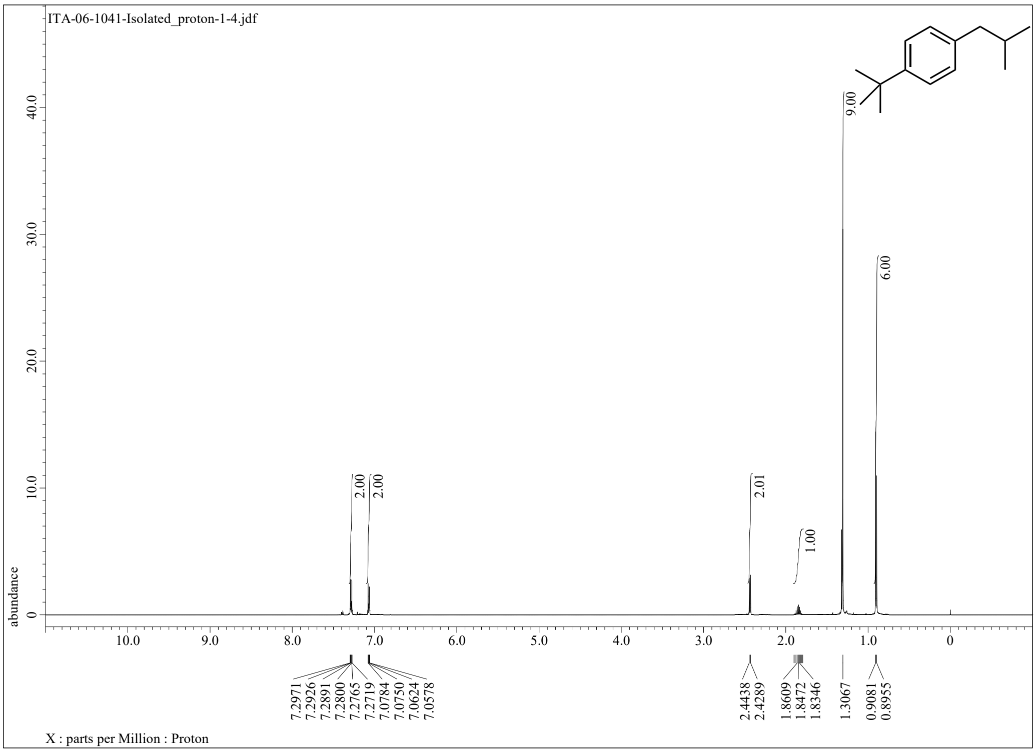

Supplementary Figure 123. ^{1}H NMR spectrum of **49** (500 MHz, $CDCl_3$).

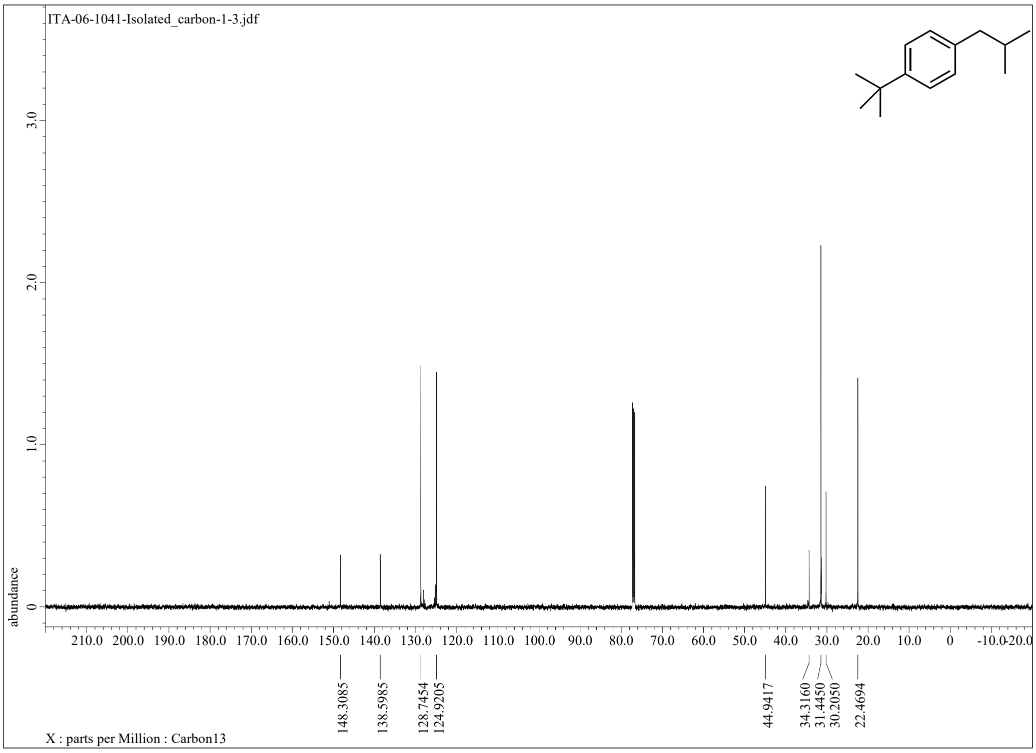

Supplementary Figure 124. ^{13}C NMR spectrum of **49** (126 MHz, $CDCl_3$).

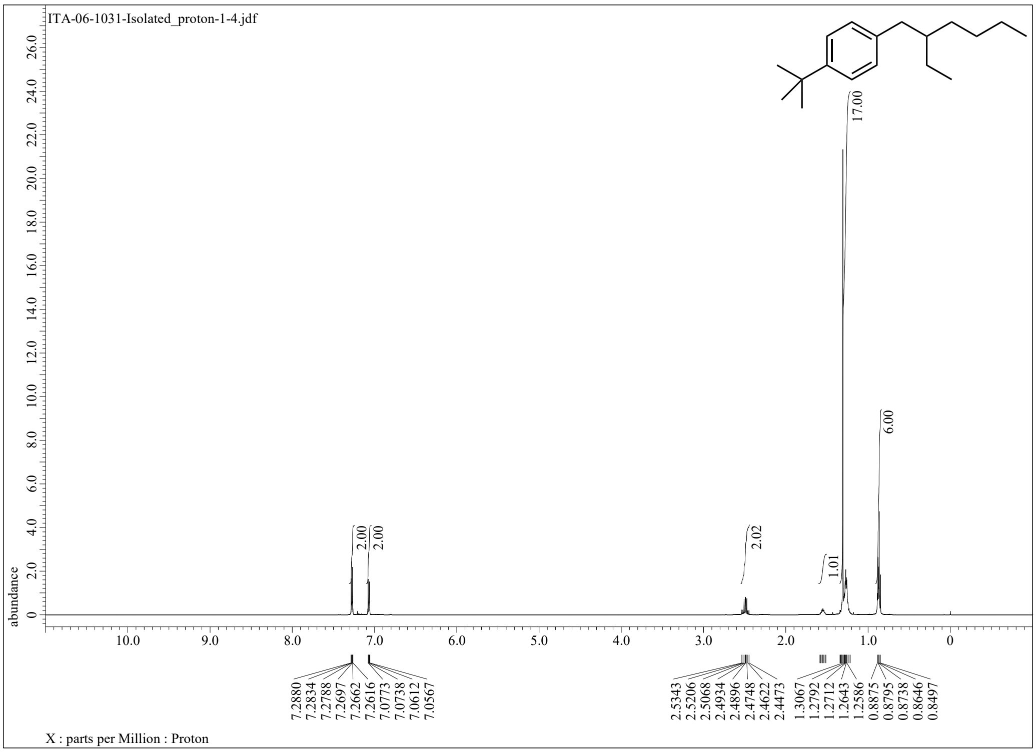

Supplementary Figure 125. ^{1}H NMR spectrum of **50** (500 MHz, $CDCl_3$).

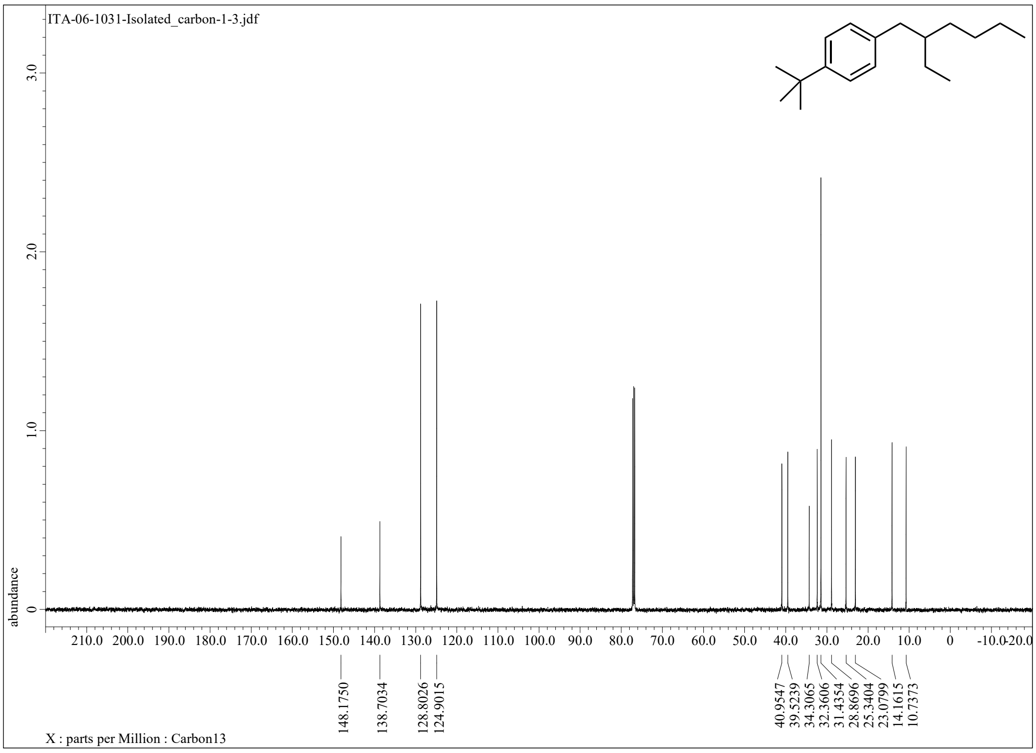

Supplementary Figure 126. ^{13}C NMR spectrum of **50** (126 MHz, $CDCl_3$).

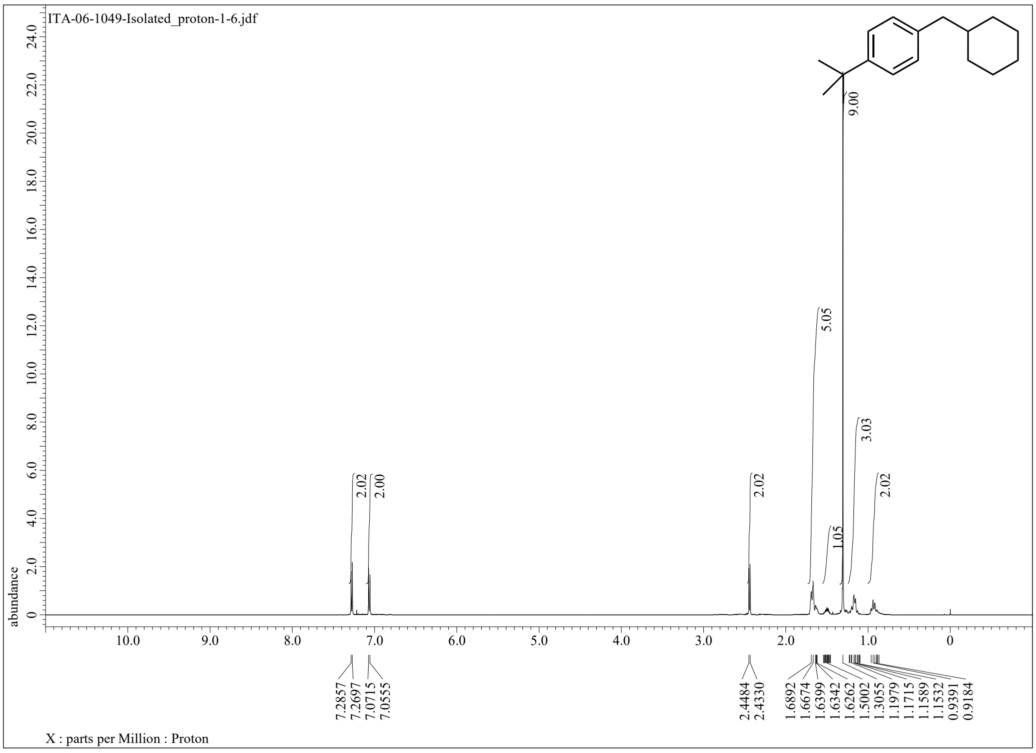

Supplementary Figure 127. ^{1}H NMR spectrum of **51** (500 MHz, $CDCl_3$).

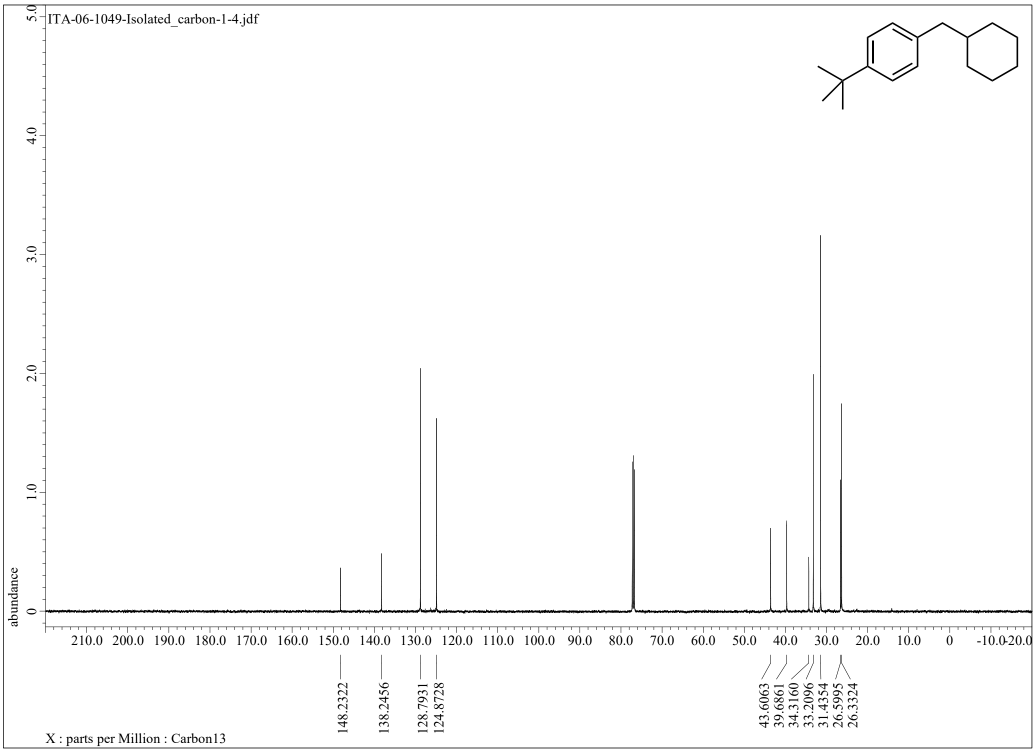

Supplementary Figure 128. ^{13}C NMR spectrum of **51** (126 MHz, $CDCl_3$).

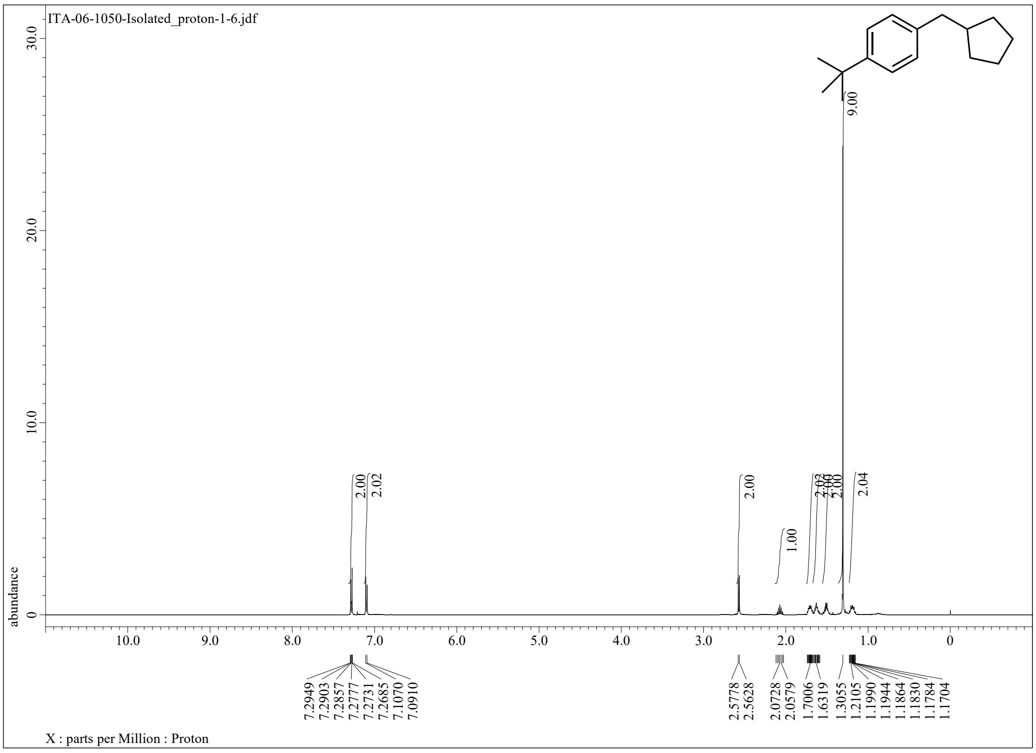

Supplementary Figure 129. ^{1}H NMR spectrum of **52** (500 MHz, $CDCl_3$).

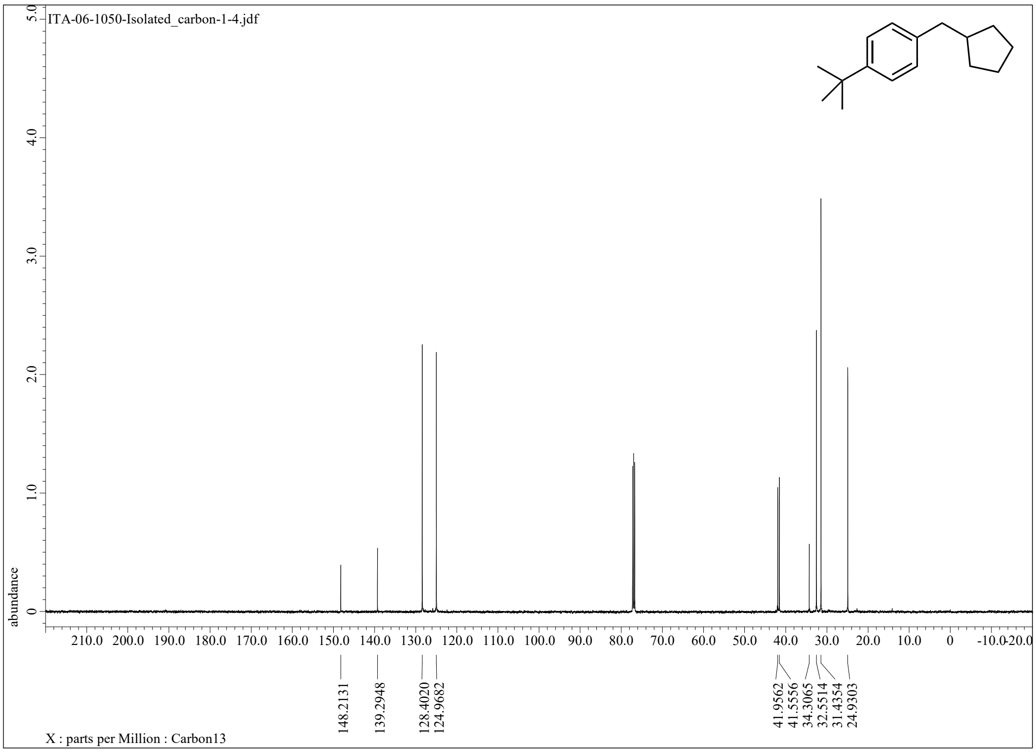

Supplementary Figure 130. ^{13}C NMR spectrum of **52** (126 MHz, $CDCl_3$).

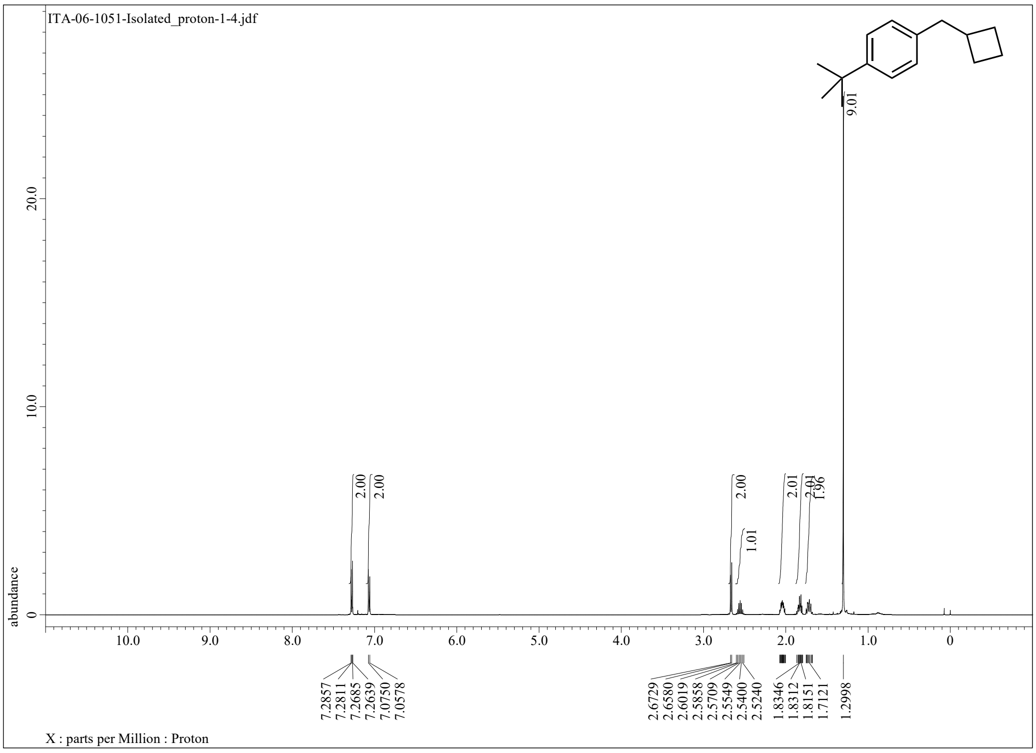

Supplementary Figure 131. ^{1}H NMR spectrum of **53** (500 MHz, $CDCl_3$).

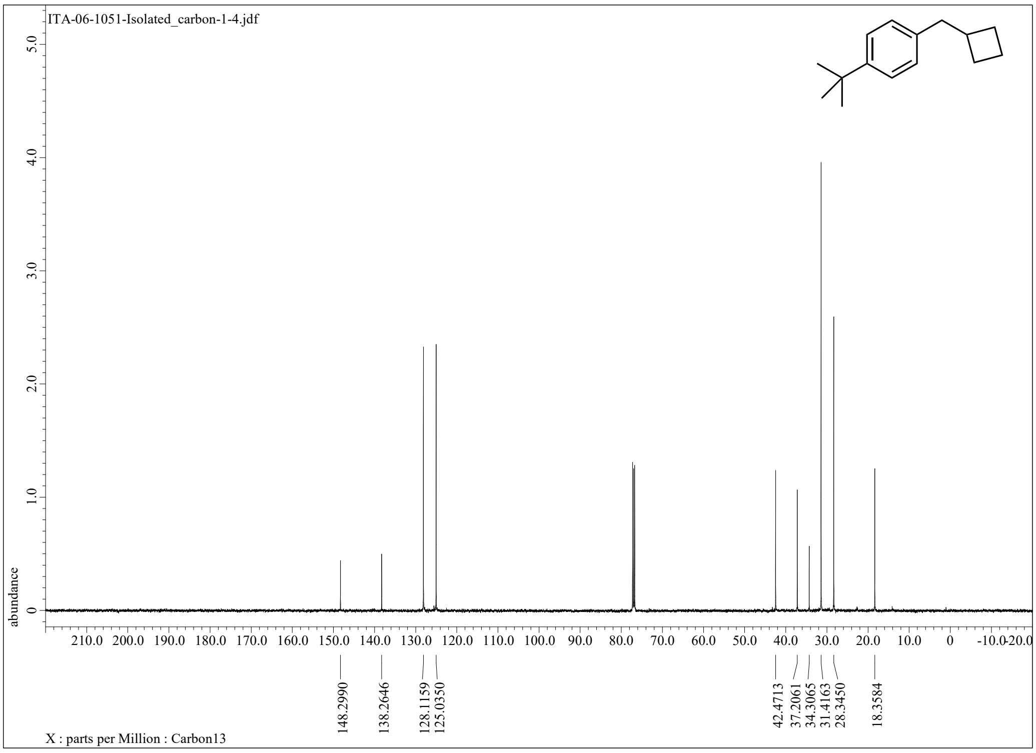

Supplementary Figure 132. ^{13}C NMR spectrum of **53** (126 MHz, $CDCl_3$).

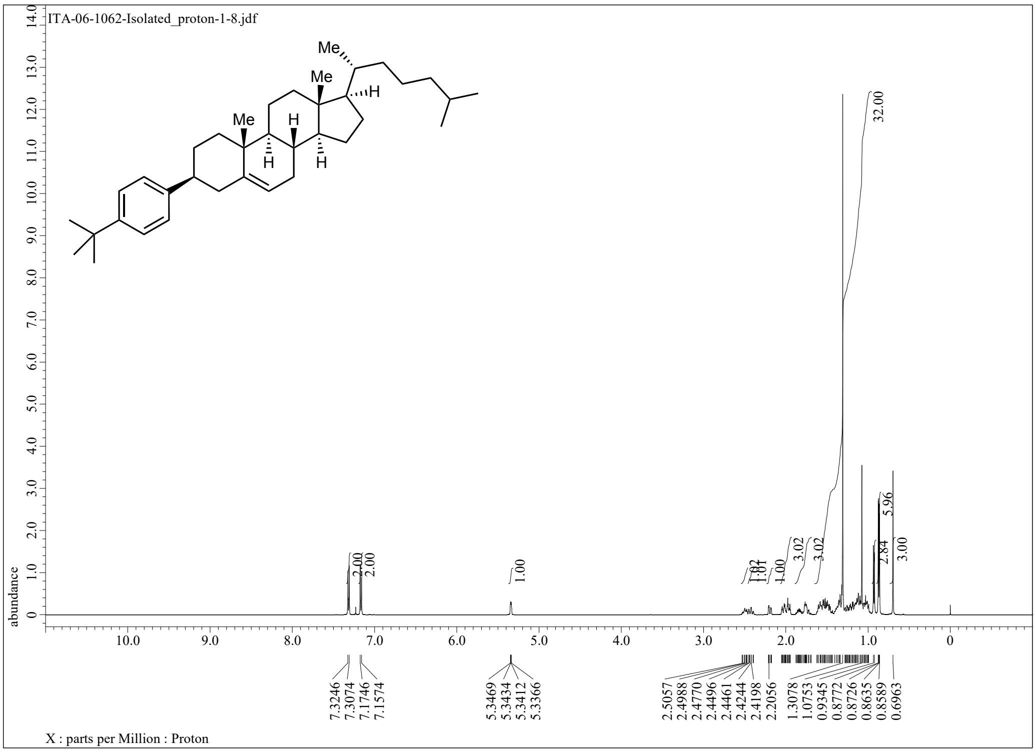

Supplementary Figure 133. ^{1}H NMR spectrum of **55** (500 MHz, $CDCl_3$).

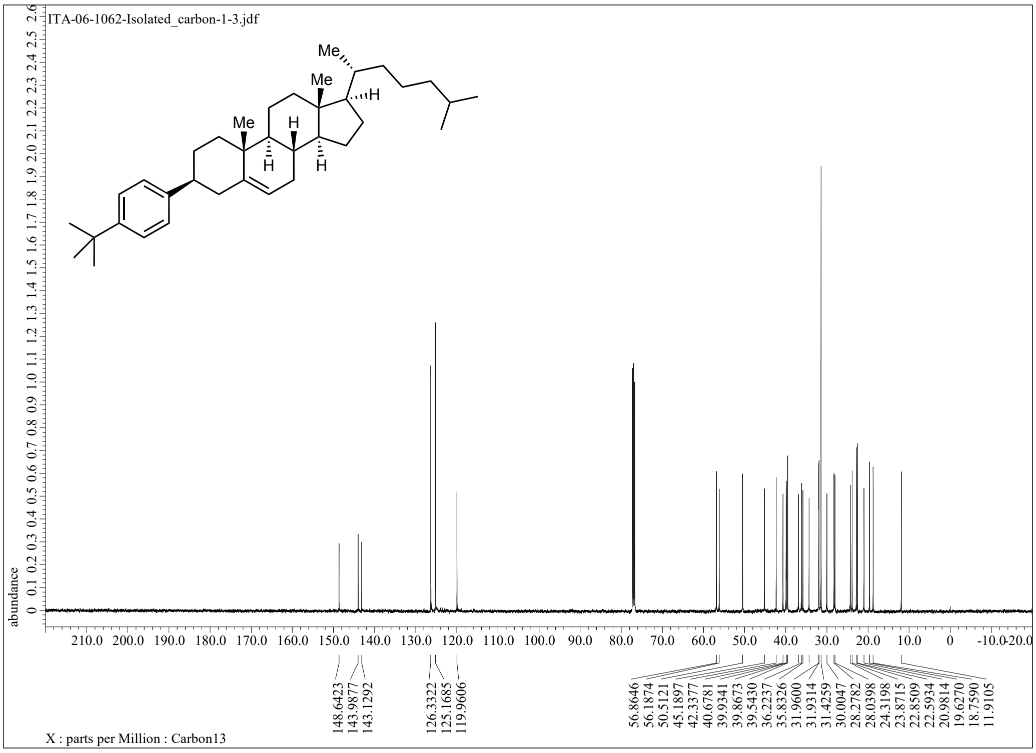

Supplementary Figure 134. ^{13}C NMR spectrum of **55** (126 MHz, $CDCl_3$).

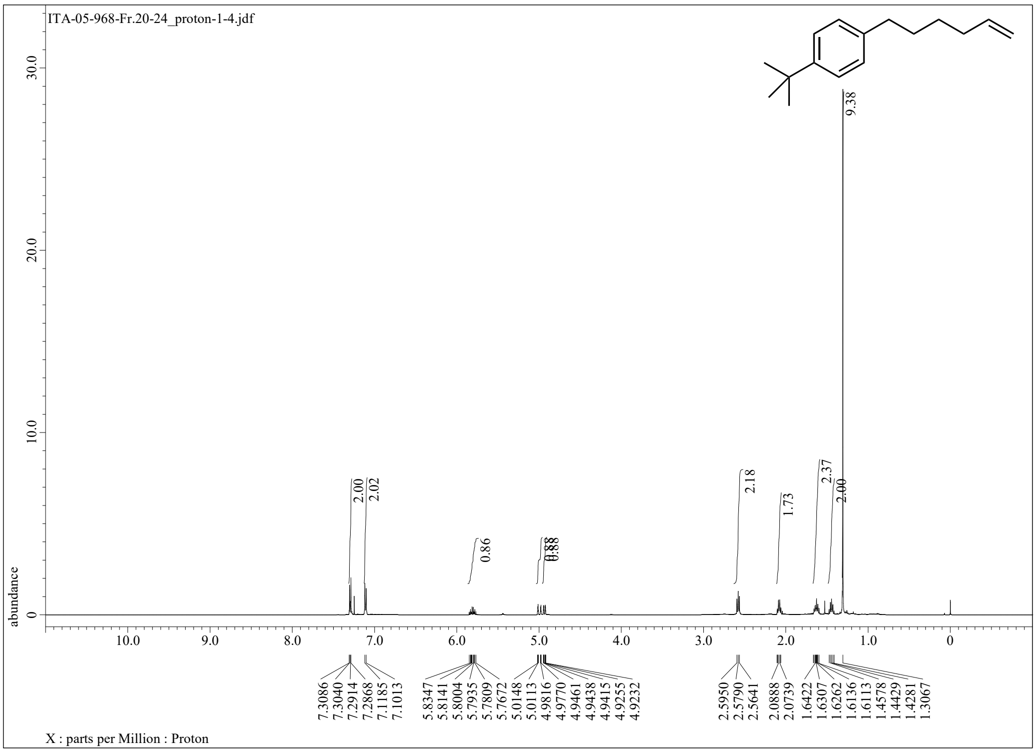

Supplementary Figure 135. ^{1}H NMR spectrum of **58** (500 MHz, $CDCl_3$).

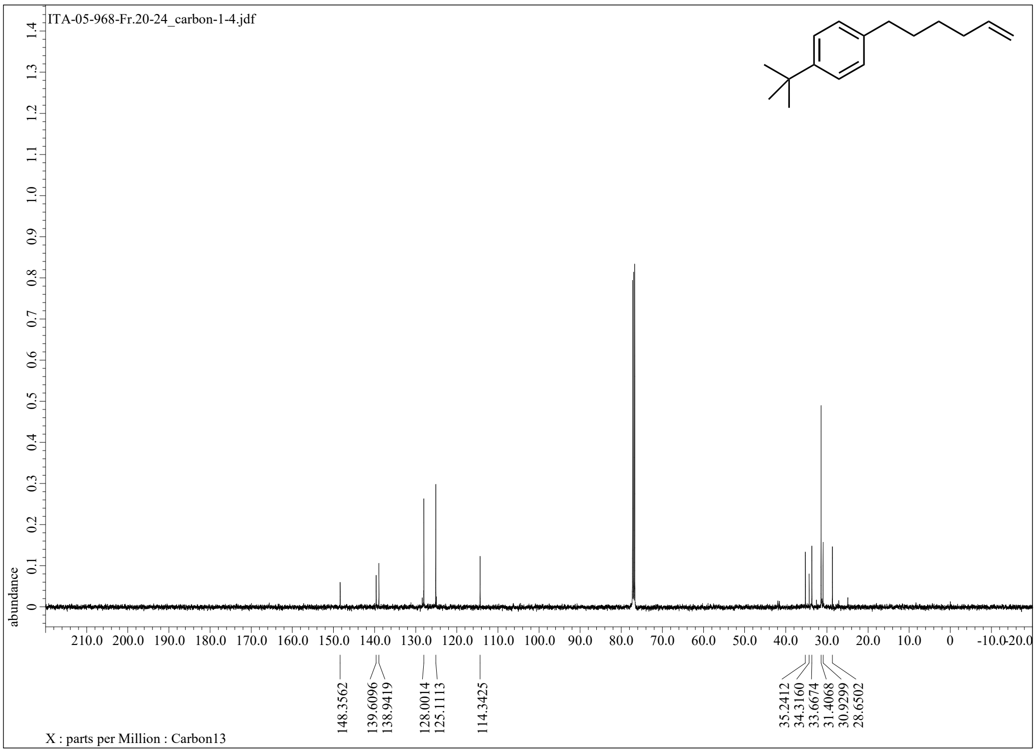

Supplementary Figure 136. ^{13}C NMR spectrum of **58** (126 MHz, $CDCl_3$).
